# US Tobacco 21 Policies and Potential Mortality Reductions by State

**DOI:** 10.1001/jamahealthforum.2024.4445

**Published:** 2024-12-20

**Authors:** Jamie Tam, Alyssa Crippen, Abigail Friedman, Jihyoun Jeon, David C. Colston, Nancy L. Fleischer, Catherine A. Vander Woude, Megan A. Boelter, Theodore R. Holford, David T. Levy, Rafael Meza

**Affiliations:** 1 Department of Health Policy and Management, Yale School of Public Health, New Haven, Connecticut; 2 Department of Epidemiology, University of Michigan School of Public Health, Ann Arbor; 3 Department of Health Behavior, University of North Carolina Gillings School of Global Public Health, Chapel Hill; 4 Preventing Tobacco Addiction Foundation / Tobacco 21; 5 Department of Biostatistics, Yale School of Public Health, New Haven, Connecticut; 6 Lombardi Comprehensive Cancer Center, Georgetown University, Washington, DC; 7 Integrative Oncology, BC Cancer Research Centre, Vancouver, British Columbia, Canada

## Abstract

**Importance:**

Research shows that Tobacco 21 (T21) policies with a minimum legal access age for tobacco products of 21 years reduce smoking, yet their impact varies across US states due to differences in smoking behaviors, mortality rates, and policy coverage.

**Objective:**

To quantify potential reductions in smoking-attributable mortality associated with Tobacco 21 policies for each of the 50 states and Washington, DC.

**Design, Setting, and Participants:**

The Cancer Intervention Surveillance and Modeling Network (CISNET) Tobacco Control Policy Model of smoking was used with detailed state-specific data on smoking initiation, smoking cessation and mortality rates as they vary by age, gender, and birth cohort for 50 US states and the District of Columbia. This was integrated with comprehensive data on T21 policy coverage at the local and state levels from 2005 to 2024, and then T21 policy effects from quasi-experimental studies were applied to simulate each state’s experience of T21 policies, quantifying potential mortality reductions from 2005 to 2100.

**Exposures:**

Cigarette smoking.

**Main Outcomes and Measures:**

Estimated smoking-attributable deaths averted and life-years gained compared with a baseline scenario.

**Results:**

Early statewide T21 adoption in California, combined with its large population, was associated with more than 27 000 premature deaths averted through 2100, whereas statewide implementation in Kentucky was associated with 15 000 averted premature deaths. In Massachusetts, T21 policies were associated with 8000 averted premature deaths, largely attributed to municipal T21 policies covering most residents. Wisconsin—lacking state or local policies—requires enforcement of federal T21 to prevent up to 10 000 premature deaths. Across the country, comprehensive enforcement of federal, state, and local T21 laws were associated with up to 526 000 premature deaths averted and 13.3 million life-years gained by 2100. Enforcement of only state and local policies was associated with 442 000 premature deaths averted.

**Conclusions and Relevance:**

This study found that early adoption and implementation of T21 policies maximizes potential premature mortality reductions. However, the strength of T21 policies and enforcement varies widely across states. Enforcement of the federal T21 law is critical in the 8 states without state-level T21 cigarette policies of their own.

## Key Points

**Question:**

What are the estimated reductions to smoking-attributable mortality associated with Tobacco 21 policies across the US?

**Findings:**

State-specific Tobacco 21 policy data and simulation models of cigarette smoking used to estimate that comprehensive enforcement of local, state, and federal Tobacco 21 laws were associated with up to 526 000 premature deaths averted and 13.3 million life-years gained across the US by 2100. Mortality reductions varied widely by state.

**Meaning:**

These findings quantify potential mortality reductions according to each state’s historical smoking, mortality, and Tobacco 21 policy profile; early adoption and strong enforcement of Tobacco 21 laws may maximize health benefits.

## Teaser

This decision simulation model quantifies potential reductions in smoking-attributable mortality associated with Tobacco 21 policies for each of the 50 states and Washington, DC, in the US.

## Introduction

In 2015, the National Academy of Medicine (NAM) report, “Public Health Implications of Raising the Minimum Age of Legal Access to Tobacco Products,”^1^ estimated the health benefits of raising the minimum age of legal access to tobacco products to 21 years—also known as Tobacco 21 (T21). Without other evidence on the policy’s effects, the commissioned modeling analyses relied on expert opinions about the likely effects of a national T21 policy. The analysis projected that a federal T21 law would avert 249 000 premature smoking-attributable deaths through 2100. However, the impact of US T21 policies as they have been implemented at the local, state, and federal levels has never been estimated.

When the NAM report was released, only 30 municipalities had T21 policies, driven by the emerging movement that began in Needham, Massachusetts in 2005.^1,2^ Hawaii became the first state to implement a statewide law in 2016, and was soon followed by California. These early adopters were joined by 14 states and 540 localities^3^ before the federal T21 law was signed in December 2019. However, enforcement of federal law is limited and relies heavily on the efforts of the states themselves. As of May 2024, 8 states do not have state-level T21 laws applied to cigarettes (Alaska, Arizona, Missouri, Mississippi, Montana, North Carolina, South Carolina, and Wisconsin; Mississippi’s T21 law only applies to e-cigarettes).^4,5,6^ Thus, the benefits of T21 policies to those states hinge on enforcement of federal law alone.

T21 impacts depend on both the timing of policy implementation and underlying population health trends. For example, state policies’ effects may be dampened in states where local and county T21 policies already cover large shares of the population. Weaknesses in T21 legislation and enforcement may also dampen policies’ effects.^4,7^ States with higher smoking rates, higher mortality, and lower life expectancy may have more to gain from T21 policies than those with healthier populations. Because national analysis obscures these important geographic differences, state-level analyses are needed to quantify T21 effects and elucidate the consequences of heterogeneous coverage and enforcement.

Numerous studies have concluded that T21 laws reduce smoking among young people in the short term.^8,9,10^ These findings, combined with comprehensive data about local and state policies^11,12,13^ provide a unique opportunity to project the long-term impacts of US T21 policies. We simulate T21 policies accounting for the unique smoking, mortality, and policy coverage profiles of 50 states and the District of Columbia. Results estimate the relative contributions of local, state, and federal T21 policies on reductions to premature mortality.

## Methods

### State Models

The Yale institutional review board determined this study to be exempt from review as no human participants were involved. We adapted the Cancer Intervention and Surveillance Modeling Network (CISNET) Tobacco Control Policy Model (previously known as the CISNET Smoking History Generator Population Model)—a deterministic compartmental Markov model—for the 50 US states and the District of Columbia using state-specific estimates of smoking initiation and cessation probabilities by age, gender, and birth cohort.^14,15^ Birth cohorts were simulated from 1908 to 2100. Individuals were considered to begin with never smoking stustus and could transition to current smoking each year; once smoking, individuals’ status could transition to former smoking each year. Exit from the population occured through mortality probabilities by smoking status and years since quitting, or on reaching age 99 years. Mortality probabilities and life expectancies were specific to age, gender, smoking status, and birth cohort. Among people who quit smoking, mortality probabilities varied by years since quitting. Each state model was validated against the Tobacco Use Supplement to the Current Population Survey (TUS-CPS) 1992 to 2019 data^16^ to ensure the reproduction of smoking prevalence estimates over time. State population sizes were based on US Census Bureau estimates^17,18^ (eTable 1 in Supplement 1).

### Policy Effects

The main parameter capturing T21 effects was based on state-level findings from Hansen et al,^9^ which translated to a 34% reduction to smoking prevalence among persons aged 18 to 20 years (95% CI: 15%-53%) years (eTables 2 and 3 in Supplement 1). To our knowledge, this quasi-experimental study offers the strongest evidence with the largest sample size in the T21 literature. T21 effects were applied in the year following enactment as percent reductions to smoking initiation probabilities among people aged 18 to 20 years (eFigure 1 in Supplement 1). In the absence of strong evidence on the effects of local or federal T21 laws, this state policy parameter was assumed to be identical to local and federal laws. Because of the lack of information about the parameter’s distribution across states, we conducted sensitivity analyses by evaluating outcomes at the lower and upper bounds of this estimate’s 95% CI to account for varying levels of enforcement and policy strength. Consistent with the 2015 NAM report,^1^ we assumed no T21 impact on smoking cessation and policy effects were modeled as constant over time (eTable 4 in Supplement 1). For states where the prior minimum age of tobacco sales was 19 years (Alabama, Alaska, New Jersey, and Utah), initiation reductions were only applied to those aged 19 to 20 years.

### Policy Coverage

Policy data were based on the University of Michigan Tobacco 21 Population Coverage Database,^13^ the University of Missouri Tobacco Control Research Center Tobacco 21 database,^19^ and Tobacco21.org.^4^ The University of Michigan Database was updated to include localities with T21 policies from 2005 to 2022.

The policy parameter was scaled based on the percent of the population already covered through local and state implementation by the beginning of that year.^11^ For example, a state with 65% of its population covered by local T21 laws as of December 2017, would experience a 22% reduction (0.34 × 0.65) to smoking initiation probabilities for those aged 18 to 20 years starting in January 2018.

### Policy Scenarios

Given the long time horizon between changes in smoking initiation and subsequent effects on mortality, we simulated 3 policy scenarios from 2005 to 2100:

1. Local T21 coverage only (local T21), where the effects of local policies continue with no changes to state or federal policy.
2. Local and state T21 coverage (state and local T21), where the federal T21 law had no impact beyond potentially influencing subsequent adoption of state T21 policies.
3. Federal, state, and local T21 coverage (combined federal T21), where the federal T21 law was effective immediately and fully enforced on its December 2019 enactment—our most optimistic set of policy conditions.

These are compared with the baseline scenario, which assumed no T21 policy coverage. Differences between policy scenarios represented the separate contribution of state-level efforts (state and local T21  –  local T21) and federal-level efforts (combined federal T21 – state and local T21) to public health progress. Differences between policy and baseline scenarios in premature smoking-attributable deaths (SADs) and years of life lost reflect the number of SADs averted and life-years gained (LYG), respectively. Estimates are provided for the point estimate of the policy effects (34% reduction to smoking initiation), with a range representing pessimistic and optimistic projections using the lower and upper 95% CI of the policy effects (15%-53% reduction). Mortality calculations were restricted to those affected by T21 policies, from the 1985 birth cohort onwards.

Model R code is available at https://github.com/NCI-CISNET/tcp-model-code. To the extent possible, we adhered to best practice guidelines for modeling research and reporting.^20,21^

## Results

**Figure 1.**
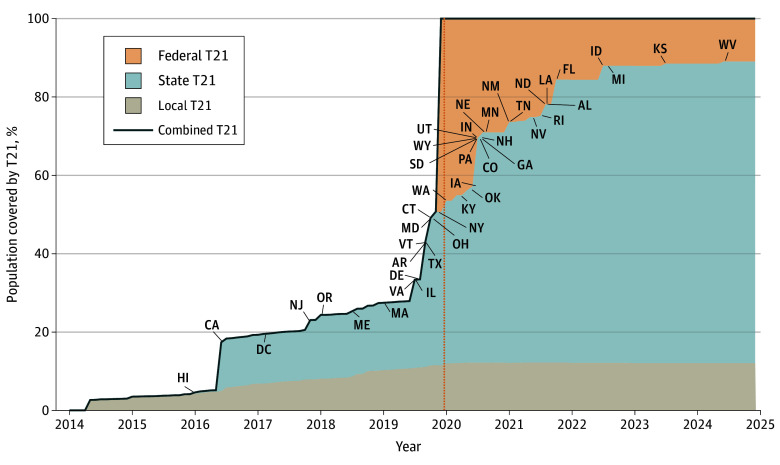
Percent of the US Resident Population Covered by Tobacco 21 (T21) Policies, 2014 to 2024 Local T21 policies are based on the percent of the population covered by local-level laws implemented between 2014 and 2020 according to the Tobacco 21 Population Coverage Database.^13^ Data from years 2005 to 2013 are not shown because only a small number of localities implemented T21 prior to 2014. State T21 policies and abbreviations reflect statewide T21 policy implementation dates based on Tobacco21.org. The federal T21 law (dotted line) was signed into law in December 2019. Combined local, state, and federal coverage is depicted by the solid black line.

**Figure 2.**
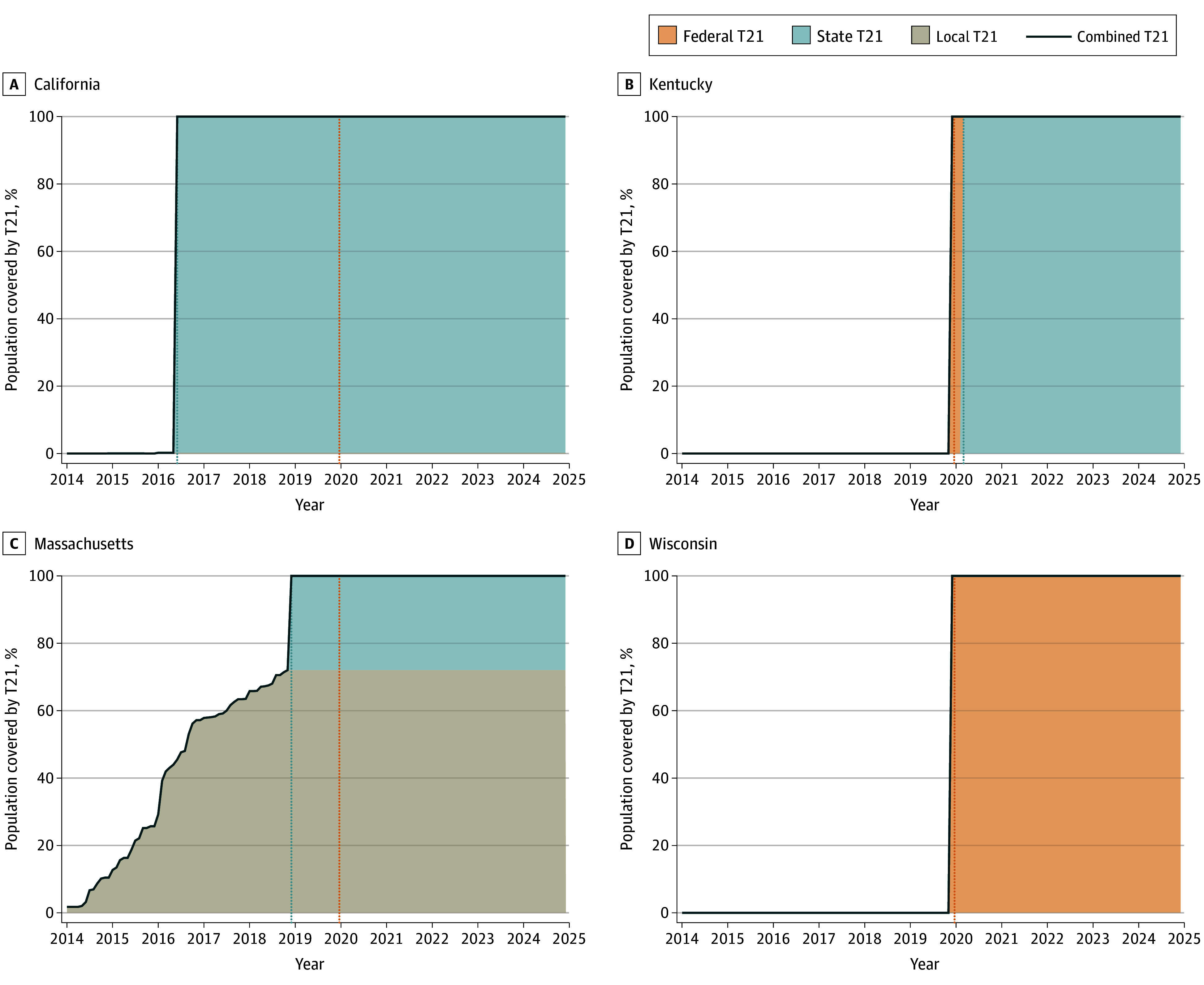
Percent of State Populations Covered by Tobacco 21 (T21) Policies, 2014 to 2024 Local T21 policies are based on the percent of the population covered by local-level laws implemented between 2014 and 2019 according to the Tobacco 21 Population Coverage Database.^13^ State T21 policy implementation dates are based on Tobacco21.org (blue dotted line). The federal T21 law (orange dotted line) was signed into law in December 2019. Combined local, state, and federal coverage is depicted by the solid black line. California (A) was an early enactor of state-level T21. Kentucky (B) passed state-level T21 less than a year after the federal law. Massachusetts (C) had substantial local level coverage before passing a state law in 2018. Wisconsin (D) has no local or state level T21 coverage and relies solely on federal enforcement.

US T21 policy coverage steadily increased from 2005 to 2024 (Figure 1). We present results for 4 exemplar states with distinct T21 policy landscapes across Census regions: California (West), Kentucky (South), Massachusetts (Northeast), and Wisconsin (Midwest). Figure 2 shows the T21 coverage timelines for each of these states. California’s state policy went into effect 3 and a half years before the federal law, whereas Kentucky’s went into effect less than a year after. In Massachusetts, more than 70% of residents were covered by a local T21 policy before that state’s law went into effect at the end of 2018. With no local or state T21 policy, Wisconsin is solely covered by federal law. State models were first validated by comparing adult smoking prevalence under the baseline scenario with observed 1992 to 2019 TUS-CPS survey data. Models generally reproduced observed historical smoking trends, but overestimated prevalence for some states in more recent survey years (eFigures 2-52 in Supplement 1). Under the baseline scenario, smoking prevalence is projected to decrease by 47.2% in California (12.5% in 2014 to 6.6% in 2100), 33.9% in Kentucky (27.1% to 17.9%), 41.9% in Massachusetts (14.8% to 8.6%), and 44.1% in Wisconsin (20.4% to 11.4%). From 2025 to 2100, models project 389 000 cumulative smoking-attributable deaths in California, 255 000 in Kentucky, 112 000 in Massachusetts, and 147 000 in Wisconsin (eTable 5 in Supplement 1).

**Figure 3.**
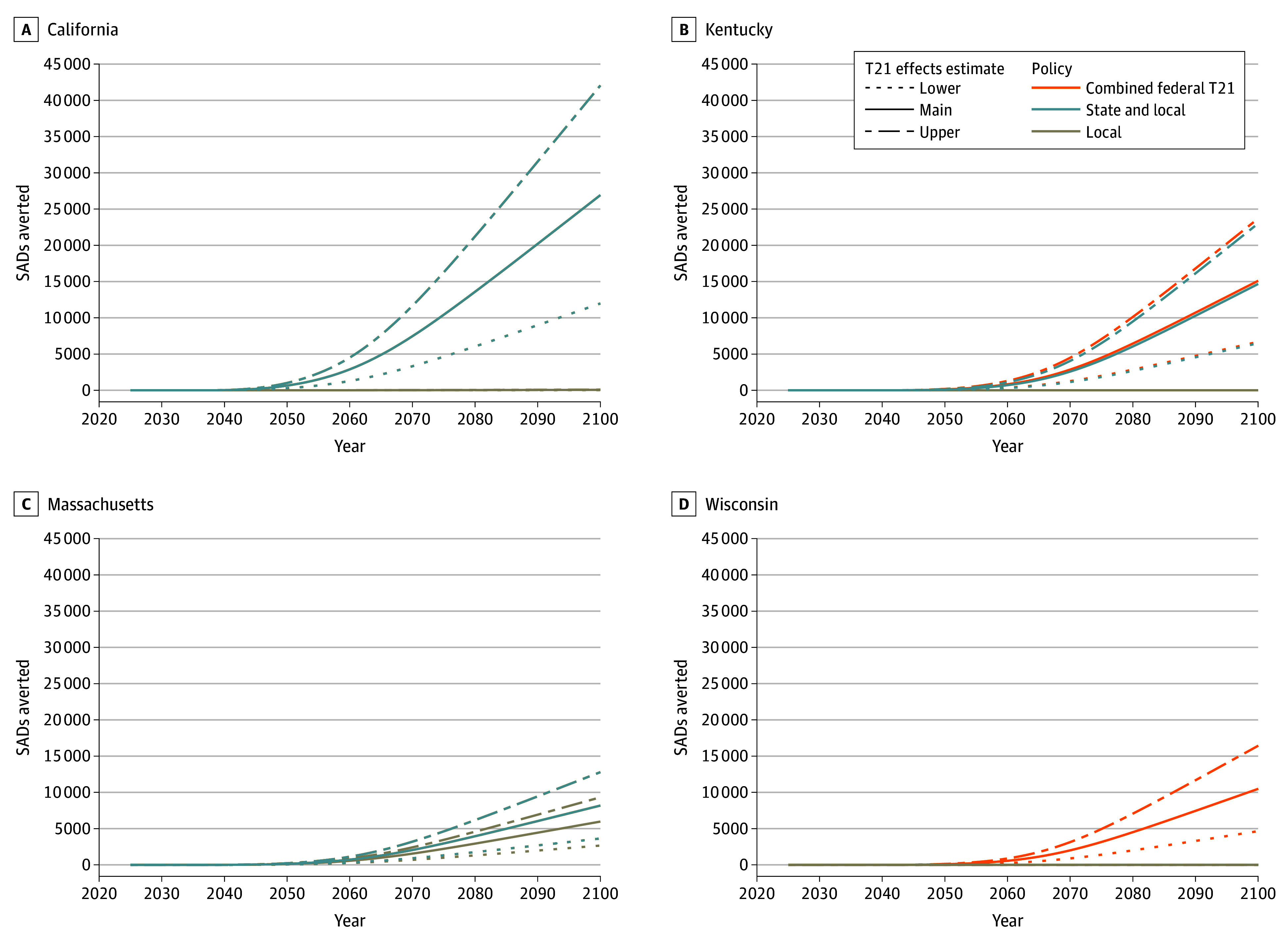
Cumulative Smoking-Attributable Deaths (SADs) Averted Under Tobacco 21 (T21) Policy Scenarios by State, 2025 to 2100 Cumulative SADs averted due to T21 policy efforts. Local T21, State and local T21, and combined federal T21 (local, state, and federal) policy scenarios are displayed as solid lines. Upper (dot-dash) and lower-bound (dotted) estimates reflect optimistic and pessimistic ranges around T21 policy effects. Results are shown from 2025 to 2100 because mortality reductions do not become apparent until decades after T21 policy changes to smoking initiation. Estimates reflect differences in population size by state. Census Bureau 2023 population estimates are as follows^17^: California: 39 million; Kentucky: 4.5 million; Massachusetts: 7.0 million; Wisconsin: 5.9 million.

Figure 3 shows cumulative SADs averted by state under each policy scenario through 2100. In California, by 2100, state and local T21 policies were associated with 27 000 premature deaths averted (range, 12 000-42 000)—identical to the combined federal T21 scenario because California’s state policy preceded the federal law. Under the local T21 scenario, California would see 60 fewer premature deaths (range, 27-93) due to limited local policy coverage before 2016. Kentucky had no local coverage, but the model projects 15 000 (range, 6000-23 000) fewer SADs associated with its state policy. The combined federal T21 scenario projects similar estimates (15 000; range, 7000-24 000) since federal T21 preceded the state law by less than a year. In Massachusetts, local T21 policies were associated with 6000 premature deaths averted (range, 3000-9000) by 2100, whereas the state and local T21 scenario indicates 8000 premature deaths averted (range, 4000-13 000), and the combined federal T21 scenario yields no further benefit. With no local or state T21 policies, associated premature deaths averted in Wisconsin were only under the combined federal T21 scenario (10 000; range, 5000-16 000).

**Figure 4.**
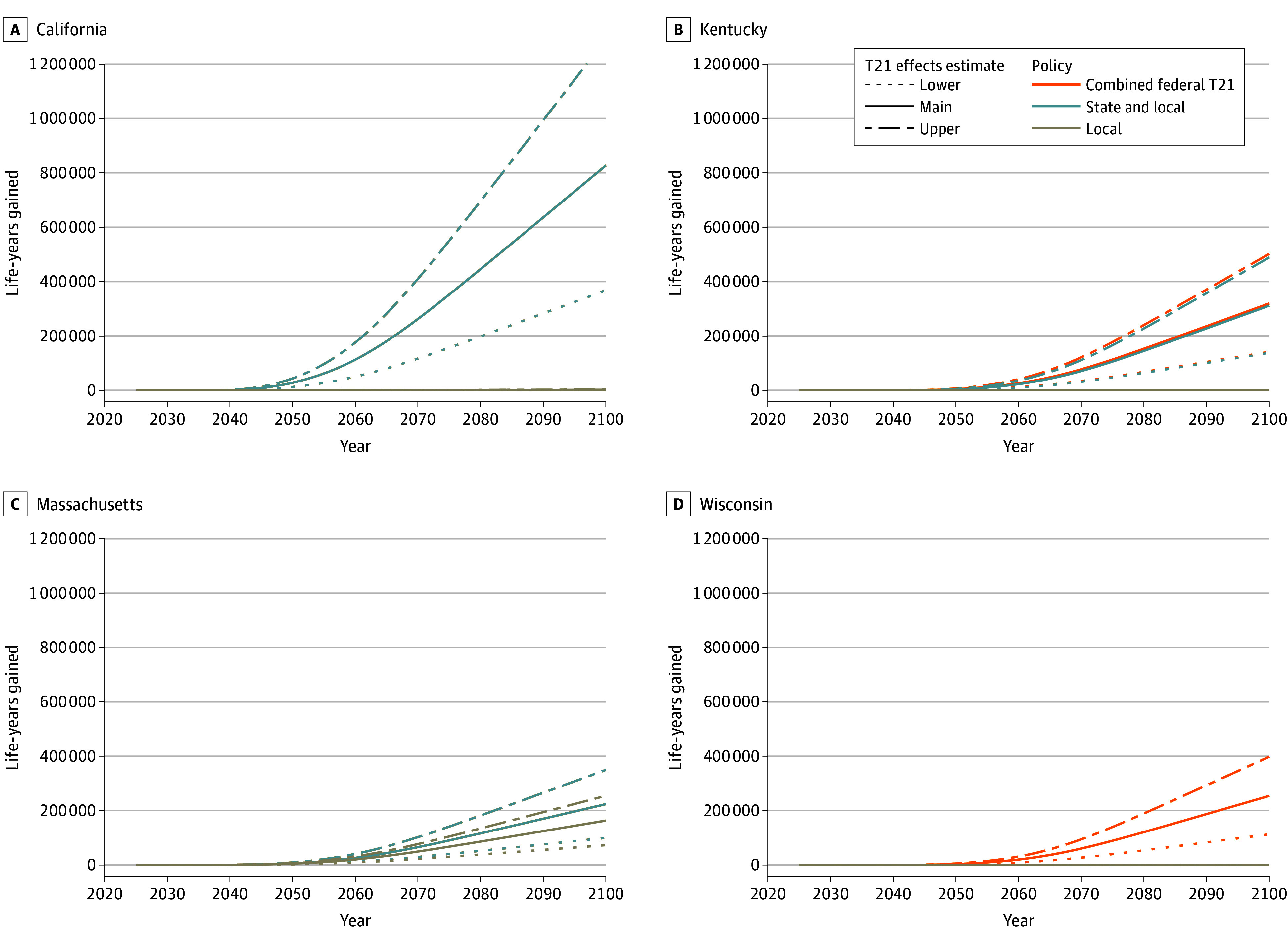
Cumulative Life-Years Gained Under Tobacco 21 (T21) Policy Scenarios by State, 2025 to 2100 Cumulative life-years gained due to T21 policy efforts. Local T21, state and local, and combined federal T21 (local, state, and federal) policy scenarios are displayed as solid lines. Upper (dot-dash) and lower-bound (dotted) estimates reflect optimistic and pessimistic ranges around T21 policy effects. Results are shown from 2025 to 2100 because mortality reductions do not become apparent until decades after T21 policy changes to smoking initiation. Estimates reflect differences in population size by state. Census Bureau 2023 population estimates are as follows^17^: California: 39 million; Kentucky: 4.5 million; Massachusetts: 7.0 million; Wisconsin: 5.9 million.

Figure 4 shows the cumulative LYG across states under each policy scenario. Local T21 policies in California were associated with 1800 (range, 800-2900) LYG by 2100. Both the state and local T21 scenario and the combined federal T21 scenario were associated with 827 000 (range, 368 000-1.3M) LYG. Kentucky’s state policy was associated with 312 000 (range, 138 000-489 000) LYG by 2100; with slightly more LYG under the combined federal T21 scenario (320 000; range, 142 000-502 000). Massachusetts’ local T21 policies were associated with 163 000 LYG (range, 73 000-254 000) on their own. Combined with state T21 policy, there would be 224 000 LYG (range, 99 000-350 000). With no local or state T21 policies, Wisconsin only benefited in the combined federal T21 scenario, which estimated 254 000 LYG (range, 113 000-398 000).

**Figure 5.**
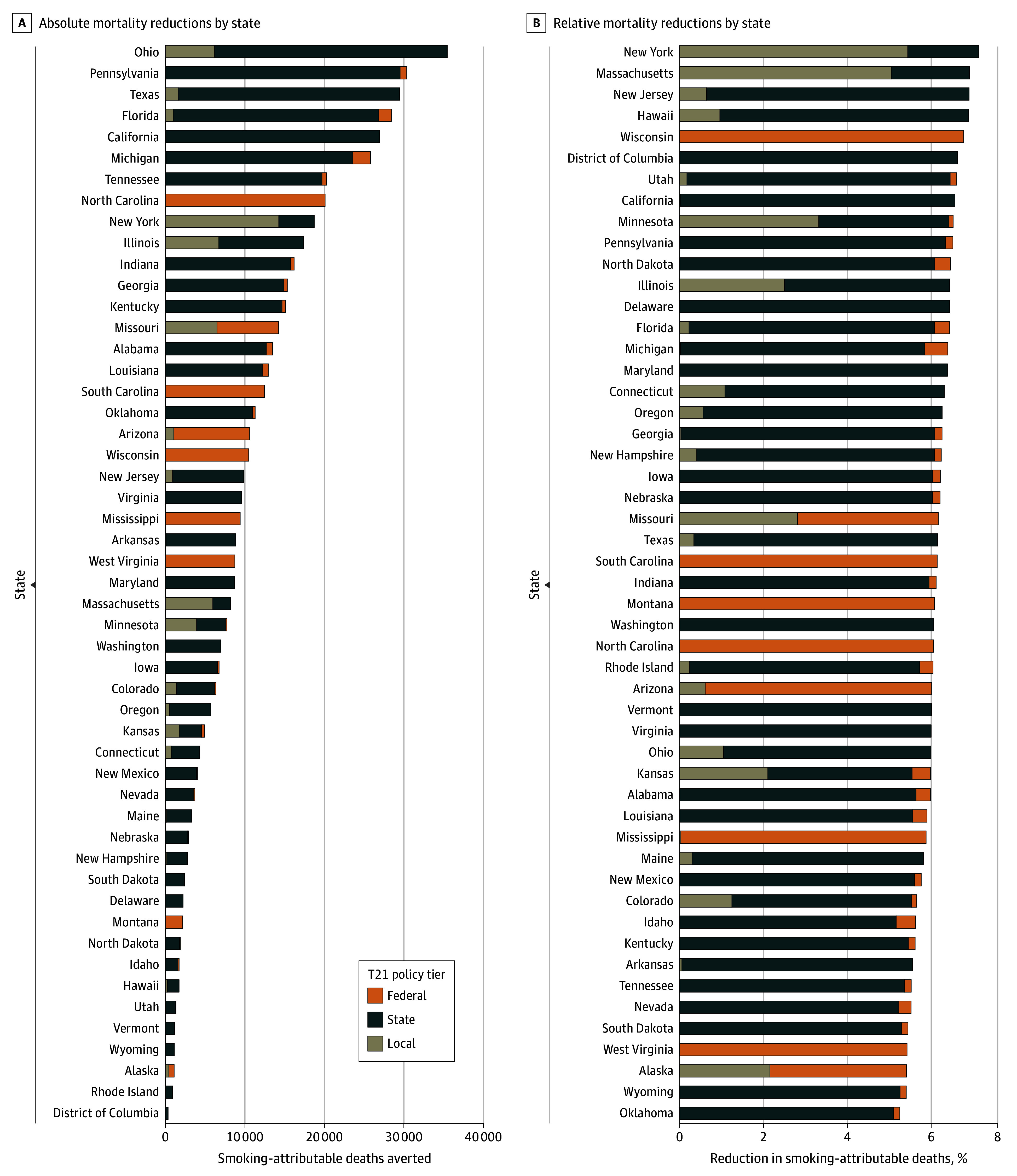
Tobacco 21 (T21) State Rankings and Absolute vs Relative Reductions in Premature Mortality Among All Persons Born After 1985, 2025 to 2100 A, State-by-state rankings of the effects of T21 policy efforts on absolute premature mortality reductions and (B) relative reductions in premature mortality. The bar plots illustrate reductions in premature mortality attributed to 3 policy scenarios: local T21, state and local T21, and combined federal T21. Each color represents a different policy tier (federal, state, or local) and indicates the source of the health gains achieved. Relative reductions in premature mortality (B) are calculated as a percent reduction in smoking-attributable deaths from the baseline scenario, incorporating state-specific life expectancies.

Figure 5 shows state-by-state comparisons of SADs averted among all those born after 1985, disaggregated according to local, state, or federal contributions to T21 policies. States with large populations (eg, Ohio, California, Texas) achieve the largest number of premature deaths averted compared with the least populous states (Figure 5A). Four states without any local- or state-level T21 policies would only realize such mortality reductions under the federal T21 law: North Carolina, South Carolina, Montana, and Wisconsin. North Carolina has the most to gain in absolute terms from federal law implementation due to its population size and lack of local and state T21 policies. Local T21 policies are major contributors to premature mortality reductions in states with extensive local policy coverage (Massachusetts, New York, Illinois, Missouri, and Minnesota).

Because state population sizes, smoking patterns, and underlying mortality rates differ substantially, we also present mortality reductions based on the percent of all projected SADs averted relative to the baseline scenario (Figure 5B). Wisconsin had the most to gain in relative terms from federal law implementation. Relative reductions were largest among early adopter states (eg, New York, Massachusetts, and Hawaii), reducing SADs by approximately 7%.

Local T21 policies were associated with 55 000 (range, 25 000-86 000) premature deaths averted through 2100. State and local T21 policies were associated with 442 000 (range, 196 000-693 000), and the combined federal T21 policy with 526 000 (range, 233 000-824 000). All local T21 policies were associated with 1.5 million (range, 660 000-2.3 million) LYG by 2100. Combined with state efforts, this would increase to 11.3 million (range, 5.0 million to 17.7 million) LYG, and then with federal T21 policy to 13.3 million (range, 5.9 million to 20.9 million) LYG by 2100.

If the federal law had an immediate impact in states without T21 policies, this would be associated with 84 000 (range, 37 000-131 000) SADs averted or 15.9% of all reductions. State policies would be the largest contributor to premature mortality reductions, with 387 000 SADs averted (range, 171 000-607 000) or 73.5% of all reductions; local policies would account for 10.5%.

See state-by-state results in eFigures 2 to 52 in Supplement 1 and at https://tobaccopolicyeffects.org/#t21. See eTable 6 and eFigures 53 to 103 in Supplement 1 for additional sensitivity analyses.

## Discussion

To our knowledge, this is the first study to quantify the potential long-term health benefits associated with T21 policies across 50 US states and Washington, DC. The work is strengthened by reliance on quasi-experimental estimates, comprehensive policy data, and models that account for each state’s historical smoking and mortality patterns.

We estimate that the US T21 movement could avert up to 526 000 premature smoking-attributable deaths, with 13.3 million life-years gained this century. This is more than double the number reported in the 2015 NAM report (249 000). The NAM committee estimated that a national T21 policy would reduce smoking initiation by 15% among adults aged 18 to 20 years; our model relied on empirical estimates from Hansen et al,^9^ which indicate 34% reductions to smoking initiation in the 18- to 20-year age group. Furthermore, the NAM report used smoking, mortality, and policy inputs for the US overall; our models reveal how state heterogeneity in health metrics and policy contexts produce disparate outcomes.

Each state’s baseline smoking and mortality rates, and policy timeline shape disparate outcomes. Consider California: despite being the nation’s most populous state and the second to implement a state T21 policy, its estimated absolute number of premature deaths averted was eclipsed in relative terms by less populous states. This is partly because of California’s lower initial smoking prevalence, which leaves less room for improvement than states with higher smoking rates. California’s longer life expectancies and lower mortality rates also diminish the impact of reduced smoking. Ohio’s population is less than one-third that of California, but its higher mortality^22^ and smoking rates^23^ make Ohio the leader in absolute premature mortality reductions. Massachusetts and New York are top-ranked because of early and extensive local adoption of T21 policies.^2^ Similarly, New Jersey and Hawaii rank highly in relative reductions as early T21 adopters.

Long-term projections are ripe with uncertainty. There are inherent challenges to predicting the future that cannot be fully addressed, even with sophisticated methodology. We focused our analysis on policy scenarios and effects—the 2 most important sources of uncertainty. Our most optimistic scenario assumed that the federal T21 policy was immediately enforced. Thus far, the federal law’s most significant impact has likely been accelerating adoption of state laws. A more conservative scenario evaluated state and local T21 policies only (Preventing Tobacco Addiction Foundation/Tobacco 21 experts’ understanding that T21 compliance is higher under local and state T21 laws) in the absence of federal law. The effects of US T21 policies likely fall between these 2 conditions. We evaluated a wide range of policy effects in our main analyses; Supplement 1 considers an additional scenario where T21 effects decline over time, though there is no empirical evidence on changing T21 policy effectiveness over time.

### Limitations

This study has some limitations. First, the disruptive effects of the COVID-19 pandemic were not explicitly incorporated. Second, in the absence of nationally representative, quasi-experimental evidence on local or federal policy effects, state policy effects were assumed to be the same for local and federal T21 policies. Third, for some states, models slightly overestimated smoking prevalence. We applied a systematic approach to each state, which facilitates direct comparability between model results but leads to lower resolution and prevalence overestimation in some states. We did not explicitly consider e-cigarettes; the analysis only reflects e-cigarettes to the extent that they may have influenced recent declines in smoking. However, overestimation of smoking under the baseline scenario is unlikely to influence the magnitude of policy impacts when comparing outcomes between 2 modeled scenarios.

Although we did not address other sociodemographic disparities, our smoking parameters indirectly reflect each state’s sociodemographic composition. For example, several states without their own T21 policies have larger shares of the population that are American Indian and Alaska Native (Alaska, Montana),^24^ living in rural areas (Mississippi, Montana),^25^ and in poverty (Missouri)^26^—factors associated with higher smoking. Future analyses should assess the effects of T21 policies on populations disproportionately harmed by tobacco use, including people with behavioral health conditions.^27^

Finally, we did not account for heterogeneity in T21 policies or their enforcement; however, our policy effects parameter was based on estimates that capture the population-weighted average effect of state policies as they were implemented. Assessing enforcement is challenging because the responsibility and level of enforcement varies widely across states. For the 44 states with state-level T21 laws, Tobacco21.org offers a thorough evaluation and grading system. Their assessment examines each state’s enforcement measures, licensing regulations, and penalties for violations, but does not cover the 8 states that rely solely on enforcement of federal law.

## Conclusion

Fully enforcing T21 policies could reduce geographic disparities, as states that have the most to gain from these policies are those with higher smoking prevalence, higher mortality risks, lower life expectancy, and, often, poor tobacco control environments. Enforcement of Federal T21 is split between the Food and Drug Administration and each state, but inspections reach a small fraction of retailers and depend on state and local capacity. Many states lack mandatory compliance checks, relying on complaint-based inspections instead. Although the federal law was signed on December 20, 2019, states have been given 5 years (until December 20, 2024), to conform to federal guidance before penalties are enforced.^28^ Penalties for lax enforcement are minimal, often limited to warning letters and nominal fines ($345 for 2 violations in a 12-month period), and do not incentivize retailer compliance.^29^ States that fall short of compliance benchmarks supposedly risk losing federal funds under the Synar Amendment, but exemptions are provided in exchange for states’ commitment to corrective action plans. Some hold-out states are resistant to enforcing T21, with some decision-makers under the mistaken belief that states do not have to comply with federal law. For the 8 remaining hold-out states, weaknesses in state and federal enforcement prevent their population from realizing substantial health gains. These analyses quantify the potential benefits achieved through T21 policies and demonstrate the urgent need for federal and state lawmakers to enhance their enforcement.

## Notes

Supplement 1. List of investigators State models eTable 1. Model parameters, assumptions, and data sources Tobacco 21 policy effects eTable 2. BRFSS estimates of T21 Impact on smoking participation (smoked ≥100 cigs & now smoke) eTable 3. Comparisons based on estimates from other quasi-experimental T21 analyses Tobacco 21 modeling results eFigure 1. Male smoking initiation probabilities by birth cohort under the combined federal T21 policy scenario in Wisconsin eTable 4. Comparison of National Academy of Medicine (NAM) report and Tobacco Control Policy (TCP) T21 modeling analyses eTable 5. State model outcomes by policy scenario: California, Kentucky, Massachusetts, Wisconsin State-by-state profile of T21 results eFigure 2. Alabama T21 model outcomes eFigure 3. Alaska T21 model outcomes eFigure 4. Arizona T21 model outcomes eFigure 5. Arkansas T21 model outcomes eFigure 6. California T21 model outcomes eFigure 7. Colorado T21 model outcomes eFigure 8. Connecticut T21 model outcomes eFigure 9. Delaware T21 model outcomes eFigure 10. District of Columbia T21 model outcomes eFigure 11. Florida T21 model outcomes eFigure 12. Georgia T21 model outcomes eFigure 13. Hawaii T21 model outcomes eFigure 14. Idaho T21 model outcomes eFigure 15. Illinois T21 model outcomes eFigure 16. Indiana T21 model outcomes eFigure 17. Iowa T21 model outcomes eFigure 18. Kansas T21 model outcomes eFigure 19. Kentucky T21 model outcomes eFigure 20. Louisiana T21 model outcomes eFigure 21. Maine T21 model outcomes eFigure 22. Maryland T21 model outcomes eFigure 23. Massachusetts T21 model outcomes eFigure 24. Michigan T21 model outcomes eFigure 25. Minnesota T21 model outcomes eFigure 26. Mississippi T21 model outcomes eFigure 27. Missouri T21 model outcomes eFigure 28. Montana T21 model outcomes eFigure 29. Nebraska T21 model outcomes eFigure 30. Nevada T21 model outcomes eFigure 31. New Hampshire T21 model outcomes eFigure 32. New Jersey T21 model outcomes eFigure 33. New Mexico T21 model outcomes eFigure 34. New York T21 model outcomes eFigure 35. North Carolina T21 model outcomes eFigure 36. North Dakota T21 model outcomes eFigure 37. Ohio T21 model outcomes eFigure 38. Oklahoma T21 model outcomes eFigure 39. Oregon T21 model outcomes eFigure 40. Pennsylvania T21 model outcomes eFigure 41. Rhode Island T21 model outcomes eFigure 42. South Carolina T21 model outcomes eFigure 43. South Dakota T21 model outcomes eFigure 44. Tennessee T21 model outcomes eFigure 45. Texas T21 model outcomes eFigure 46. Utah T21 model outcomes eFigure 47. Vermont T21 model outcomes eFigure 48. Virginia T21 model outcomes eFigure 49. Washington T21 model outcomes eFigure 50. West Virginia T21 model outcomes eFigure 51. Wisconsin T21 model outcomes eFigure 52. Wyoming T21 model outcomes Additional sensitivity analyses eTable 6. Sensitivity analysis extending cohort smoking trends by an additional 10 birth cohorts State-by-state profile of T21 results assuming policy effects decline over time eFigure 53. Alabama T21 model outcomes with policy decay eFigure 54. Alaska T21 model outcomes with policy decay eFigure 55. Arizona T21 model outcomes with policy decay eFigure 56. Arkansas T21 model outcomes with policy decay eFigure 57. California T21 model outcomes with policy decay eFigure 58. Colorado T21 model outcomes with policy decay eFigure 59. Connecticut T21 model outcomes with policy decay eFigure 60. Delaware T21 model outcomes with policy decay eFigure 61. District of Columbia T21 model outcomes with policy decay eFigure 62. Florida T21 model outcomes with policy decay eFigure 63. Georgia T21 model outcomes with policy decay eFigure 64. Hawaii T21 model outcomes with policy decay eFigure 65. Idaho T21 model outcomes with policy decay eFigure 66. Illinois T21 model outcomes with policy decay eFigure 67. Indiana T21 model outcomes with policy decay eFigure 68. Iowa T21 model outcomes with policy decay eFigure 69. Kansas T21 model outcomes with policy decay eFigure 70. Kentucky T21 model outcomes with policy decay eFigure 71. Louisiana T21 model outcomes with policy decay eFigure 72. Maine T21 model outcomes with policy decay eFigure 73. Maryland T21 model outcomes with policy decay eFigure 74. Massachusetts T21 model outcomes with policy decay eFigure 75. Michigan T21 model outcomes with policy decay eFigure 76. Minnesota T21 model outcomes with policy decay eFigure 77. Mississippi T21 model outcomes with policy decay eFigure 78. Missouri T21 model outcomes with policy decay eFigure 79. Montana T21 model outcomes with policy decay eFigure 80. Nebraska T21 model outcomes with policy decay eFigure 81. Nevada T21 model outcomes with policy decay eFigure 82. New Hampshire T21 model outcomes with policy decay eFigure 83. New Jersey T21 model outcomes with policy decay eFigure 84. New Mexico T21 model outcomes with policy decay eFigure 85. New York T21 model outcomes with policy decay eFigure 86. North Carolina T21 model outcomes with policy decay eFigure 87. North Dakota T21 model outcomes with policy decay eFigure 88. Ohio T21 model outcomes with policy decay eFigure 89. Oklahoma T21 model outcomes with policy decay eFigure 90. Oregon T21 model outcomes with policy decay eFigure 91. Pennsylvania T21 model outcomes with policy decay eFigure 92. Rhode Island T21 model outcomes with policy decay eFigure 93. South Carolina T21 model outcomes with policy decay eFigure 94. South Dakota T21 model outcomes with policy decay eFigure 95. Tennessee T21 model outcomes with policy decay eFigure 96. Texas T21 model outcomes with policy decay eFigure 97. Utah T21 model outcomes with policy decay eFigure 98. Vermont T21 model outcomes with policy decay eFigure 99. Virginia T21 model outcomes with policy decay eFigure 100. Washington T21 model outcomes with policy decay eFigure 101. West Virginia T21 model outcomes with policy decay eFigure 102. Wisconsin T21 model outcomes with policy decay eFigure 103. Wyoming T21 model outcomes with policy decay References

Supplement 2. Data Sharing Statement
