# Supplementary material for: US Tobacco 21 Policies and Potential Mortality Reductions by State
Source: JAMA Health Forum. 2024 Dec 20;5(12):e244445. doi: 10.1001/jamahealthforum.2024.4445 (PMC11662258; doi:10.1001/jamahealthforum.2024.4445)
Supplement: Supplement 1. — List of investigators State models eTable 1. Model parameters, assumptions, and data sources Tobacco 21 policy effects eTable 2. BRFSS estimates of T21 Impact on smoking participation (smoked ≥100 cigs & now smoke) eTable 3. Comparisons based on estimates from other quasi-experimental T21 analyses Tobacco 21 modeling results eFigure 1. Male smoking initiation probabilities by birth cohort under the combined federal T21 policy scenario in Wisconsin eTable 4. Comparison of National Academy of Medicine (NAM) report and Tobacco Control Policy (TCP) T21 modeling analyses eTable 5. State model outcomes by policy scenario: California, Kentucky, Massachusetts, Wisconsin State-by-state profile of T21 results eFigure 2. Alabama T21 model outcomes eFigure 3. Alaska T21 model outcomes eFigure 4. Arizona T21 model outcomes eFigure 5. Arkansas T21 model outcomes eFigure 6. California T21 model outcomes eFigure 7. Colorado T21 model outcomes eFigure 8. Connecticut T21 model outcomes eFigure 9. Delaware T21 model outcomes eFigure 10. District of Columbia T21 model outcomes eFigure 11. Florida T21 model outcomes eFigure 12. Georgia T21 model outcomes eFigure 13. Hawaii T21 model outcomes eFigure 14. Idaho T21 model outcomes eFigure 15. Illinois T21 model outcomes eFigure 16. Indiana T21 model outcomes eFigure 17. Iowa T21 model outcomes eFigure 18. Kansas T21 model outcomes eFigure 19. Kentucky T21 model outcomes eFigure 20. Louisiana T21 model outcomes eFigure 21. Maine T21 model outcomes eFigure 22. Maryland T21 model outcomes eFigure 23. Massachusetts T21 model outcomes eFigure 24. Michigan T21 model outcomes eFigure 25. Minnesota T21 model outcomes eFigure 26. Mississippi T21 model outcomes eFigure 27. Missouri T21 model outcomes eFigure 28. Montana T21 model outcomes eFigure 29. Nebraska T21 model outcomes eFigure 30. Nevada T21 model outcomes eFigure 31. New Hampshire T21 model outcomes eFigure 32. New Jersey T21 model outcomes eFigure 33. New Mexico T21 model outcomes eFigure [file jamahealthforum-e244445-s001.pdf]

Supplemental Online Content

Tam J, Crippen A, Friedman A, et al. US Tobacco 21 Policies and Potential Mortality Reductions by State. *JAMA Health Forum.* Published online December 20, 2024. doi:10.1001/jamahealthforum.2024.4445

List of investigators

State models

eTable 1. Model parameters, assumptions, and data sources

Tobacco 21 policy effects

eTable 2. BRFSS estimates of T21 Impact on smoking participation (smoked ≥100 cigs & now smoke)

eTable 3. Comparisons based on estimates from other quasi-experimental T21 analyses

Tobacco 21 modeling results

eFigure 1. Male smoking initiation probabilities by birth cohort under the combined federal T21 policy scenario in Wisconsin

eTable 4. Comparison of National Academy of Medicine (NAM) report and Tobacco Control Policy (TCP) T21 modeling analyses

eTable 5. State model outcomes by policy scenario: California, Kentucky, Massachusetts, Wisconsin

State-by-state profile of T21 results

eFigure 2. Alabama T21 model outcomes

eFigure 3. Alaska T21 model outcomes

eFigure 4. Arizona T21 model outcomes

eFigure 5. Arkansas T21 model outcomes

eFigure 6. California T21 model outcomes

eFigure 7. Colorado T21 model outcomes

eFigure 8. Connecticut T21 model outcomes

eFigure 9. Delaware T21 model outcomes

© 2025 Tam J et al. *JAMA Health Forum.*

eFigure 10. District of Columbia T21 model outcomes
eFigure 11. Florida T21 model outcomes
eFigure 12. Georgia T21 model outcomes
eFigure 13. Hawaii T21 model outcomes
eFigure 14. Idaho T21 model outcomes
eFigure 15. Illinois T21 model outcomes
eFigure 16. Indiana T21 model outcomes
eFigure 17. Iowa T21 model outcomes
eFigure 18. Kansas T21 model outcomes
eFigure 19. Kentucky T21 model outcomes
eFigure 20. Louisiana T21 model outcomes
eFigure 21. Maine T21 model outcomes
eFigure 22. Maryland T21 model outcomes
eFigure 23. Massachusetts T21 model outcomes
eFigure 24. Michigan T21 model outcomes
eFigure 25. Minnesota T21 model outcomes
eFigure 26. Mississippi T21 model outcomes
eFigure 27. Missouri T21 model outcomes
eFigure 28. Montana T21 model outcomes
eFigure 29. Nebraska T21 model outcomes
eFigure 30. Nevada T21 model outcomes
eFigure 31. New Hampshire T21 model outcomes
eFigure 32. New Jersey T21 model outcomes
eFigure 33. New Mexico T21 model outcomes
eFigure 34. New York T21 model outcomes
eFigure 35. North Carolina T21 model outcomes
eFigure 36. North Dakota T21 model outcomes
eFigure 37. Ohio T21 model outcomes
eFigure 38. Oklahoma T21 model outcomes
eFigure 39. Oregon T21 model outcomes
eFigure 40. Pennsylvania T21 model outcomes

© 2025 Tam J et al. *JAMA Health Forum*.

eFigure 41. Rhode Island T21 model outcomes

eFigure 42. South Carolina T21 model outcomes

eFigure 43. South Dakota T21 model outcomes

eFigure 44. Tennessee T21 model outcomes

eFigure 45. Texas T21 model outcomes

eFigure 46. Utah T21 model outcomes

eFigure 47. Vermont T21 model outcomes

eFigure 48. Virginia T21 model outcomes

eFigure 49. Washington T21 model outcomes

eFigure 50. West Virginia T21 model outcomes

eFigure 51. Wisconsin T21 model outcomes

eFigure 52. Wyoming T21 model outcomes

Additional sensitivity analyses

eTable 6. Sensitivity analysis extending cohort smoking trends by an additional 10 birth cohorts

State-by-state profile of T21 results assuming policy effects decline over time

eFigure 53. Alabama T21 model outcomes with policy decay

eFigure 54. Alaska T21 model outcomes with policy decay

eFigure 55. Arizona T21 model outcomes with policy decay

eFigure 56. Arkansas T21 model outcomes with policy decay

eFigure 57. California T21 model outcomes with policy decay

eFigure 58. Colorado T21 model outcomes with policy decay

eFigure 59. Connecticut T21 model outcomes with policy decay

eFigure 60. Delaware T21 model outcomes with policy decay

eFigure 61. District of Columbia T21 model outcomes with policy decay

eFigure 62. Florida T21 model outcomes with policy decay

eFigure 63. Georgia T21 model outcomes with policy decay

eFigure 64. Hawaii T21 model outcomes with policy decay

eFigure 65. Idaho T21 model outcomes with policy decay

eFigure 66. Illinois T21 model outcomes with policy decay

eFigure 67. Indiana T21 model outcomes with policy decay

© 2025 Tam J et al. *JAMA Health Forum*.

eFigure 68. Iowa T21 model outcomes with policy decay
eFigure 69. Kansas T21 model outcomes with policy decay
eFigure 70. Kentucky T21 model outcomes with policy decay
eFigure 71. Louisiana T21 model outcomes with policy decay
eFigure 72. Maine T21 model outcomes with policy decay
eFigure 73. Maryland T21 model outcomes with policy decay
eFigure 74. Massachusetts T21 model outcomes with policy decay
eFigure 75. Michigan T21 model outcomes with policy decay
eFigure 76. Minnesota T21 model outcomes with policy decay
eFigure 77. Mississippi T21 model outcomes with policy decay
eFigure 78. Missouri T21 model outcomes with policy decay
eFigure 79. Montana T21 model outcomes with policy decay
eFigure 80. Nebraska T21 model outcomes with policy decay
eFigure 81. Nevada T21 model outcomes with policy decay
eFigure 82. New Hampshire T21 model outcomes with policy decay
eFigure 83. New Jersey T21 model outcomes with policy decay
eFigure 84. New Mexico T21 model outcomes with policy decay
eFigure 85. New York T21 model outcomes with policy decay
eFigure 86. North Carolina T21 model outcomes with policy decay
eFigure 87. North Dakota T21 model outcomes with policy decay
eFigure 88. Ohio T21 model outcomes with policy decay
eFigure 89. Oklahoma T21 model outcomes with policy decay
eFigure 90. Oregon T21 model outcomes with policy decay
eFigure 91. Pennsylvania T21 model outcomes with policy decay
eFigure 92. Rhode Island T21 model outcomes with policy decay
eFigure 93. South Carolina T21 model outcomes with policy decay
eFigure 94. South Dakota T21 model outcomes with policy decay
eFigure 95. Tennessee T21 model outcomes with policy decay
eFigure 96. Texas T21 model outcomes with policy decay
eFigure 97. Utah T21 model outcomes with policy decay
eFigure 98. Vermont T21 model outcomes with policy decay

© 2025 Tam J et al. *JAMA Health Forum.*

eFigure 99. Virginia T21 model outcomes with policy decay **eFigure 100.** Washington T21 model outcomes with policy decay **eFigure 101.** West Virginia T21 model outcomes with policy decay **eFigure 102.** Wisconsin T21 model outcomes with policy decay **eFigure 103.** Wyoming T21 model outcomes with policy decay References

eReferences

This supplemental material has been provided by the authors to give readers additional information about their work.

© 2025 Tam J et al. *JAMA Health Forum*.

List of investigators

Jamie Tam, MPH, PhD
Alyssa Crippen, PhD
Abigail Friedman, PhD
Jihyoun Jeon, PhD
David C. Colston, MPH
Nancy L. Fleischer, PhD
Catherine A. Vander Woude, MPH
Theodore R Holford, PhD
David T Levy, PhD
Rafael Meza, PhD

© 2025 Tam J et al. JAMA Health Forum.

State models

Smoking history parameters. Each state model's underlying initiation and cessation parameters were generated by the Cancer Intervention and Surveillance Modeling Network (CISNET) using an age-period-cohort statistical modeling framework that reconstructed smoking histories for each state.[1] The method used data from the 1992-2019 Tobacco Use Supplement to the Current Population Survey (TUS-CPS) to obtain parameters for each state, and the 1965-2018 National Health Interview Survey (NHIS) to obtain parameters for whole US when the span of time covered by TUS-CPS was inadequate. NHIS data were used to estimate the relationship between age and ever-smoking prevalence for the entire country, which can be affected by differential mortality by smoking status after age 30 and recall bias. These parameters for age were used as offsets in the model for ever-smoking prevalence for each state, thus fixing the contribution of mortality and recall bias across states. Cohort parameters in the age-period-cohort model were fixed for 1989 and subsequent years, thus hold constant the last birth cohort year for the available data at age 30; (individuals from the 1989 birth cohort were 30 years old in 2019, the last year for which TUS-CPS data were available).

Smoking initiation probabilities were estimated for each state using data derived from TUS-CPS, and these were calibrated so that the cumulative initiation probability agreed with the ever-smoking prevalence estimate at age 30. Constrained natural splines for the effects of age, period, and cohort were used in a logistic regression model that incorporated sample weights. Estimated age and cohort parameters were held constant for future years, and the net effect is for smoking initiation estimates to be held constant after the 2011 cohort for model projections through the year 2100.

Similarly, a constrained natural splines for the effects of age, period and cohort in a logistic regression model was used to estimate the smoking cessation probabilities with parameters held constant for future years. The net effect of this was that cessation probabilities were held constant after the 2003 birth cohort. Cessation parameters reflect permanent quitting with no relapse, thus an individual was defined as having quit if smoking had ceased for two years —consistent with prior models.[2-6] Because smoking prevalence estimates in both the model and in the TUS-CPS data include people who recently quit smoking, they are therefore higher than those produced using traditional survey definitions of current smoking (excludes all individuals who self-report quitting, regardless of how recently they quit).

Models reproduced most historical smoking trends in the TUS-CPS under the baseline scenario. However, some state models overestimated smoking prevalence in more recent years such as the TUS-CPS 2018-2019; this was more prominent among men than women. In recent years, smoking prevalence has declined at an accelerated rate among young adults;[7] some factors likely affecting these trends are e-cigarette use[8] and use of little cigars and cigarillos.[9,10]

The smoking history parameters were generated with data that have less information for recent birth cohorts. For example, the 1990 birth cohort has fewer data points available in the NHIS or TUS-CPS compared to the 1970 birth cohort, such that recent declines in smoking among young people are less represented. Baseline smoking prevalence projections should be interpreted as conservative estimates.

Mortality. State-specific mortality rates were estimated using 1969-2020 National Center for Health Statistics data on the number of annual deaths by gender, state, and single year of age.[11] Midyear population estimates were obtained from the SEER website.[12] An age-cohort model was fitted to these data using constrained natural splines for the age and cohort effects. For age, the final knot was set to 35, forcing the age trend to be linear for those older than 35. That constraint corresponds to the Gompertz model[13] and agreed well with the available data. This model provided estimates of mortality by age up to the 1990 birth cohort. Cohort probabilities were assumed to be constant from the 1990 birth cohort onwards. The Rosenberg method was used with our estimates of mortality and smoking history parameters to obtain estimates of mortality for current, former, and never smoking.[14]

Like previous models,[4,5] premature smoking-attributable deaths (SADs) associated with current smoking were calculated by taking the difference in mortality between never and current-smoking individuals for a given age, gender, and year, and applying this to the population of people who currently smoke within that age, gender, and year. The latter was determined by multiplying model estimates of prevalence by Census Bureau population estimates for the state's annual resident population from 2010-2021, holding state population estimates constant

© 2025 Tam J et al. JAMA Health Forum.

from 2021-2100.[15,16] (At the time of writing, state population estimates by single year of age for 2022-2023 were not available.[15]) A parallel calculation was conducted for SADs associated with former smoking and summed across the population.[4] For each death at a given age among those who were current or former smoking, the number of smoking-attributable life-years lost was calculated by taking the remaining life expectancy of that individual if they had never smoked; this is then summed across the population to produce the total number of life-years lost.[4,5]

eTable 1 outlines all of the model parameters, data sources, and assumptions.

© 2025 Tam J et al. JAMA Health Forum.

eTable 1. Model parameters, assumptions, and data sources

Parameters and inputs	Description	Assumptions	Source
Policy effect estimates	Quasi-experimental estimates of the effect of T21 policies on smoking among adults ages 18-20 were translated into percent reductions to smoking initiation probabilities (34%, with 95% CI: 15%-53%).	Policy effect reduces smoking initiation only among persons ages 18-20. Main model estimate assumes no decay in the effect over time. Supplement 1 considers an additional sensitivity analysis scenario with the policy effect decaying over time.	Hansen et al., 2022[2]
Smoking initiation probabilities	State-specific probabilities of smoking initiation by age, gender, and birth cohort generated by the Cancer Intervention and Surveillance Modeling Network (CISNET) Lung consortium.	Probabilities held constant from the 2011 birth cohort onwards. Sensitivity analysis carry cohorts' initiation trends into the future.	Holford et al., 2023[4]; CISNET Lung Working Group, 2023[17]
Smoking cessation probabilities	State-specific probabilities of smoking cessation by age, gender, and birth cohort generated by the CISNET Lung consortium.	Probabilities held constant from the 2003 birth cohort onwards. Sensitivity analysis carry cohorts' cessation trends into the future.	Holford et al., 2023[4]; CISNET Lung Working Group, 2023[17]
Mortality probabilities	State-specific mortality probabilities by age, gender, and smoking status generated by the CISNET Lung consortium. These were estimated using an age-cohort model with inputs from the National Center for Health Statistics[14] and SEER.[15] Estimates of overall mortality were partitioned by smoking status using the Rosenberg method.[14] For people who formerly smoked, mortality probabilities are applied as a function of years-since-quitting. Details regarding the methods used for mortality risk among those who formerly smoked are described in the supplement of Tam et al. 2021.[4]	Mortality probabilities are assumed constant from the 1989 birth cohort onwards.	CISNET Lung Working Group, 2024[18]
Population sizes	Annual state population estimates from 2010-2023 are used to calculate the number of smoking-attributable deaths at the state level.	Population projections assumed constant from 2023-2100.	United States Census Bureau.[18,19]

© 2025 Tam J et al. JAMA Health Forum.

Tobacco 21 policy effects

To identify parameter estimates for Tobacco-21 (T21) effects, we reviewed the T21 literature, limiting consideration to nationally representative studies that used quasi-experimental research designs—that is, statistical methods capable of generating causal estimates in the absence of randomization if key assumptions hold—to assess T21 policies' impacts on cigarette smoking, and carried out key assumption tests to confirm that causal interpretation was appropriate. Three papers met these requirements: Friedman and Wu (2020),[19] Hansen et al (2022),[20] and Abouk, De, and Pesko (2024).[21]

Of these three articles, only Hansen et al. estimated T21 effects on both minors and 18-20 year-olds. Those analyses used repeated cross-sectional data from the 2009-2019 waves of the biennial Youth Risk Behavior Surveillance System ("YRBSS," representative for US high school students) and the annual Behavioral Risk Factor Surveillance System ("BRFSS", representative for noninstitutionalized US civilians ages 18+). Note that these surveys' measures of smoking participation differ slightly: YRBSS asks about past 30 day use, whereas BRFSS conditions reporting that one "now smoke cigarettes" on having smoked ≥100 cigarettes in one's lifetime.

Multivariable logistic regressions estimated both two-way fixed effect (TWFE) and triple-difference (DDD) analyses—two quasi-experimental approaches—and reported results as marginal effects along with standard errors. Using YRBSS data, the authors do not find statistically significant T21 effects on smoking participation (i.e., with $p<0.05$) for 16-17 year-olds or 18 year-olds alone (see Table 6 in Hansen et al, 2023). While those analyses of 18 year-olds do show significant reductions in frequent smoking using TWFE, that estimate does not retain significance and is markedly smaller under stacked difference-in-differences ("stacked DD") approaches designed to address potential bias in the TWFE estimates.

Using BRFSS, Hansen et al (2022) find statistically significant T21 effects on smoking among 18-20 year-olds, in both TWFE analyses and stacked DD analyses (see Table 1 in Hansen et al, 2023). **eTable 2** below converts estimates from that paper's full covariate analyses of smoking participation into percent changes. In selecting modeling parameters, we focus on TWFE analyses as these offer a cleaner interpretation than DDD.[1] Noting that BRFSS is not designed to be representative for single years of age and the age-18 effect estimates seem implausibly large (**eTable 2**, see rows 3 and 5), we judged the estimates from analyses of 18-20 year-olds as a more reasonable basis for our parameters (**eTable 2**, rows 1 & 2). Reassuringly, row 1's 95% confidence interval brackets the percent changes implied by results from the two other aforementioned quasi-experimental analyses (**eTable 3**). Thus, we use corresponding estimates for our parameter capturing T21 policies' effects on 18-20 year-olds (-34%; 95% CI: -53%, -15%). We set our T21 policy effects parameter to 0 for youth younger than 18 years-old, as neither Hansen et al's (2023) estimates of effects on 16-17 year-olds' smoking participation nor Abouk, De, and Pesko's (2024) estimates for 8th and 10th graders were significant at the 5% level. Results by gender were not consistent across specifications, so effects on men and women ages 18-20 were assumed identical in the simulation models.

We recognize that staggered treatment adoption can introduce potential bias in TWFE analyses, particularly when treatment effects vary across periods or states. However, when Hansen et al. applied a "stacked DD" approach, which is robust to this specific bias, the resulting estimate (-0.037) was virtually identical to and statistically indistinguishable from the TWFE estimate (-0.039). Given the negligible difference between the two estimates and the lack of evidence for bias in the TWFE estimate, we proceeded with the standard TWFE approach for our baseline parameter estimates. We believe this approach effectively captures the policy's impact while ensuring comparability across different models.

[1]TWFE compares changes in the outcome variable among exposed relative to unexposed 18-20 year-olds, pre-vs-post exposure. DDD compares such changes among 18-20 year-olds to the same relative changes among an older group that should not be bound by the policy (e.g., 21-23 year-olds). This latter approach ensures that changes common to the treated and untreated age groups do not bias the policy effect estimate, but means that the effect estimate is inherently shaped by the comparator age-group. Indeed, Hansen et al's DDD estimates differ when using ages 21-23 vs 24-28 as comparators.

© 2025 Tam J et al. JAMA Health Forum.

eTable 2. BRFSS estimates of T21 Impact on smoking participation (smoked ≥100 cigs & now smoke)

Specification & Ages		Marginal Effects, full covariate specification: Marginal Effect (ß)	SE	95% CI LB = ß - (1.96*SE)	95% CI UB= ß + (1.96*SE)	Mean Pre-treatment Smoking Participation	Converting to Percent Changes: Based on ß	Based on 95% CI LB	Based on 95% CI UB
1.	TWFE, 18-20	-0.039**	0.011	-0.06056	-0.01744	0.115	-33.91%	-52.66%	-15.17%
2.	Stacked DD, 18-20	-0.037**	0.009	-0.05464	-0.01936	0.083	-44.58%	-65.83%	-23.33%
3.	TWFE, 18	-0.074**	0.017	-0.10732	-0.04068	0.094	-78.72%	-114.17%	-43.28%
4.	TWFE, 19-20	-0.027*	0.014	-0.05444	0.00044	0.127	-21.26%	-42.87%	0.35%‡
5.	DDD, 18 vs 21-23	-0.060**	0.014	-0.08744	-0.03256	0.094	-63.83%	-93.02%	-34.64%
6.	DDD, 19-20 vs 21-23	-0.015*	0.007	-0.02872	-0.00128	0.127	-11.81%	-22.61%	-1.01%

Notes: Rows 1-2 are based on estimates from Hansen et al (2022) Table 1, while 3-6 use estimates in Table 4.[20] Effect estimates for ages 16-17 are not included as these were not statistically significant at conventional levels. "CI"=Confidence Interval. "UB"=Upper Bound of 95% CI. "LB"=Lower Bound of 95% CI. * p<0.05, ** p<0.01

‡ The positive upper bound of this statistically significant estimate's confidence interval likely reflects rounding error. For example, if the -0.027 point estimate is rounded down (e.g., from -0.0274) and the standard error of 0.014 rounded up (e.g., from 0.0136), the 95% CI's upper bound would have come out as negative: -0.0274+(1.96*0.0136) = -0.0007. More precise measures from Hansen et al would be needed to confirm this.

eTable 3. Comparisons based on estimates from other quasi-experimental T21 analyses

Specification	Marginal Effect (ß)	SE	95% CI LB = ß - (1.96*SE)	95% CI UB= ß + (1.96*SE)	Based on ß	Based on 95% CI LB	Based on 95% CI UB
Friedman & Wu (2020),[19] Ages 18-20	-0.0306	0.012	-0.05412	-0.00708	-25.71%	-45.48%	-5.95%
Abouk, De, & Pesko,[21] OLS of 12th graders	-0.0230**	0.0086	-0.039856	-0.006144	-18.74%	-32.48%	-5.01%
Abouk, De, & Pesko, 2SDID of 12th graders	-0.0208*	0.0100	-0.0404	-0.0012	-16.95%	-32.93%	-0.98%

Notes: Each row's entries are based on the noted article's estimates of T21 policies' impacts on smoking participation in the corresponding grade/age group, with mean smoking participation at 11.9% in Friedman & Wu (2019) and 12.27% in Abouk, De, & Pesko (2024). "CI"=Confidence Interval. "UB"=Upper Bound of 95% CI. "LB"=Lower Bound of 95% CI. * p<0.05, ** p<0.01

© 2025 Tam J et al. JAMA Health Forum.

Tobacco 21 modeling results

eFigure 1 illustrates the impact of policy effects under a combined federal T21 scenario on smoking initiation probabilities for men in Wisconsin.

eTable 4 compares our model analyses with the 2015 National Academy of Medicine report.[22]

eTable 5 presents smoking prevalence and mortality results across the four exemplar states.

eFigure 1. Male smoking initiation probabilities by birth cohort under the combined federal T21 policy scenario in Wisconsin

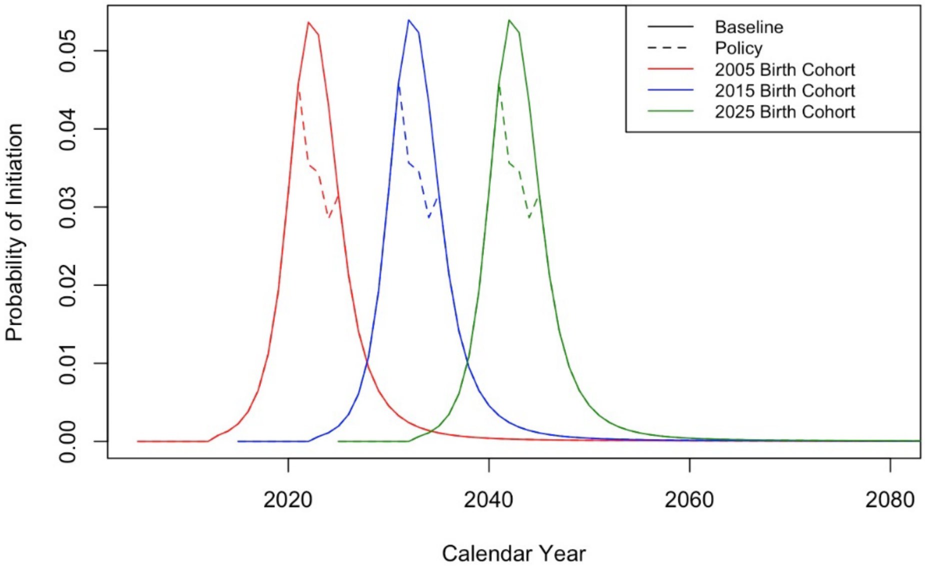

Notes: Changes to smoking initiation probabilities under the policy scenario are applied to ages 18-20 for all affected birth cohorts.

© 2025 Tam J et al. JAMA Health Forum.

eTable 4. Comparison of National Academy of Medicine (NAM) report and Tobacco Control Policy (TCP) T21 modeling analyses

<table>
<tr><th></th><th>NAM report modeling analysis (2015)[22]</th><th>TCP model T21 analysis (2024)</th></tr>
<tr><td>Objective</td><td>Simulate the long-term health effects associated with raising the minimum age of legal tobacco purchase (MLA) to 19, 21, or 25 from 2015-2100
Evaluate outcomes associated with a hypothetical federal law scenario</td><td>Simulate the long-term health effects associated with T21 policies based on real-world local, state, and federal T21 policies from 2005-2100
Evaluate outcomes associated with 3 scenarios: local T21, state and local, and combined T21 policies (i.e., local, state, and federal)</td></tr>
<tr><td>Population(s)</td><td>United States population</td><td>51 populations representing each of the 50 US states and District of Columbia</td></tr>
<tr><td>Policy effect sizes by age</td><td>Committee of experts categorized policy effects on smoking initiation for ages 0-25 as ‘small, medium, or large’. These were then assigned ranges of fixed 5% increments from 5-30%.
Assumes no effects on smoking cessation
<table>
<tr><th>Reduction in initiation by age</th><th>MLA 19</th><th>MLA 21</th><th>MLA 25</th></tr>
<tr><td>0-14</td><td>5%</td><td>15%</td><td>15%</td></tr>
<tr><td>15-17</td><td>10%</td><td>25%</td><td>30%</td></tr>
<tr><td>18</td><td>10%</td><td>15%</td><td>20%</td></tr>
<tr><td>19-20</td><td>0%</td><td>15%</td><td>20%</td></tr>
<tr><td>21-25</td><td>0%</td><td>0%</td><td>5%</td></tr>
</table></td><td>Estimated from quasi-experimental studies of T21 policy effects (Hansen et al.) for ages 18-20, with upper and lower bounds based on effect estimates’ 95% confidence intervals.
Assumes no effects on smoking initiation at ages <18 and >20 based on the absence of statistically significant policy effect estimates for those ages in Hansen et al.
Assumes no effects on smoking cessation
<table>
<tr><th>Reduction in initiation by age</th><th>Main estimate (95% CI)</th></tr>
<tr><td>18-20</td><td>34% (15% - 53%)</td></tr>
</table></td></tr>
<tr><td>Parameters</td><td>Does not incorporate real-world MLA policy coverage
National estimates of smoking initiation, cessation, and mortality probabilities that vary by age, birth cohort, and gender[23]</td><td>Incorporates real-world data on T21 policy coverage[24]
State-specific estimates of smoking initiation, cessation, and mortality probabilities that vary by age, birth cohort, and gender[1]</td></tr>
<tr><td>Methodology</td><td>Compares two US simulation models
Does not include smoking intensity
Mortality among those who formerly smoked does not vary based on years since quitting</td><td>Simulation models for each state
Does not include smoking intensity
Mortality among those who formerly smoked varies based on years since quitting</td></tr>
<tr><td>Mortality outcomes associated with policy</td><td>249,000 premature smoking-attributable deaths averted, 4.2 million life years gained from 2015-2100
Evaluates all-cause mortality and lung cancer-specific mortality</td><td>526,000 premature smoking-attributable deaths averted, 13.3 million life-years gained from 2005-2100 under combined T21 scenario
Evaluates all-cause mortality</td></tr>
</table>

© 2025 Tam J et al. JAMA Health Forum.

eTable 5. State model outcomes by policy scenario: California, Kentucky, Massachusetts, Wisconsin

<table>
<tr><th rowspan="2">State</th><th rowspan="2">Gender</th><th rowspan="2">Scenario</th><th colspan="4">Smoking prevalence (%)</th><th>Smoking-attributable deaths (thousands)</th><th>Life-years Lost (millions)</th></tr>
<tr><th>2025</th><th>2050</th><th>2075</th><th>2100</th><th>2025-2100</th><th>2025-2100</th></tr>
<tr><td rowspan="10">California
(local and state T21 coverage prior to federal T21)</td><td rowspan="5">Men</td><td>Baseline</td><td>11.1</td><td>8.4</td><td>8.2</td><td>8.2</td><td>322</td><td>9.8</td></tr>
<tr><td>Local T21</td><td>11.1
(11.1-11.1)</td><td>8.4
(8.4-8.4)</td><td>8.2
(8.2-8.2)</td><td>8.2
(8.2-8.2)</td><td>322
(322-322)</td><td>9.8
(9.8-9.8)</td></tr>
<tr><td>State & Local T21</td><td rowspan="2">10.8
(11-10.6)</td><td rowspan="2">7.6
(8.0-7.1)</td><td rowspan="2">7.3
(7.8-6.8)</td><td rowspan="2">7.3
(7.8-6.8)</td><td rowspan="2">299
(312-286)</td><td rowspan="2">9.0
(9.4-8.6)</td></tr>
<tr><td>Combined Federal T21</td></tr>
<tr><td rowspan="1" style="display:none"></td></tr>
<tr><td rowspan="5">Women</td><td>Baseline</td><td>6.8</td><td>5.0</td><td>5.0</td><td>5.0</td><td>67</td><td>1.8</td></tr>
<tr><td>Local T21</td><td>6.8
(6.8-6.8)</td><td>5.0
(5.0-5.0)</td><td>5.0
(5.0-5.0)</td><td>5.0
(5.0-5.0)</td><td>67
(67-67)</td><td>1.8
(1.8- 1.8)</td></tr>
<tr><td>State & Local T21</td><td rowspan="2">6.7
(6.8-6.6)</td><td rowspan="2">4.6
(4.8-4.3)</td><td rowspan="2">4.5
(4.8-4.2)</td><td rowspan="2">4.5
(4.8-4.2)</td><td rowspan="2">63
(65-61)</td><td rowspan="2">1.7
(1.7-1.6)</td></tr>
<tr><td>Combined Federal T21</td></tr>
<tr><td rowspan="1" style="display:none"></td></tr>
<tr><td rowspan="8">Kentucky
(no local T21 coverage)</td><td rowspan="4">Men</td><td>Baseline</td><td rowspan="2">23.9</td><td rowspan="2">19.4</td><td rowspan="2">19.0</td><td rowspan="2">19.0</td><td rowspan="2">168</td><td rowspan="2">3.6</td></tr>
<tr><td>Local T21</td></tr>
<tr><td>State & Local T21</td><td>23.6
(23.7-23.4)</td><td>17.9
(18.7-17.1)</td><td>17.2
(18.2-16.1)</td><td>17.1
(18.2-16.1)</td><td>158
(163-152)</td><td>3.4
(3.5-3.3)</td></tr>
<tr><td>Combined Federal T21</td><td>23.5
(23.7-23.3)</td><td>17.9
(18.7-17.1)</td><td>17.2
(18.2-16.1)</td><td>17.1
(18.2-16.1)</td><td>157
(163-151)</td><td>3.4
(3.5-3.3)</td></tr>
<tr><td rowspan="4">Women</td><td>Baseline</td><td rowspan="2">20.7</td><td rowspan="2">17.0</td><td rowspan="2">16.9</td><td rowspan="2">16.9</td><td rowspan="2">87</td><td rowspan="2">1.5</td></tr>
<tr><td>Local T21</td></tr>
<tr><td>State & Local T21</td><td>20.5
(20.6-20.3)</td><td>15.8
(16.5-15.2)</td><td>15.4
(16.2-14.5)</td><td>15.3
(16.2-14.5)</td><td>83
(85-80)</td><td>1.5
(1.5-1.4)</td></tr>
<tr><td>Combined Federal T21</td><td>20.4
(20.6-20.3)</td><td>15.8
(16.4-15.1)</td><td>15.4
(16.2-14.5)</td><td>15.3
(16.2-14.5)</td><td>83
(85-80)</td><td>1.5
(1.5-1.4)</td></tr>
</table>

© 2025 Tam J et al. JAMA Health Forum.

<table>
<tr><th rowspan="2">State</th><th rowspan="2">Gender</th><th rowspan="2">Scenario</th><th colspan="4">Smoking prevalence (%)</th><th>Smoking-attributable deaths (thousands)</th><th>Life-years Lost (millions)</th></tr>
<tr><th>2025</th><th>2050</th><th>2075</th><th>2100</th><th>2025-2100</th><th>2025-2100</th></tr>
<tr><td rowspan="8">Massachusetts
(local and state T21 prior to federal T21)</td><td rowspan="4">Men</td><td>Baseline</td><td>12.5</td><td>9.4</td><td>9.2</td><td>9.2</td><td>85</td><td>2.3</td></tr>
<tr><td>Local T21</td><td>12.2
(12.4-12.0)</td><td>8.7
(9.1-8.4)</td><td>8.4
(8.8-8.0)</td><td>8.4
(8.8-8.0)</td><td>80
(83-78)</td><td>2.2
(2.2-2.1)</td></tr>
<tr><td>State & Local T21</td><td rowspan="2">12.1
(12.3-11.9)</td><td rowspan="2">8.5
(9.0-8.0)</td><td rowspan="2">8.1
(8.7-7.6)</td><td rowspan="2">8.1
(8.7-7.6)</td><td rowspan="2">79
(82-75)</td><td rowspan="2">2.1
(2.2-2.0)</td></tr>
<tr><td>Combined Federal T21</td></tr>
<tr><td rowspan="4">Women</td><td>Baseline</td><td>10.0</td><td>8.1</td><td>8.1</td><td>8.0</td><td>27</td><td>0.67</td></tr>
<tr><td>Local T21</td><td>9.8
(10.0-9.7)</td><td>7.5
(7.8-7.2)</td><td>7.4
(7.8-7.0)</td><td>7.4
(7.8-7.0)</td><td>26
(27-25)</td><td>0.64
(0.66-0.62)</td></tr>
<tr><td>State & Local T21</td><td rowspan="2">9.8
(9.9-9.6)</td><td rowspan="2">7.3
(7.7-6.9)</td><td rowspan="2">7.1
(7.6-6.6)</td><td rowspan="2">7.1
(7.7-6.6)</td><td rowspan="2">25
(27-24)</td><td rowspan="2">0.62
(0.65-0.60)</td></tr>
<tr><td>Combined Federal T21</td></tr>
<tr><td rowspan="8">Wisconsin
(no local or state T21 coverage)</td><td rowspan="4">Men</td><td>Baseline</td><td rowspan="3">15.5</td><td rowspan="3">10.9</td><td rowspan="3">10.6</td><td rowspan="3">10.6</td><td rowspan="3">100</td><td rowspan="3">2.4</td></tr>
<tr><td>Local T21</td></tr>
<tr><td>State & Local T21</td></tr>
<tr><td>Combined Federal T21</td><td>15.2
(15.4-15)</td><td>9.9
(10.5-9.3)</td><td>9.3
(10-8.6)</td><td>9.3
(10-8.5)</td><td>92
(96-88)</td><td>2.2
(2.3-2.1)</td></tr>
<tr><td rowspan="4">Women</td><td>Baseline</td><td rowspan="3">15.7</td><td rowspan="3">12.3</td><td rowspan="3">12.3</td><td rowspan="3">12.3</td><td rowspan="3">47</td><td rowspan="3">1.0</td></tr>
<tr><td>Local T21</td></tr>
<tr><td>State & Local T21</td></tr>
<tr><td>Combined Federal T21</td><td>15.4
(15.5-15.2)</td><td>11.2
(11.8-10.6)</td><td>10.9
(11.6-10.1)</td><td>10.9
(11.6-10.1)</td><td>44
(46-43)</td><td>0.95
(0.99-0.92)</td></tr>
</table>

Notes: Numbers may appear identical due to rounding. Parentheses reflect model runs using the upper and lower bound estimates of Tobacco 21 policy effects (34% reduction to initiation; range: 15%-53%). Therefore, baseline prevalence estimates do not have ranges. Because the lower bound estimate results in higher smoking prevalence and mortality compared to the upper bound estimate, the first number in the parentheses is larger than the second. Merged cells reflect when estimates did not differ between scenarios because of the absence of policies at the local or state levels, or because the federal law was passed after a state implemented its own Tobacco 21 policy. Smoking-attributable death and life years lost estimates reflect mortality among those born in or after 1985 only.

© 2025 Tam J et al. JAMA Health Forum.

State-by-state profile of T21 results

eFigures 2-52 offer a state-by-state profile of each model with estimated adult smoking prevalence, Tobacco 21 policy coverage, and mortality reductions. These results can also be explored interactively at https://tobaccopolicyeffects.org/#t21.

© 2025 Tam J et al. JAMA Health Forum.

eFigure 2. Alabama T21 model outcomes

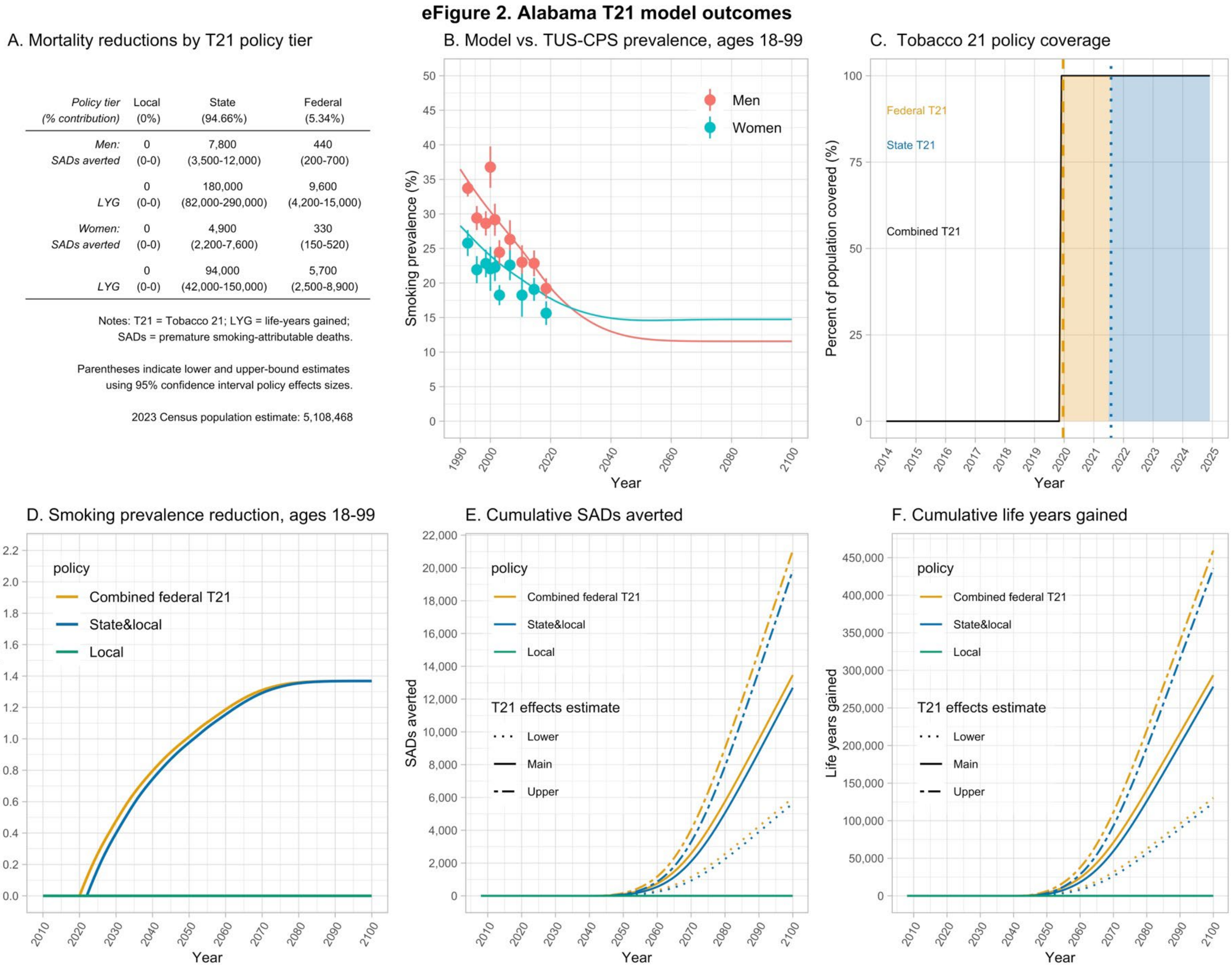

Policy tier (% contribution)	Local (0%)	State (94.66%)	Federal (5.34%)
Men: SADs averted	0 (0-0)	7,800 (3,500-12,000)	440 (200-700)
LYG	0 (0-0)	180,000 (82,000-290,000)	9,600 (4,200-15,000)
Women: SADs averted	0 (0-0)	4,900 (2,200-7,600)	330 (150-520)
LYG	0 (0-0)	94,000 (42,000-150,000)	5,700 (2,500-8,900)

Notes: T21 = Tobacco 21; LYG = life-years gained; SADs = premature smoking-attributable deaths.

Parentheses indicate lower and upper-bound estimates using 95% confidence interval policy effects sizes.

2023 Census population estimate: 5,108,468

© 2025 Tam J et al. JAMA Health Forum.

eFigure 3. Alaska T21 model outcomes

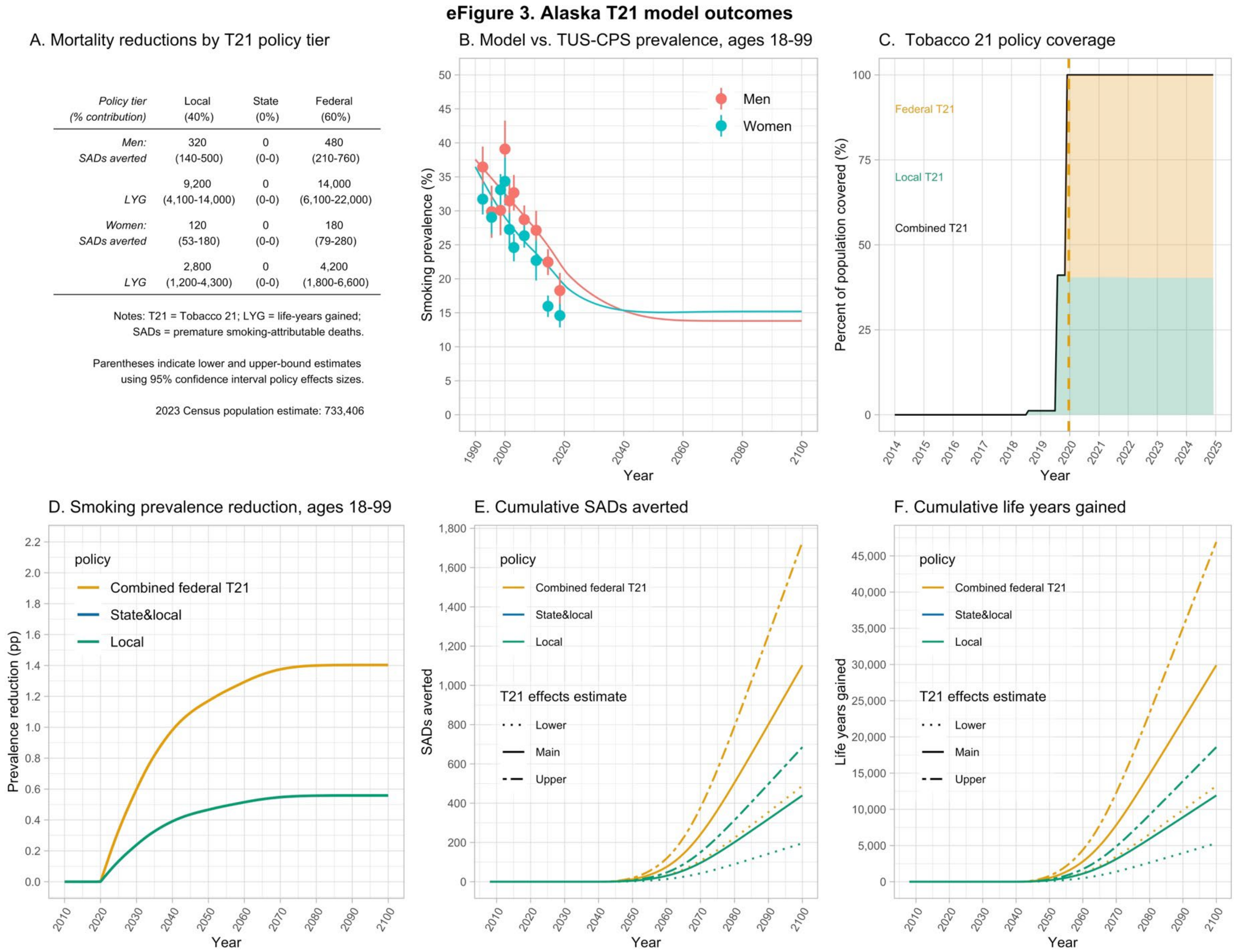

Policy tier (% contribution)	Local (40%)	State (0%)	Federal (60%)
Men: SADs averted	320 (140-500)	0 (0-0)	480 (210-760)
LYG	9,200 (4,100-14,000)	0 (0-0)	14,000 (6,100-22,000)
Women: SADs averted	120 (53-180)	0 (0-0)	180 (79-280)
LYG	2,800 (1,200-4,300)	0 (0-0)	4,200 (1,800-6,600)

© 2025 Tam J et al. JAMA Health Forum.

eFigure 4. Arizona T21 model outcomes

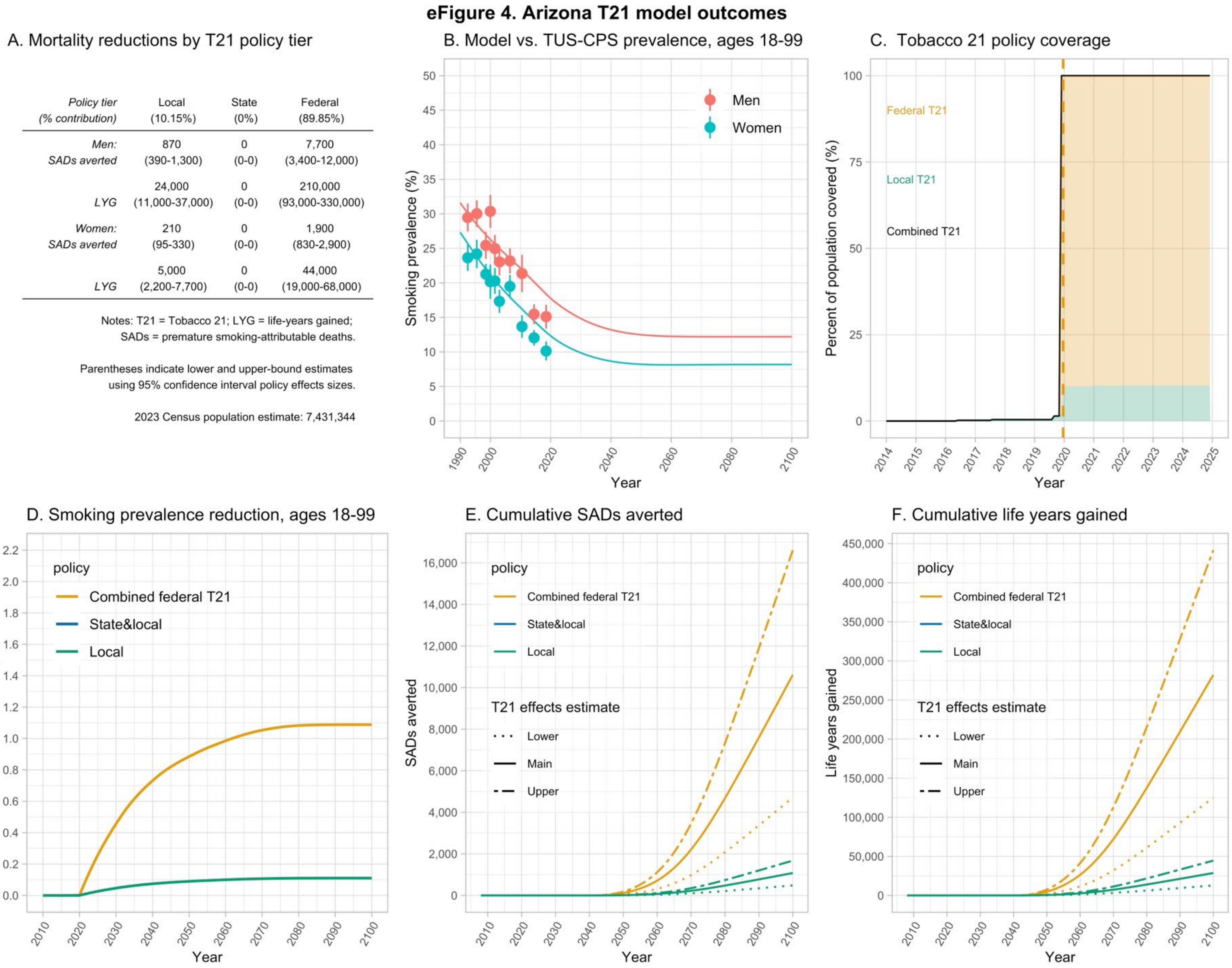

© 2025 Tam J et al. JAMA Health Forum.

eFigure 5. Arkansas T21 model outcomes

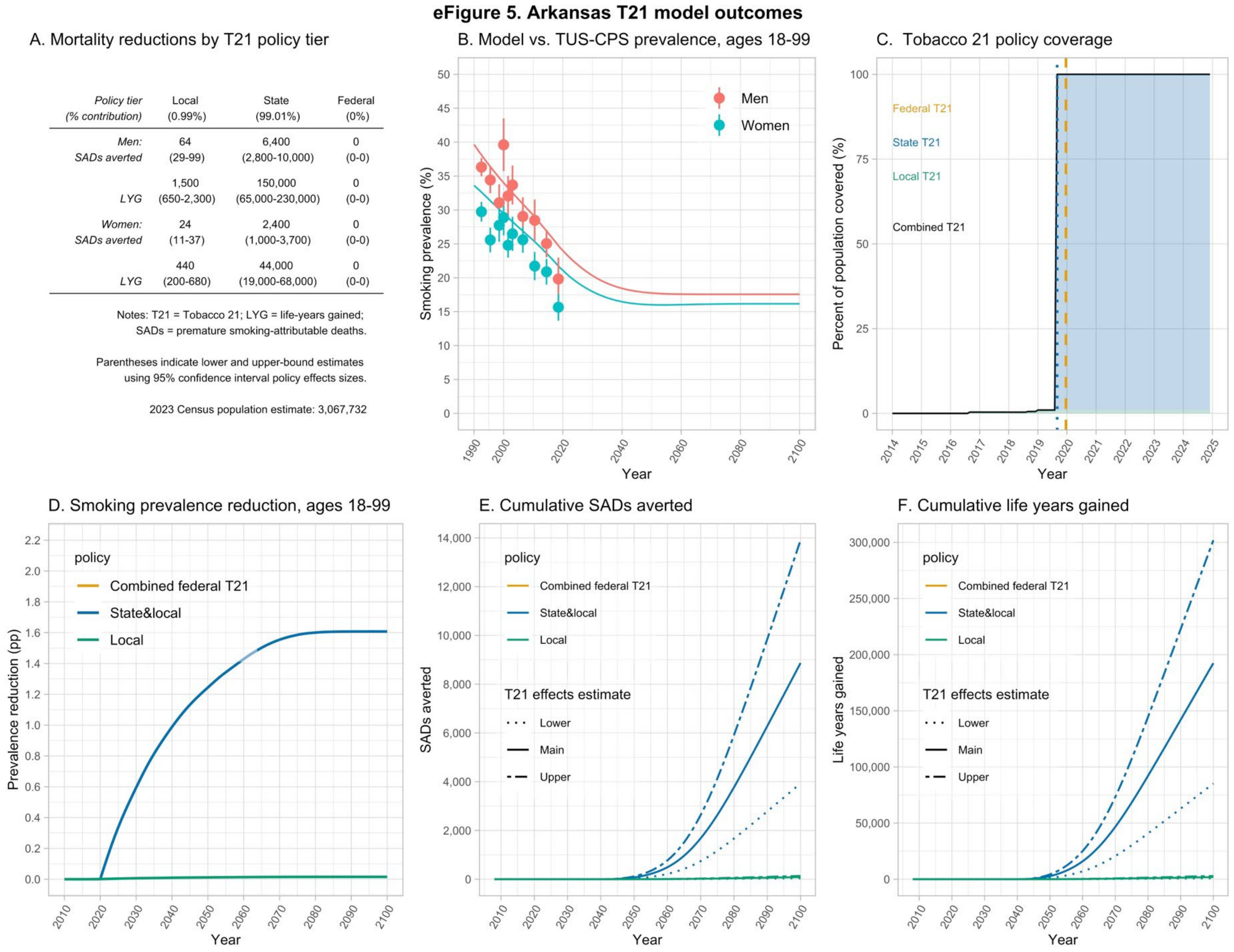

© 2025 Tam J et al. JAMA Health Forum.

eFigure 6. California T21 model outcomes

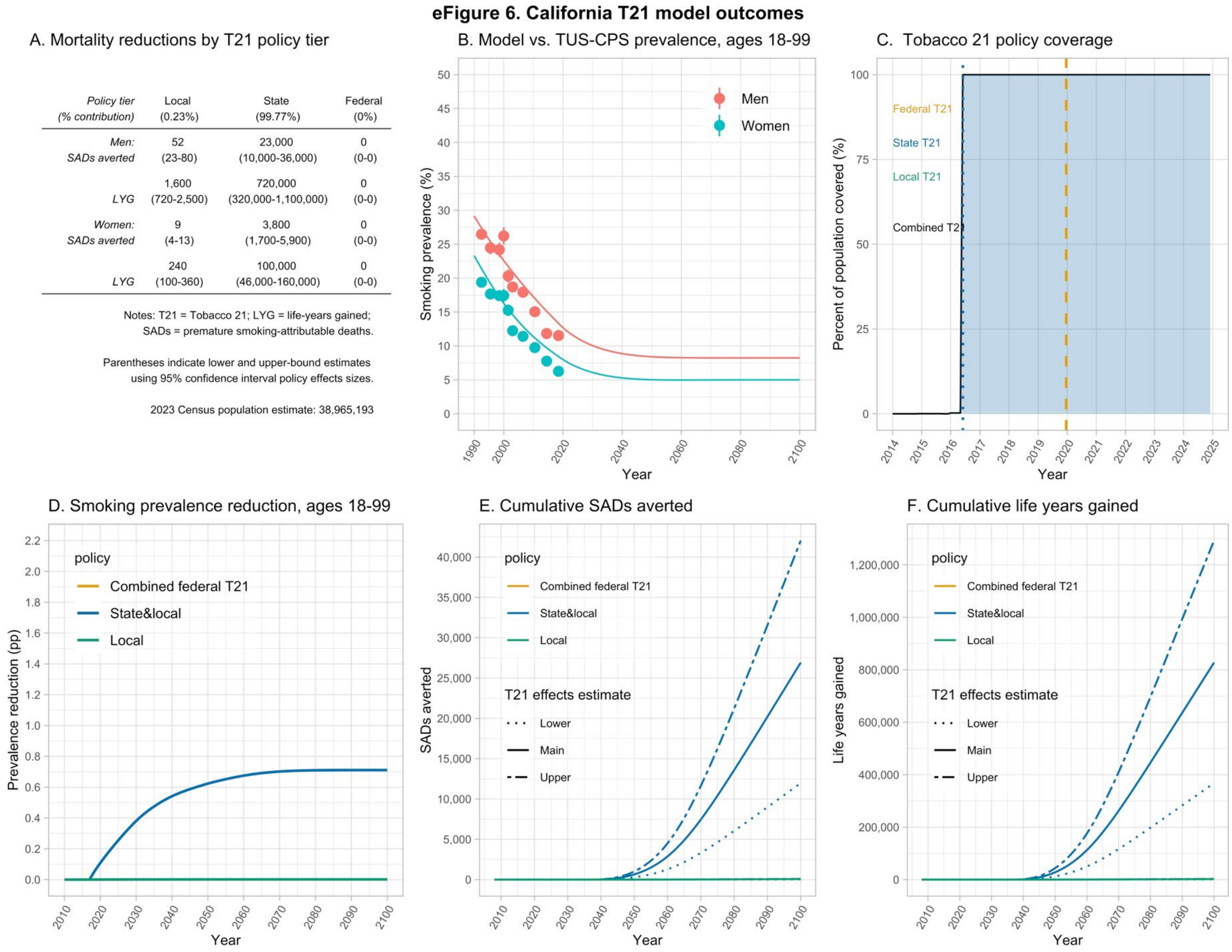

© 2025 Tam J et al. JAMA Health Forum.

© 2025 Tam J et al. JAMA Health Forum.

eFigure 7. Colorado T21 model outcomes

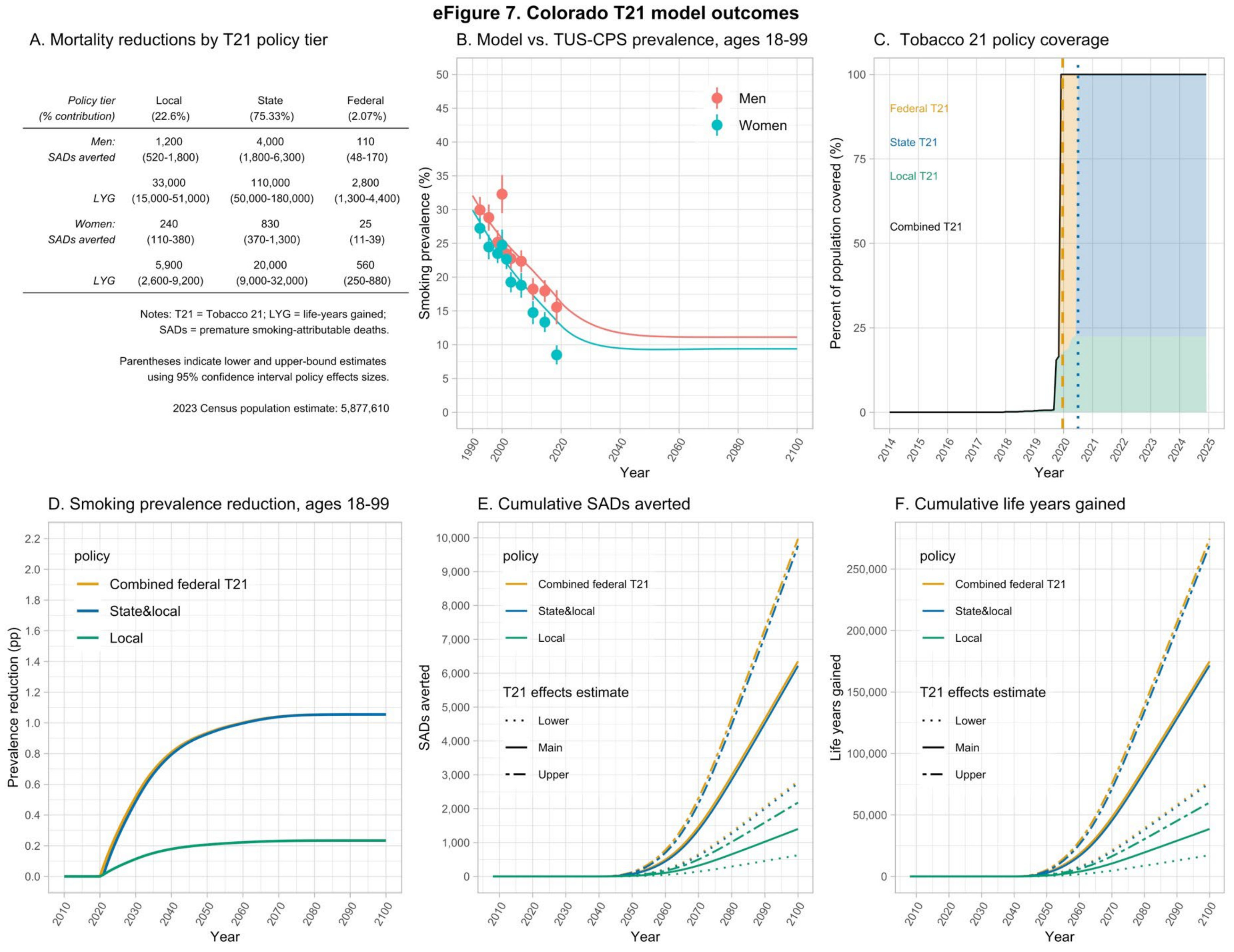

Policy tier (% contribution)	Local (22.6%)	State (75.33%)	Federal (2.07%)
Men: *SADs averted*	1,200 (520-1,800)	4,000 (1,800-6,300)	110 (48-170)
LYG	33,000 (15,000-51,000)	110,000 (50,000-180,000)	2,800 (1,300-4,400)
Women: *SADs averted*	240 (110-380)	830 (370-1,300)	25 (11-39)
LYG	5,900 (2,600-9,200)	20,000 (9,000-32,000)	560 (250-880)

© 2025 Tam J et al. JAMA Health Forum.

© 2025 Tam J et al. JAMA Health Forum.

eFigure 8. Connecticut T21 model outcomes

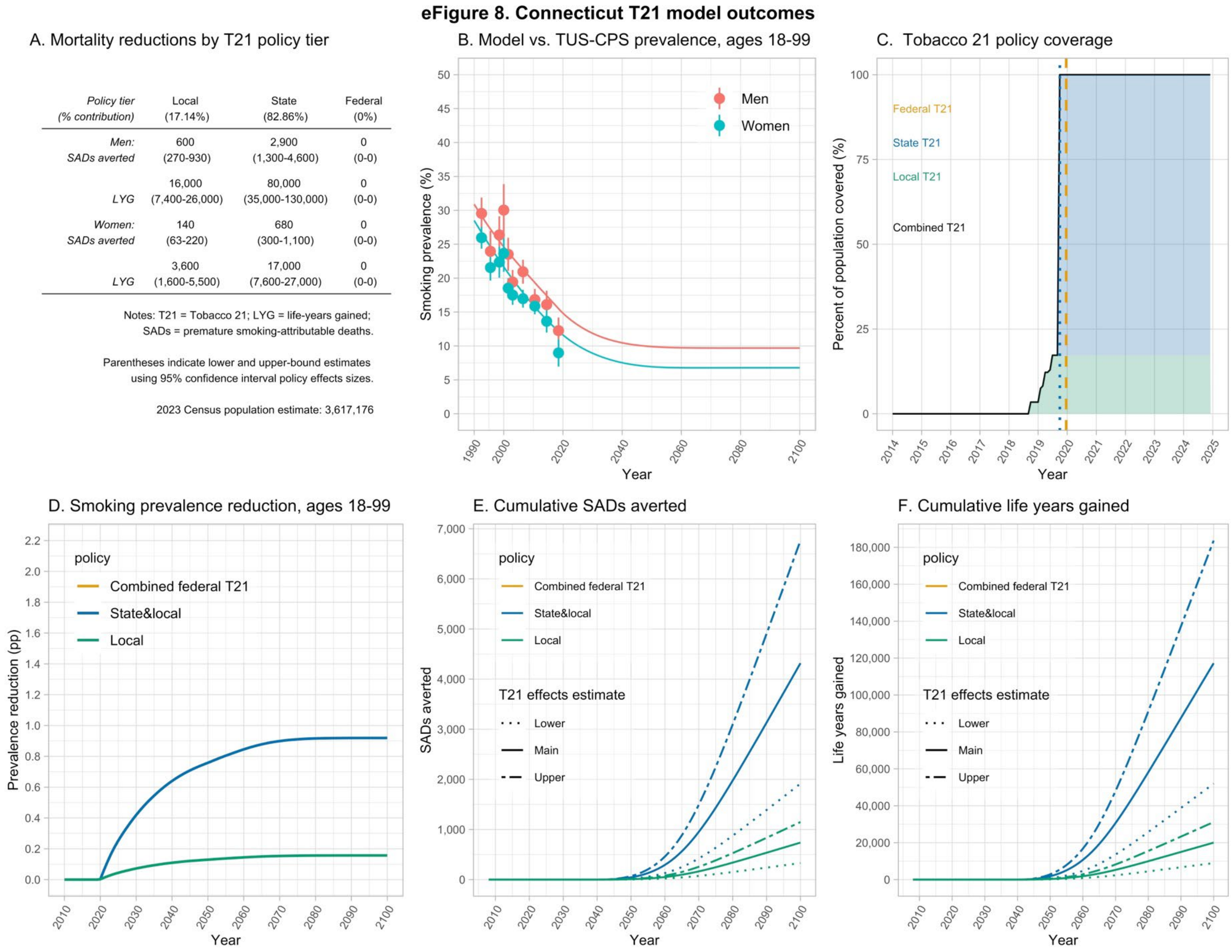

© 2025 Tam J et al. JAMA Health Forum.

© 2025 Tam J et al. JAMA Health Forum.

eFigure 9. Delaware T21 model outcomes

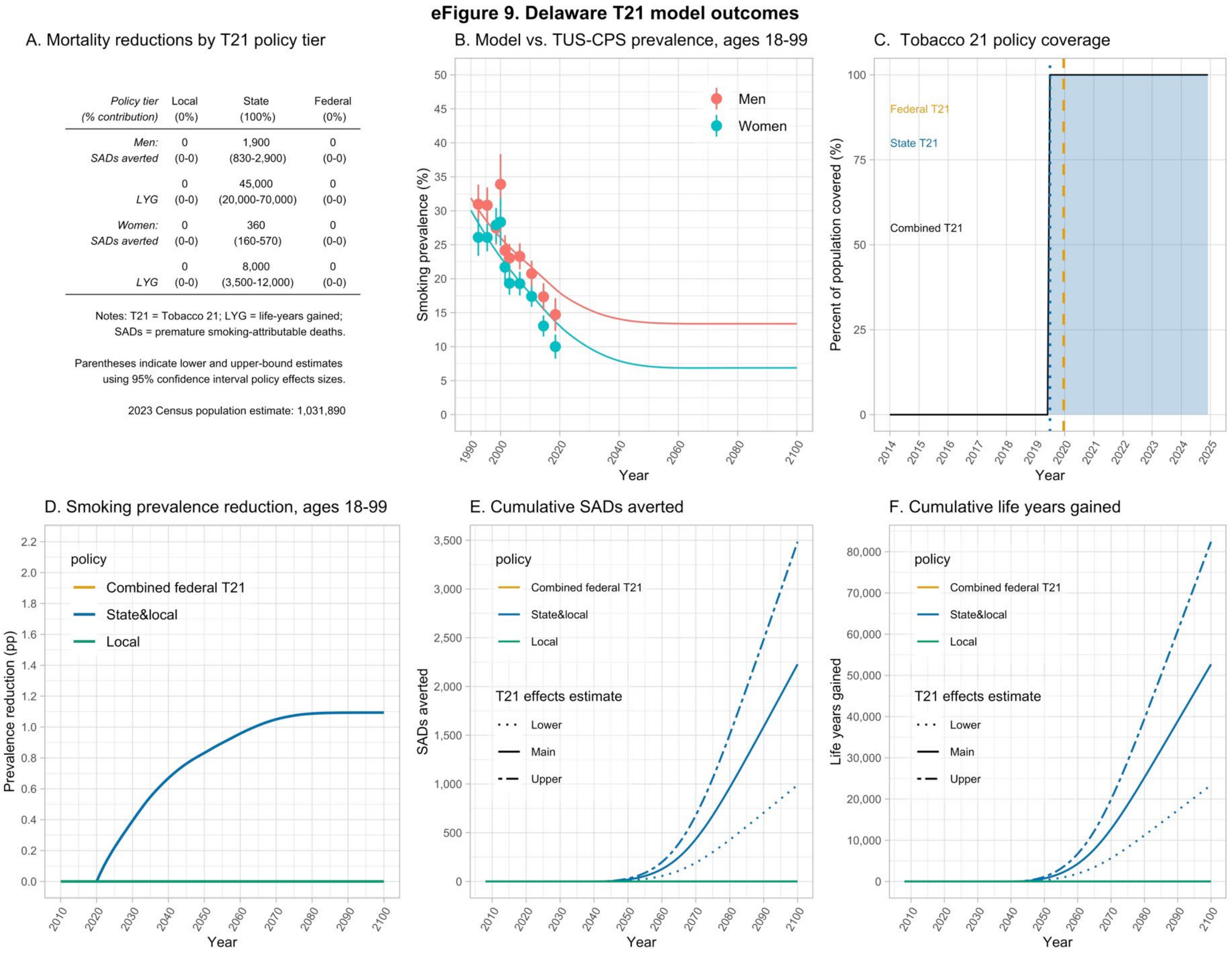

© 2025 Tam J et al. JAMA Health Forum.

© 2025 Tam J et al. JAMA Health Forum.

eFigure 10. District of Columbia T21 model outcomes

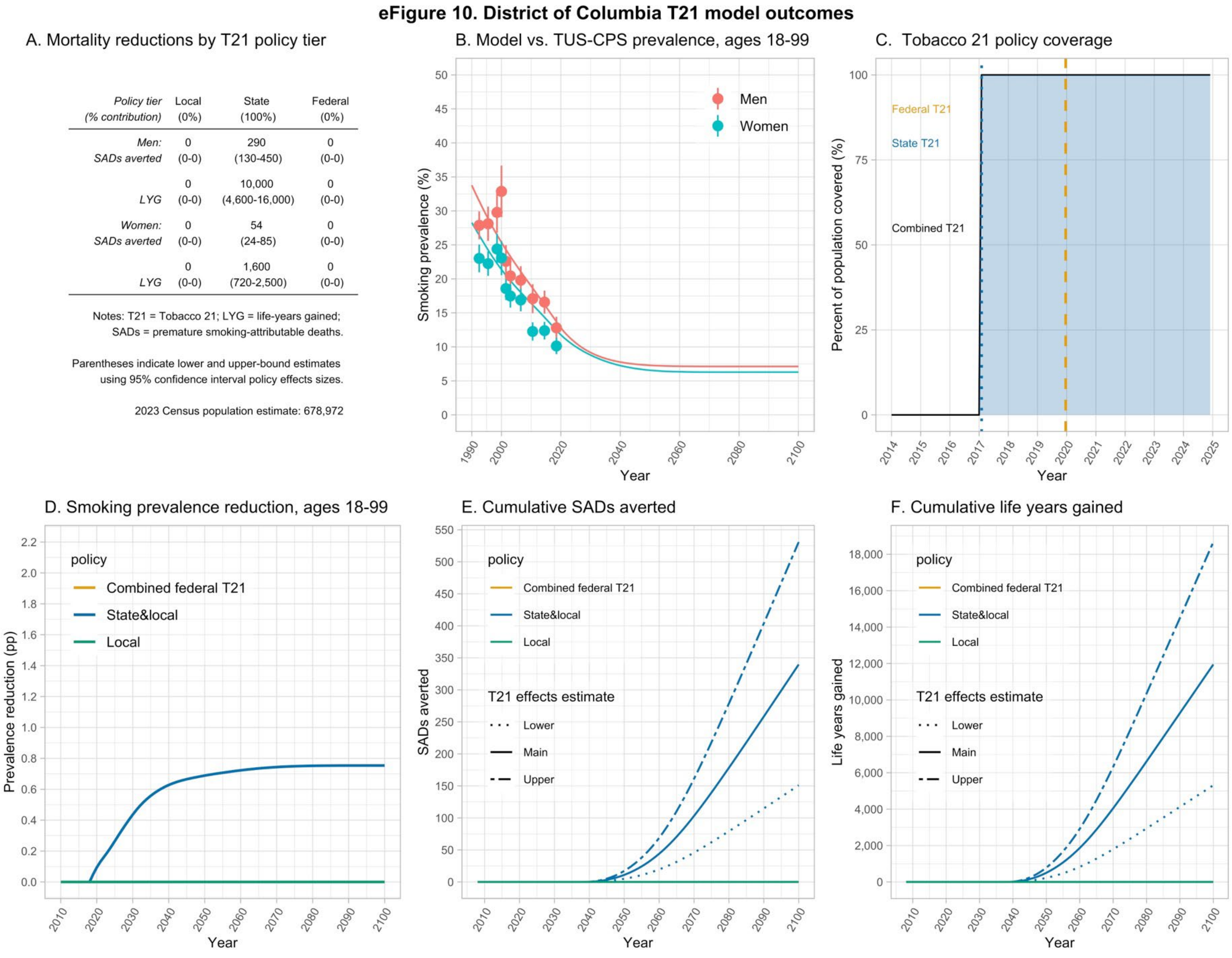

© 2025 Tam J et al. JAMA Health Forum.

© 2025 Tam J et al. JAMA Health Forum.

eFigure 11. Florida T21 model outcomes

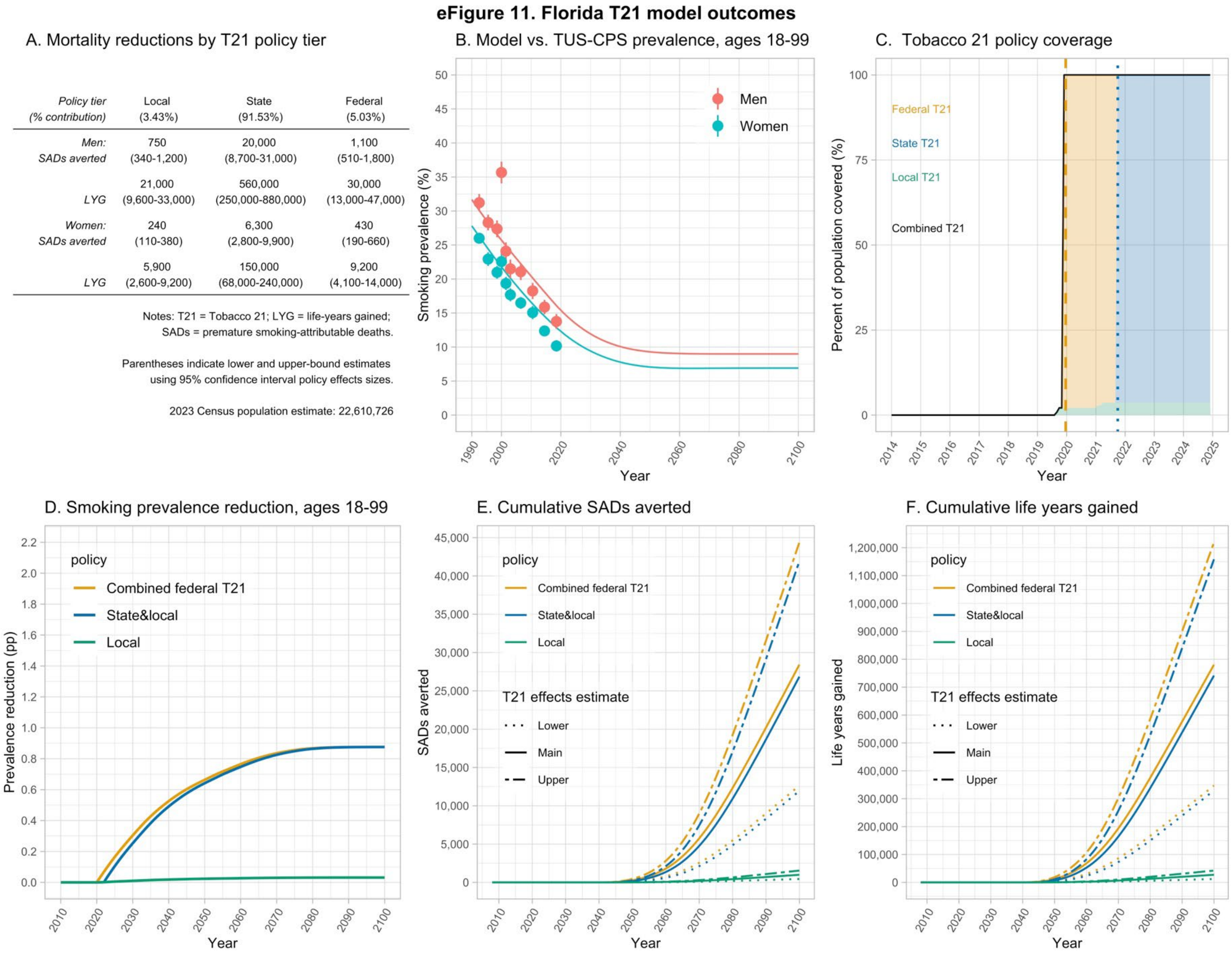

Policy tier (% contribution)	Local (3.43%)	State (91.53%)	Federal (5.03%)
Men: SADs averted	750 (340-1,200)	20,000 (8,700-31,000)	1,100 (510-1,800)
LYG	21,000 (9,600-33,000)	560,000 (250,000-880,000)	30,000 (13,000-47,000)
Women: SADs averted	240 (110-380)	6,300 (2,800-9,900)	430 (190-660)
LYG	5,900 (2,600-9,200)	150,000 (68,000-240,000)	9,200 (4,100-14,000)

Notes: T21 = Tobacco 21; LYG = life-years gained; SADs = premature smoking-attributable deaths.

Parentheses indicate lower and upper-bound estimates using 95% confidence interval policy effects sizes.

2023 Census population estimate: 22,610,726

© 2025 Tam J et al. JAMA Health Forum.

eFigure 12. Georgia T21 model outcomes

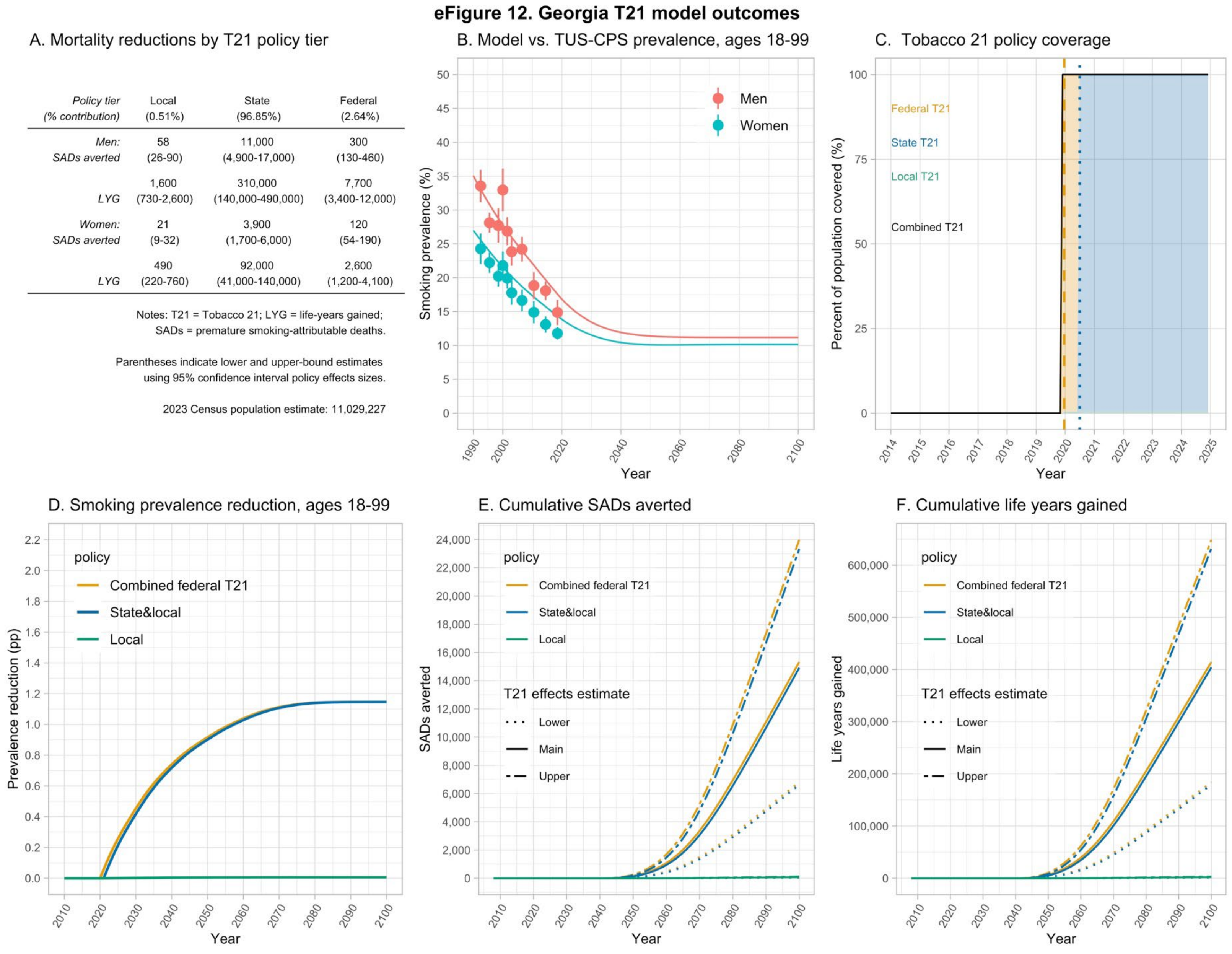

Policy tier (% contribution)	Local (0.51%)	State (96.85%)	Federal (2.64%)
Men: SADs averted	58 (26-90)	11,000 (4,900-17,000)	300 (130-460)
LYG	1,600 (730-2,600)	310,000 (140,000-490,000)	7,700 (3,400-12,000)
Women: SADs averted	21 (9-32)	3,900 (1,700-6,000)	120 (54-190)
LYG	490 (220-760)	92,000 (41,000-140,000)	2,600 (1,200-4,100)

Notes: T21 = Tobacco 21; LYG = life-years gained; SADs = premature smoking-attributable deaths.

Parentheses indicate lower and upper-bound estimates using 95% confidence interval policy effects sizes.

2023 Census population estimate: 11,029,227

© 2025 Tam J et al. JAMA Health Forum.

eFigure 13. Hawaii T21 model outcomes

eFigure 13. Hawaii T21 model outcomes

A. Mortality reductions by T21 policy tier

Policy tier (% contribution)	Local (13.67%)	State (86.33%)	Federal (0%)
Men: SADs averted	190 (85-300)	1,200 (520-1,900)	0 (0-0)
LYG	5,300 (2,400-8,200)	33,000 (15,000-51,000)	0 (0-0)
Women: SADs averted	49 (22-76)	300 (130-470)	0 (0-0)
LYG	1,300 (580-2,000)	8,000 (3,600-13,000)	0 (0-0)

Notes: T21 = Tobacco 21; LYG = life-years gained; SADs = premature smoking-attributable deaths.

Parentheses indicate lower and upper-bound estimates using 95% confidence interval policy effects sizes.

2023 Census population estimate: 1,435,138

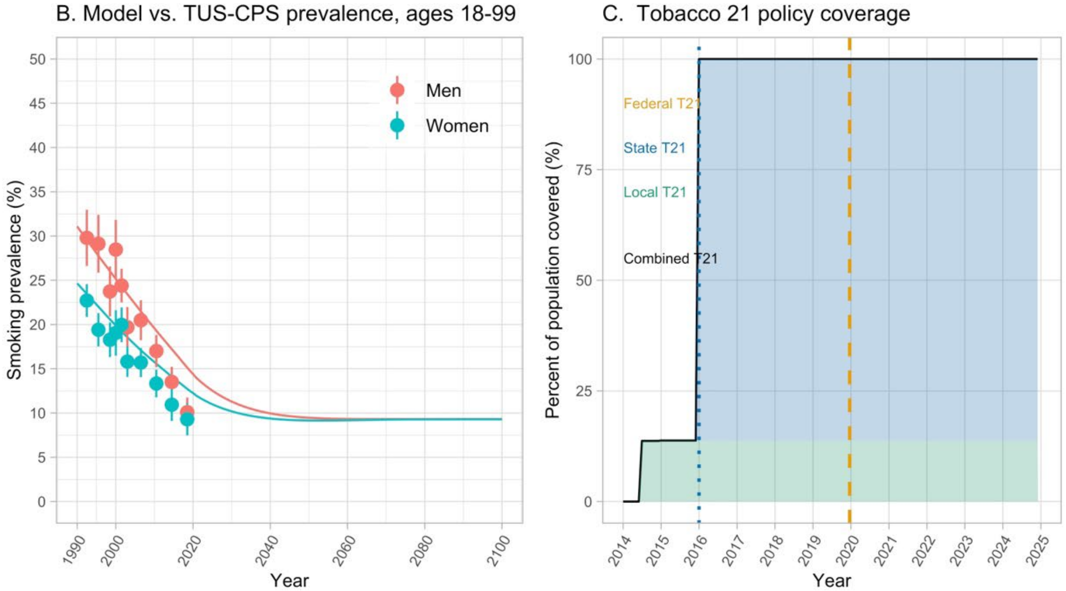

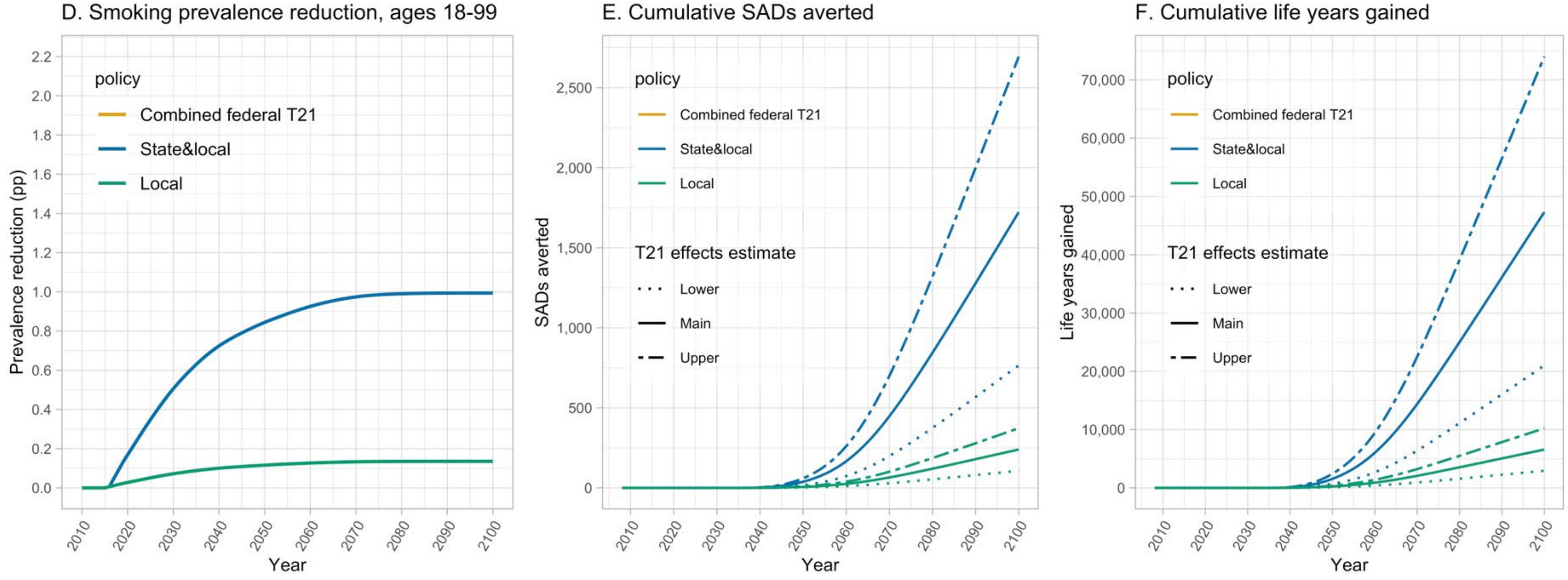

© 2025 Tam J et al. JAMA Health Forum.

eFigure 14. Idaho T21 model outcomes

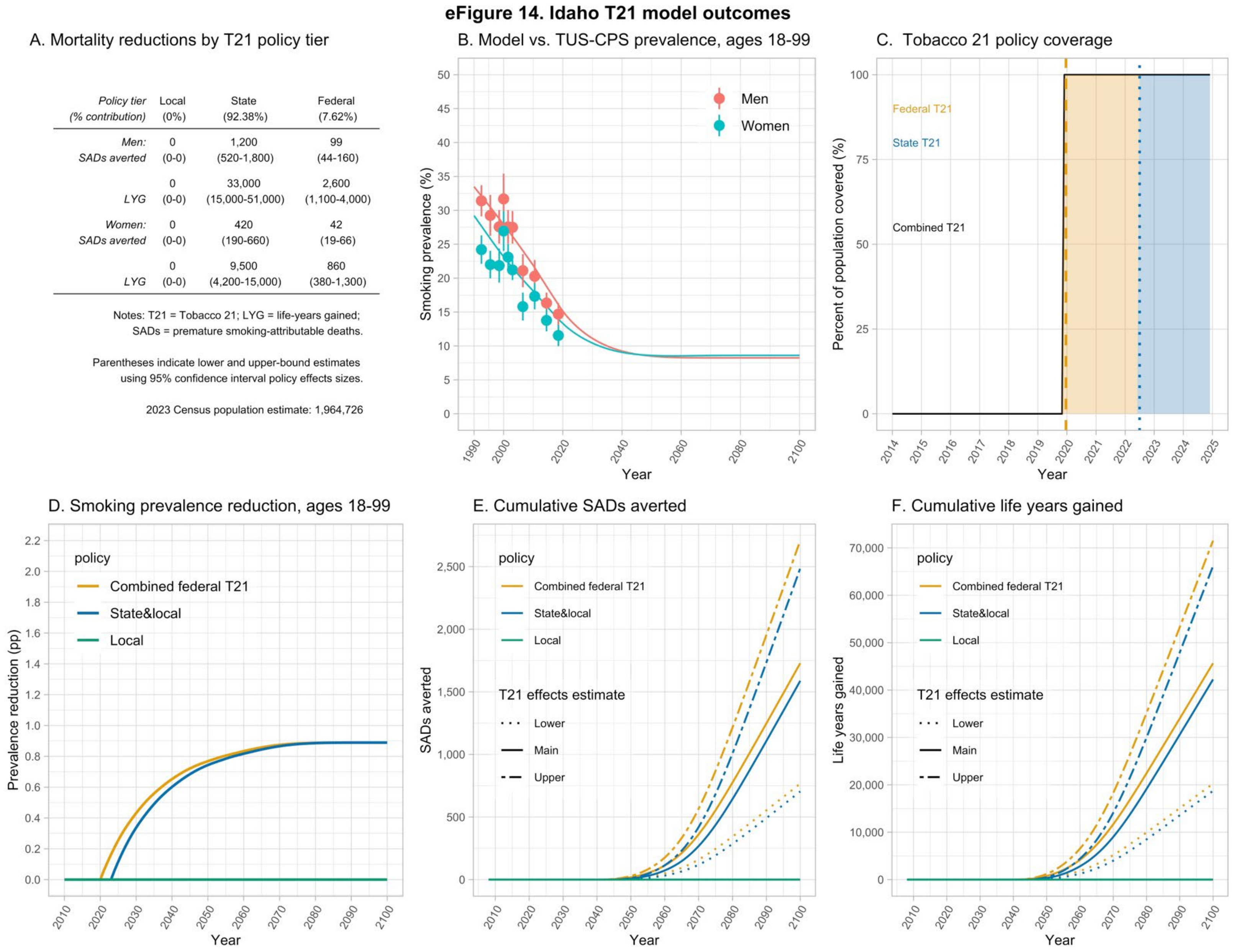

© 2025 Tam J et al. JAMA Health Forum.

© 2025 Tam J et al. JAMA Health Forum.

eFigure 15. Illinois T21 model outcomes

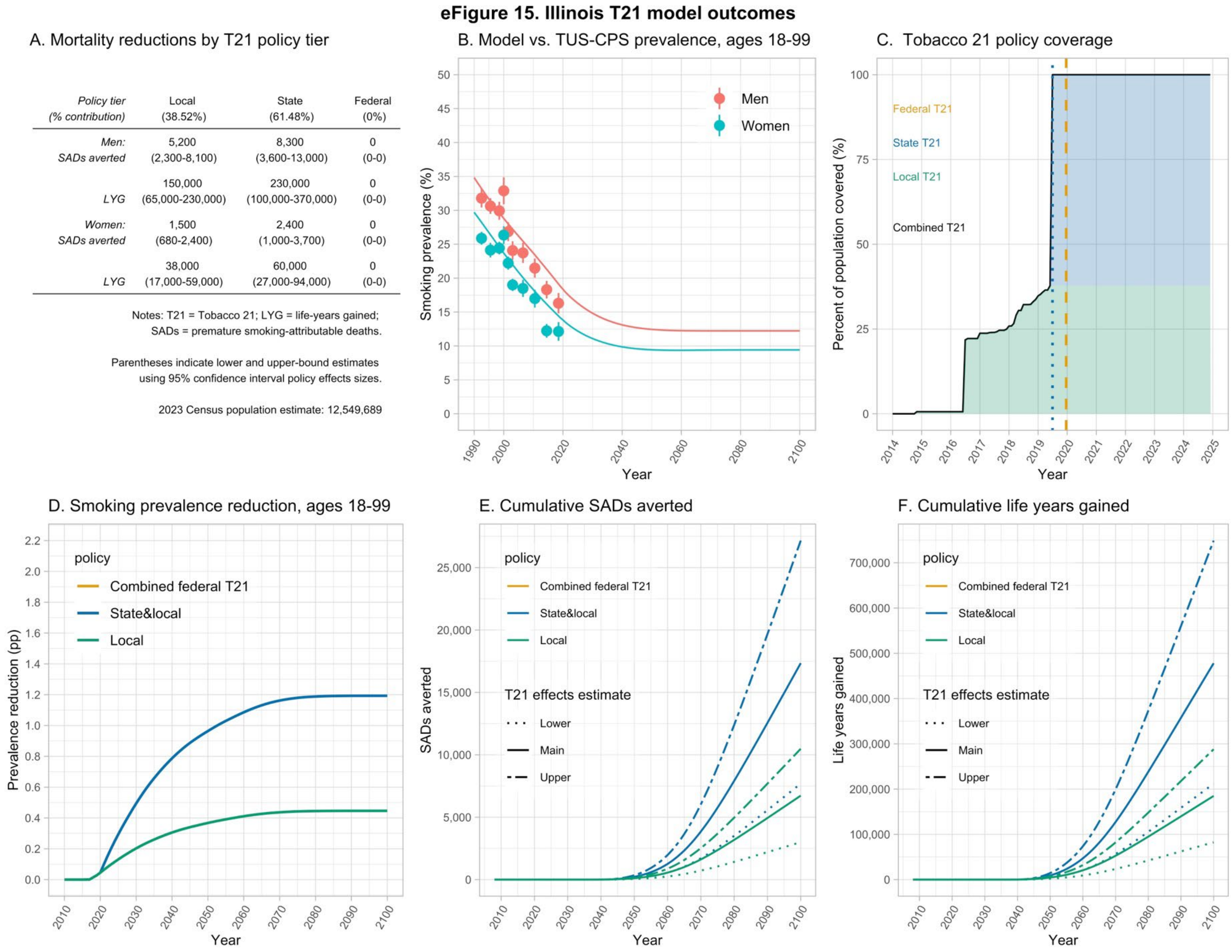

Policy tier (% contribution)	Local (38.52%)	State (61.48%)	Federal (0%)
Men: SADs averted	5,200 (2,300-8,100)	8,300 (3,600-13,000)	0 (0-0)
LYG	150,000 (65,000-230,000)	230,000 (100,000-370,000)	0 (0-0)
Women: SADs averted	1,500 (680-2,400)	2,400 (1,000-3,700)	0 (0-0)
LYG	38,000 (17,000-59,000)	60,000 (27,000-94,000)	0 (0-0)

Notes: T21 = Tobacco 21; LYG = life-years gained; SADs = premature smoking-attributable deaths.

Parentheses indicate lower and upper-bound estimates using 95% confidence interval policy effects sizes.

2023 Census population estimate: 12,549,689

© 2025 Tam J et al. JAMA Health Forum.

eFigure 16. Indiana T21 model outcomes

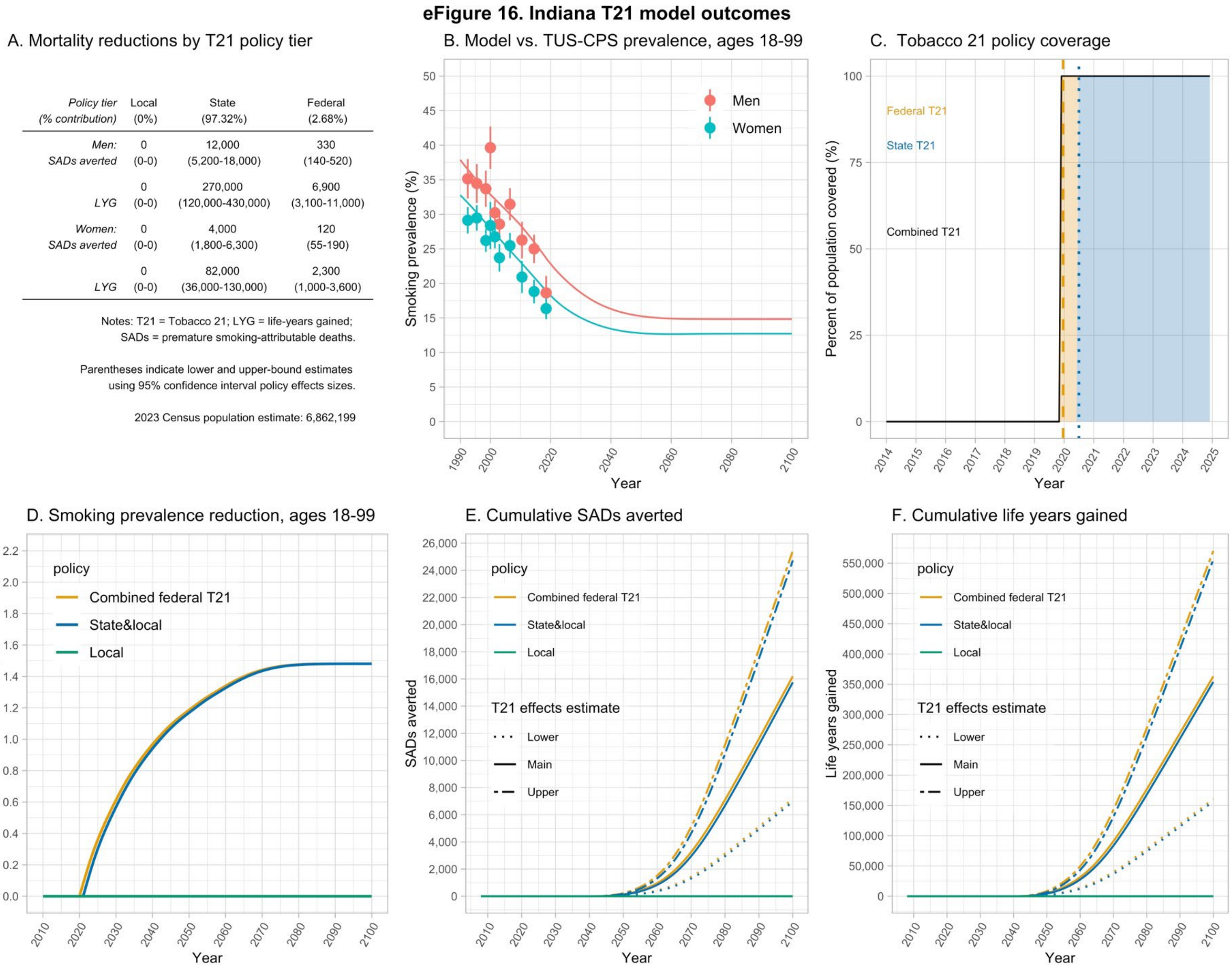

© 2025 Tam J et al. JAMA Health Forum.

© 2025 Tam J et al. JAMA Health Forum.

eFigure 17. Iowa T21 model outcomes

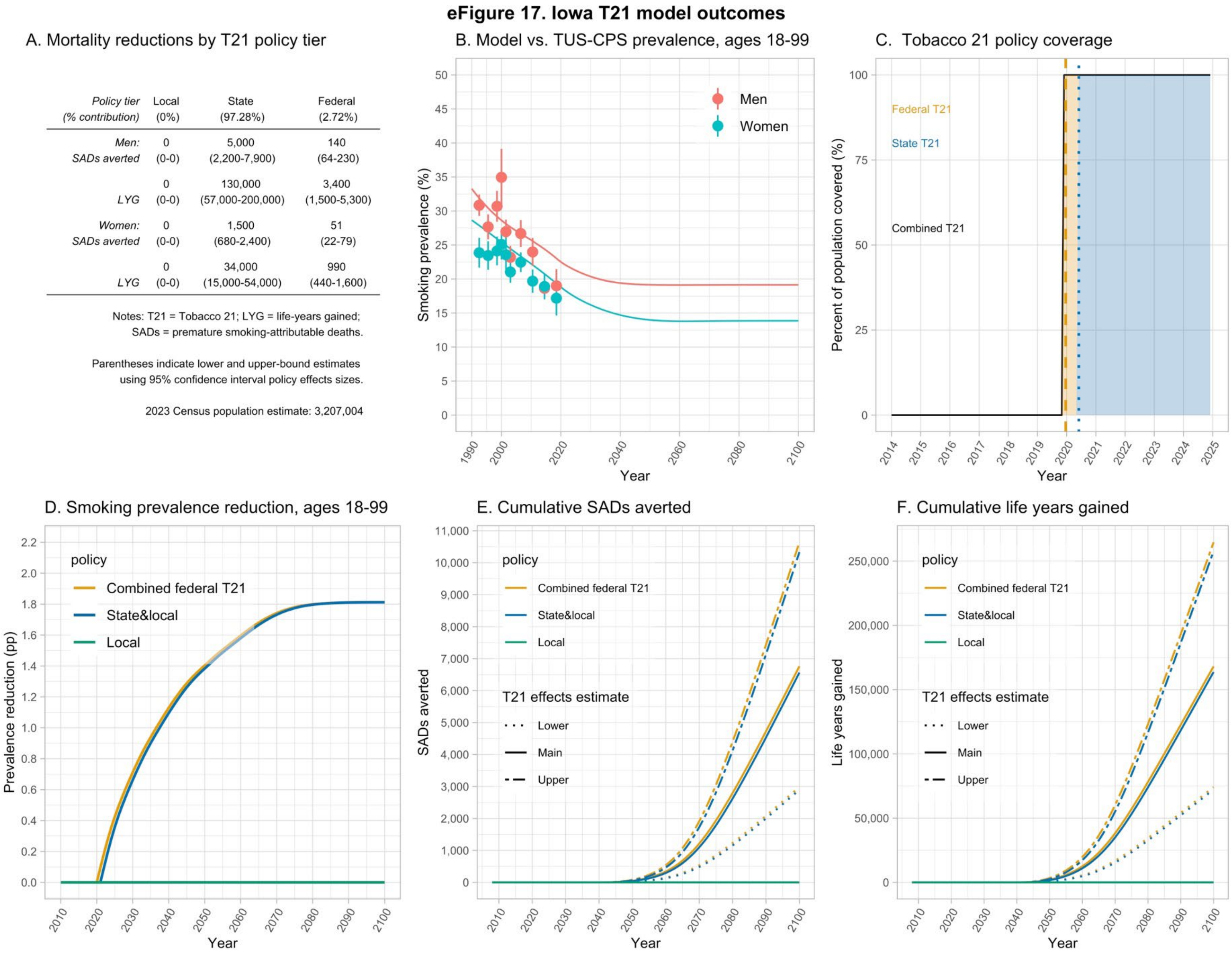

© 2025 Tam J et al. JAMA Health Forum.

© 2025 Tam J et al. JAMA Health Forum.

eFigure 18. Kansas T21 model outcomes

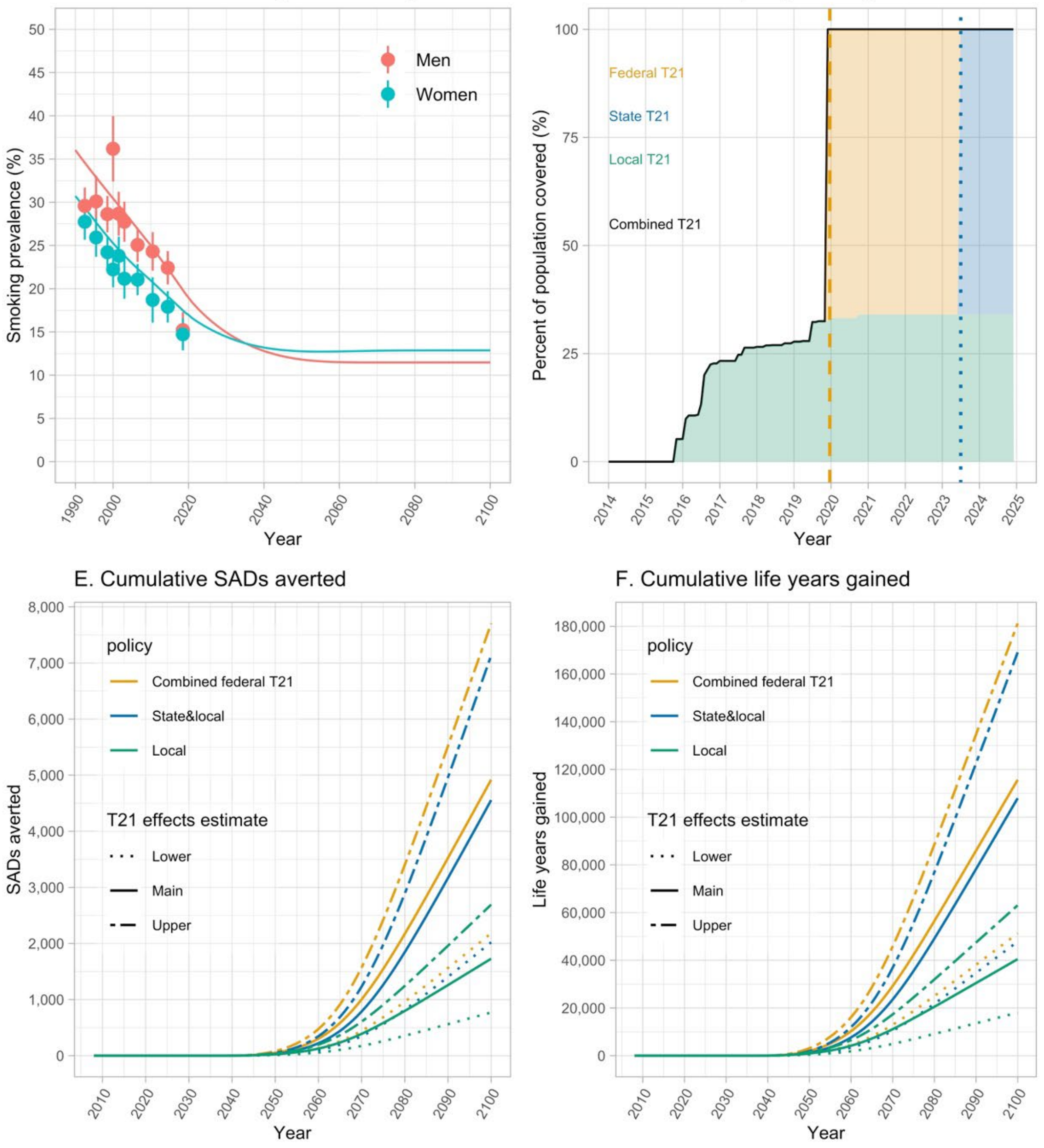

Policy tier (% contribution)	Local (34.88%)	State (58.14%)	Federal (6.98%)
Men: SADs averted	1,200 (540-1,900)	2,000 (880-3,100)	240 (110-380)
LYG	29,000 (13,000-46,000)	49,000 (22,000-77,000)	5,400 (2,400-8,500)
Women: SADs averted	530 (240-820)	840 (370-1,300)	120 (54-190)
LYG	11,000 (5,000-17,000)	18,000 (8,100-29,000)	2,300 (1,000-3,700)

Notes: T21 = Tobacco 21; LYG = life-years gained; SADs = premature smoking-attributable deaths.

Parentheses indicate lower and upper-bound estimates using 95% confidence interval policy effects sizes.

2023 Census population estimate: 2,940,546

© 2025 Tam J et al. JAMA Health Forum.

© 2025 Tam J et al. JAMA Health Forum.

eFigure 19. Kentucky T21 model outcomes

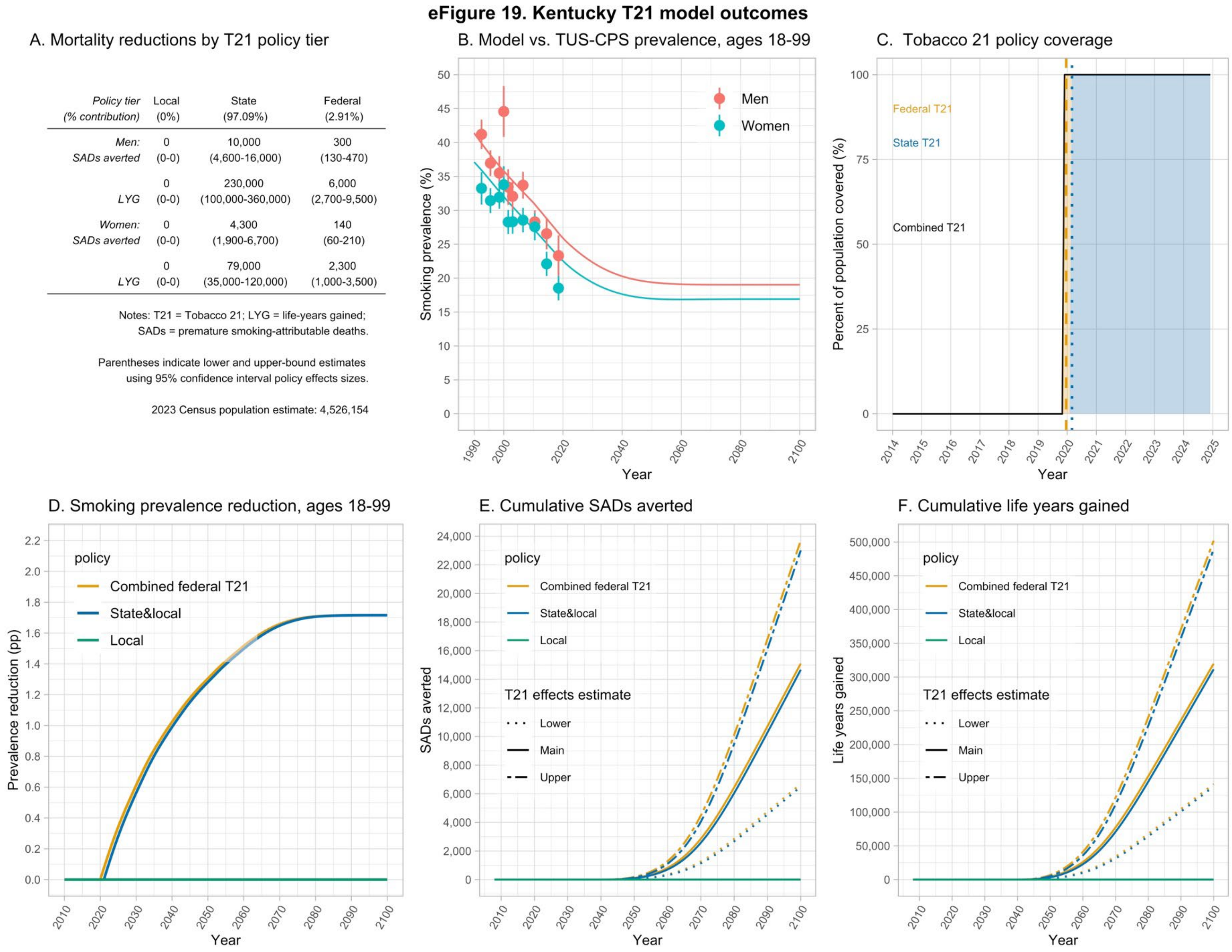

Policy tier (% contribution)	Local (0%)	State (97.09%)	Federal (2.91%)
Men: SADs averted	0 (0-0)	10,000 (4,600-16,000)	300 (130-470)
LYG	0 (0-0)	230,000 (100,000-360,000)	6,000 (2,700-9,500)
Women: SADs averted	0 (0-0)	4,300 (1,900-6,700)	140 (60-210)
LYG	0 (0-0)	79,000 (35,000-120,000)	2,300 (1,000-3,500)

Notes: T21 = Tobacco 21; LYG = life-years gained; SADs = premature smoking-attributable deaths.

Parentheses indicate lower and upper-bound estimates using 95% confidence interval policy effects sizes.

2023 Census population estimate: 4,526,154

© 2025 Tam J et al. JAMA Health Forum.

© 2025 Tam J et al. JAMA Health Forum.

eFigure 20. Louisiana T21 model outcomes

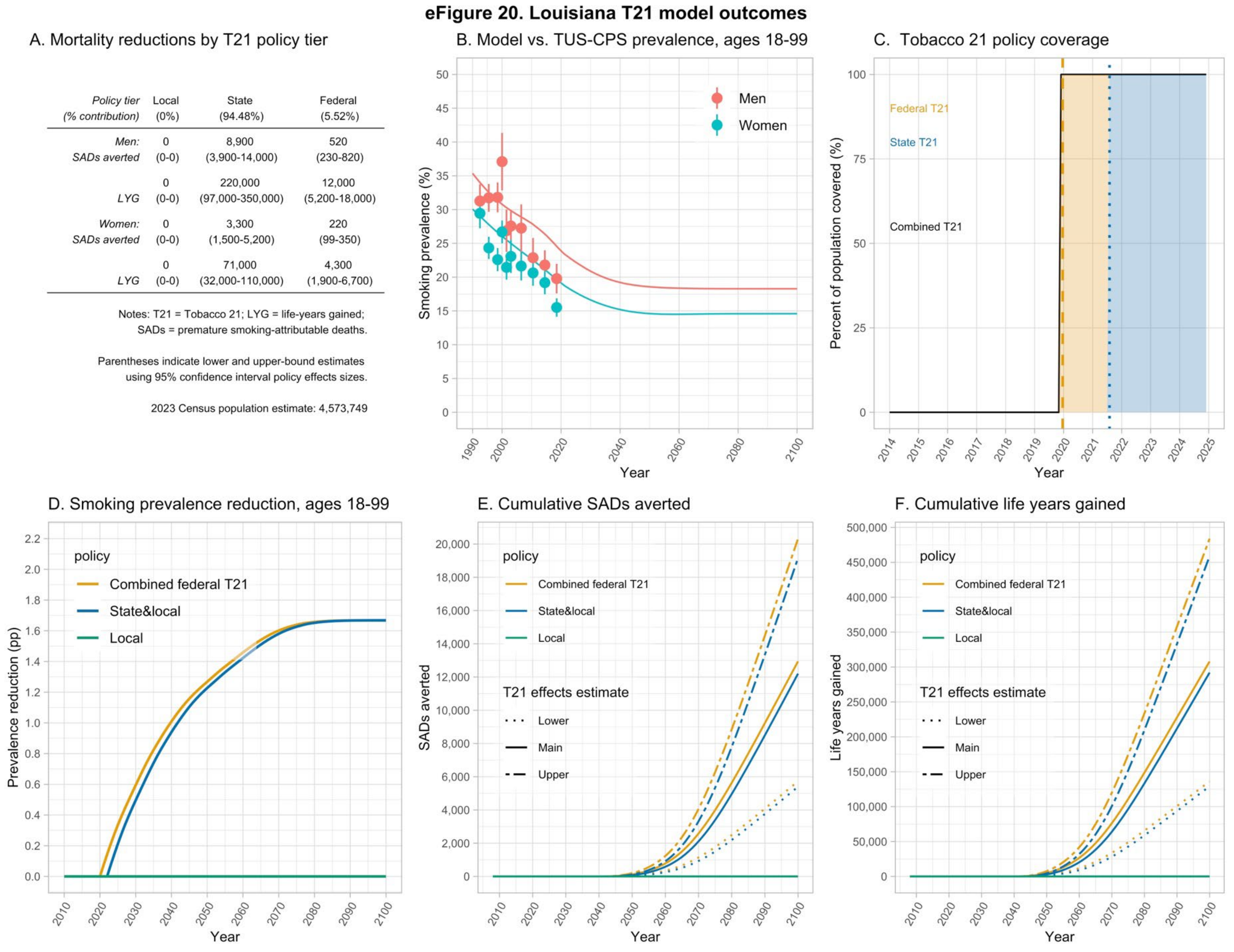

© 2025 Tam J et al. JAMA Health Forum.

© 2025 Tam J et al. JAMA Health Forum.

eFigure 21. Maine T21 model outcomes

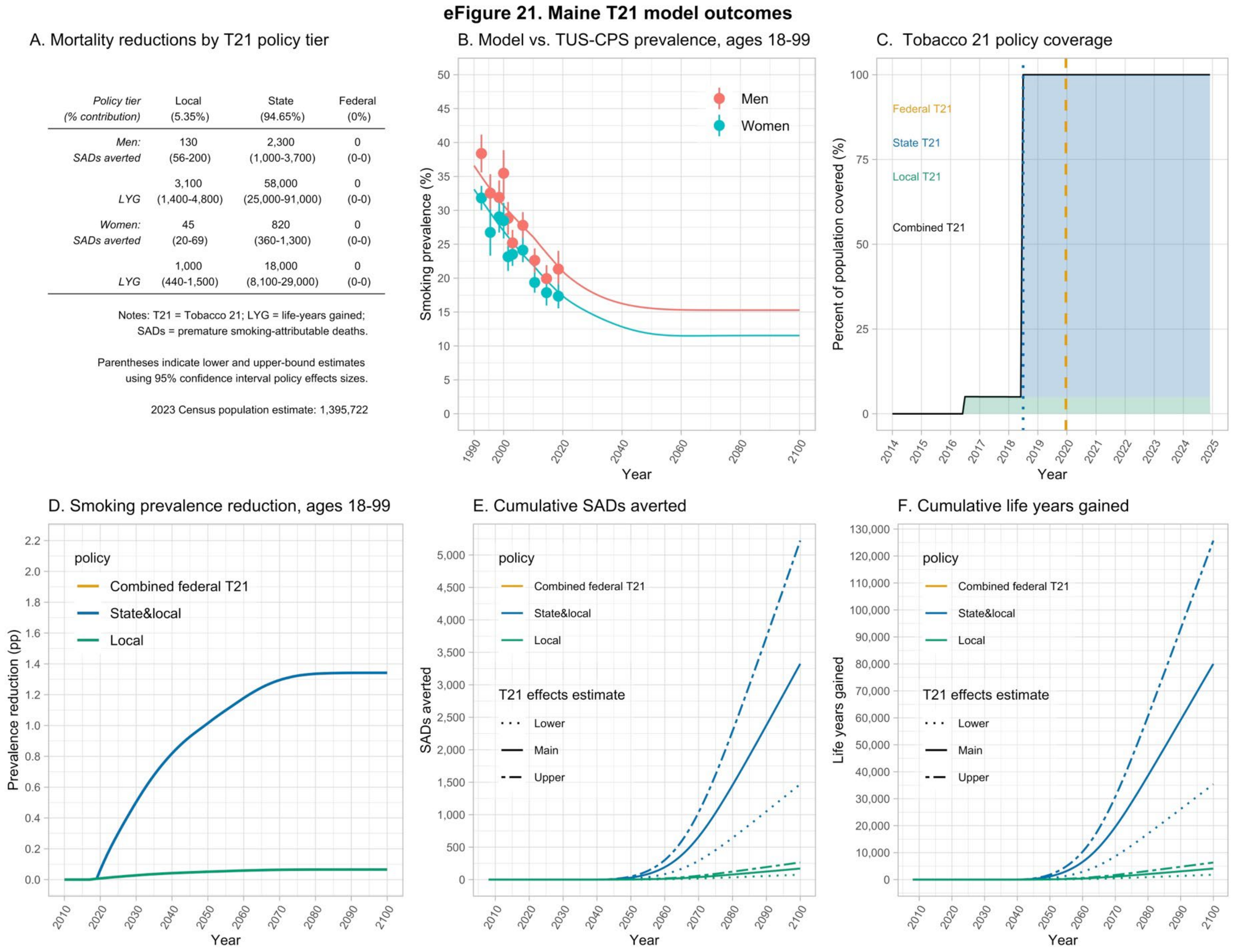

Policy tier (% contribution)	Local (5.35%)	State (94.65%)	Federal (0%)
Men: SADs averted	130 (56-200)	2,300 (1,000-3,700)	0 (0-0)
LYG	3,100 (1,400-4,800)	58,000 (25,000-91,000)	0 (0-0)
Women: SADs averted	45 (20-69)	820 (360-1,300)	0 (0-0)
LYG	1,000 (440-1,500)	18,000 (8,100-29,000)	0 (0-0)

Notes: T21 = Tobacco 21; LYG = life-years gained; SADs = premature smoking-attributable deaths.

Parentheses indicate lower and upper-bound estimates using 95% confidence interval policy effects sizes.

2023 Census population estimate: 1,395,722

© 2025 Tam J et al. JAMA Health Forum.

© 2025 Tam J et al. JAMA Health Forum.

eFigure 22. Maryland T21 model outcomes

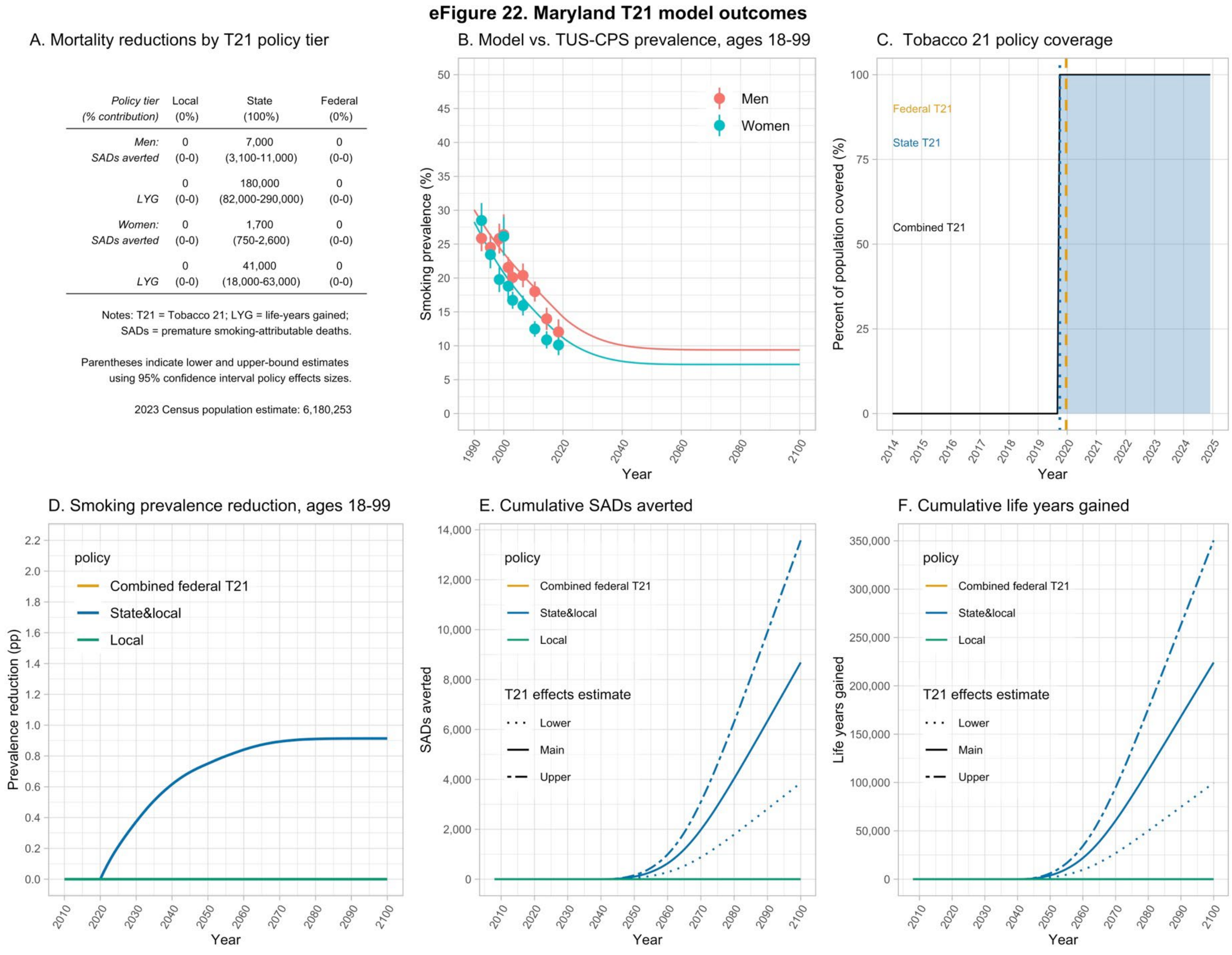

© 2025 Tam J et al. JAMA Health Forum.

© 2025 Tam J et al. JAMA Health Forum.

eFigure 23. Massachusetts T21 model outcomes

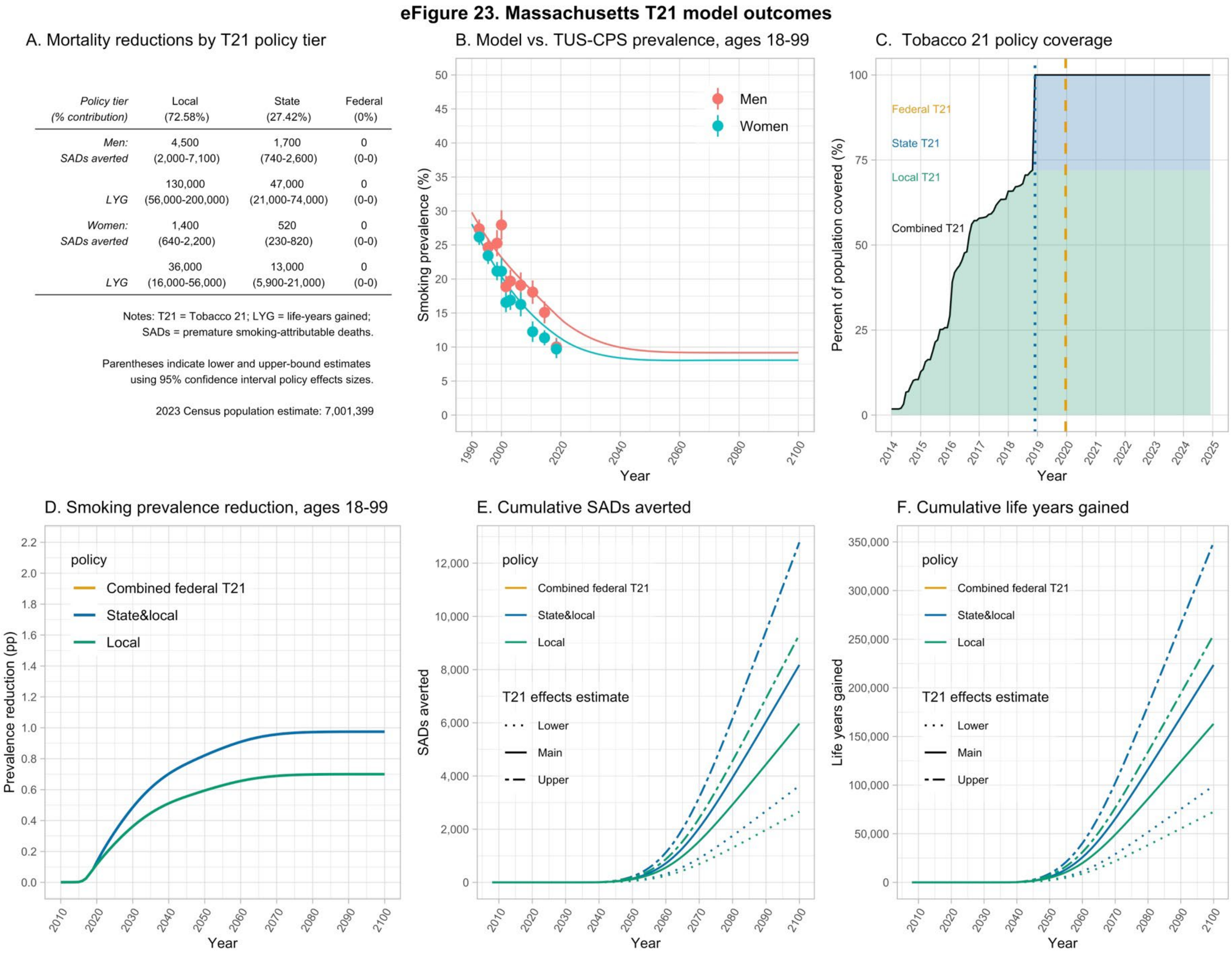

© 2025 Tam J et al. JAMA Health Forum.

© 2025 Tam J et al. JAMA Health Forum.

eFigure 24. Michigan T21 model outcomes

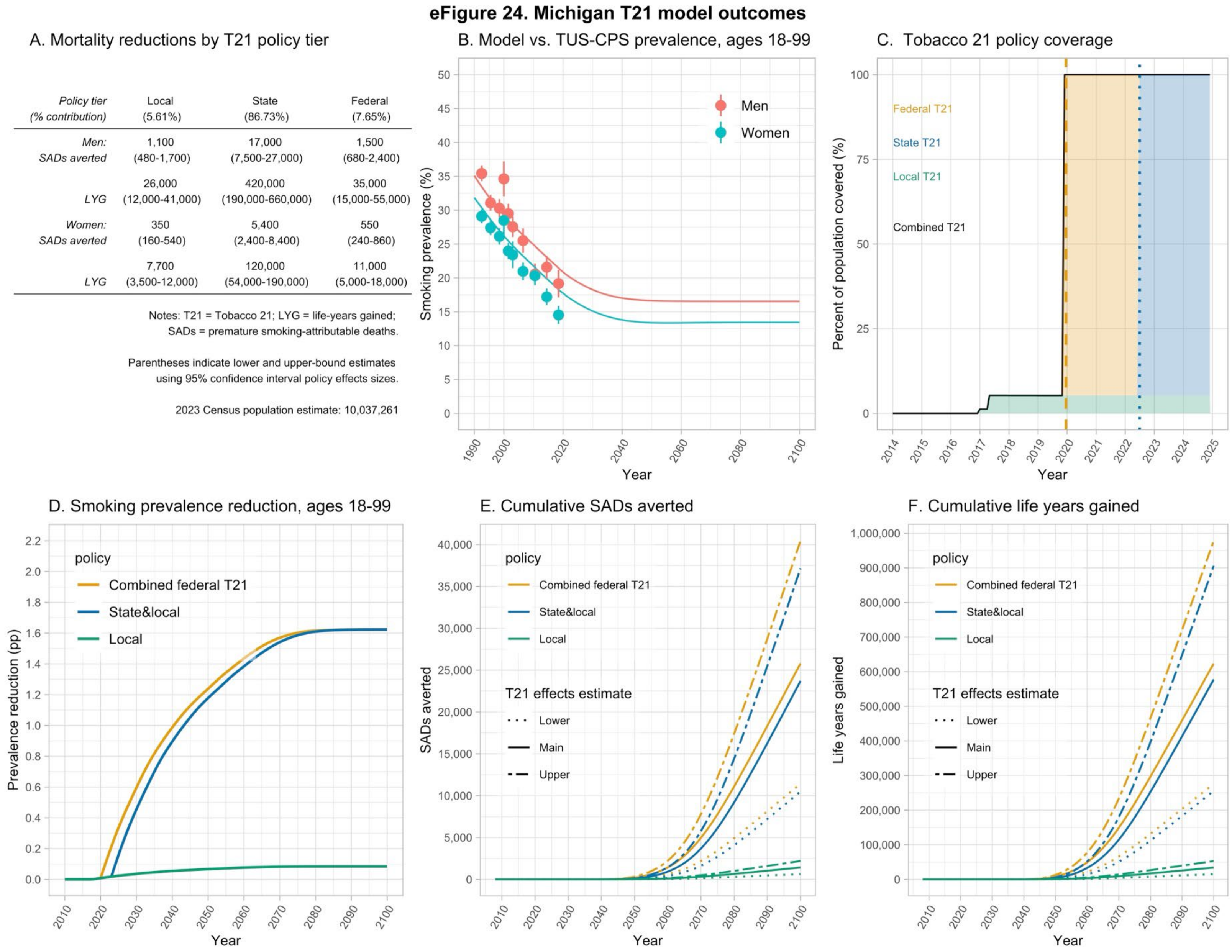

Policy tier (% contribution)	Local (5.61%)	State (86.73%)	Federal (7.65%)
Men: SADs averted	1,100 (480-1,700)	17,000 (7,500-27,000)	1,500 (680-2,400)
LYG	26,000 (12,000-41,000)	420,000 (190,000-660,000)	35,000 (15,000-55,000)
Women: SADs averted	350 (160-540)	5,400 (2,400-8,400)	550 (240-860)
LYG	7,700 (3,500-12,000)	120,000 (54,000-190,000)	11,000 (5,000-18,000)

Notes: T21 = Tobacco 21; LYG = life-years gained; SADs = premature smoking-attributable deaths.

Parentheses indicate lower and upper-bound estimates using 95% confidence interval policy effects sizes.

2023 Census population estimate: 10,037,261

© 2025 Tam J et al. JAMA Health Forum.

© 2025 Tam J et al. JAMA Health Forum.

eFigure 25. Minnesota T21 model outcomes

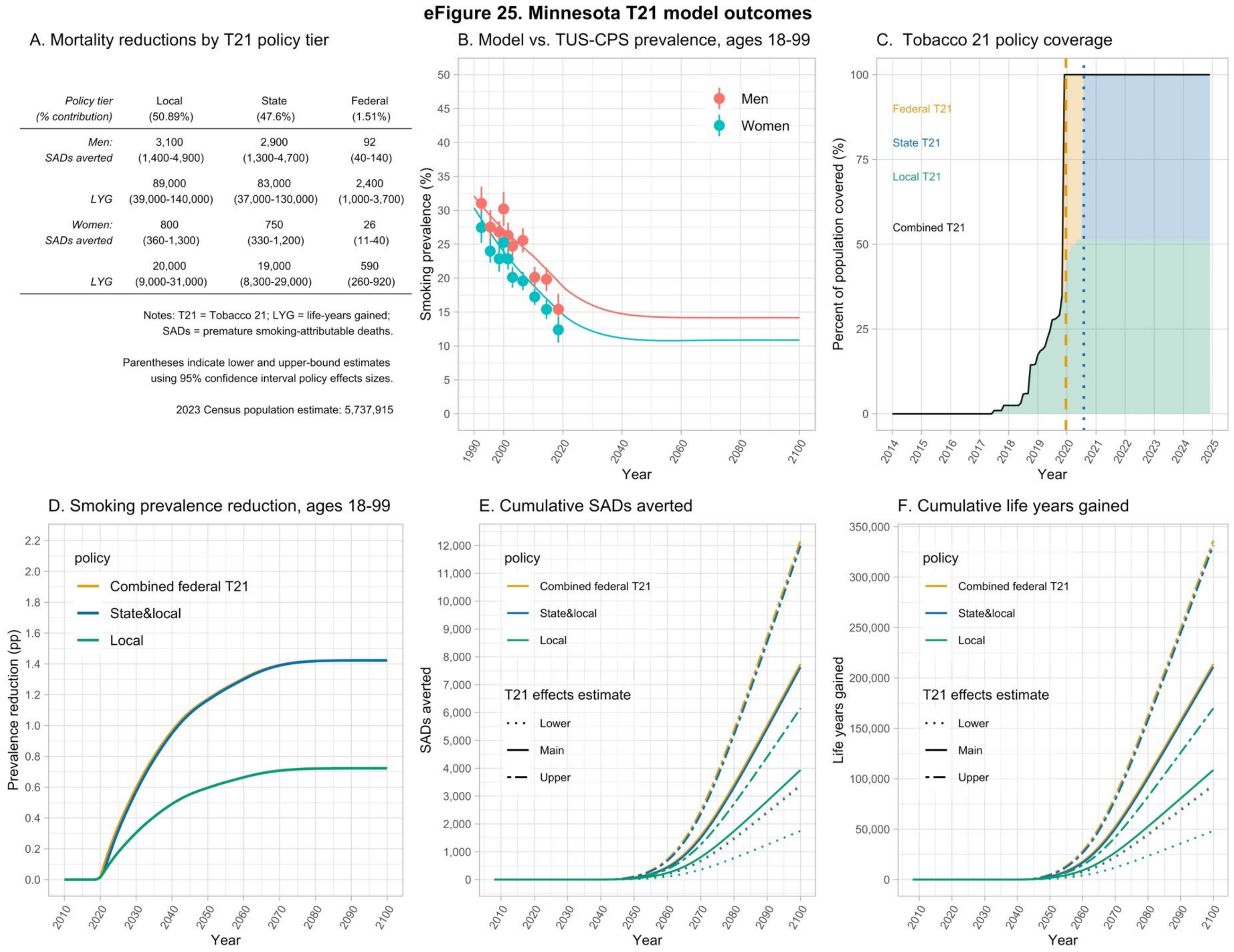

Policy tier (% contribution)	Local (50.89%)	State (47.6%)	Federal (1.51%)
Men: SADs averted	3,100 (1,400-4,900)	2,900 (1,300-4,700)	92 (40-140)
LYG	89,000 (39,000-140,000)	83,000 (37,000-130,000)	2,400 (1,000-3,700)
Women: SADs averted	800 (360-1,300)	750 (330-1,200)	26 (11-40)
LYG	20,000 (9,000-31,000)	19,000 (8,300-29,000)	590 (260-920)

Notes: T21 = Tobacco 21; LYG = life-years gained; SADs = premature smoking-attributable deaths.

Parentheses indicate lower and upper-bound estimates using 95% confidence interval policy effects sizes.

2023 Census population estimate: 5,737,915

© 2025 Tam J et al. JAMA Health Forum.

© 2025 Tam J et al. JAMA Health Forum.

eFigure 26. Mississippi T21 model outcomes

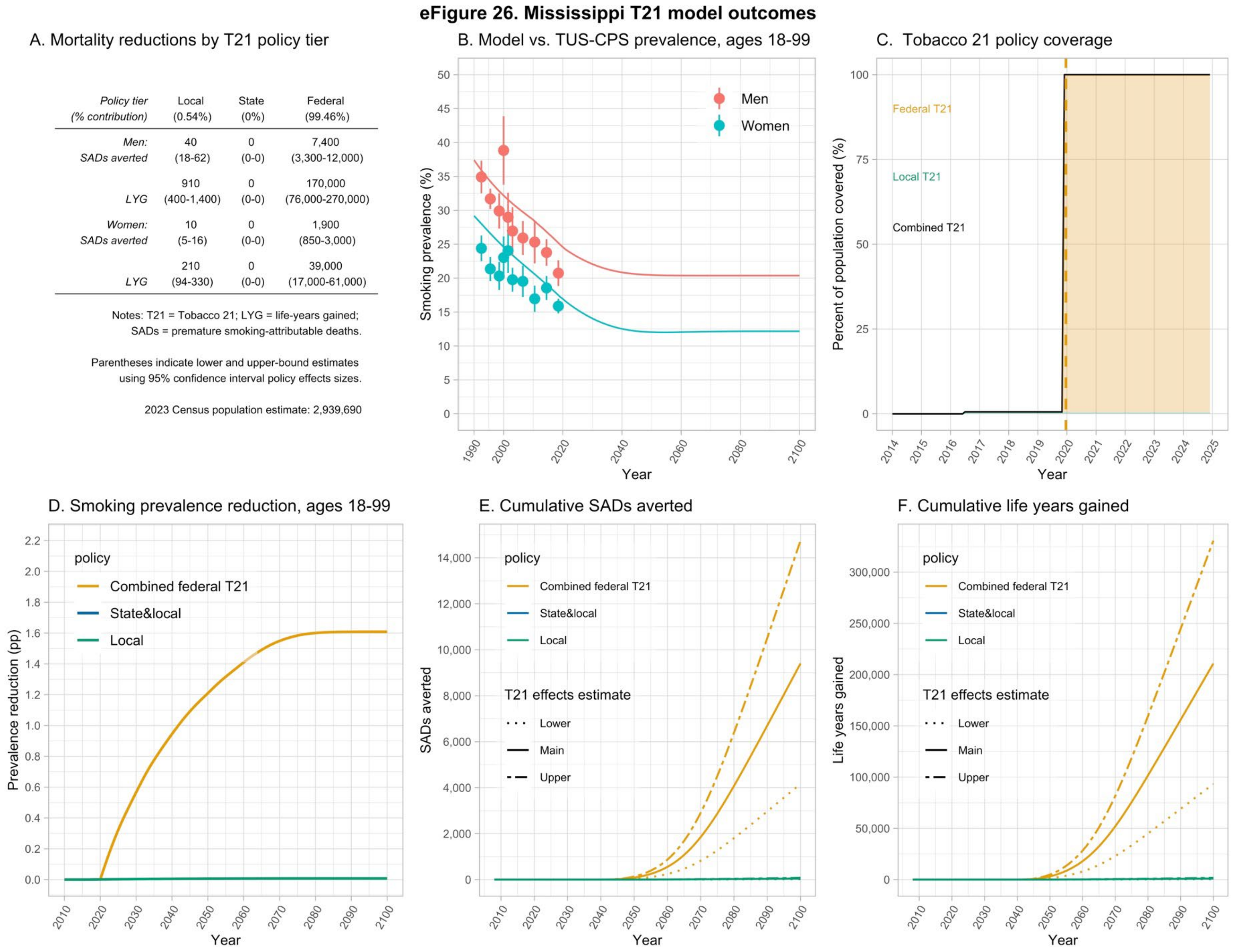

© 2025 Tam J et al. JAMA Health Forum.

© 2025 Tam J et al. JAMA Health Forum.

eFigure 27. Missouri T21 model outcomes

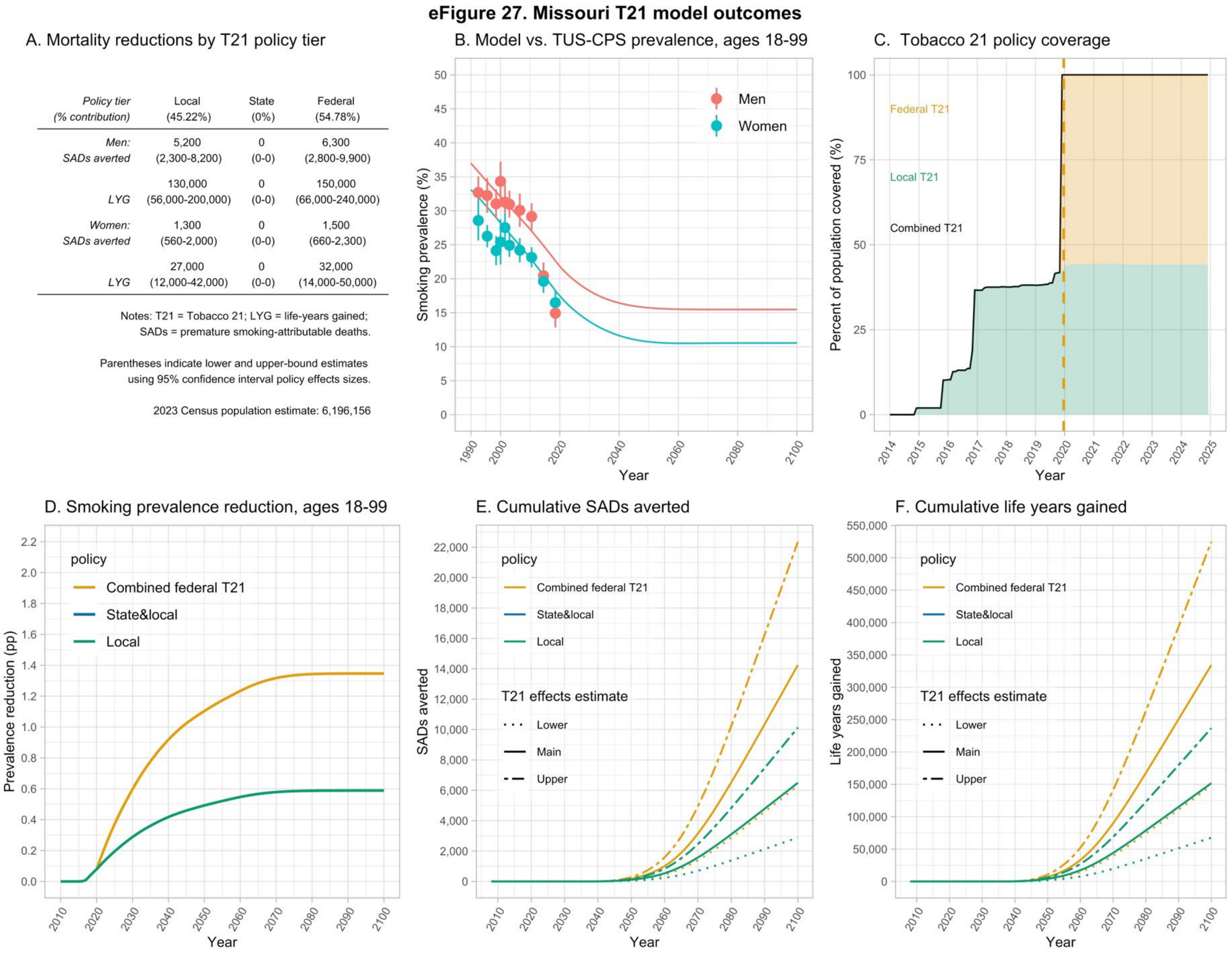

© 2025 Tam J et al. JAMA Health Forum.

© 2025 Tam J et al. JAMA Health Forum.

eFigure 28. Montana T21 model outcomes

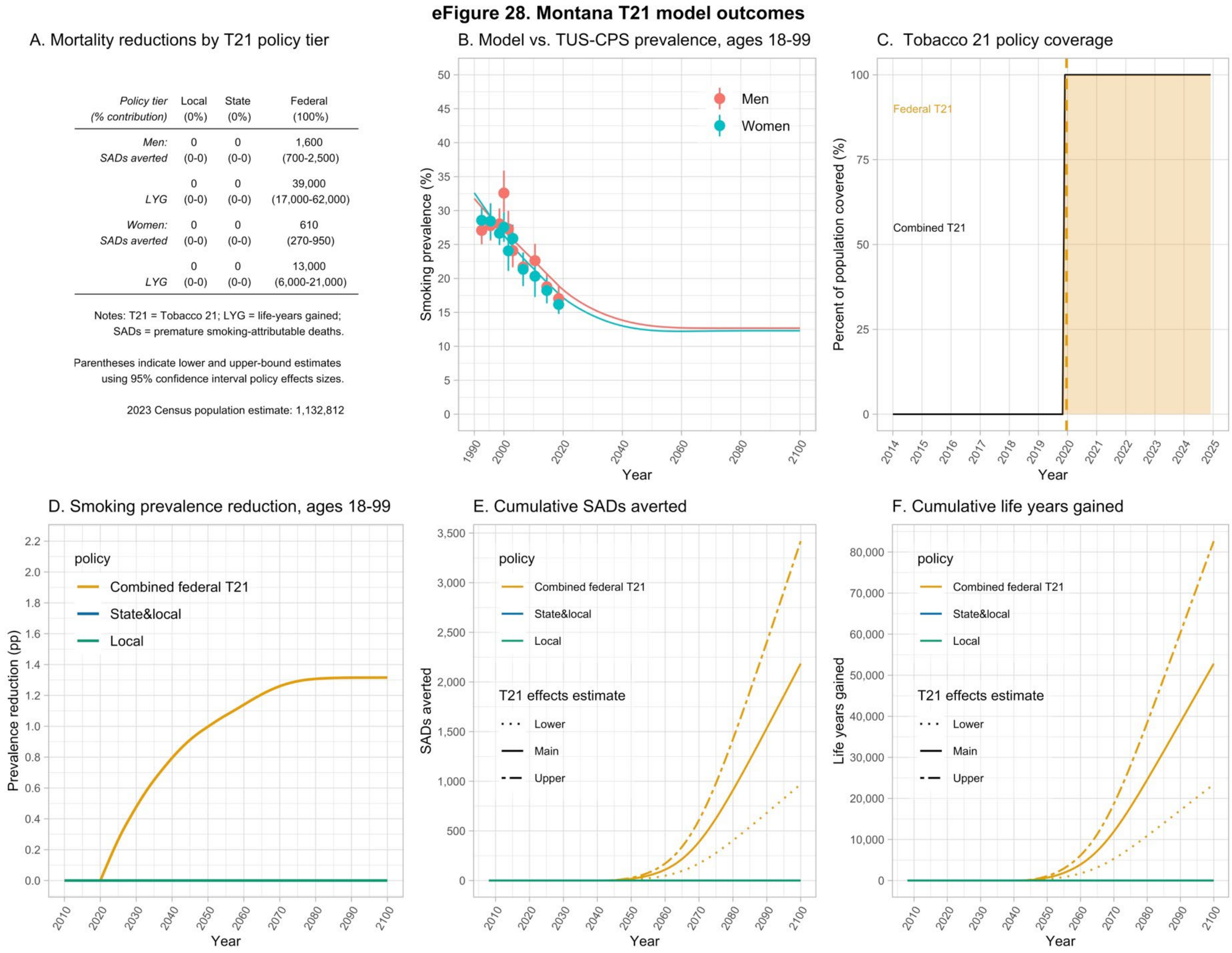

Policy tier (% contribution)	Local (0%)	State (0%)	Federal (100%)
Men: SADs averted	0 (0-0)	0 (0-0)	1,600 (700-2,500)
LYG	0 (0-0)	0 (0-0)	39,000 (17,000-62,000)
Women: SADs averted	0 (0-0)	0 (0-0)	610 (270-950)
LYG	0 (0-0)	0 (0-0)	13,000 (6,000-21,000)

Notes: T21 = Tobacco 21; LYG = life-years gained; SADs = premature smoking-attributable deaths.

Parentheses indicate lower and upper-bound estimates using 95% confidence interval policy effects sizes.

2023 Census population estimate: 1,132,812

© 2025 Tam J et al. JAMA Health Forum.

© 2025 Tam J et al. JAMA Health Forum.

eFigure 29. Nebraska T21 model outcomes

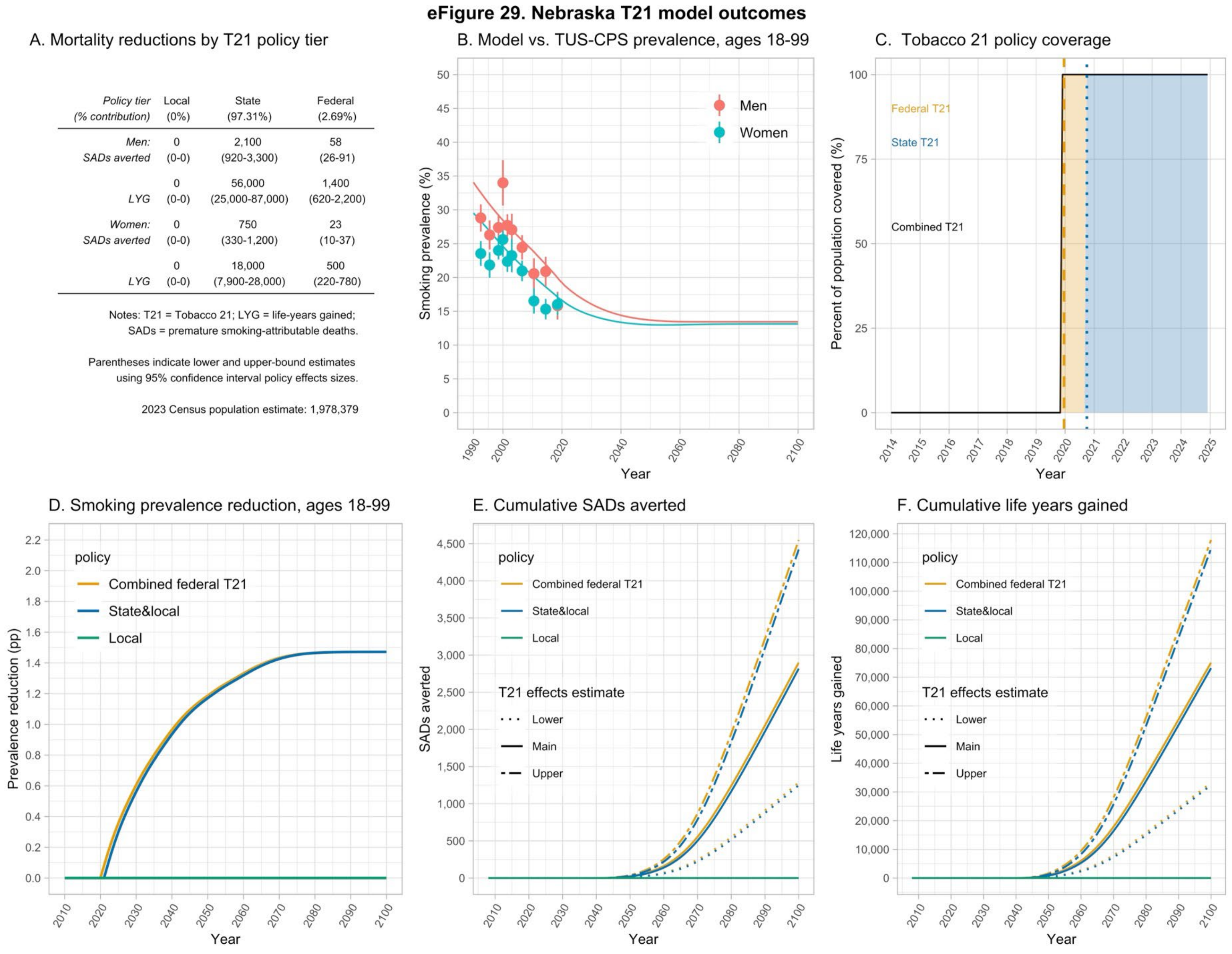

Policy tier (% contribution)	Local (0%)	State (97.31%)	Federal (2.69%)
Men: SADs averted	0 (0-0)	2,100 (920-3,300)	58 (26-91)
LYG	0 (0-0)	56,000 (25,000-87,000)	1,400 (620-2,200)
Women: SADs averted	0 (0-0)	750 (330-1,200)	23 (10-37)
LYG	0 (0-0)	18,000 (7,900-28,000)	500 (220-780)

Notes: T21 = Tobacco 21; LYG = life-years gained; SADs = premature smoking-attributable deaths.

Parentheses indicate lower and upper-bound estimates using 95% confidence interval policy effects sizes.

2023 Census population estimate: 1,978,379

© 2025 Tam J et al. JAMA Health Forum.

© 2025 Tam J et al. JAMA Health Forum.

eFigure 30. Nevada T21 model outcomes

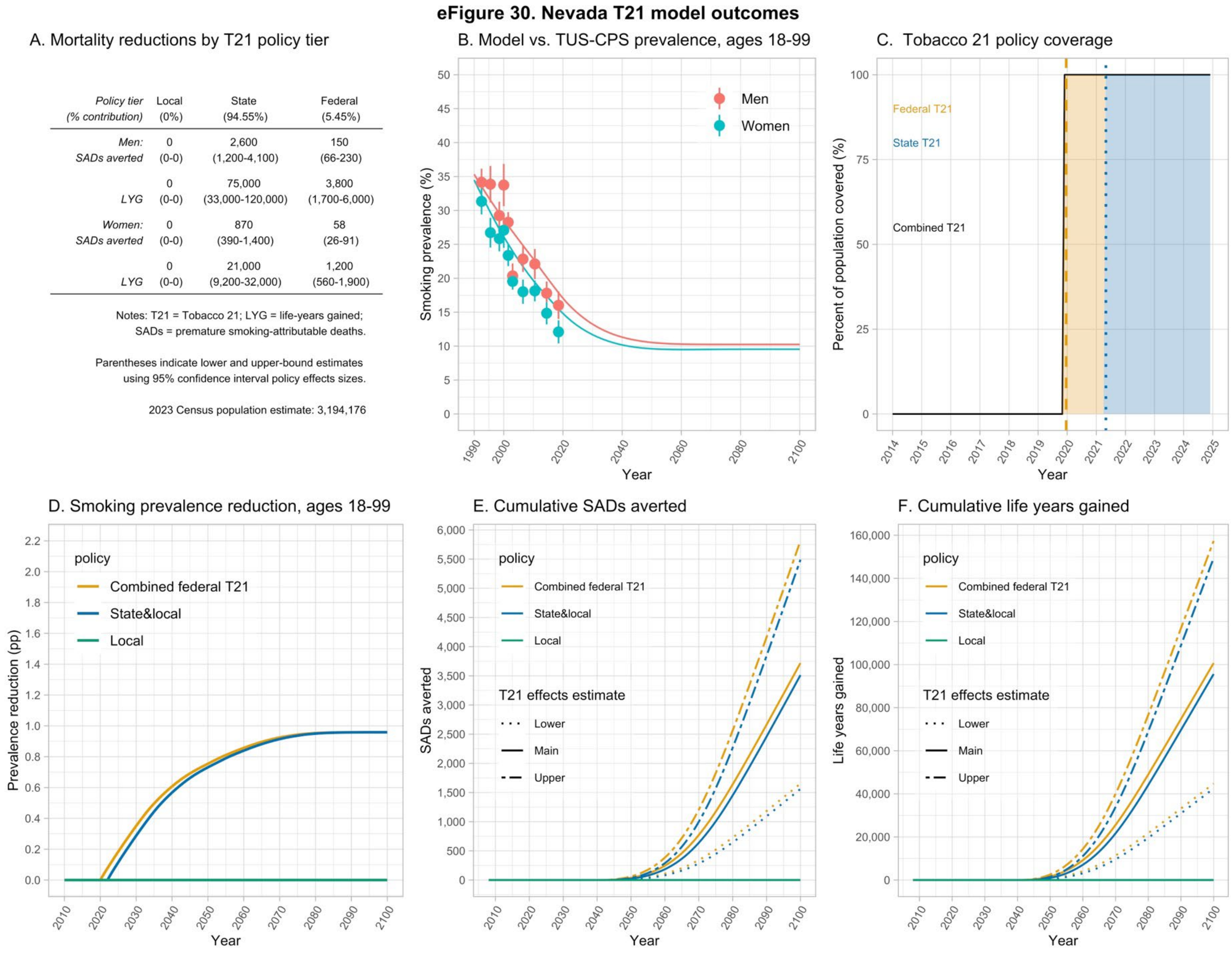

Policy tier (% contribution)	Local (0%)	State (94.55%)	Federal (5.45%)
Men: SADs averted	0 (0-0)	2,600 (1,200-4,100)	150 (66-230)
LYG	0 (0-0)	75,000 (33,000-120,000)	3,800 (1,700-6,000)
Women: SADs averted	0 (0-0)	870 (390-1,400)	58 (26-91)
LYG	0 (0-0)	21,000 (9,200-32,000)	1,200 (560-1,900)

Notes: T21 = Tobacco 21; LYG = life-years gained; SADs = premature smoking-attributable deaths.

Parentheses indicate lower and upper-bound estimates using 95% confidence interval policy effects sizes.

2023 Census population estimate: 3,194,176

© 2025 Tam J et al. JAMA Health Forum.

© 2025 Tam J et al. JAMA Health Forum.

eFigure 31. New Hampshire T21 model outcomes

eFigure 31. New Hampshire T21 model outcomes

A. Mortality reductions by T21 policy tier

Policy tier (% contribution)	Local (6.69%)	State (90.82%)	Federal (2.49%)
Men: SADs averted	140 (60-210)	1,900 (830-2,900)	52 (23-81)
LYG	3,300 (1,500-5,200)	47,000 (21,000-73,000)	1,200 (520-1,900)
Women: SADs averted	49 (22-76)	670 (300-1,100)	20 (9-31)
LYG	1,100 (500-1,800)	16,000 (7,000-25,000)	420 (190-660)

Notes: T21 = Tobacco 21; LYG = life-years gained; SADs = premature smoking-attributable deaths.

Parentheses indicate lower and upper-bound estimates using 95% confidence interval policy effects sizes.

2023 Census population estimate: 1,402,054

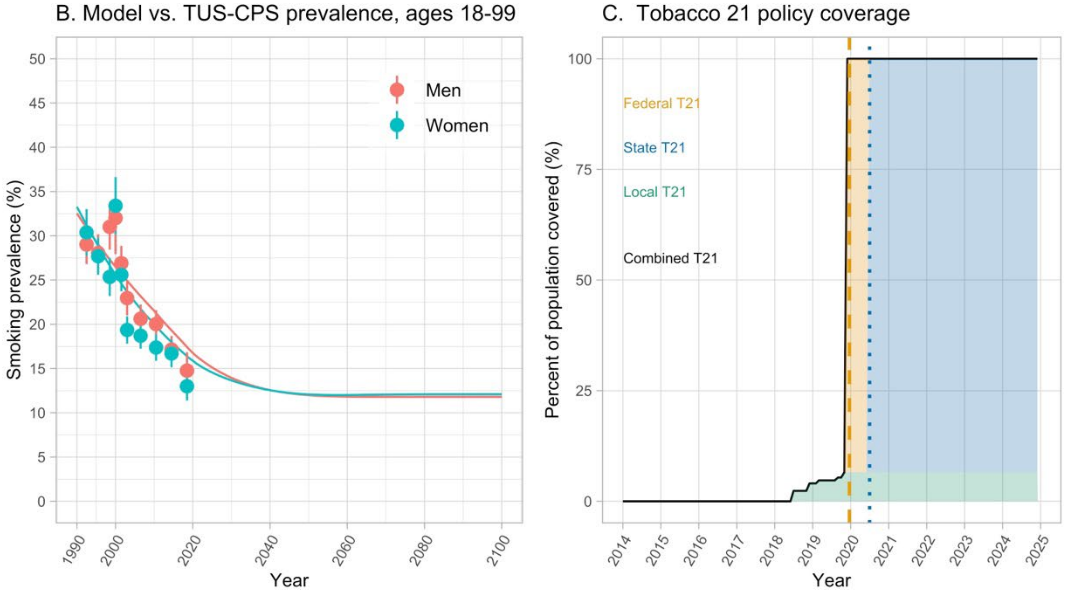

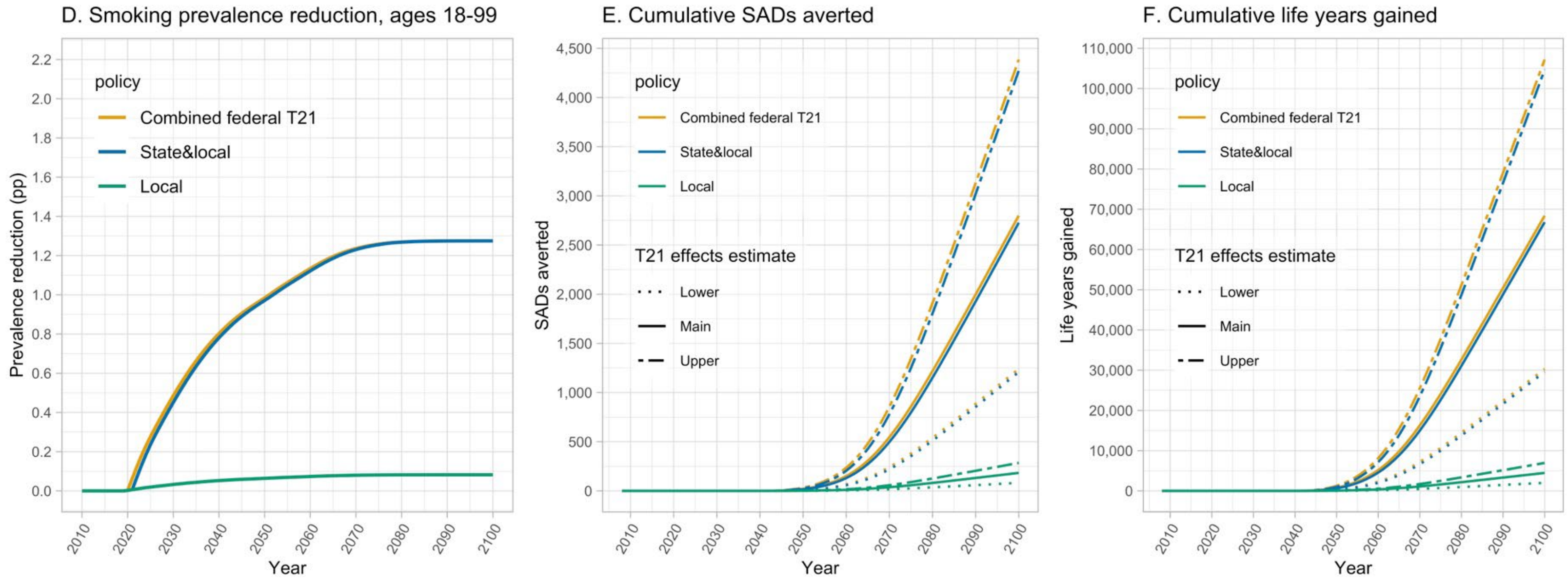

© 2025 Tam J et al. JAMA Health Forum.

© 2025 Tam J et al. JAMA Health Forum.

eFigure 32. New Jersey T21 model outcomes

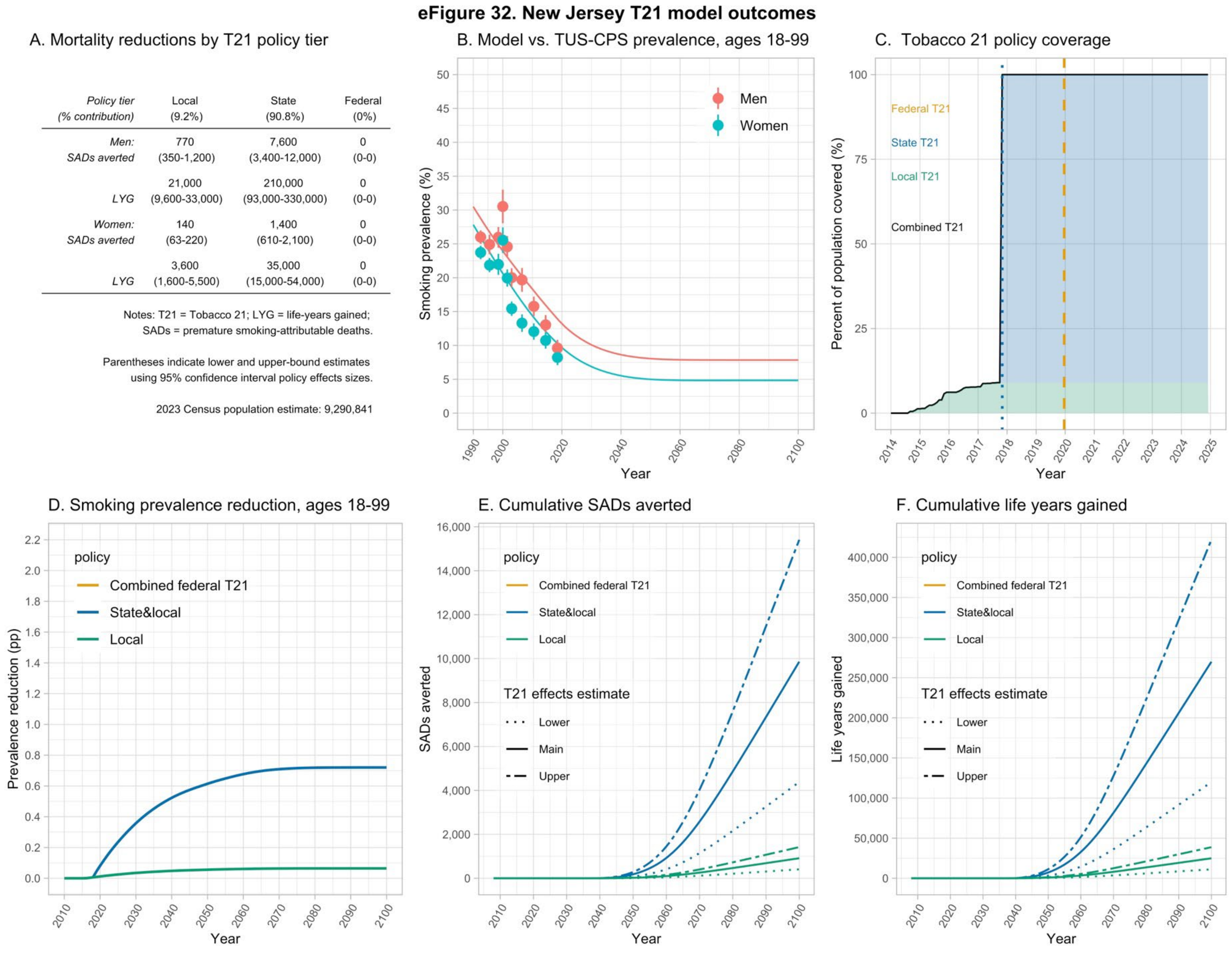

Policy tier (% contribution)	Local (9.2%)	State (90.8%)	Federal (0%)
Men: SADs averted	770 (350-1,200)	7,600 (3,400-12,000)	0 (0-0)
LYG	21,000 (9,600-33,000)	210,000 (93,000-330,000)	0 (0-0)
Women: SADs averted	140 (63-220)	1,400 (610-2,100)	0 (0-0)
LYG	3,600 (1,600-5,500)	35,000 (15,000-54,000)	0 (0-0)

Notes: T21 = Tobacco 21; LYG = life-years gained; SADs = premature smoking-attributable deaths.

Parentheses indicate lower and upper-bound estimates using 95% confidence interval policy effects sizes.

2023 Census population estimate: 9,290,841

© 2025 Tam J et al. JAMA Health Forum.

© 2025 Tam J et al. JAMA Health Forum.

eFigure 33. New Mexico T21 model outcomes

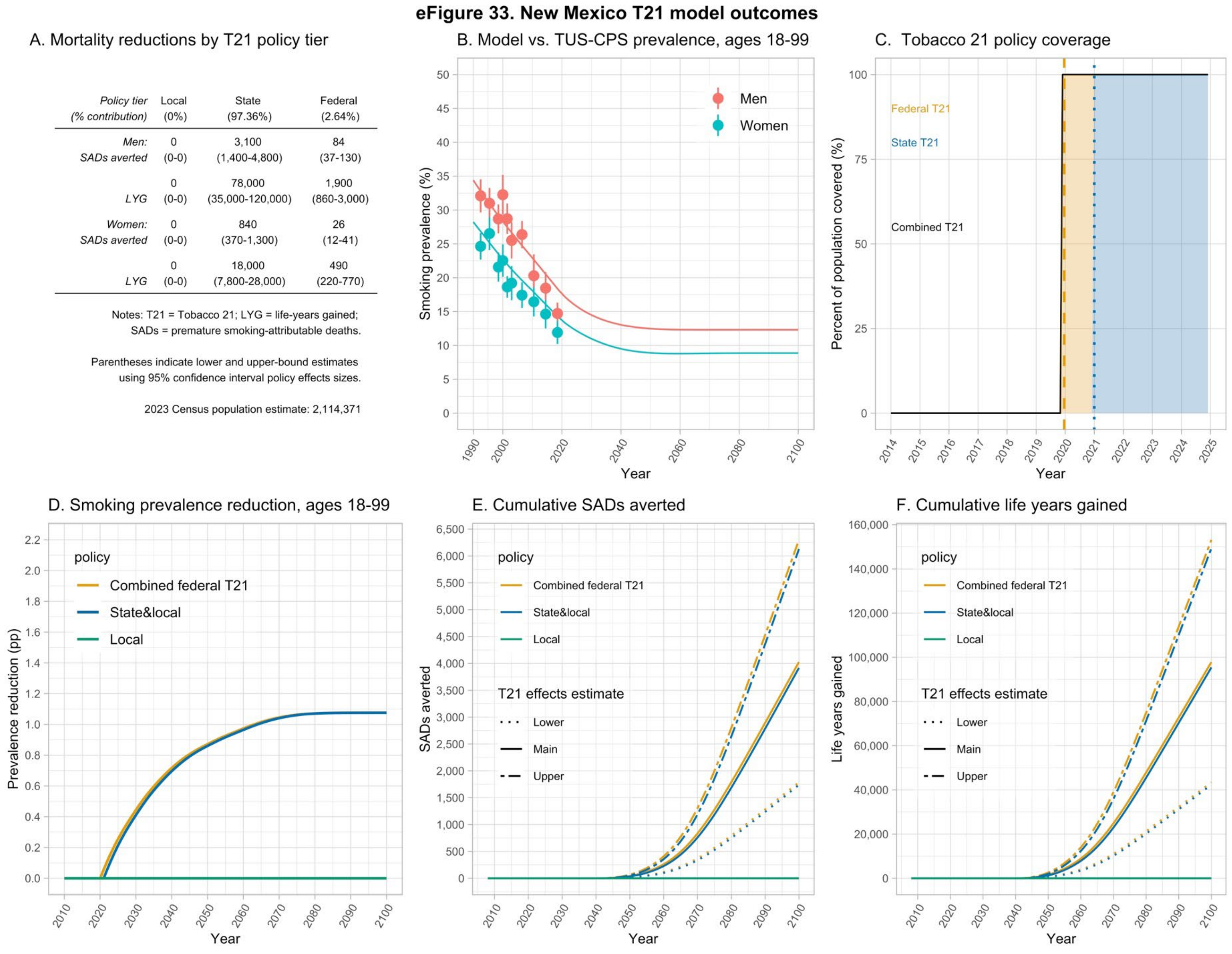

Policy tier (% contribution)	Local (0%)	State (97.36%)	Federal (2.64%)
Men: SADs averted	0 (0-0)	3,100 (1,400-4,800)	84 (37-130)
LYG	0 (0-0)	78,000 (35,000-120,000)	1,900 (860-3,000)
Women: SADs averted	0 (0-0)	840 (370-1,300)	26 (12-41)
LYG	0 (0-0)	18,000 (7,800-28,000)	490 (220-770)

Notes: T21 = Tobacco 21; LYG = life-years gained; SADs = premature smoking-attributable deaths.

Parentheses indicate lower and upper-bound estimates using 95% confidence interval policy effects sizes.

2023 Census population estimate: 2,114,371

© 2025 Tam J et al. JAMA Health Forum.

© 2025 Tam J et al. JAMA Health Forum.

eFigure 34. New York T21 model outcomes

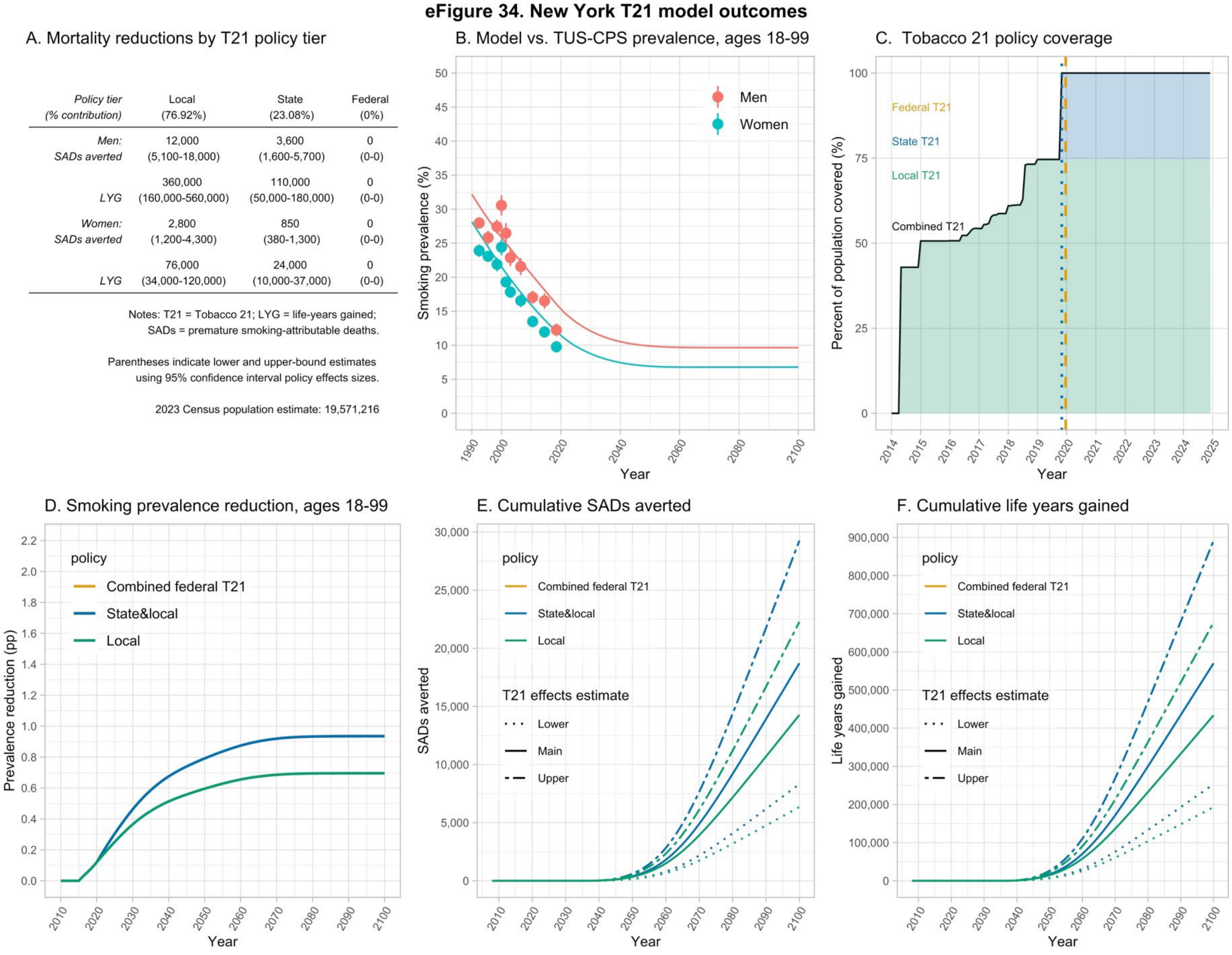

Policy tier (% contribution)	Local (76.92%)	State (23.08%)	Federal (0%)
Men: SADs averted	12,000 (5,100-18,000)	3,600 (1,600-5,700)	0 (0-0)
LYG	360,000 (160,000-560,000)	110,000 (50,000-180,000)	0 (0-0)
Women: SADs averted	2,800 (1,200-4,300)	850 (380-1,300)	0 (0-0)
LYG	76,000 (34,000-120,000)	24,000 (10,000-37,000)	0 (0-0)

Notes: T21 = Tobacco 21; LYG = life-years gained; SADs = premature smoking-attributable deaths.

Parentheses indicate lower and upper-bound estimates using 95% confidence interval policy effects sizes.

2023 Census population estimate: 19,571,216

© 2025 Tam J et al. JAMA Health Forum.

© 2025 Tam J et al. JAMA Health Forum.

eFigure 35. North Carolina T21 model outcomes

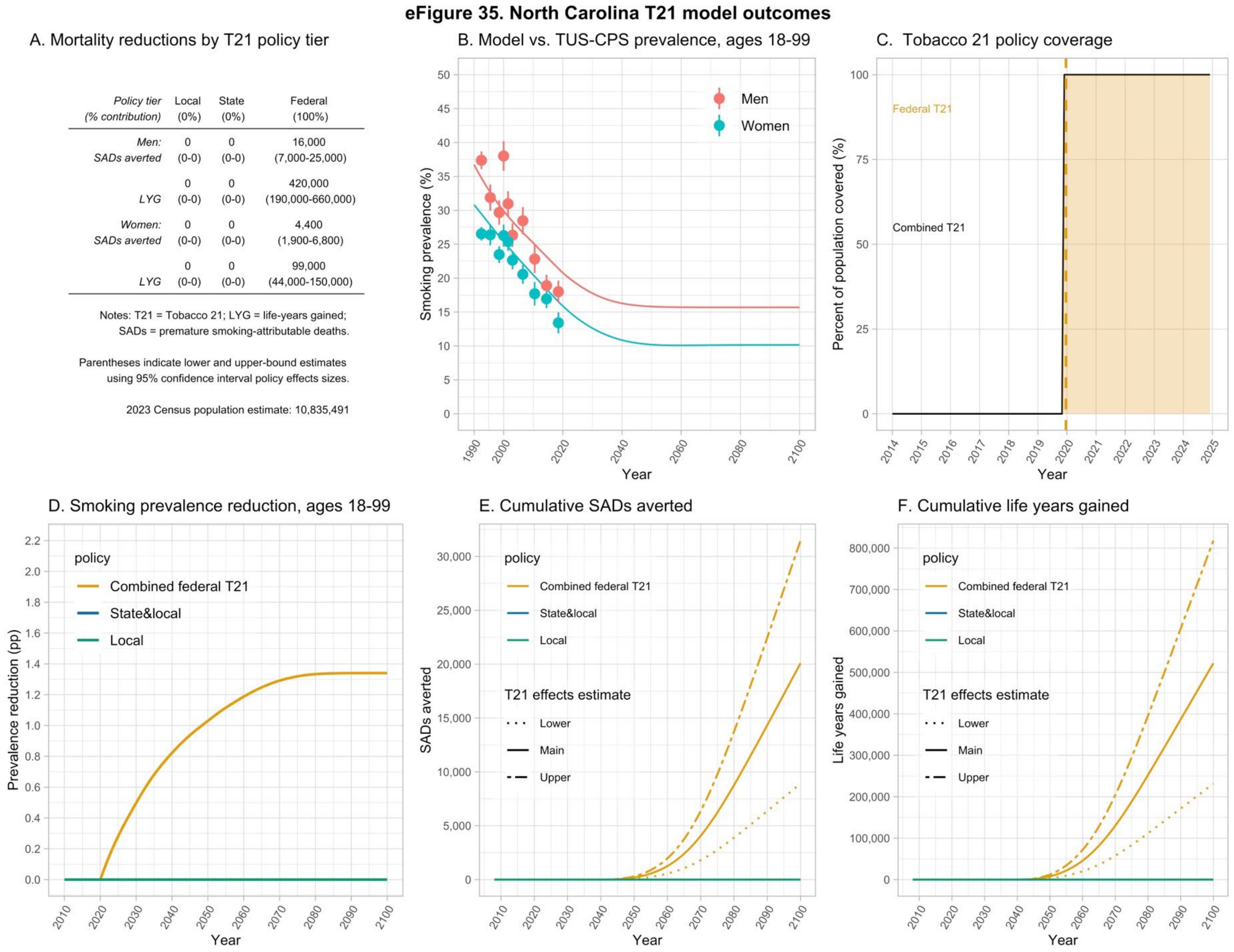

A. Mortality reductions by T21 policy tier

Policy tier (% contribution)	Local (0%)	State (0%)	Federal (100%)
Men: SADs averted	0 (0-0)	0 (0-0)	16,000 (7,000-25,000)
LYG	0 (0-0)	0 (0-0)	420,000 (190,000-660,000)
Women: SADs averted	0 (0-0)	0 (0-0)	4,400 (1,900-6,800)
LYG	0 (0-0)	0 (0-0)	99,000 (44,000-150,000)

Notes: T21 = Tobacco 21; LYG = life-years gained; SADs = premature smoking-attributable deaths.

Parentheses indicate lower and upper-bound estimates using 95% confidence interval policy effects sizes.

2023 Census population estimate: 10,835,491

© 2025 Tam J et al. JAMA Health Forum.

© 2025 Tam J et al. JAMA Health Forum.

eFigure 36. North Dakota T21 model outcomes

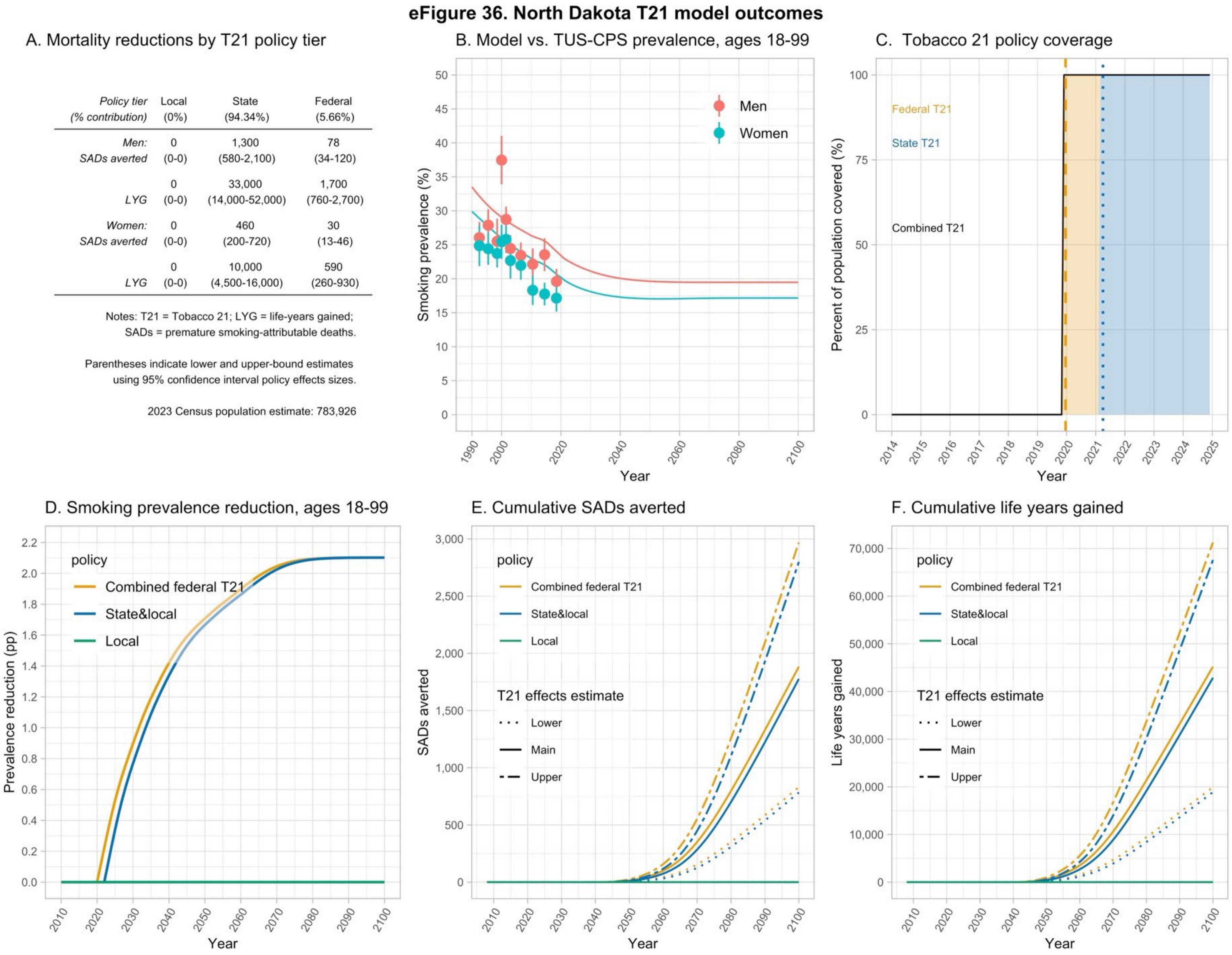

© 2025 Tam J et al. JAMA Health Forum.

© 2025 Tam J et al. JAMA Health Forum.

eFigure 37. Ohio T21 model outcomes

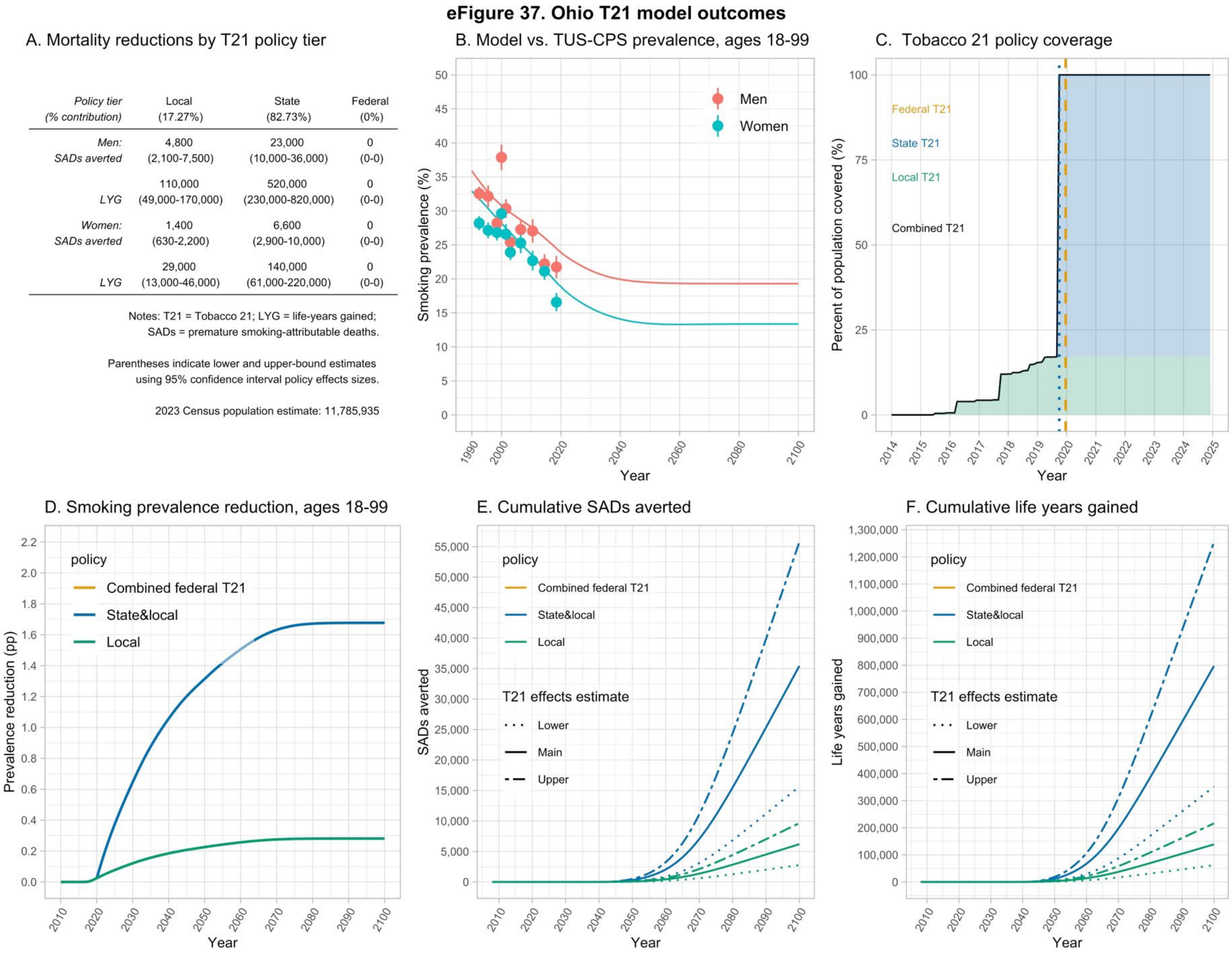

Policy tier (% contribution)	Local (17.27%)	State (82.73%)	Federal (0%)
Men: SADs averted	4,800 (2,100-7,500)	23,000 (10,000-36,000)	0 (0-0)
LYG	110,000 (49,000-170,000)	520,000 (230,000-820,000)	0 (0-0)
Women: SADs averted	1,400 (630-2,200)	6,600 (2,900-10,000)	0 (0-0)
LYG	29,000 (13,000-46,000)	140,000 (61,000-220,000)	0 (0-0)

Notes: T21 = Tobacco 21; LYG = life-years gained; SADs = premature smoking-attributable deaths.

Parentheses indicate lower and upper-bound estimates using 95% confidence interval policy effects sizes.

2023 Census population estimate: 11,785,935

© 2025 Tam J et al. JAMA Health Forum.

© 2025 Tam J et al. JAMA Health Forum.

eFigure 38. Oklahoma T21 model outcomes

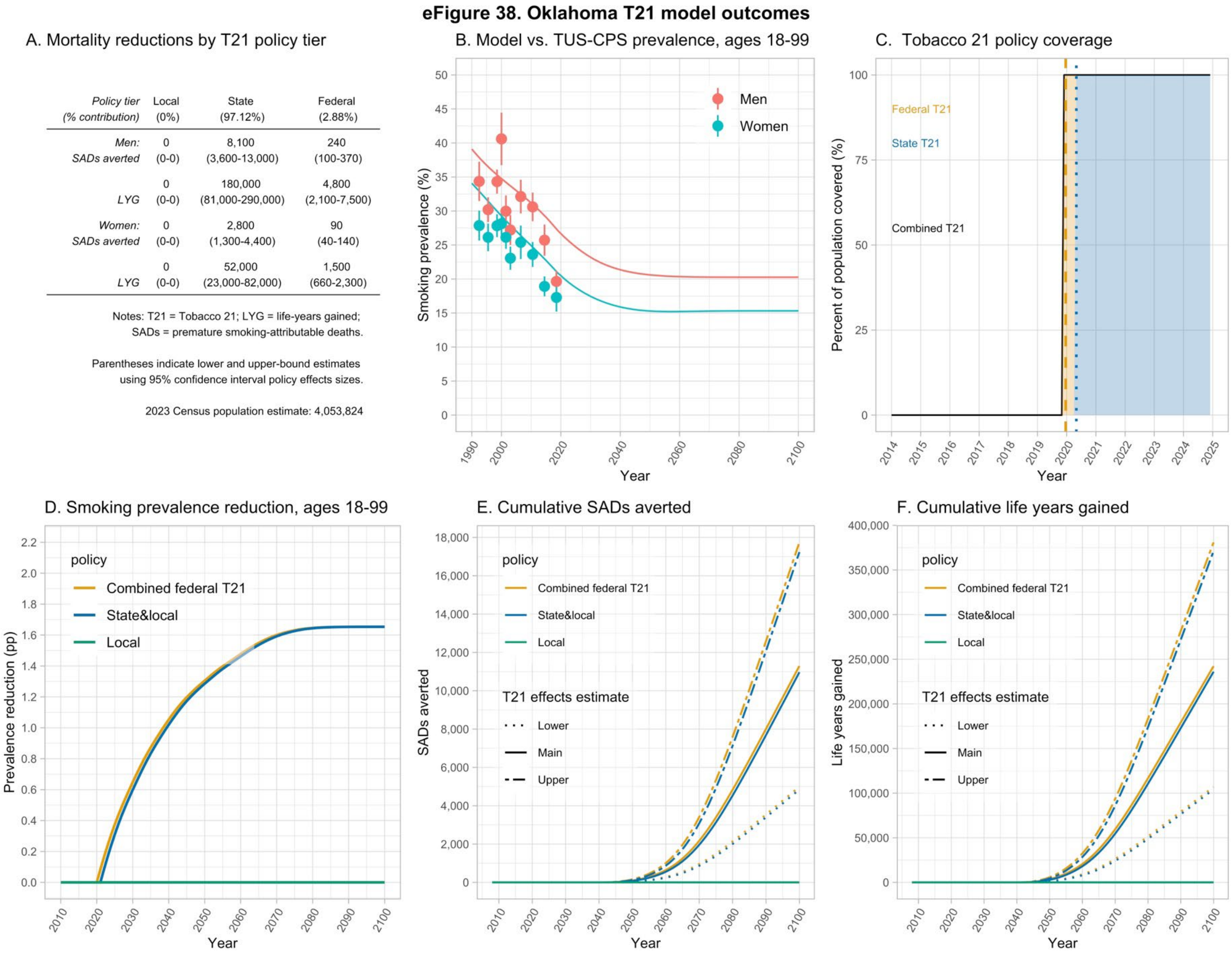

© 2025 Tam J et al. JAMA Health Forum.

© 2025 Tam J et al. JAMA Health Forum.

eFigure 39. Oregon T21 model outcomes

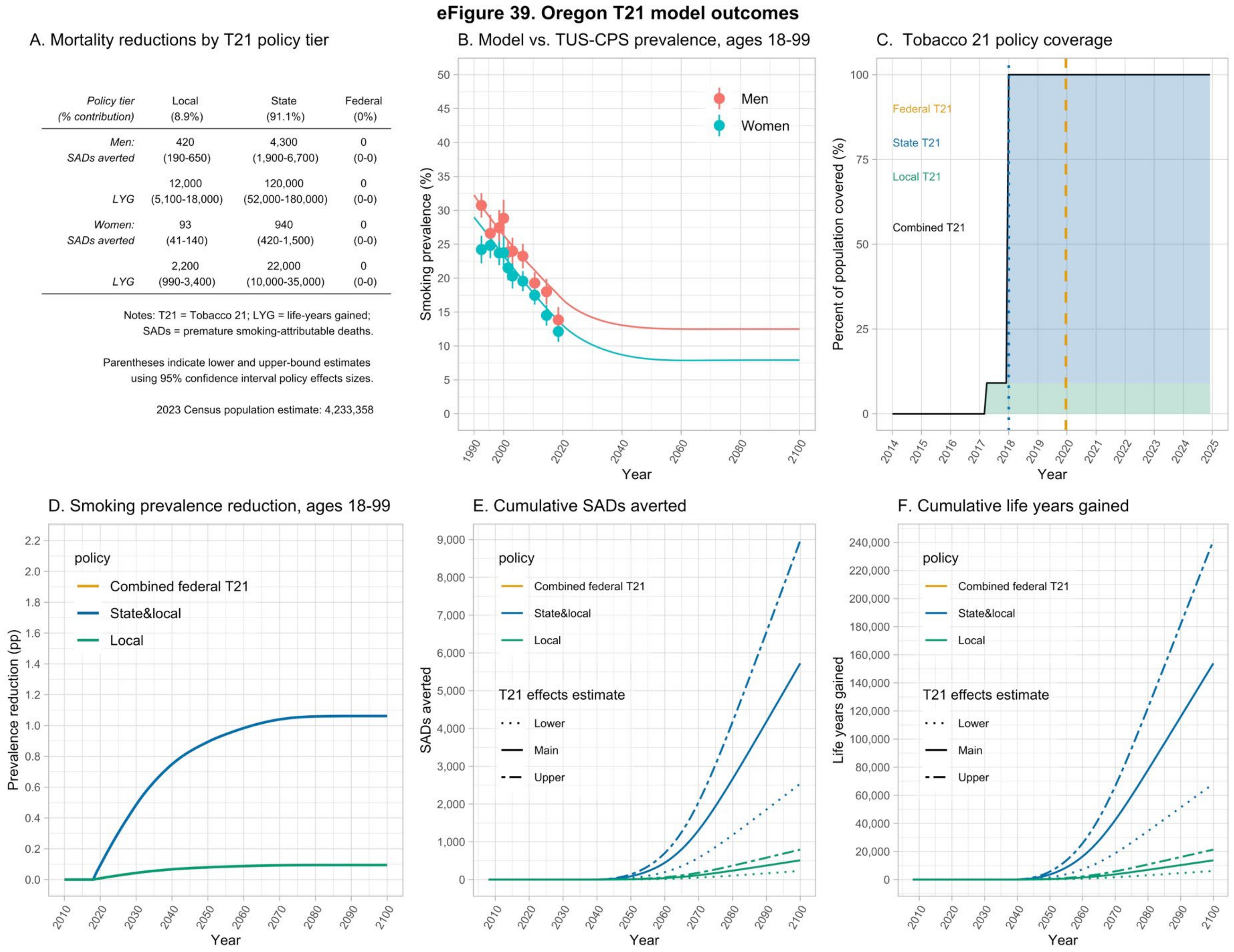

© 2025 Tam J et al. JAMA Health Forum.

© 2025 Tam J et al. JAMA Health Forum.

eFigure 40. Pennsylvania T21 model outcomes

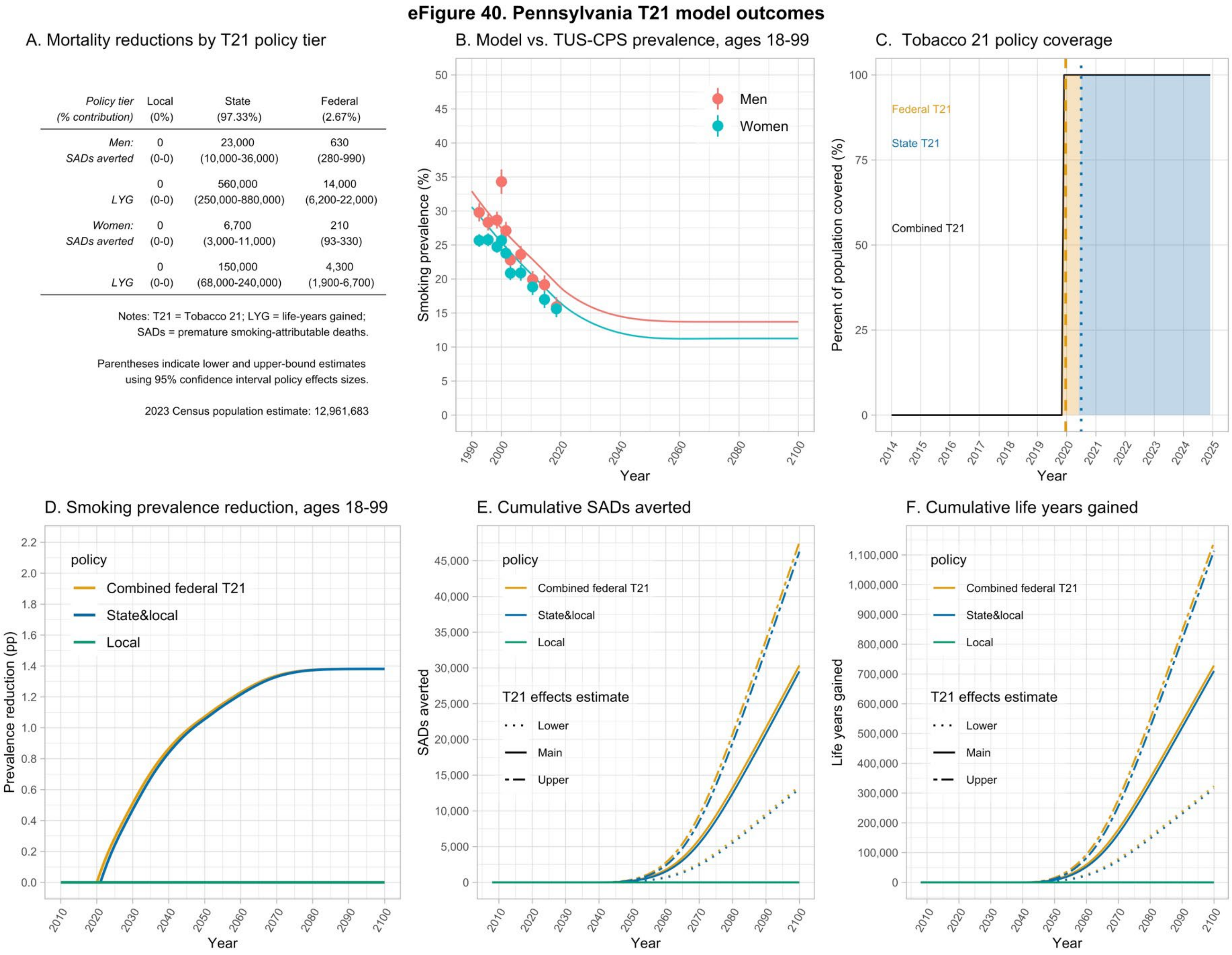

© 2025 Tam J et al. JAMA Health Forum.

© 2025 Tam J et al. JAMA Health Forum.

eFigure 41. Rhode Island T21 model outcomes

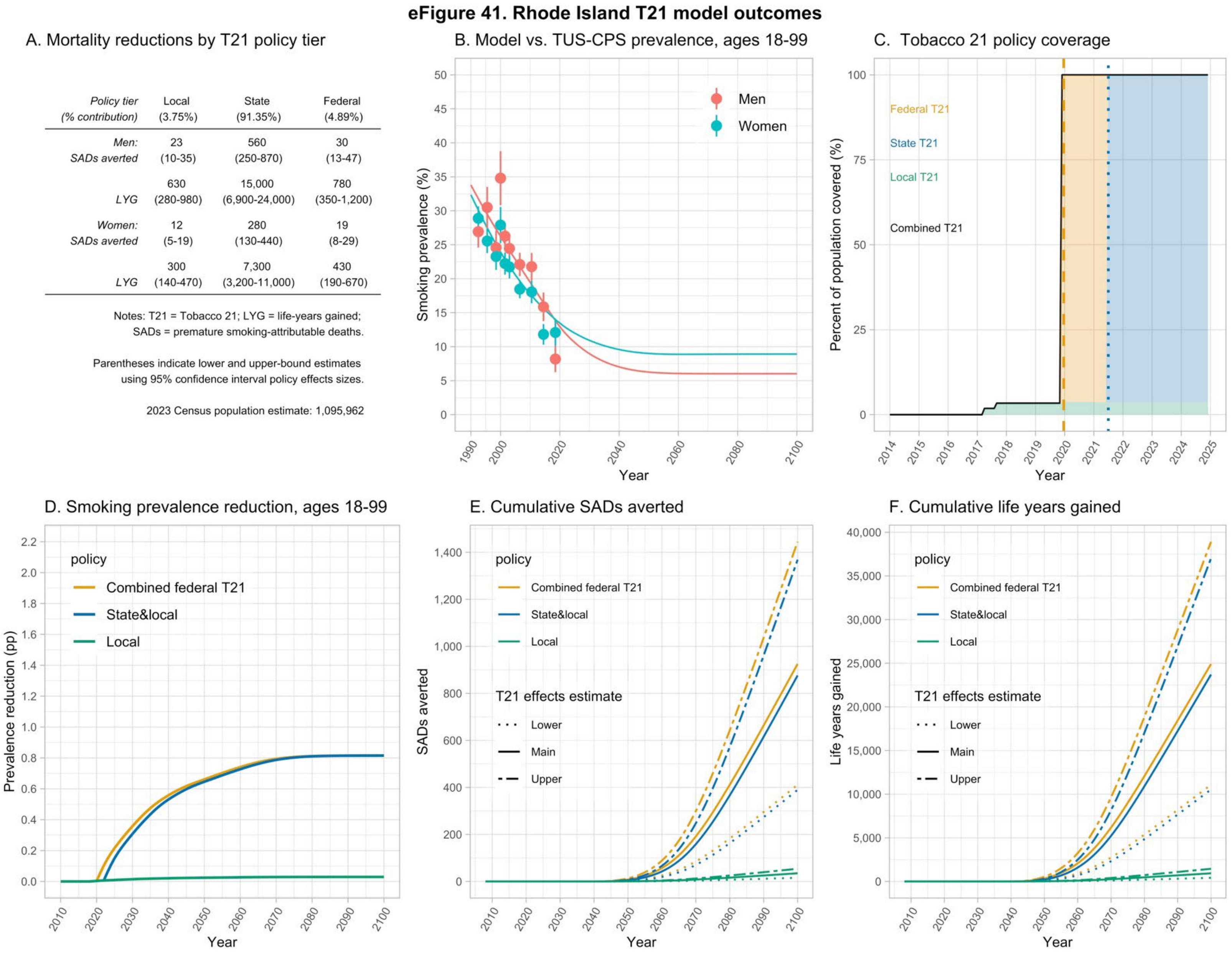

© 2025 Tam J et al. JAMA Health Forum.

© 2025 Tam J et al. JAMA Health Forum.

eFigure 42. South Carolina T21 model outcomes

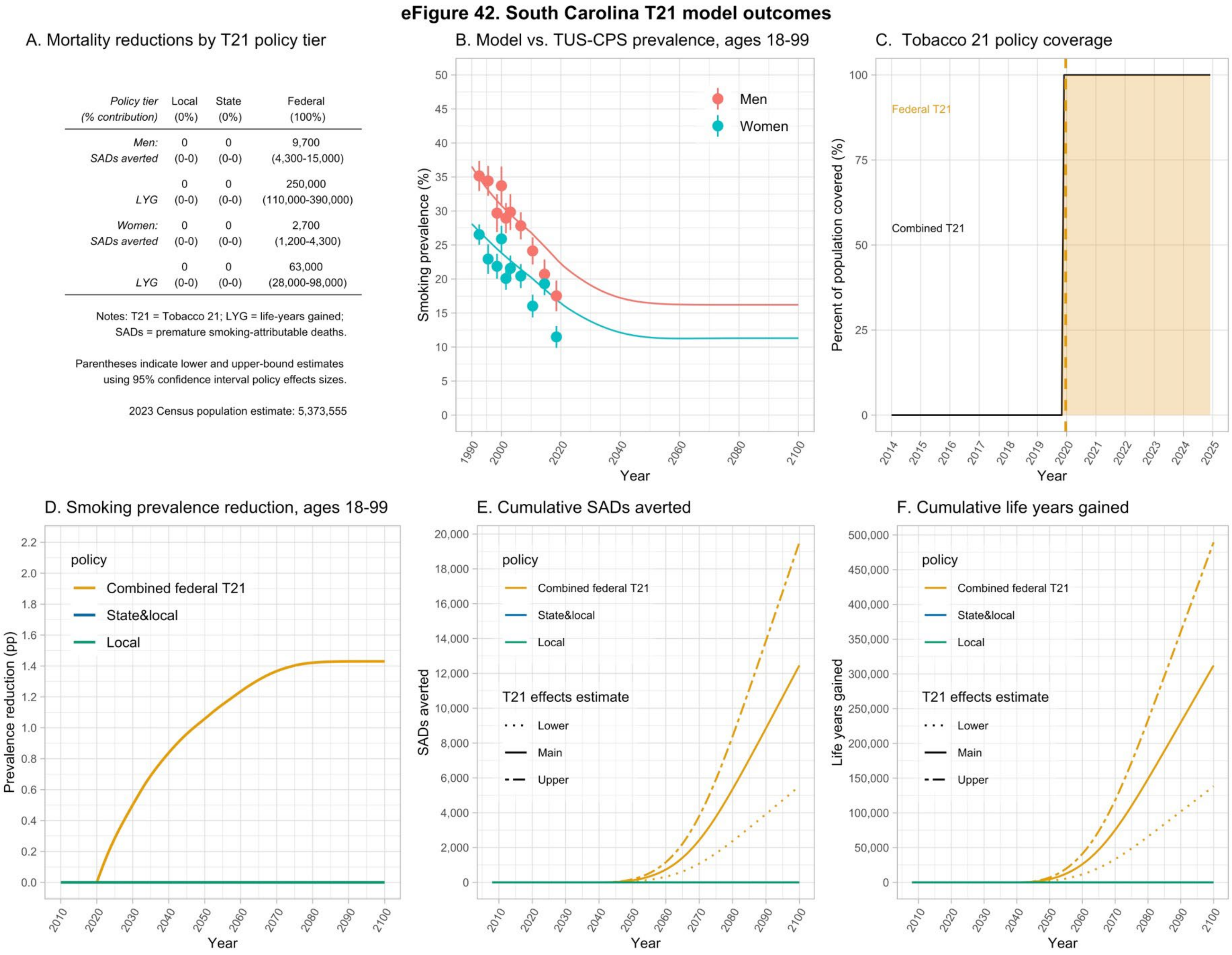

© 2025 Tam J et al. JAMA Health Forum.

© 2025 Tam J et al. JAMA Health Forum.

eFigure 43. South Dakota T21 model outcomes

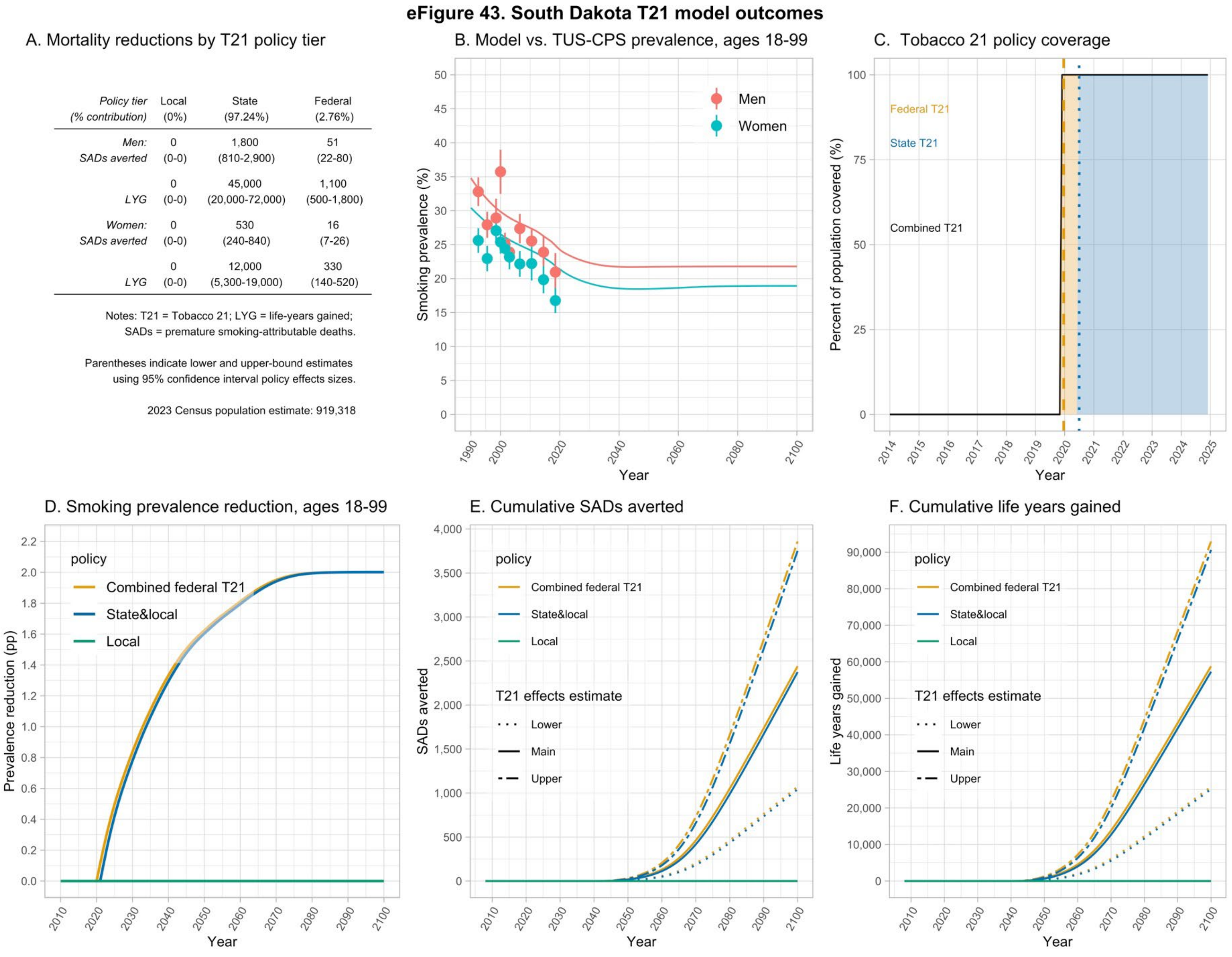

© 2025 Tam J et al. JAMA Health Forum.

© 2025 Tam J et al. JAMA Health Forum.

eFigure 44. Tennessee T21 model outcomes

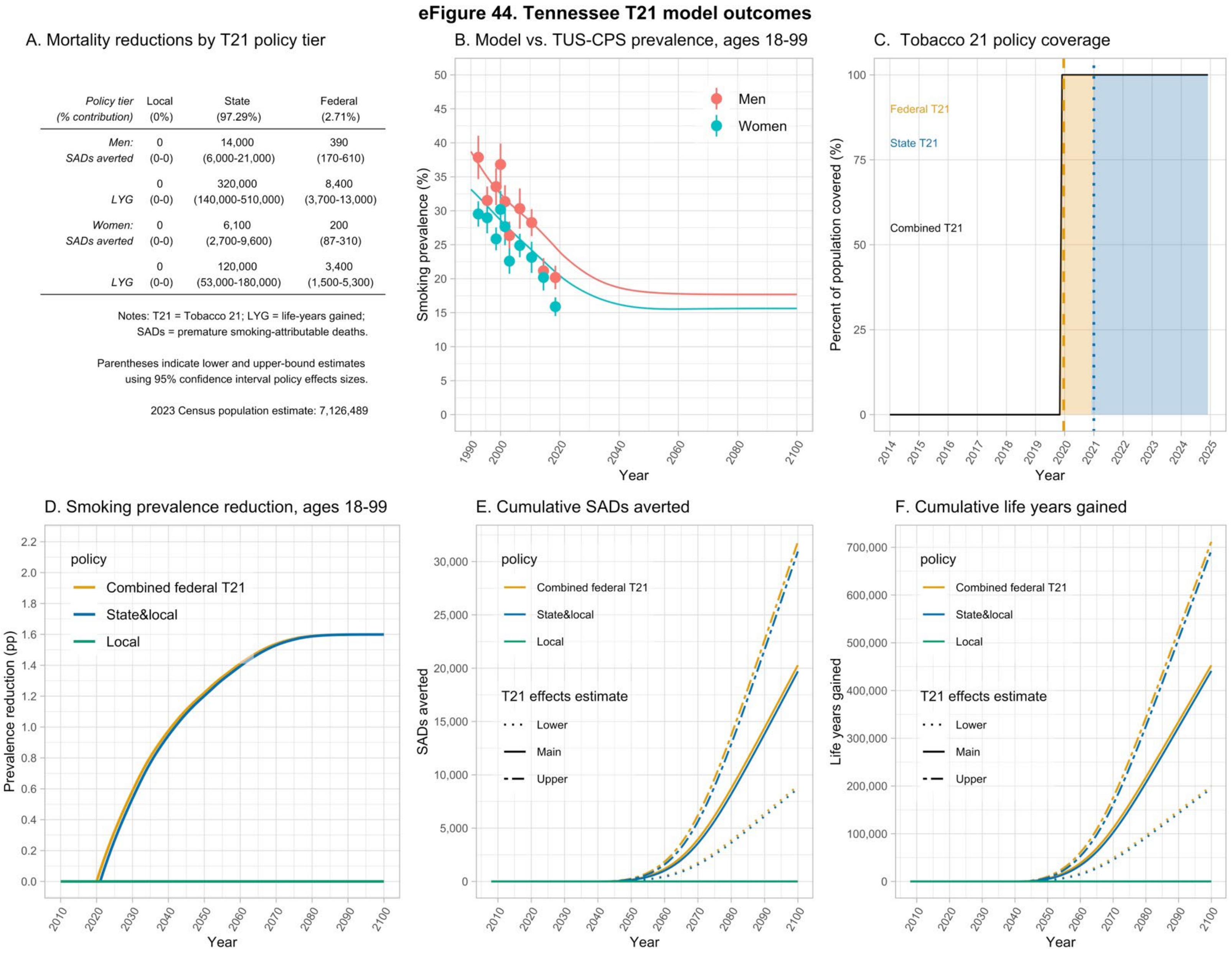

Policy tier (% contribution)	Local (0%)	State (97.29%)	Federal (2.71%)
Men: SADs averted	0 (0-0)	14,000 (6,000-21,000)	390 (170-610)
LYG	0 (0-0)	320,000 (140,000-510,000)	8,400 (3,700-13,000)
Women: SADs averted	0 (0-0)	6,100 (2,700-9,600)	200 (87-310)
LYG	0 (0-0)	120,000 (53,000-180,000)	3,400 (1,500-5,300)

Notes: T21 = Tobacco 21; LYG = life-years gained; SADs = premature smoking-attributable deaths.

Parentheses indicate lower and upper-bound estimates using 95% confidence interval policy effects sizes.

2023 Census population estimate: 7,126,489

© 2025 Tam J et al. JAMA Health Forum.

© 2025 Tam J et al. JAMA Health Forum.

eFigure 45. Texas T21 model outcomes

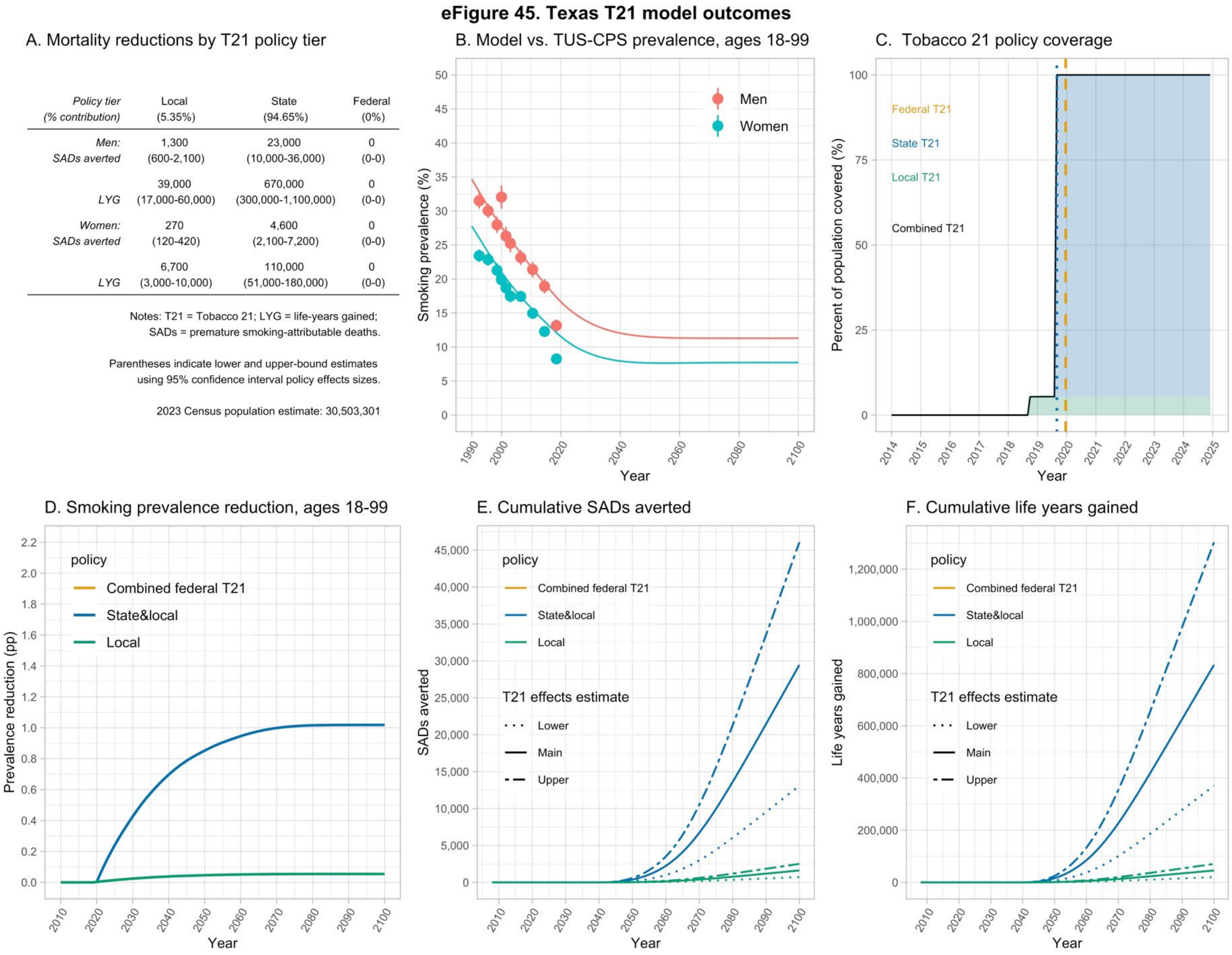

© 2025 Tam J et al. JAMA Health Forum.

© 2025 Tam J et al. JAMA Health Forum.

eFigure 46. Utah T21 model outcomes

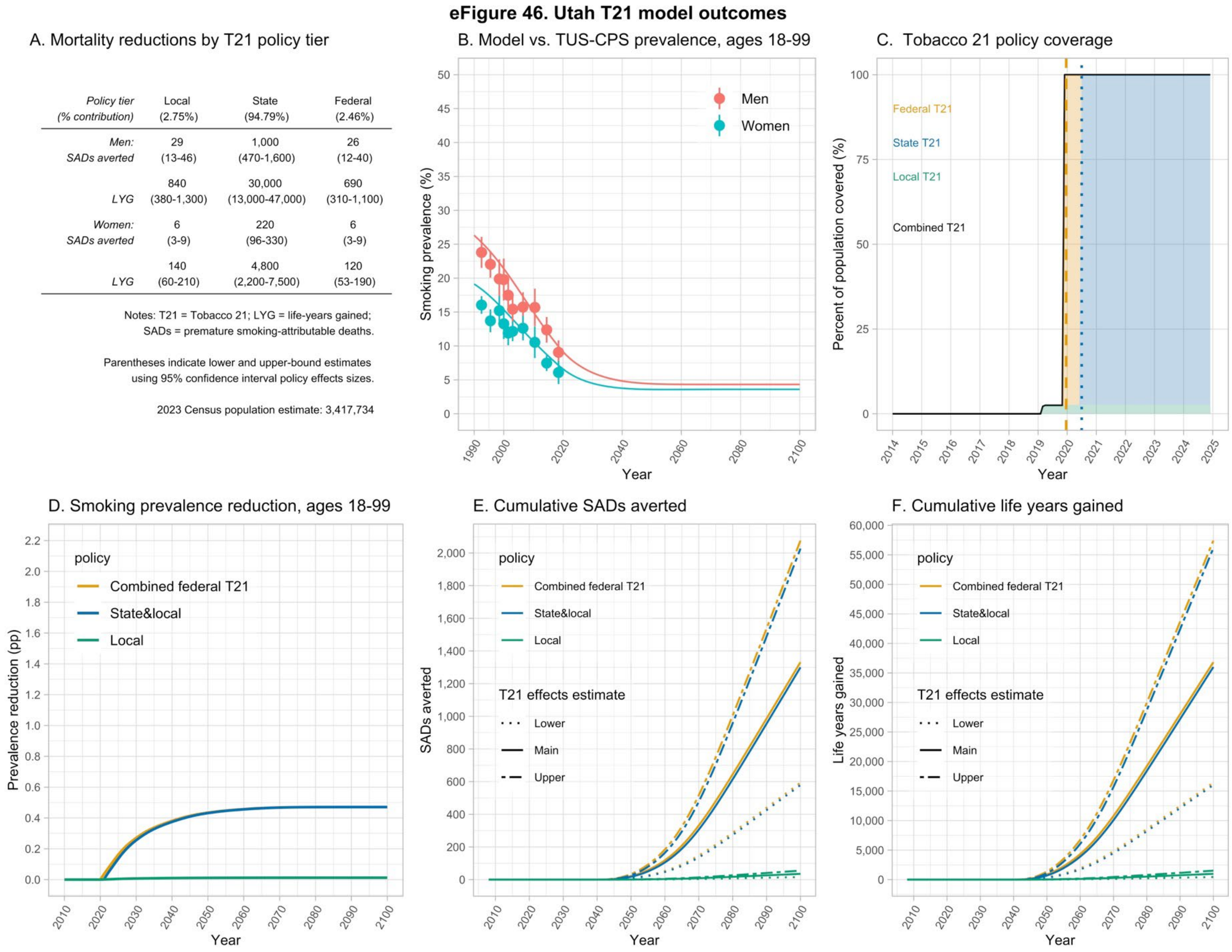

© 2025 Tam J et al. JAMA Health Forum.

© 2025 Tam J et al. JAMA Health Forum.

eFigure 47. Vermont T21 model outcomes

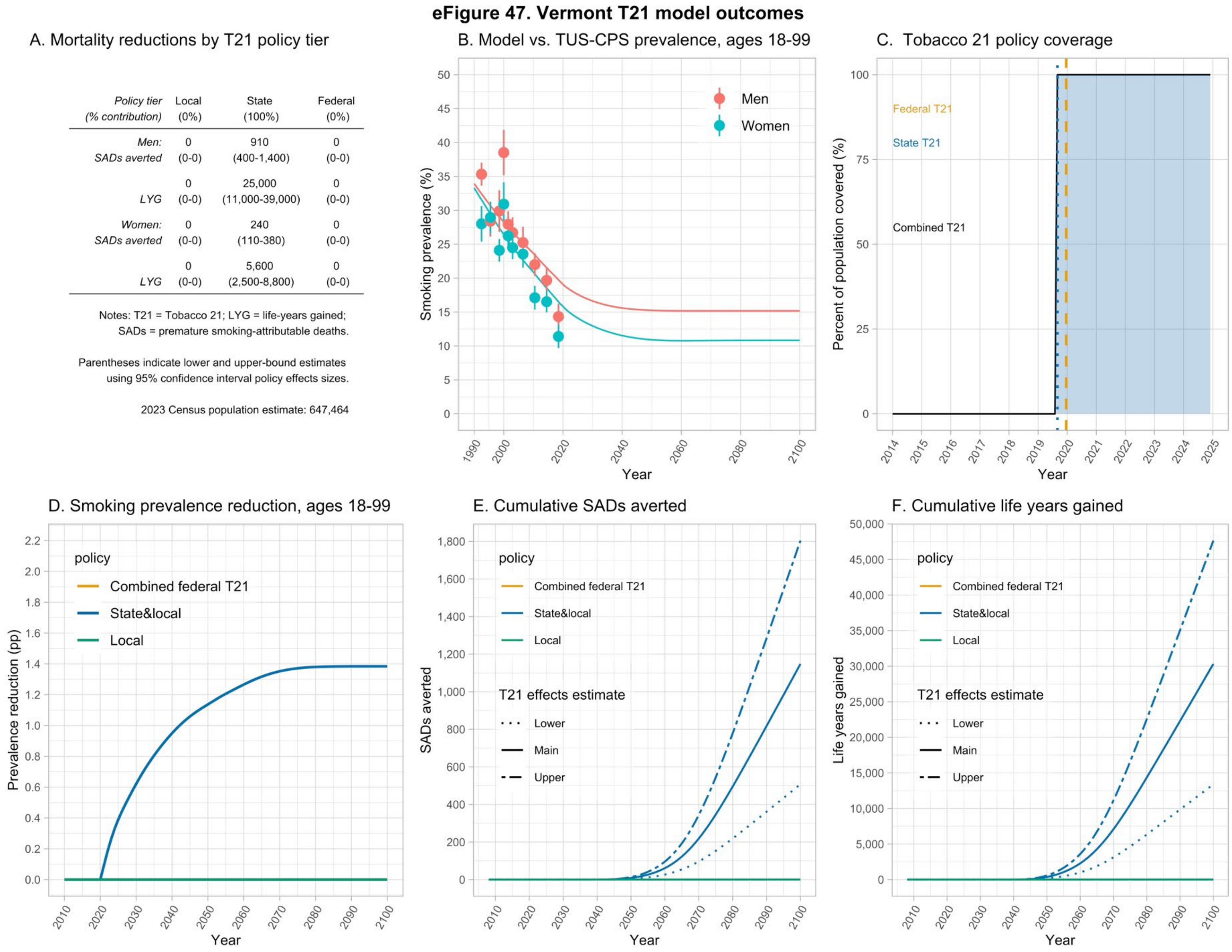

Policy tier (% contribution)	Local (0%)	State (100%)	Federal (0%)
Men: SADs averted	0 (0-0)	910 (400-1,400)	0 (0-0)
LYG	0 (0-0)	25,000 (11,000-39,000)	0 (0-0)
Women: SADs averted	0 (0-0)	240 (110-380)	0 (0-0)
LYG	0 (0-0)	5,600 (2,500-8,800)	0 (0-0)

© 2025 Tam J et al. JAMA Health Forum.

© 2025 Tam J et al. JAMA Health Forum.

eFigure 48. Virginia T21 model outcomes

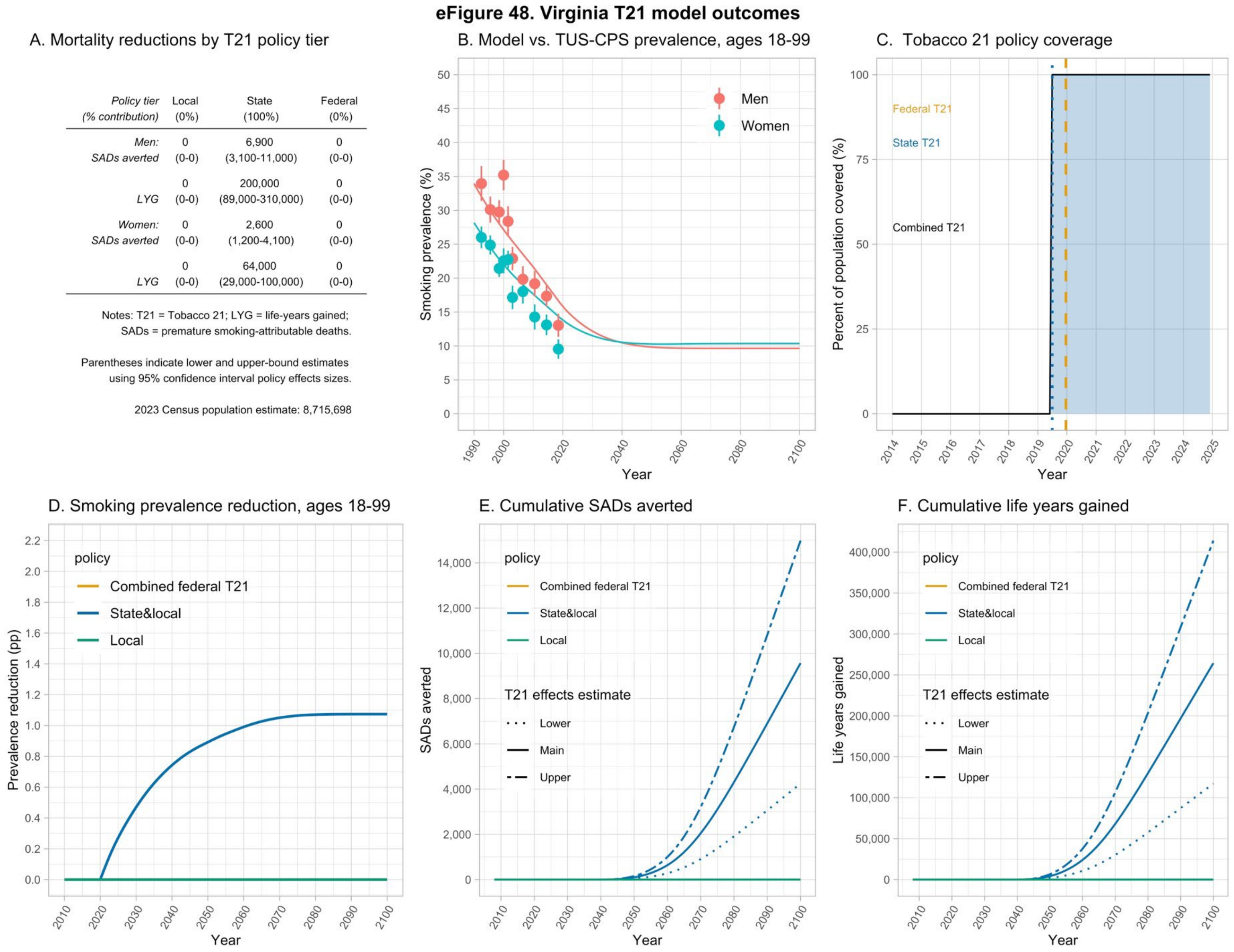

© 2025 Tam J et al. JAMA Health Forum.

© 2025 Tam J et al. JAMA Health Forum.

eFigure 49. Washington T21 model outcomes

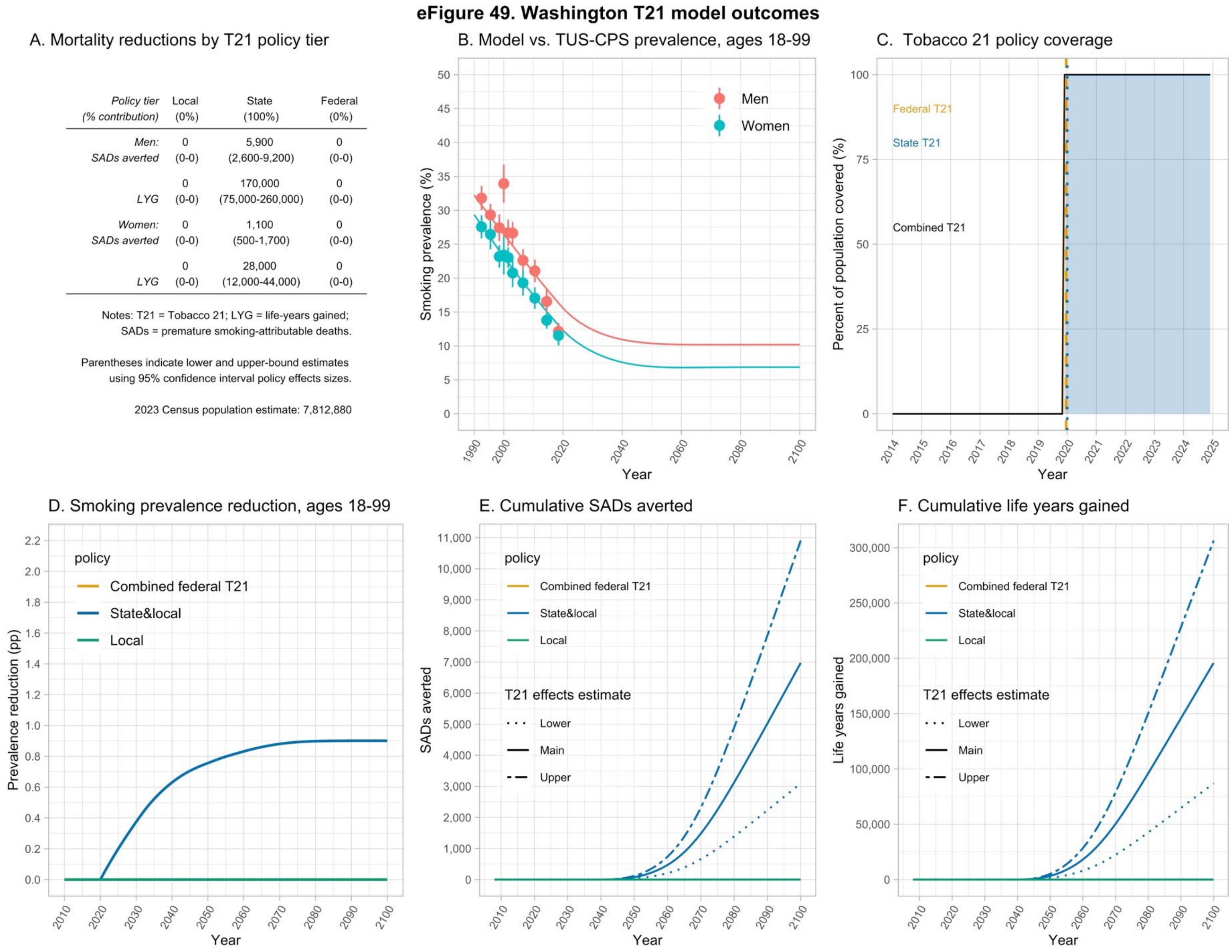

© 2025 Tam J et al. JAMA Health Forum.

© 2025 Tam J et al. JAMA Health Forum.

eFigure 50. West Virginia T21 model outcomes

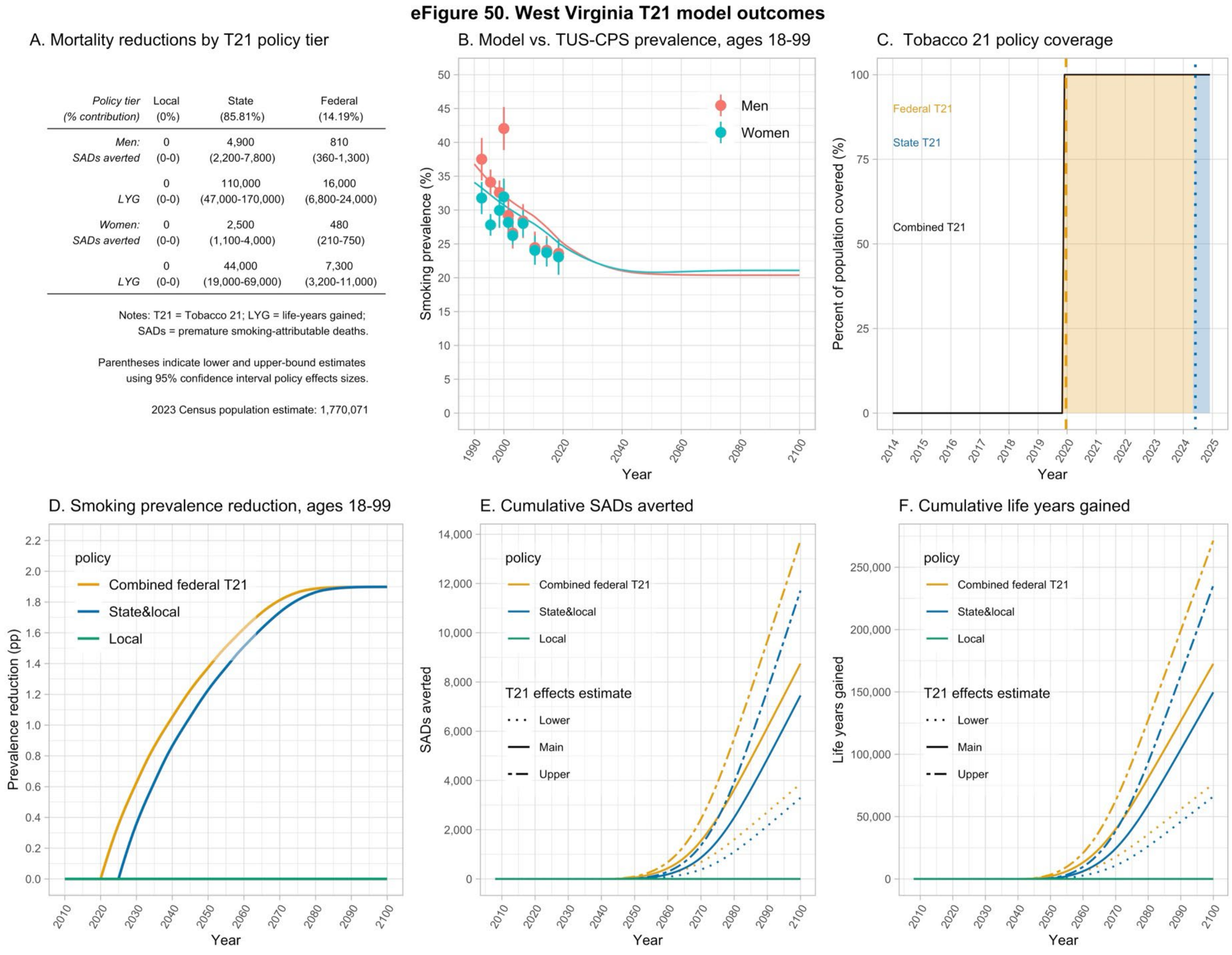

Policy tier (% contribution)	Local (0%)	State (85.81%)	Federal (14.19%)
Men: SADs averted	0 (0-0)	4,900 (2,200-7,800)	810 (360-1,300)
LYG	0 (0-0)	110,000 (47,000-170,000)	16,000 (6,800-24,000)
Women: SADs averted	0 (0-0)	2,500 (1,100-4,000)	480 (210-750)
LYG	0 (0-0)	44,000 (19,000-69,000)	7,300 (3,200-11,000)

Notes: T21 = Tobacco 21; LYG = life-years gained; SADs = premature smoking-attributable deaths.

Parentheses indicate lower and upper-bound estimates using 95% confidence interval policy effects sizes.

2023 Census population estimate: 1,770,071

© 2025 Tam J et al. JAMA Health Forum.

© 2025 Tam J et al. JAMA Health Forum.

eFigure 51. Wisconsin T21 model outcomes

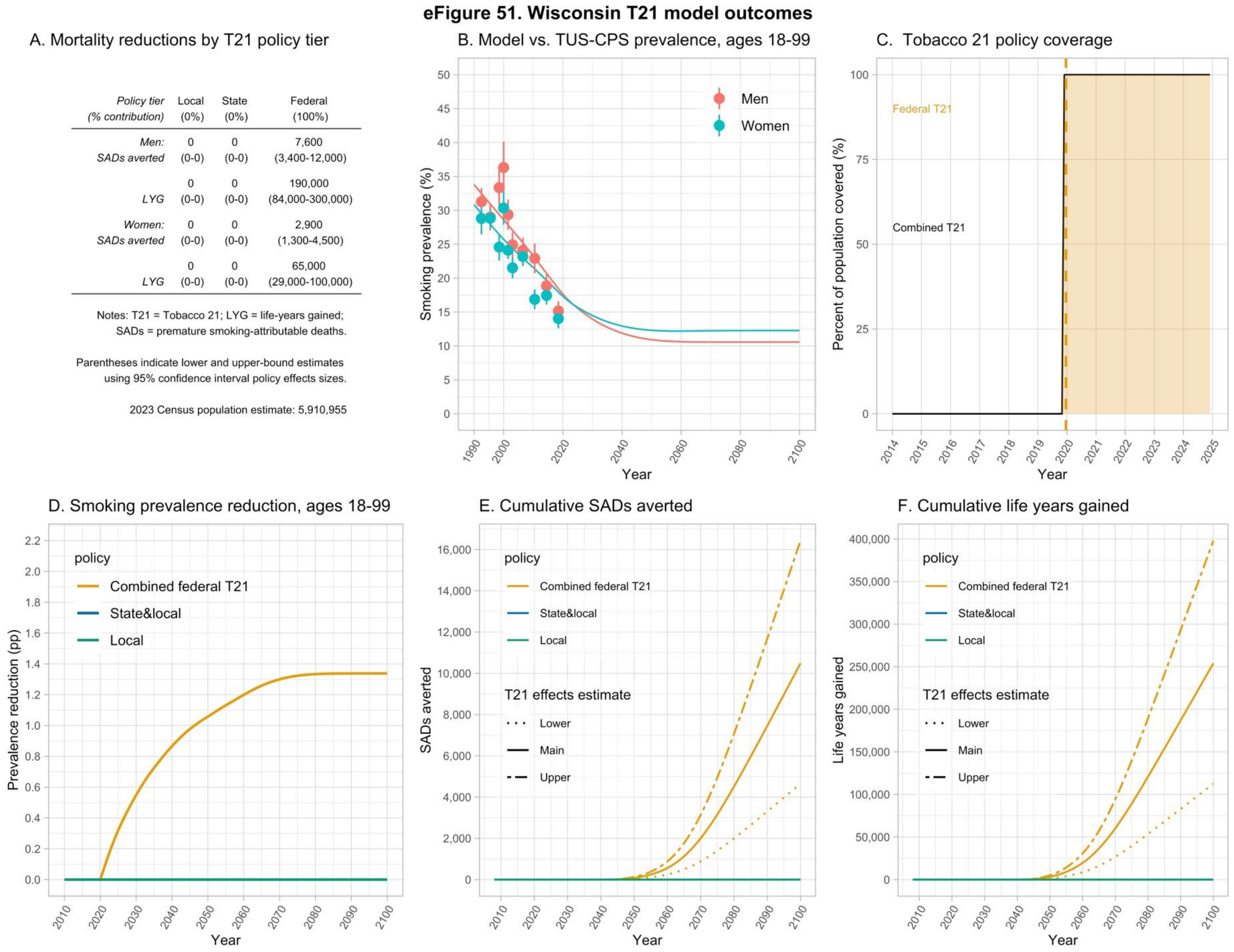

© 2025 Tam J et al. JAMA Health Forum.

© 2025 Tam J et al. JAMA Health Forum.

eFigure 52. Wyoming T21 model outcomes

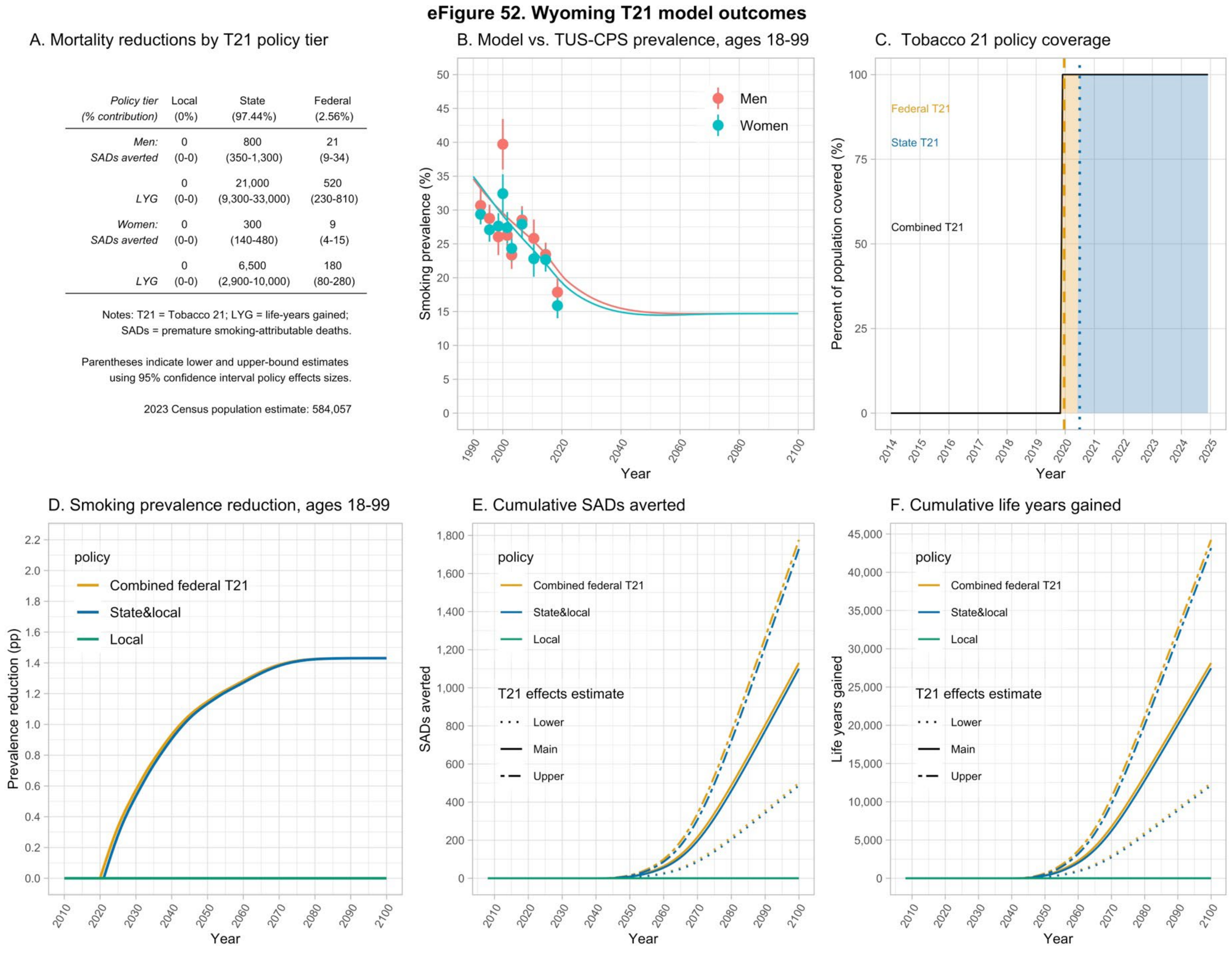

© 2025 Tam J et al. JAMA Health Forum.

Additional sensitivity analyses

In our main analysis, birth cohort trends are held constant at specific birth cohorts going forward (see above). To evaluate the impact of this assumption on our model outcomes, we conducted additional sensitivity analysis in which we vary smoking parameters for future birth cohorts for the four exemplar states, CA, KY, MA, and WI. To determine the trends in cohort effects that are extended forward, we took the average percent change in smoking initiation and cessation probabilities from the five preceding birth cohorts, and applied this percent change to the next ten birth cohorts going forward. This allowed us to run two scenarios:

1. Initiation probabilities continue to decline for another ten birth cohorts (from the 2012-2021 birth cohorts) before being held constant to the 2100 birth cohort.
2. Cessation probabilities continue to increase for another ten birth cohorts (from the 2004-2013 birth cohorts) before being held constant to the 2100 birth cohort.

Under a combined federal T21 policy scenario, continuing initiation cohort trends were associated with an average 0.2% reduction in SADs averted and LYG across the four exemplar states. This average was brought up by Wisconsin, where continuing initiation trends had the largest estimated change. Continuing cessation trends were associated with an average 0.4% reduction in SADs averted and LYG in the policy scenario. The results of this sensitivity analysis show that the assumption of fixed cohort effects going forward had very minor influence on our estimates. **eTable 6** below displays the difference in mortality outcomes relative to the main analysis.

eTable 6. Sensitivity analysis extending cohort smoking trends by an additional 10 birth cohorts

State	Probabilities changed	Change in SADs averted	Change in LYG	% change, SADs averted	% change, LYG
CA	initiation	-34	-1061	-0.12%	-0.13%
CA	cessation	-99	-3038	-0.37%	-0.37%
KY	initiation	-18	-351	-0.12%	-0.11%
KY	cessation	-56	-1170	-0.37%	-0.37%
MA	initiation	-12	-342	-0.15%	-0.15%
MA	cessation	-30	-846	-0.37%	-0.38%
WI	initiation	-42	-1072	-0.40%	-0.42%
WI	cessation	-48	-1141	-0.45%	-0.45%

Notes: Mortality estimates reflect the difference in cumulative SADs averted and LYG by 2100 relative to the main results presented in the manuscript. The policy scenario evaluated was the combined federal T21 scenario, using the main policy effects size of a 34% reduction in smoking initiation.

© 2025 Tam J et al. JAMA Health Forum.

State-by-state profile of T21 results assuming policy effects decline over time

eFigures 53-103 present a state-by-state profile of each model under an additional policy scenario in which Tobacco 21 policies become less effective over time. This scenario uses policy effects that decrease with a 20% exponential decay rate beginning in 2030. For example, if the main policy effect is a 34% reduction to initiation probabilities applied to ages 18-20, by 2030, this decreases to 27.2% $(= 0.34 \times (1 - r)^t)$, where r = decay rate (20%) and t = time in years (1 year). Each state profile under this scenario contains estimated smoking prevalence, Tobacco 21 policy coverage, and mortality reductions. This scenario shows substantially lower mortality reductions compared to our main results.

© 2025 Tam J et al. JAMA Health Forum.

eFigure 53. Alabama T21 model outcomes with policy decay

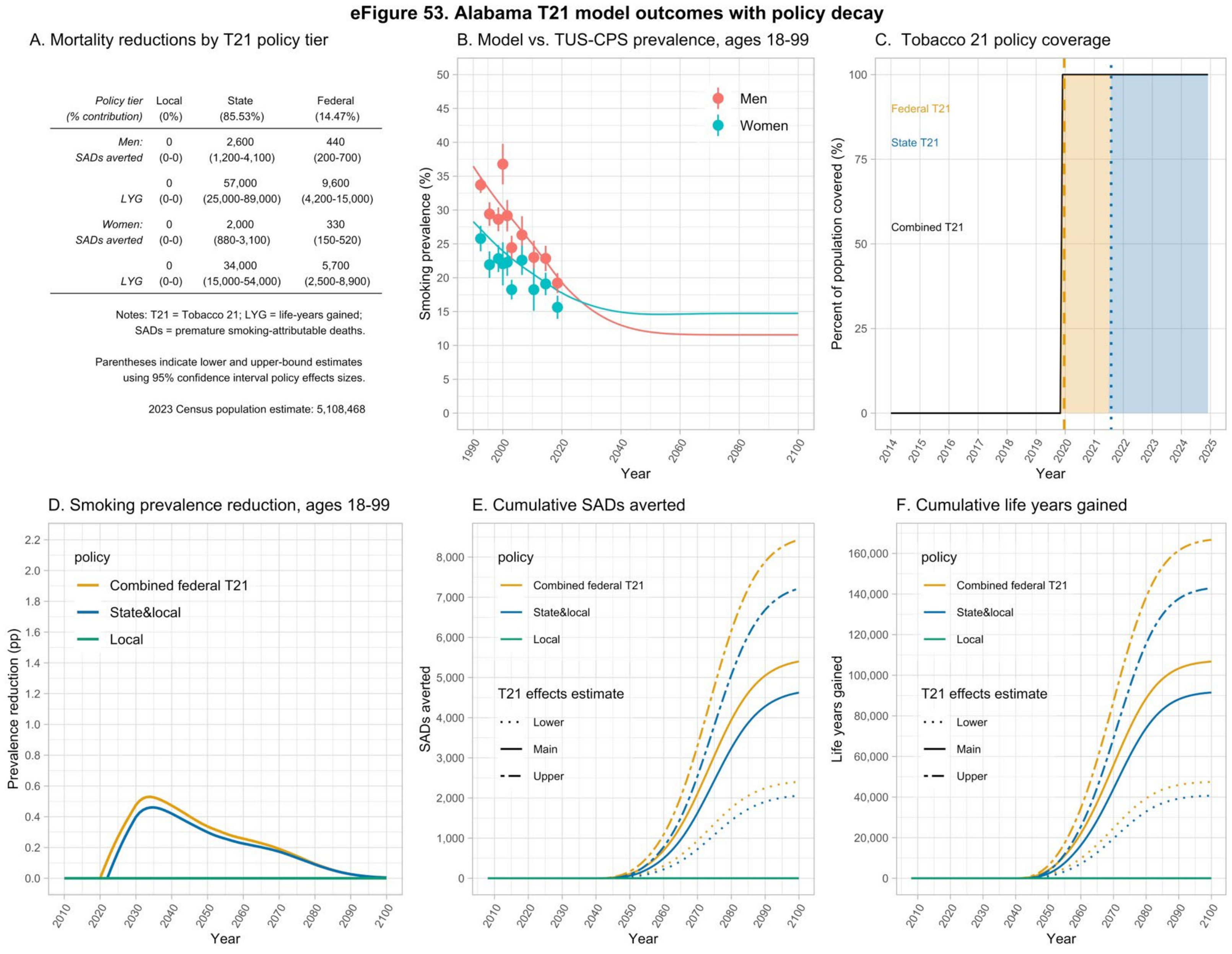

© 2025 Tam J et al. JAMA Health Forum.

eFigure 54. Alaska T21 model outcomes with policy decay

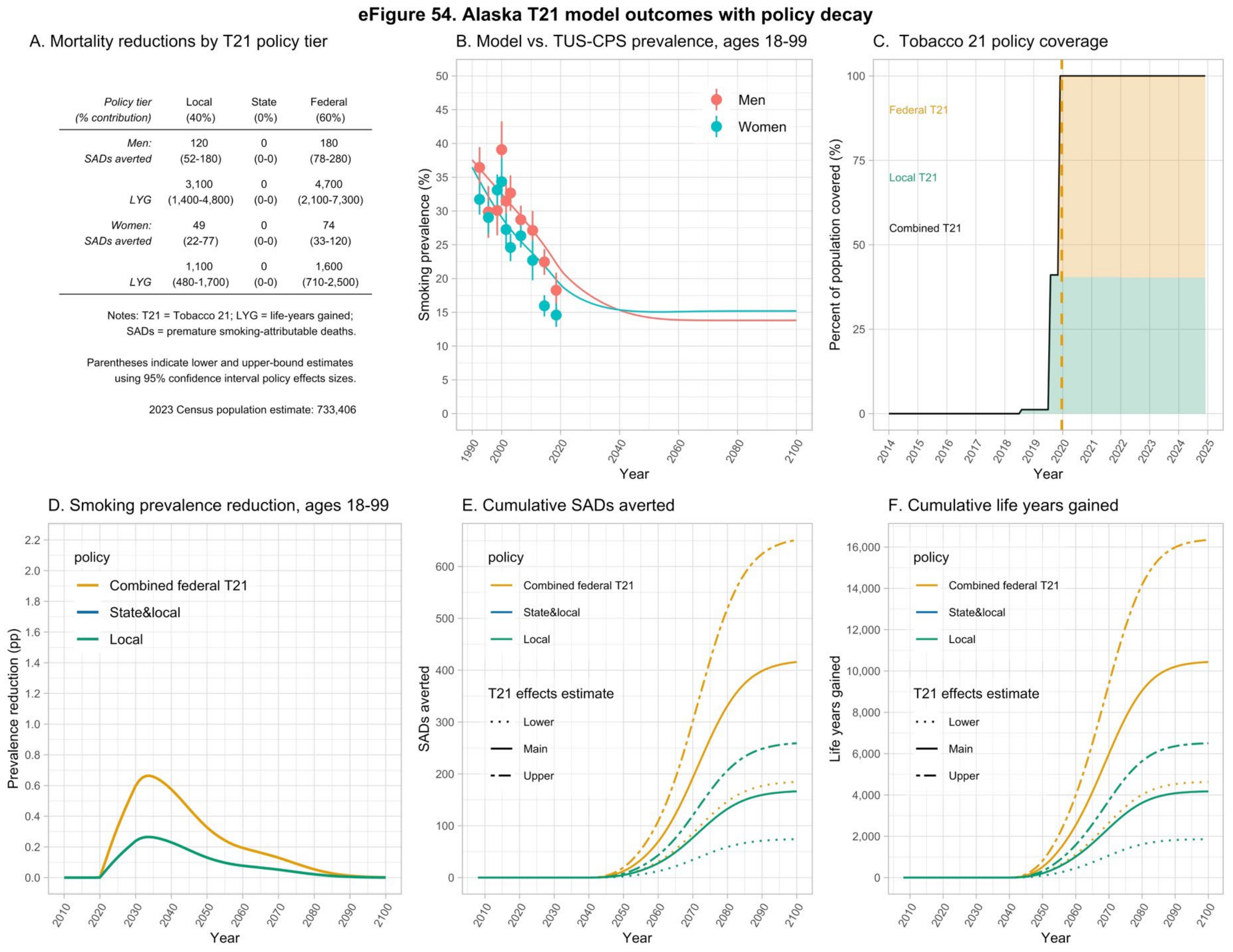

Policy tier (% contribution)	Local (40%)	State (0%)	Federal (60%)
Men: SADs averted	120 (52-180)	0 (0-0)	180 (78-280)
LYG	3,100 (1,400-4,800)	0 (0-0)	4,700 (2,100-7,300)
Women: SADs averted	49 (22-77)	0 (0-0)	74 (33-120)
LYG	1,100 (480-1,700)	0 (0-0)	1,600 (710-2,500)

Notes: T21 = Tobacco 21; LYG = life-years gained; SADs = premature smoking-attributable deaths.

Parentheses indicate lower and upper-bound estimates using 95% confidence interval policy effects sizes.

2023 Census population estimate: 733,406

© 2025 Tam J et al. JAMA Health Forum.

eFigure 55. Arizona T21 model outcomes with policy decay

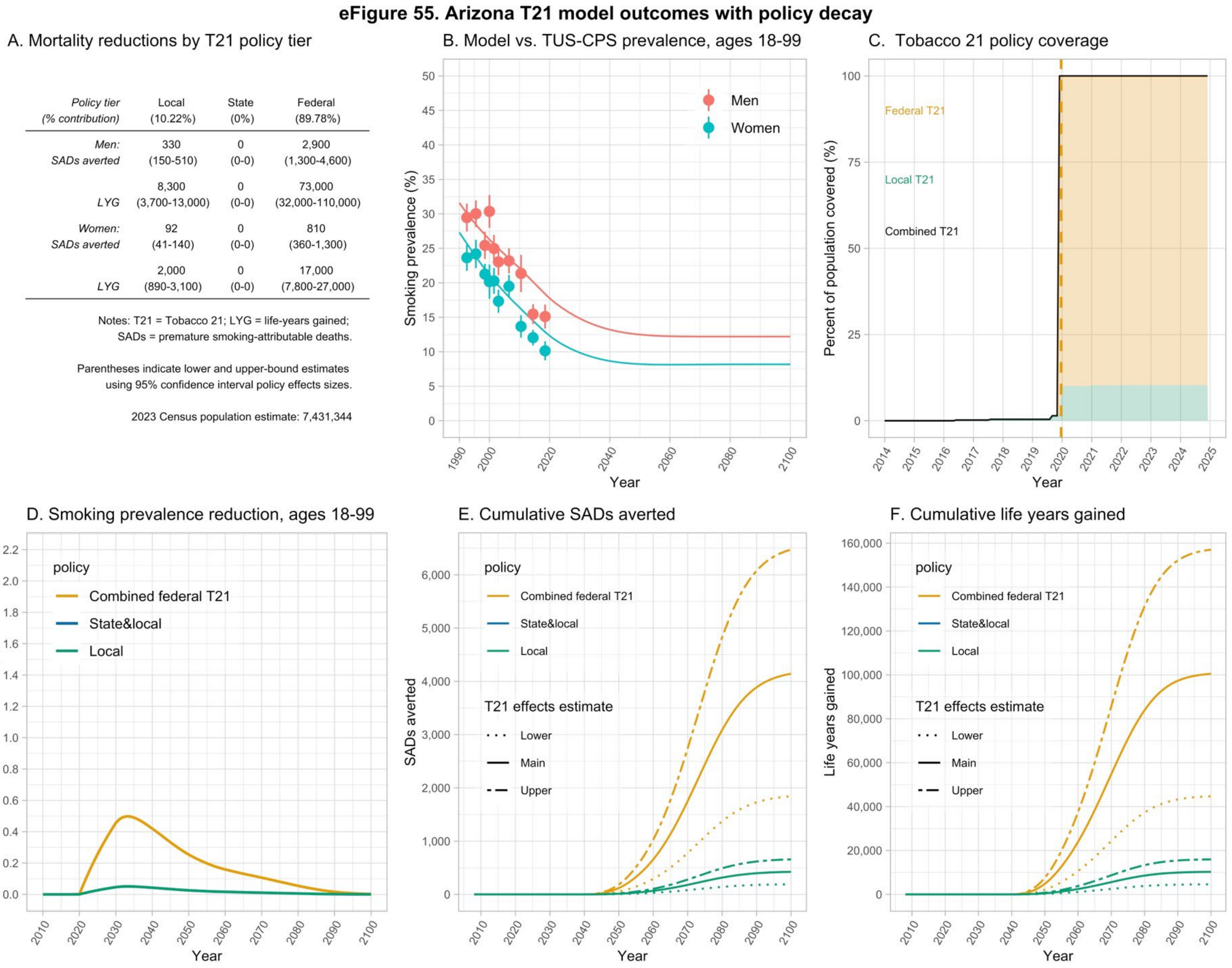

Policy tier (% contribution)	Local (10.22%)	State (0%)	Federal (89.78%)
Men: SADs averted	330 (150-510)	0 (0-0)	2,900 (1,300-4,600)
LYG	8,300 (3,700-13,000)	0 (0-0)	73,000 (32,000-110,000)
Women: SADs averted	92 (41-140)	0 (0-0)	810 (360-1,300)
LYG	2,000 (890-3,100)	0 (0-0)	17,000 (7,800-27,000)

Notes: T21 = Tobacco 21; LYG = life-years gained; SADs = premature smoking-attributable deaths.

Parentheses indicate lower and upper-bound estimates using 95% confidence interval policy effects sizes.

2023 Census population estimate: 7,431,344

© 2025 Tam J et al. JAMA Health Forum.

© 2025 Tam J et al. JAMA Health Forum.

eFigure 56. Arkansas T21 model outcomes with policy decay

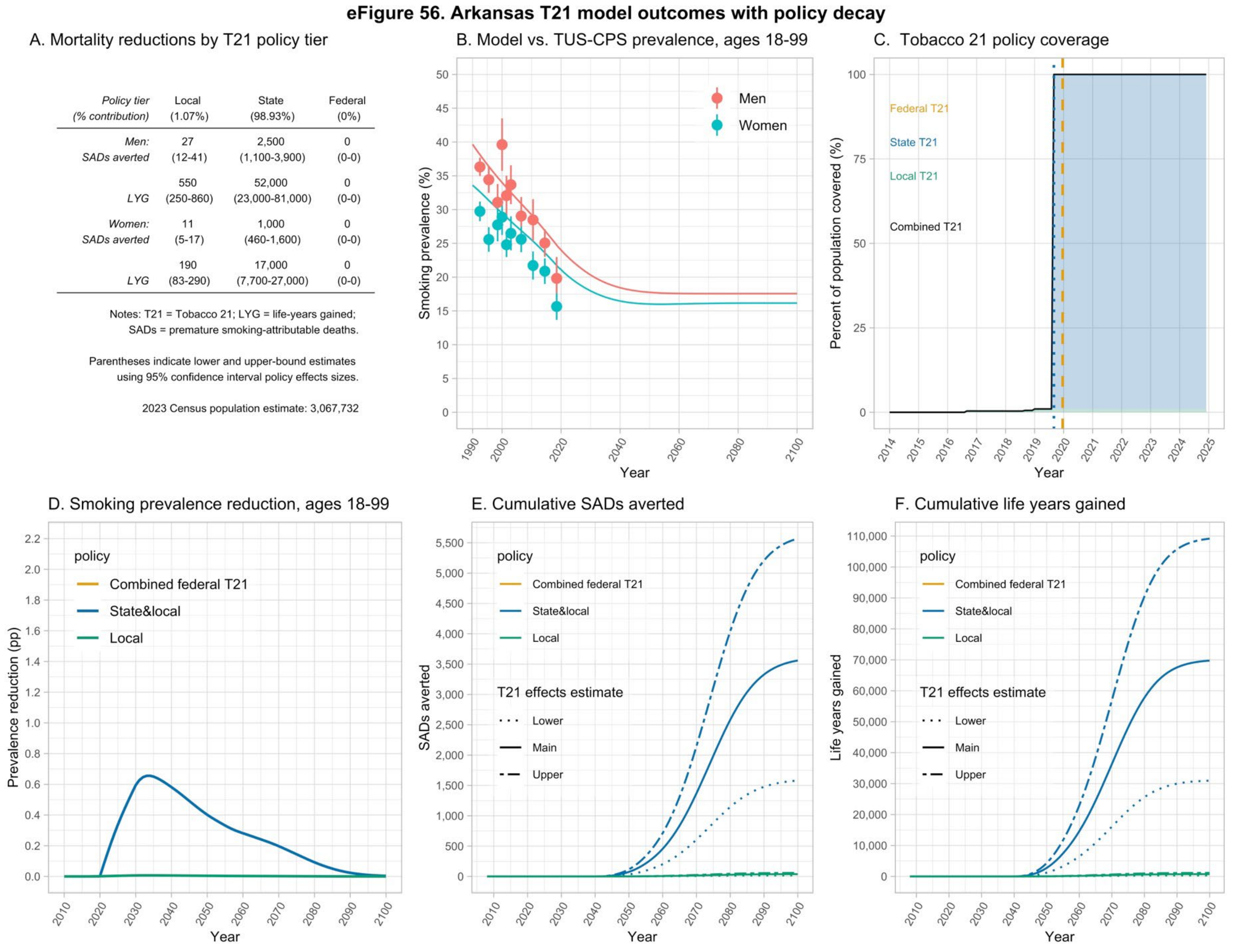

Policy tier (% contribution)	Local (1.07%)	State (98.93%)	Federal (0%)
Men: SADs averted	27 (12-41)	2,500 (1,100-3,900)	0 (0-0)
LYG	550 (250-860)	52,000 (23,000-81,000)	0 (0-0)
Women: SADs averted	11 (5-17)	1,000 (460-1,600)	0 (0-0)
LYG	190 (83-290)	17,000 (7,700-27,000)	0 (0-0)

Notes: T21 = Tobacco 21; LYG = life-years gained; SADs = premature smoking-attributable deaths.

Parentheses indicate lower and upper-bound estimates using 95% confidence interval policy effects sizes.

2023 Census population estimate: 3,067,732

© 2025 Tam J et al. JAMA Health Forum.

© 2025 Tam J et al. JAMA Health Forum.

eFigure 57. California T21 model outcomes with policy decay

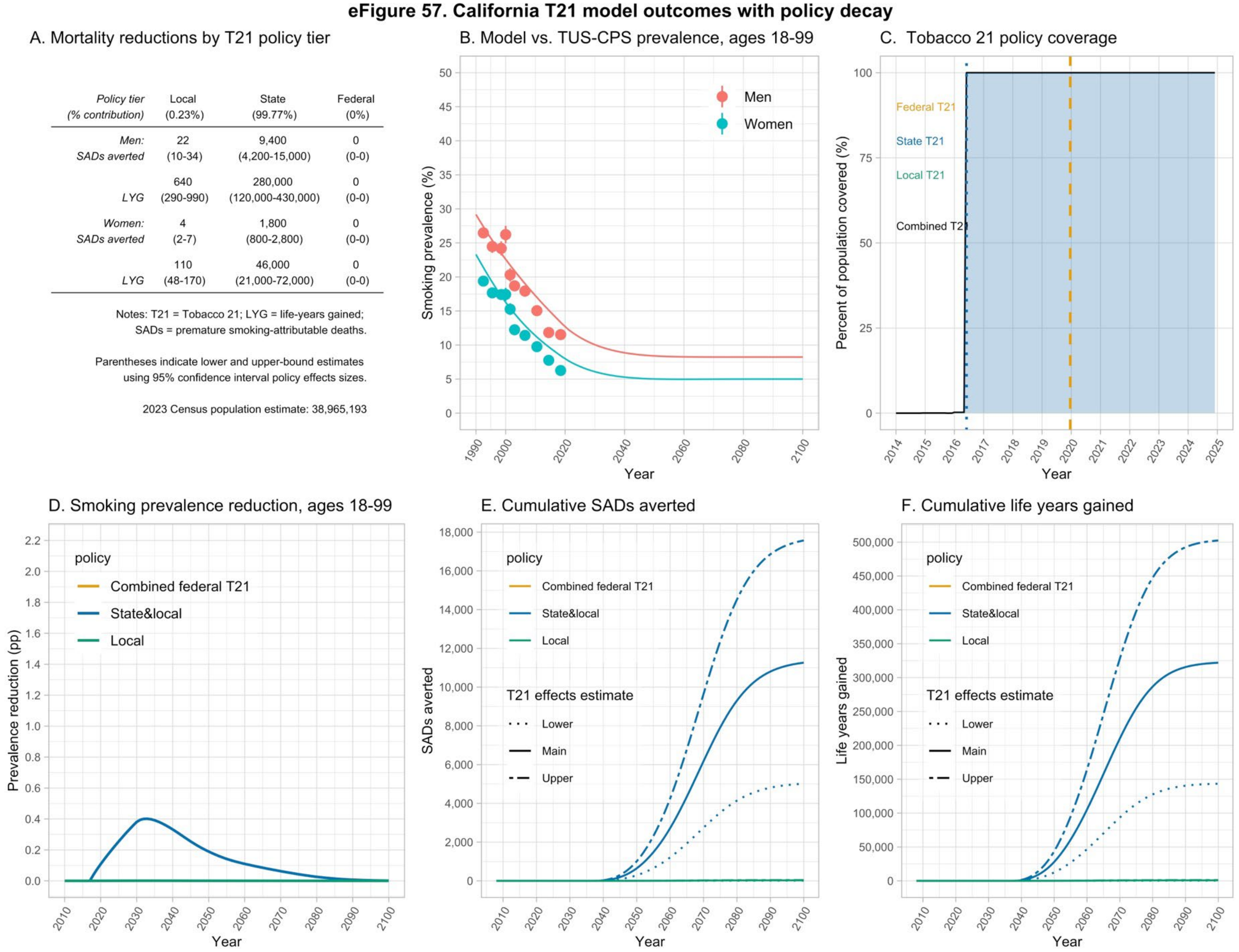

Policy tier (% contribution)	Local (0.23%)	State (99.77%)	Federal (0%)
Men: SADs averted	22 (10-34)	9,400 (4,200-15,000)	0 (0-0)
LYG	640 (290-990)	280,000 (120,000-430,000)	0 (0-0)
Women: SADs averted	4 (2-7)	1,800 (800-2,800)	0 (0-0)
LYG	110 (48-170)	46,000 (21,000-72,000)	0 (0-0)

Notes: T21 = Tobacco 21; LYG = life-years gained; SADs = premature smoking-attributable deaths.

Parentheses indicate lower and upper-bound estimates using 95% confidence interval policy effects sizes.

2023 Census population estimate: 38,965,193

© 2025 Tam J et al. JAMA Health Forum.

© 2025 Tam J et al. JAMA Health Forum.

eFigure 58. Colorado T21 model outcomes with policy decay

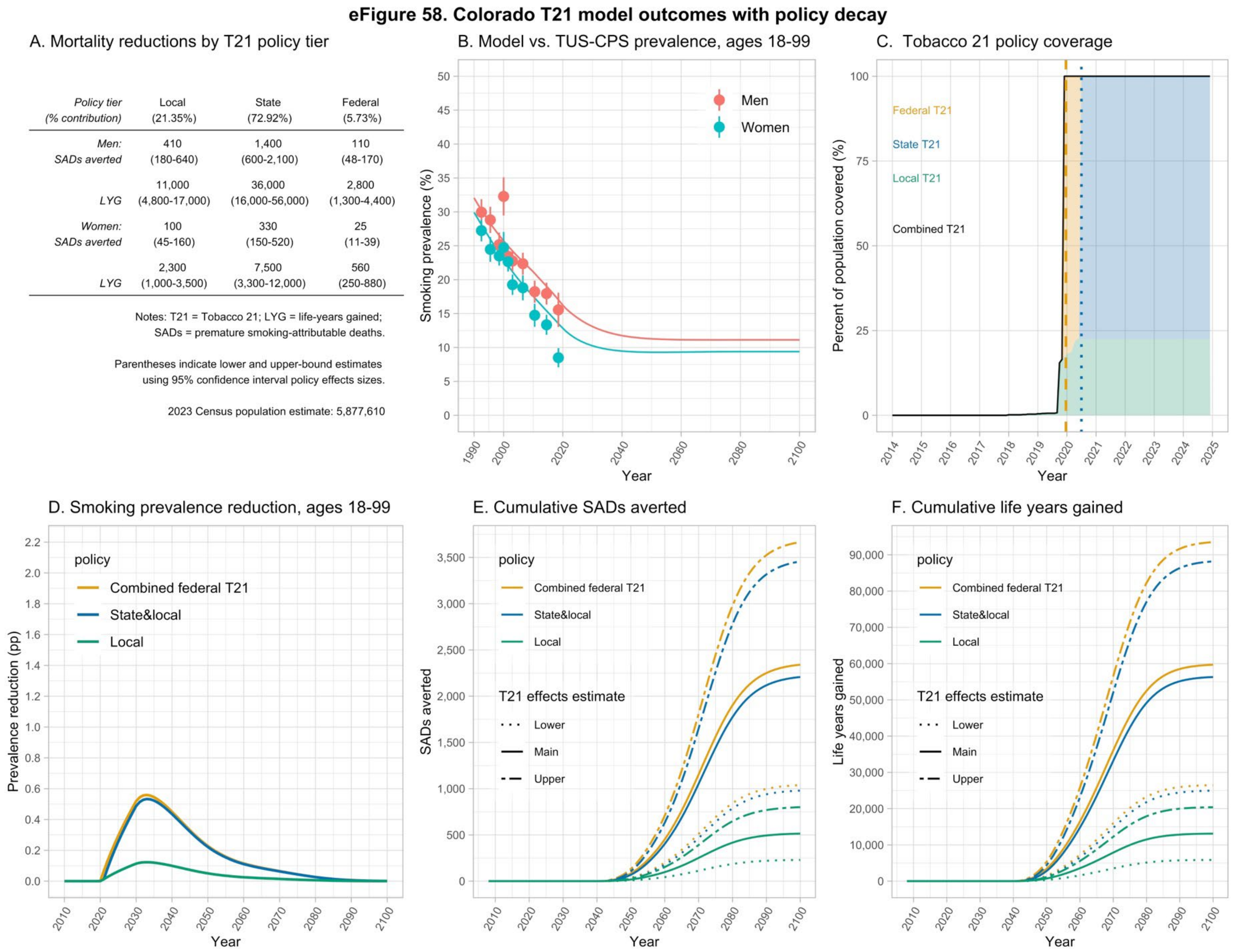

© 2025 Tam J et al. JAMA Health Forum.

© 2025 Tam J et al. JAMA Health Forum.

eFigure 59. Connecticut T21 model outcomes with policy decay

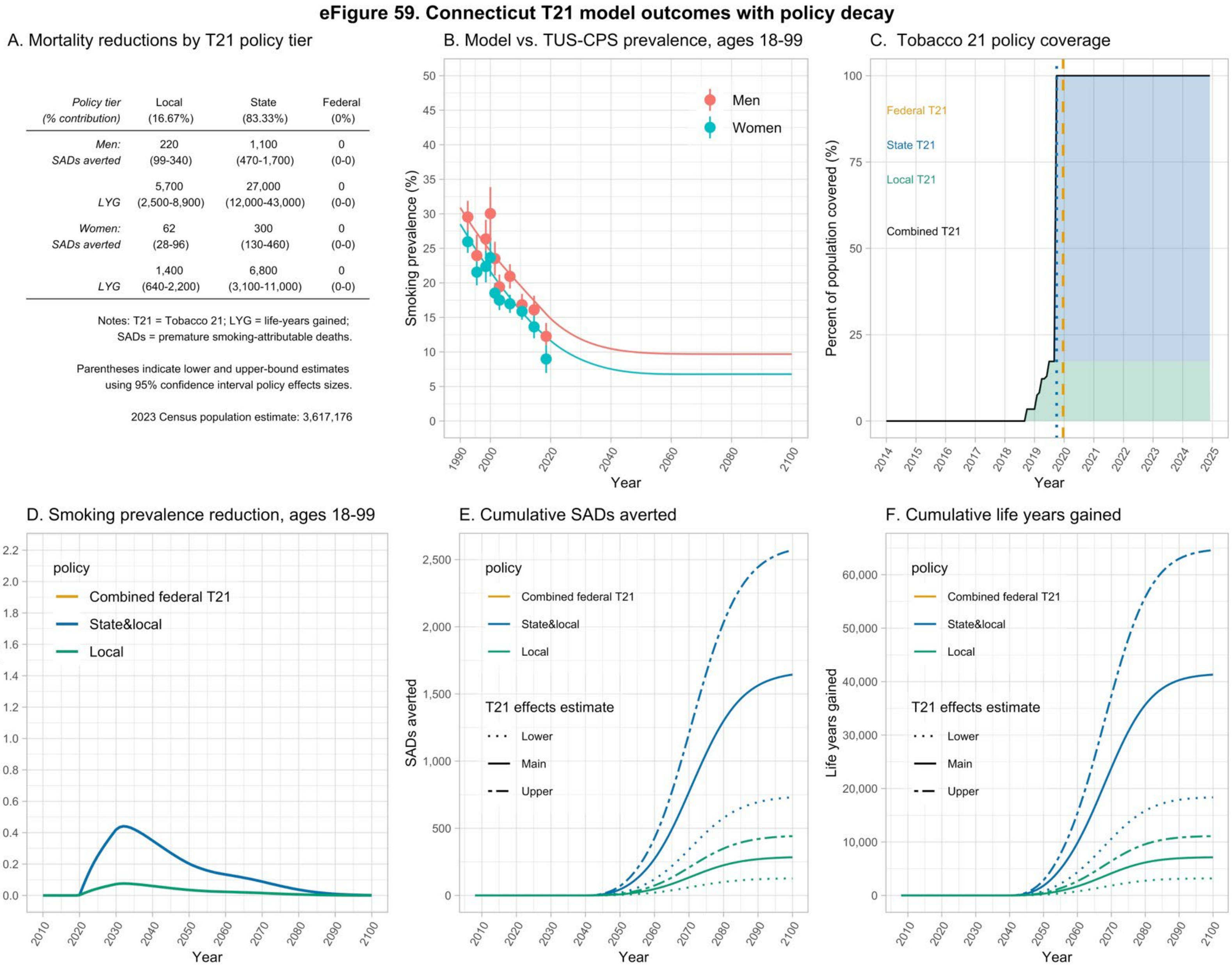

© 2025 Tam J et al. JAMA Health Forum.

© 2025 Tam J et al. JAMA Health Forum.

eFigure 60. Delaware T21 model outcomes with policy decay

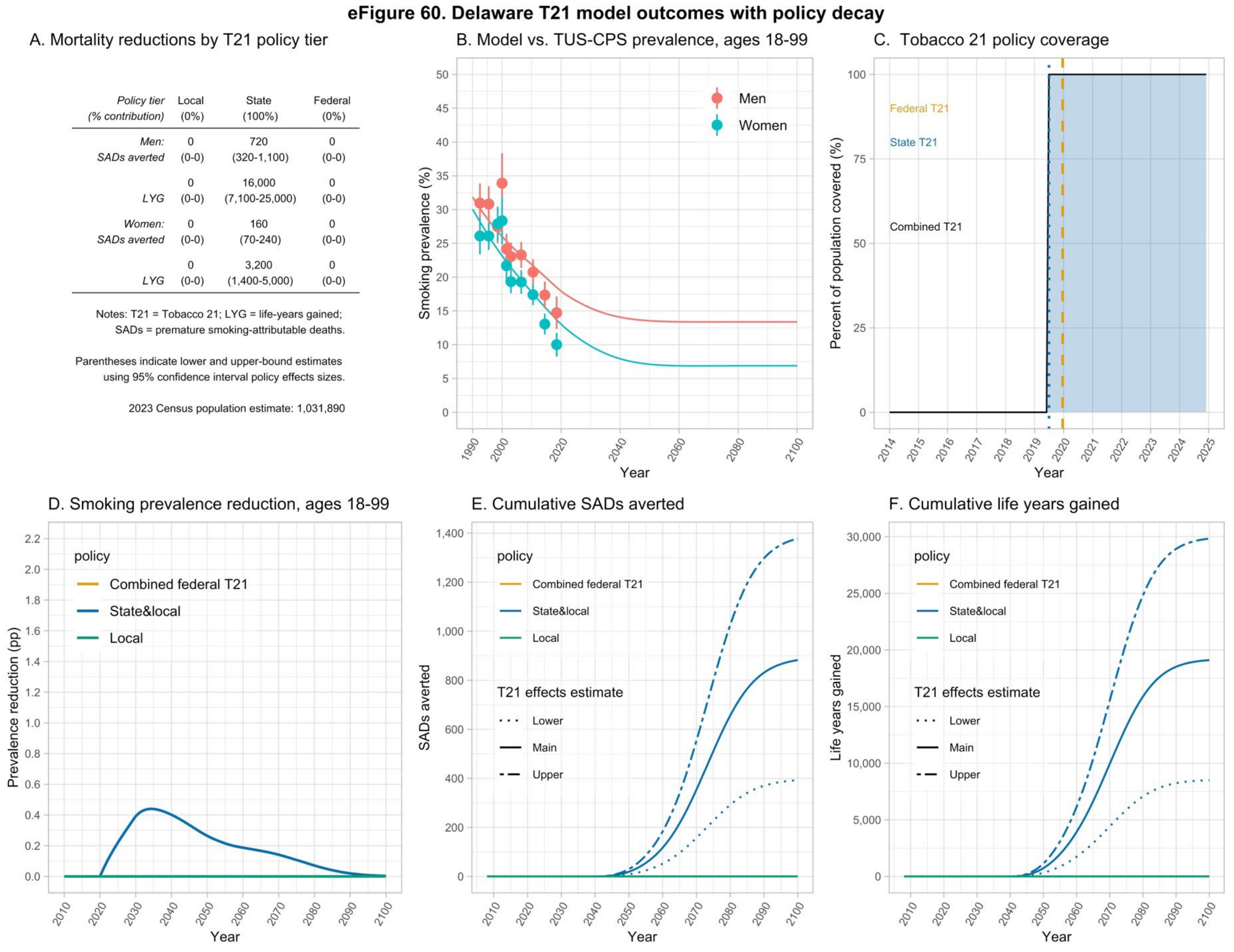

© 2025 Tam J et al. JAMA Health Forum.

© 2025 Tam J et al. JAMA Health Forum.

eFigure 61. District of Columbia T21 model outcomes with policy decay

eFigure 61. District of Columbia T21 model outcomes with policy decay

A. Mortality reductions by T21 policy tier

Policy tier (% contribution)	Local (0%)	State (100%)	Federal (0%)
Men: SADs averted	0 (0-0)	100 (46-160)	0 (0-0)
LYG	0 (0-0)	3,500 (1,600-5,500)	0 (0-0)
Women: SADs averted	0 (0-0)	25 (11-39)	0 (0-0)
LYG	0 (0-0)	690 (310-1,100)	0 (0-0)

Notes: T21 = Tobacco 21; LYG = life-years gained; SADs = premature smoking-attributable deaths.

Parentheses indicate lower and upper-bound estimates using 95% confidence interval policy effects sizes.

2023 Census population estimate: 678,972

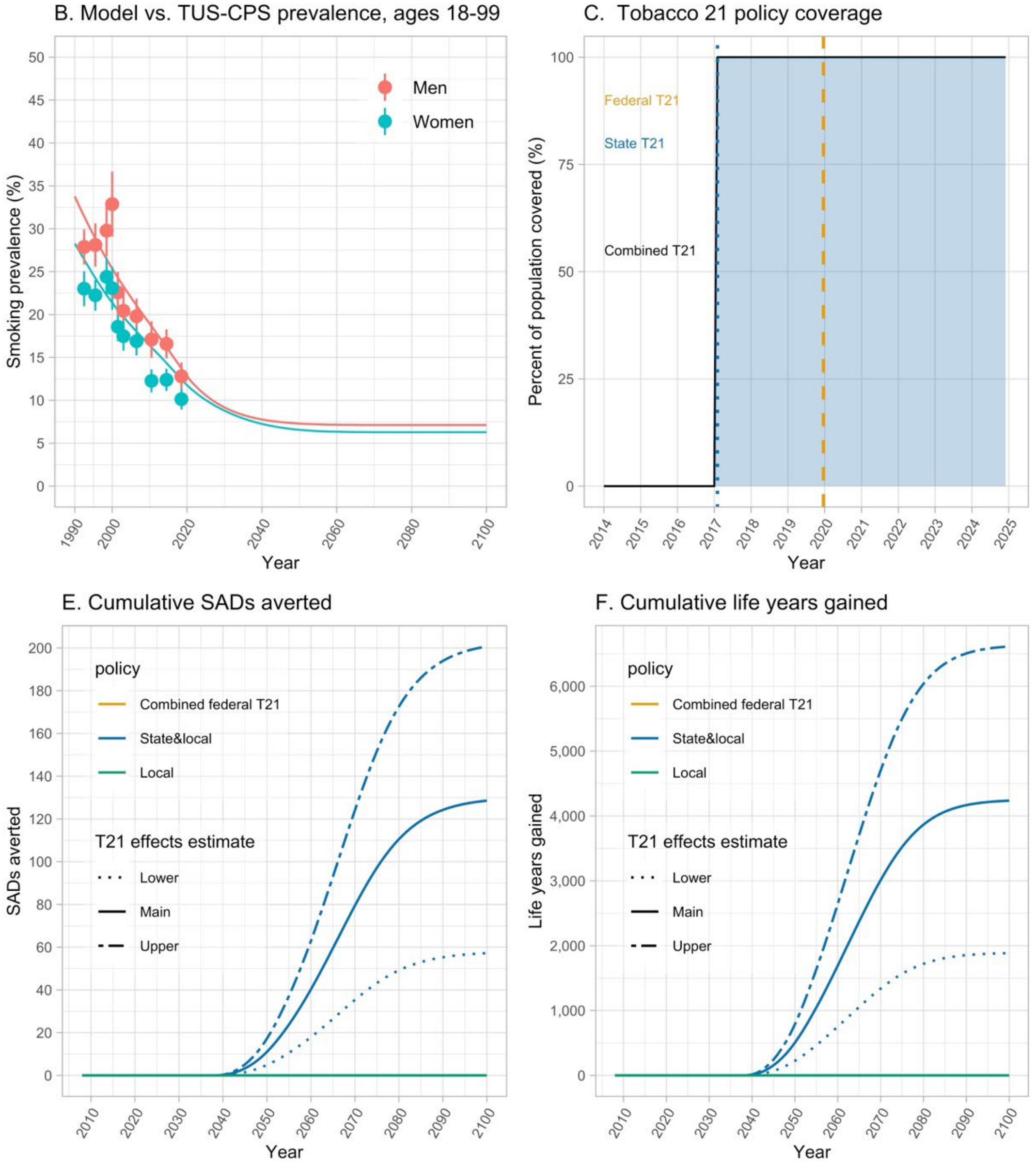

© 2025 Tam J et al. JAMA Health Forum.

© 2025 Tam J et al. JAMA Health Forum.

eFigure 62. Florida T21 model outcomes with policy decay

eFigure 62. Florida T21 model outcomes with policy decay

A. Mortality reductions by T21 policy tier

Policy tier (% contribution)	Local (3.47%)	State (82.92%)	Federal (13.61%)
Men: SADs averted	280 (120-430)	6,700 (3,000-11,000)	1,100 (510-1,800)
LYG	7,200 (3,200-11,000)	180,000 (79,000-280,000)	30,000 (13,000-47,000)
Women: SADs averted	110 (47-160)	2,600 (1,200-4,000)	430 (190-660)
LYG	2,300 (1,000-3,600)	57,000 (25,000-89,000)	9,200 (4,100-14,000)

Notes: T21 = Tobacco 21; LYG = life-years gained; SADs = premature smoking-attributable deaths.

Parentheses indicate lower and upper-bound estimates using 95% confidence interval policy effects sizes.

2023 Census population estimate: 22,610,726

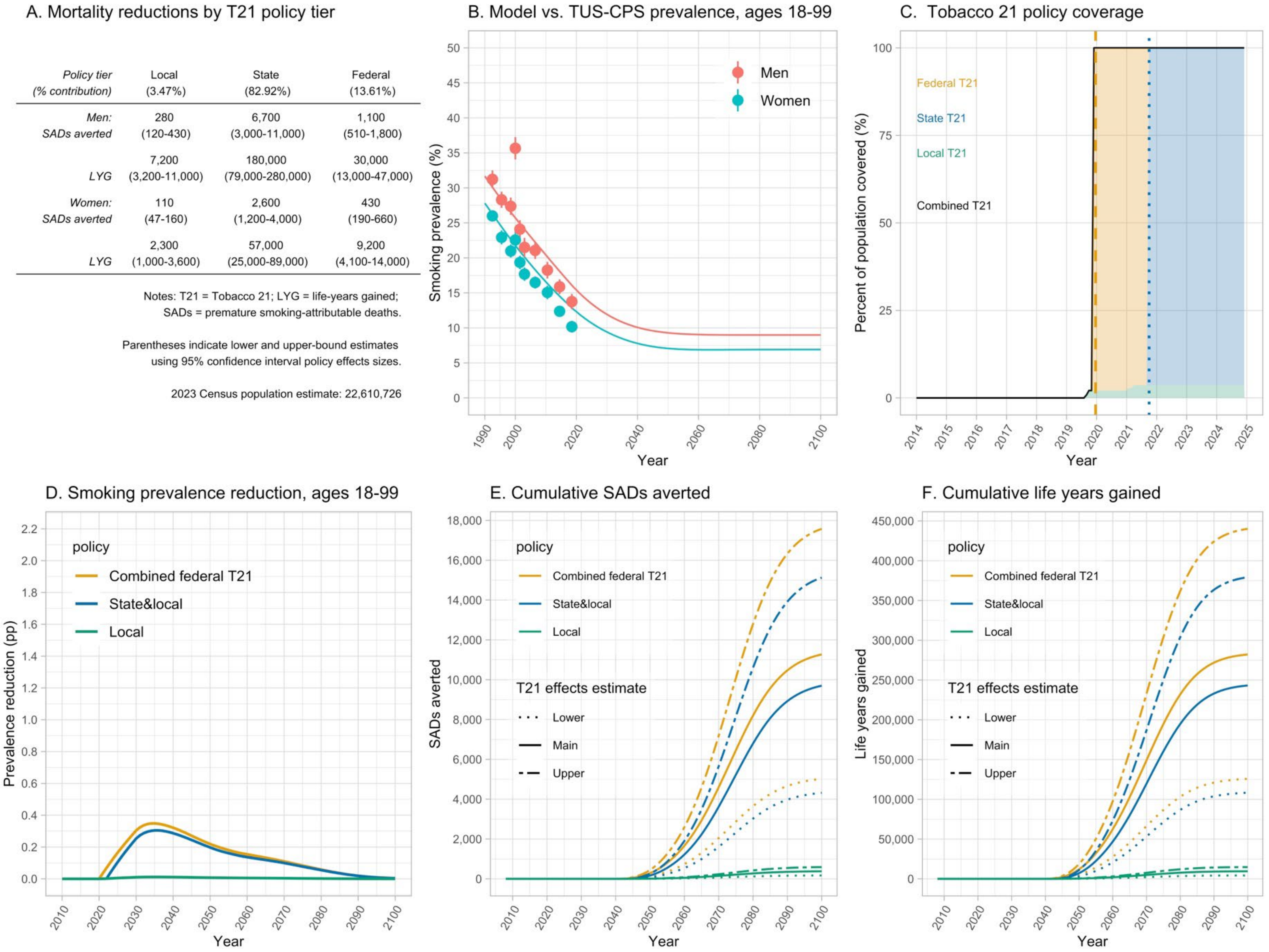

© 2025 Tam J et al. JAMA Health Forum.

© 2025 Tam J et al. JAMA Health Forum.

eFigure 63. Georgia T21 model outcomes with policy decay

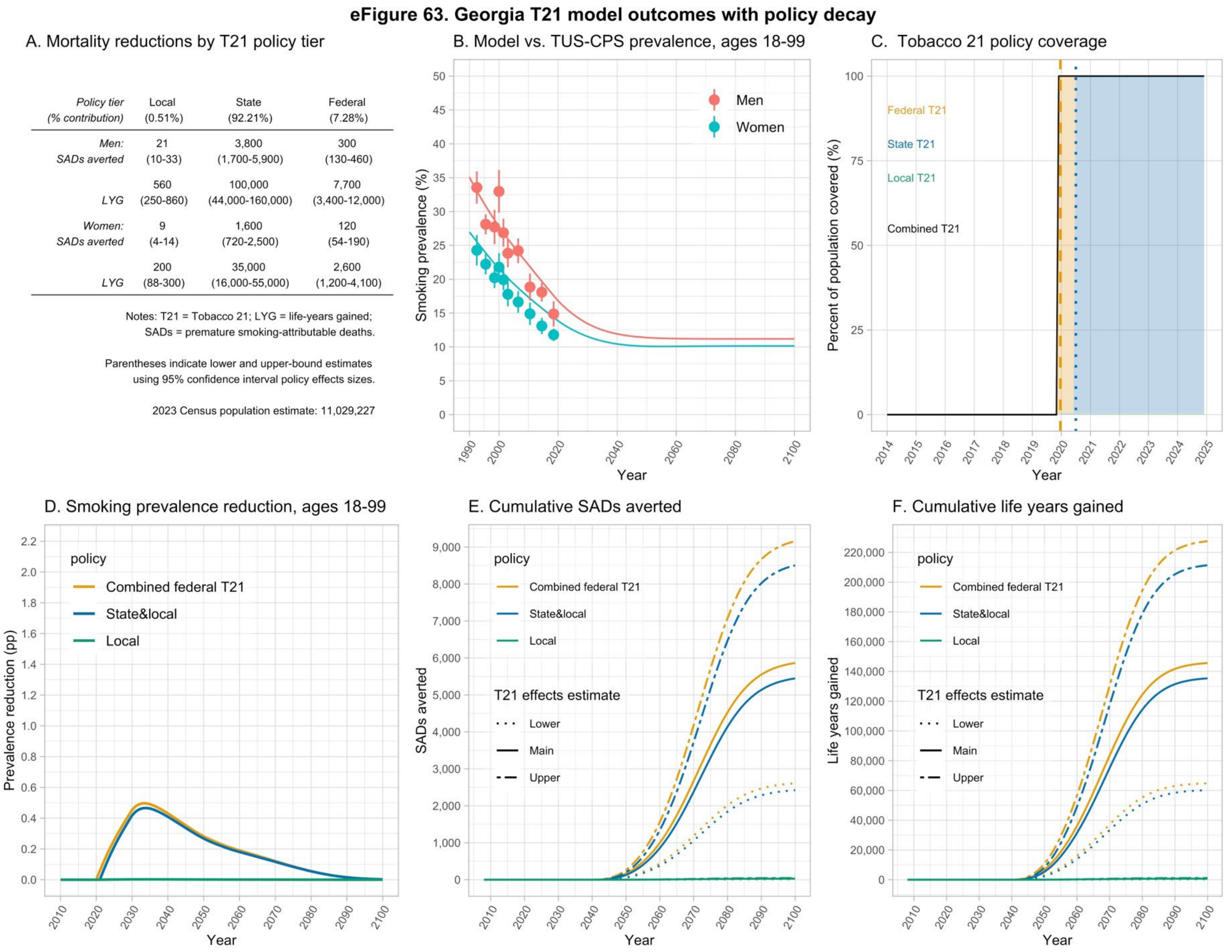

© 2025 Tam J et al. JAMA Health Forum.

© 2025 Tam J et al. JAMA Health Forum.

eFigure 64. Hawaii T21 model outcomes with policy decay

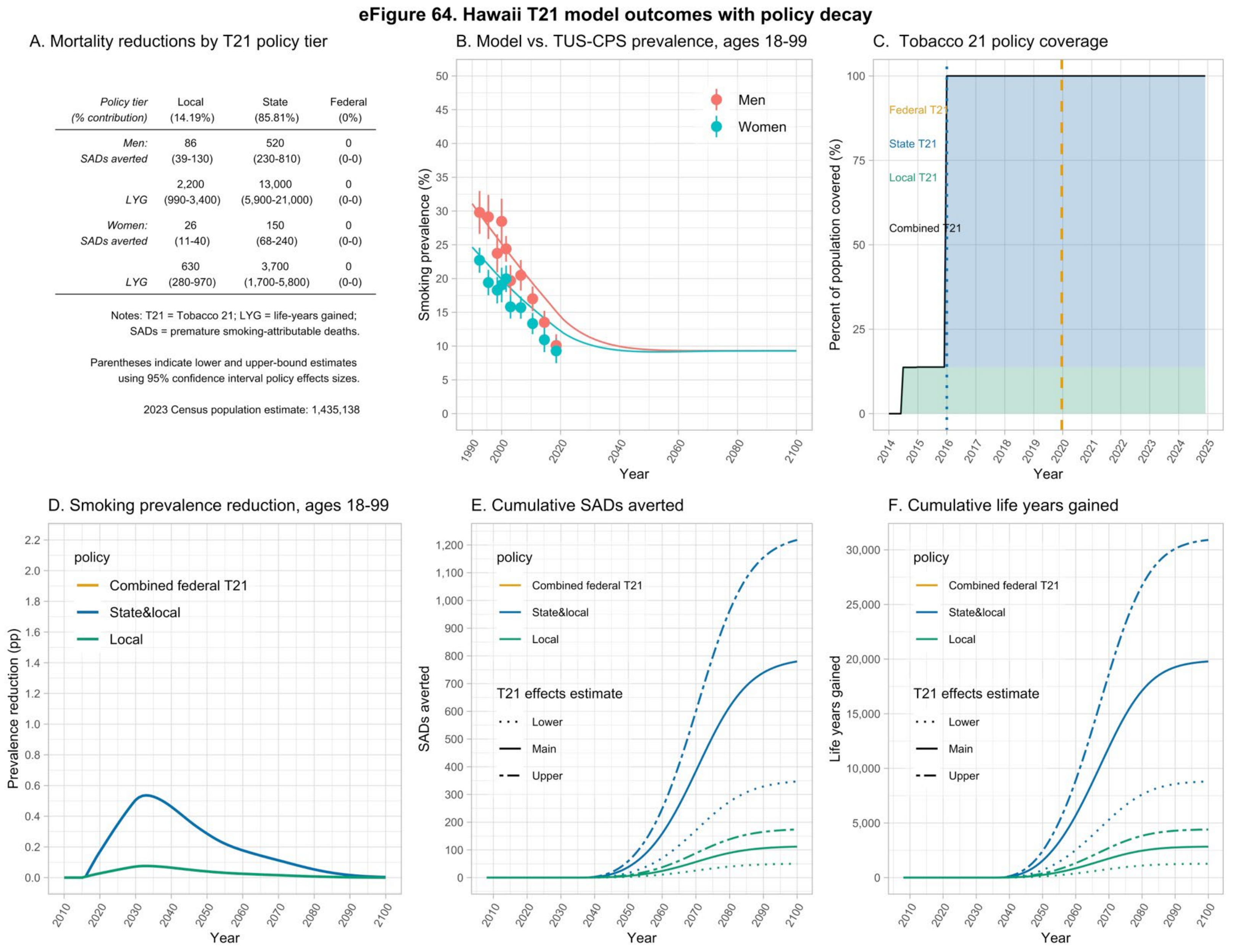

Policy tier (% contribution)	Local (14.19%)	State (85.81%)	Federal (0%)
Men: SADs averted	86 (39-130)	520 (230-810)	0 (0-0)
LYG	2,200 (990-3,400)	13,000 (5,900-21,000)	0 (0-0)
Women: SADs averted	26 (11-40)	150 (68-240)	0 (0-0)
LYG	630 (280-970)	3,700 (1,700-5,800)	0 (0-0)

Notes: T21 = Tobacco 21; LYG = life-years gained; SADs = premature smoking-attributable deaths.

Parentheses indicate lower and upper-bound estimates using 95% confidence interval policy effects sizes.

2023 Census population estimate: 1,435,138

© 2025 Tam J et al. JAMA Health Forum.

© 2025 Tam J et al. JAMA Health Forum.

eFigure 65. Idaho T21 model outcomes with policy decay

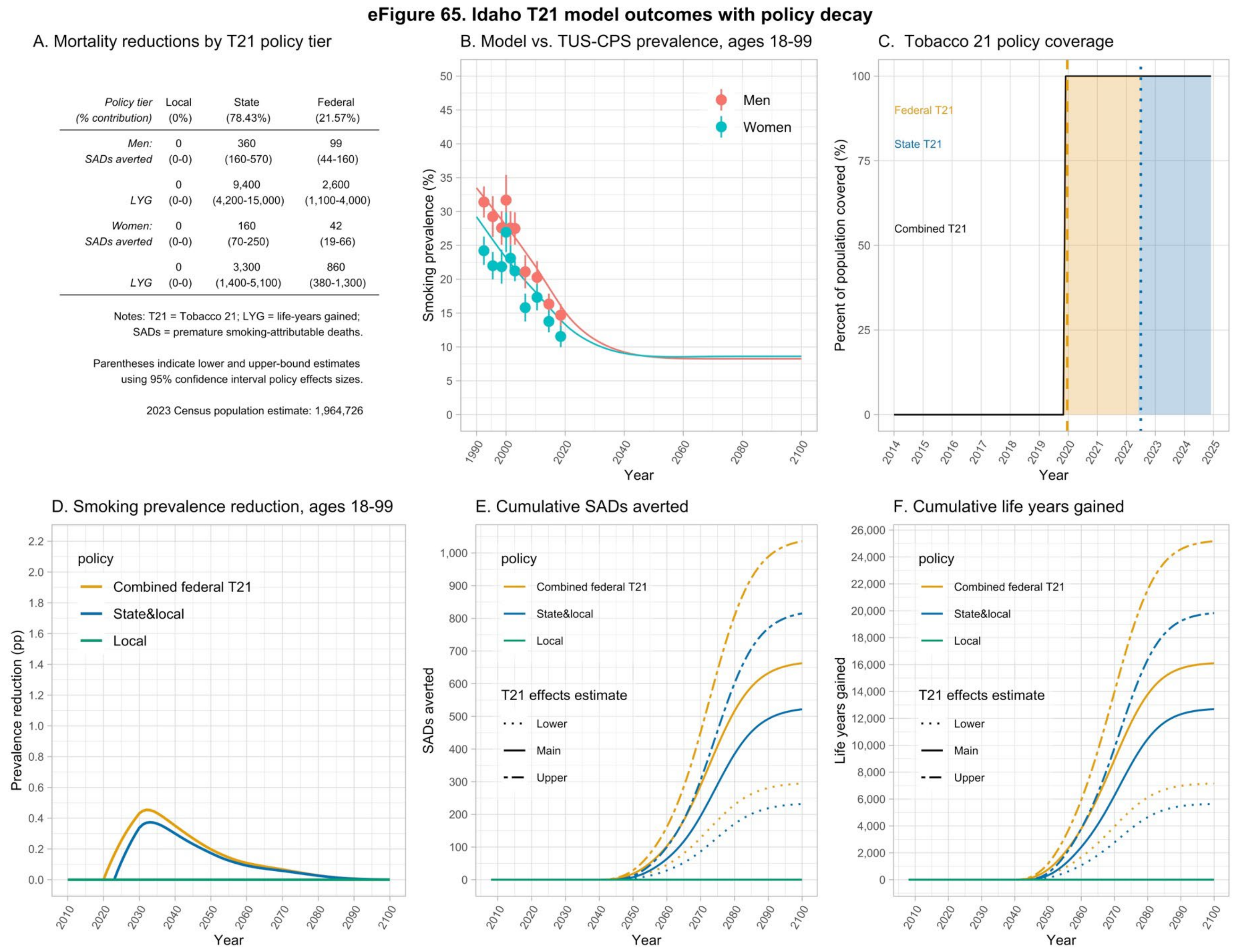

© 2025 Tam J et al. JAMA Health Forum.

© 2025 Tam J et al. JAMA Health Forum.

eFigure 66. Illinois T21 model outcomes with policy decay

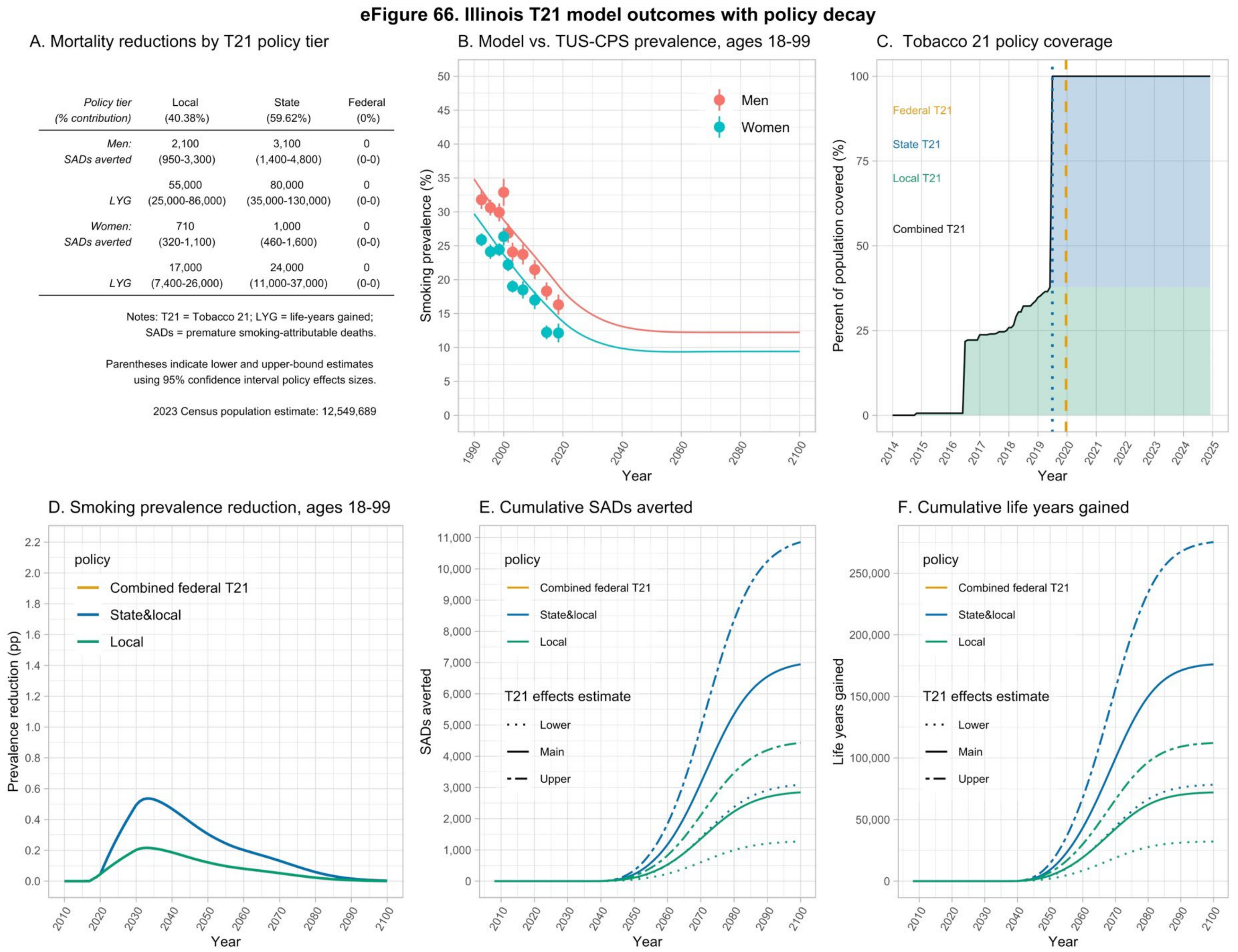

© 2025 Tam J et al. JAMA Health Forum.

© 2025 Tam J et al. JAMA Health Forum.

eFigure 67. Indiana T21 model outcomes with policy decay

eFigure 67. Indiana T21 model outcomes with policy decay

A. Mortality reductions by T21 policy tier

Policy tier (% contribution)	Local (0%)	State (92.72%)	Federal (7.28%)
Men: SADs averted	0 (0-0)	4,200 (1,900-6,600)	330 (140-520)
LYG	0 (0-0)	89,000 (40,000-140,000)	6,900 (3,100-11,000)
Women: SADs averted	0 (0-0)	1,700 (740-2,600)	120 (55-190)
LYG	0 (0-0)	31,000 (14,000-49,000)	2,300 (1,000-3,600)

Notes: T21 = Tobacco 21; LYG = life-years gained; SADs = premature smoking-attributable deaths.

Parentheses indicate lower and upper-bound estimates using 95% confidence interval policy effects sizes.

2023 Census population estimate: 6,862,199

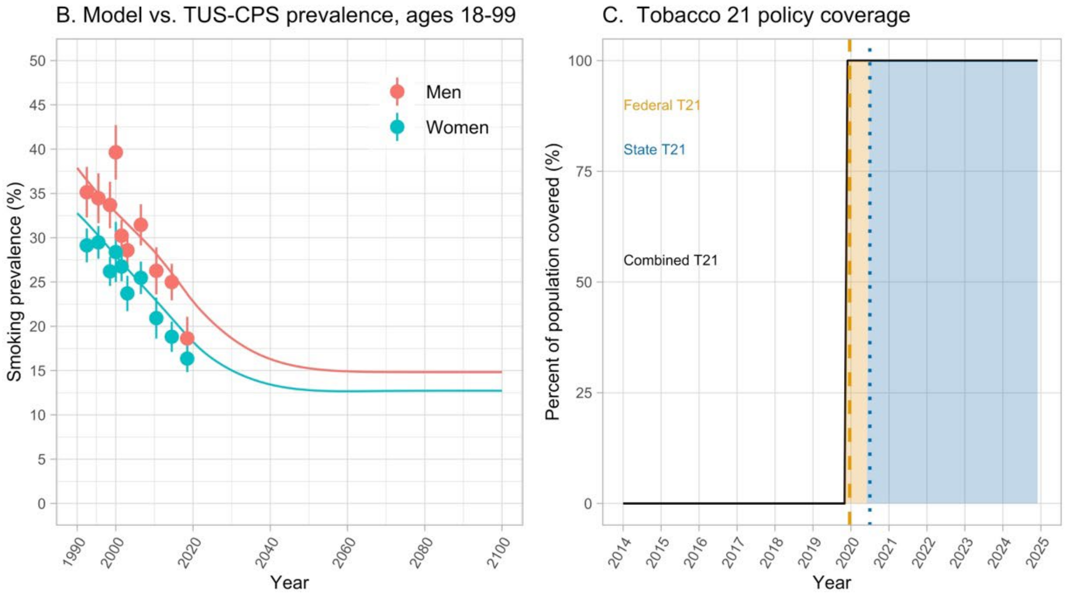

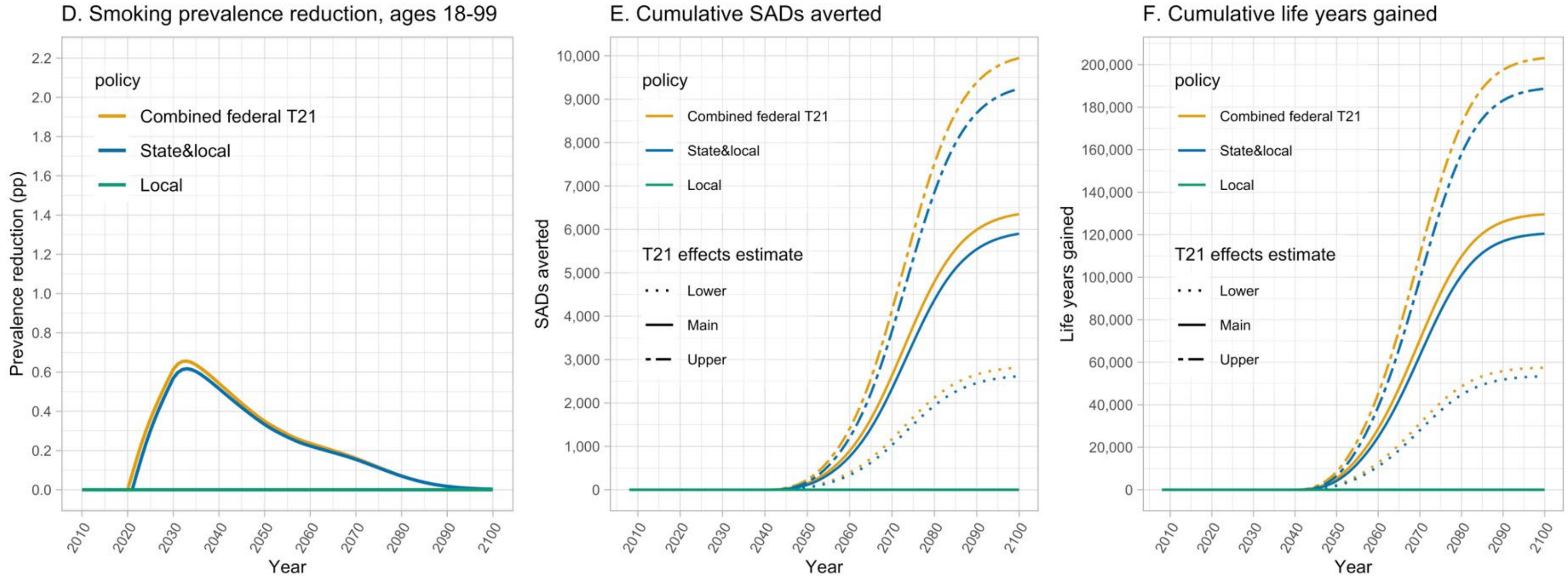

© 2025 Tam J et al. JAMA Health Forum.

© 2025 Tam J et al. JAMA Health Forum.

eFigure 68. Iowa T21 model outcomes with policy decay

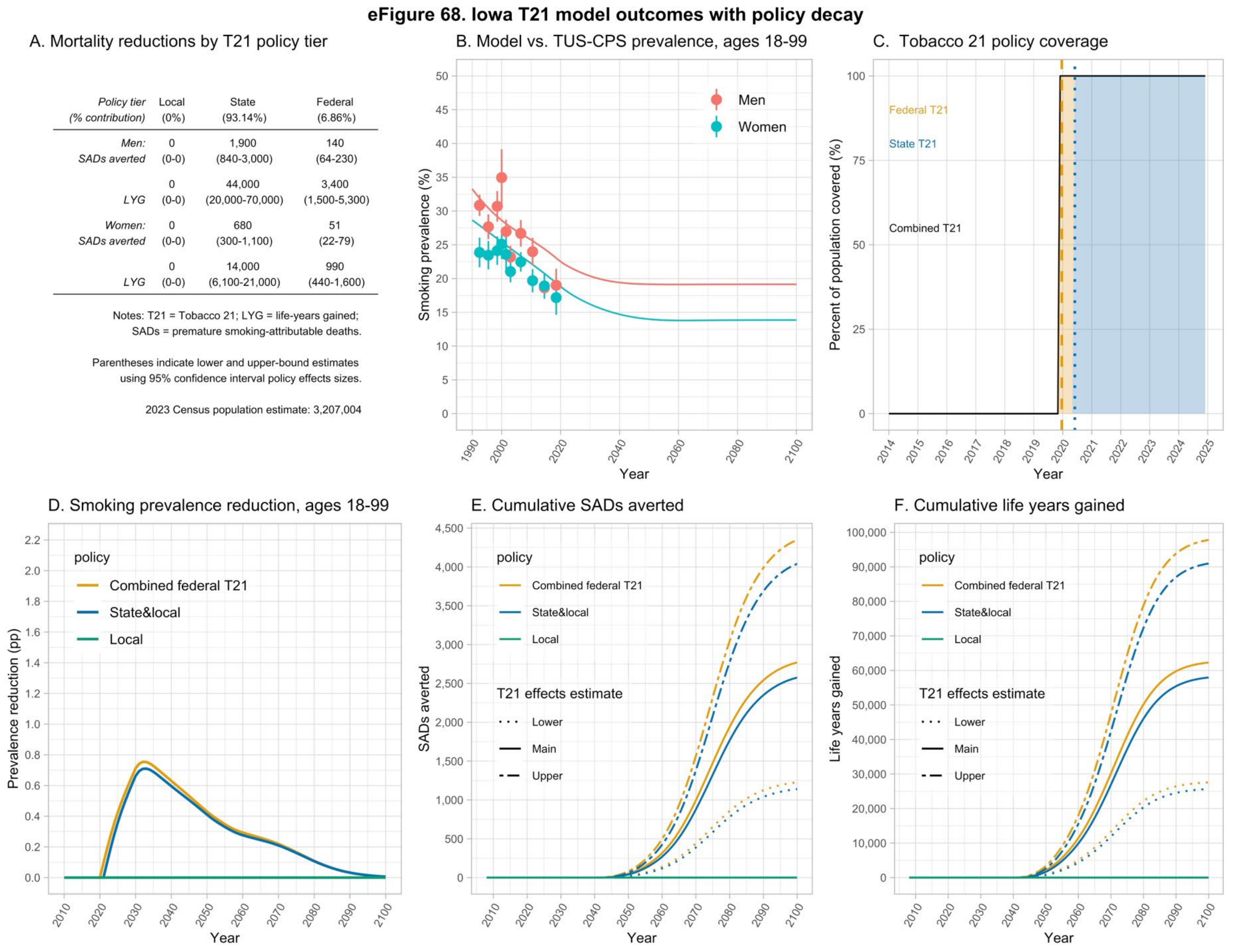

© 2025 Tam J et al. JAMA Health Forum.

© 2025 Tam J et al. JAMA Health Forum.

eFigure 69. Kansas T21 model outcomes with policy decay

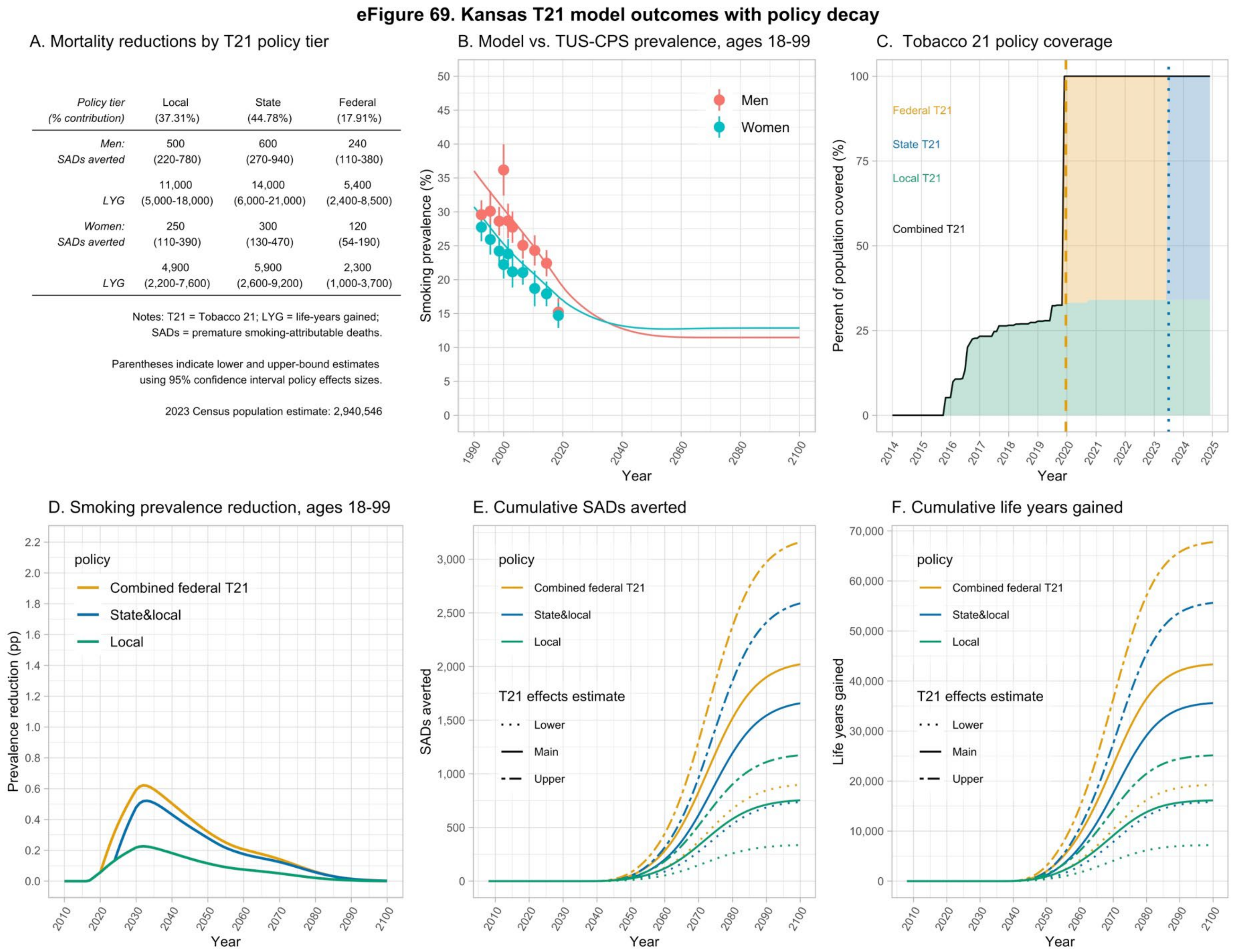

© 2025 Tam J et al. JAMA Health Forum.

© 2025 Tam J et al. JAMA Health Forum.

eFigure 70. Kentucky T21 model outcomes with policy decay

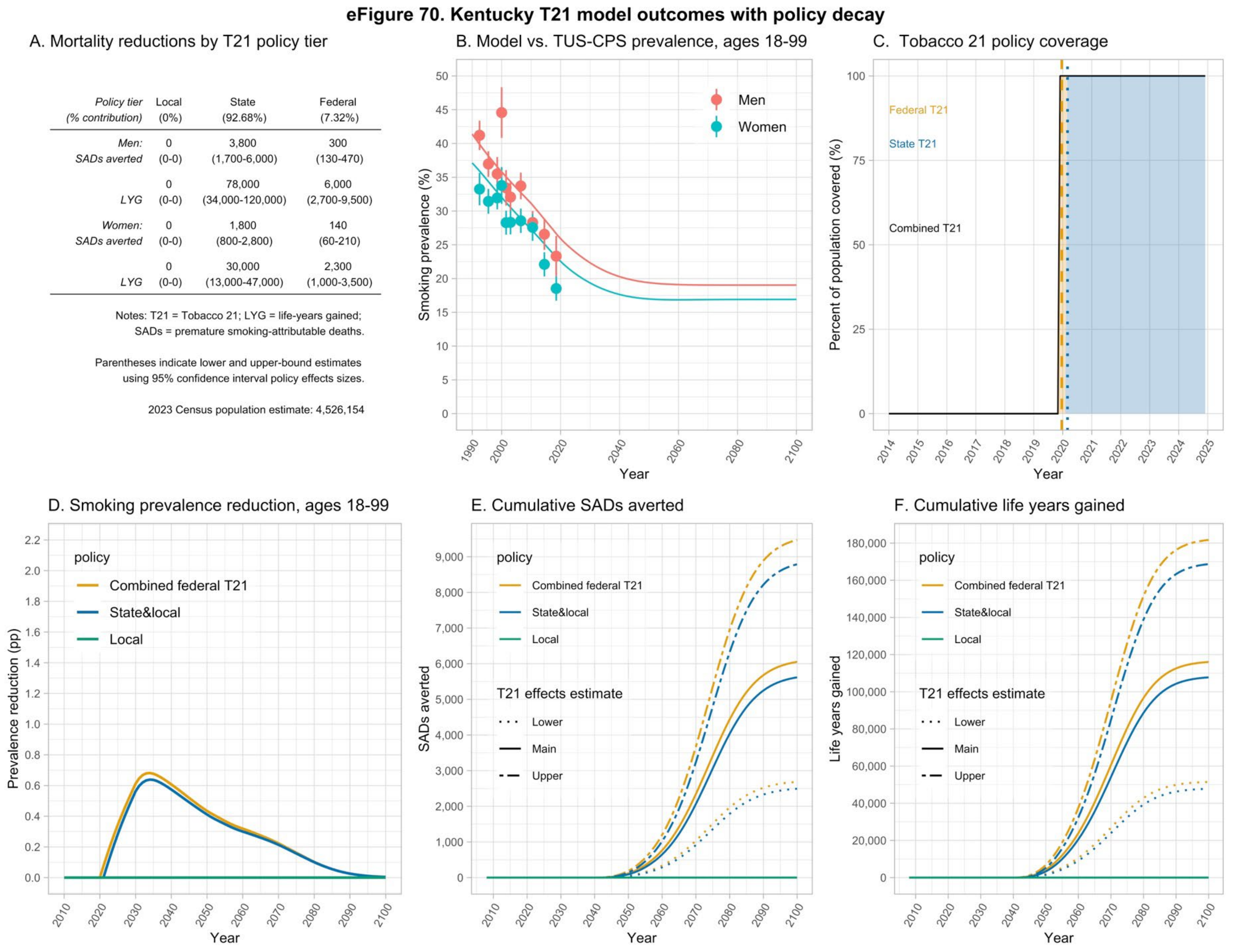

© 2025 Tam J et al. JAMA Health Forum.

© 2025 Tam J et al. JAMA Health Forum.

eFigure 71. Louisiana T21 model outcomes with policy decay

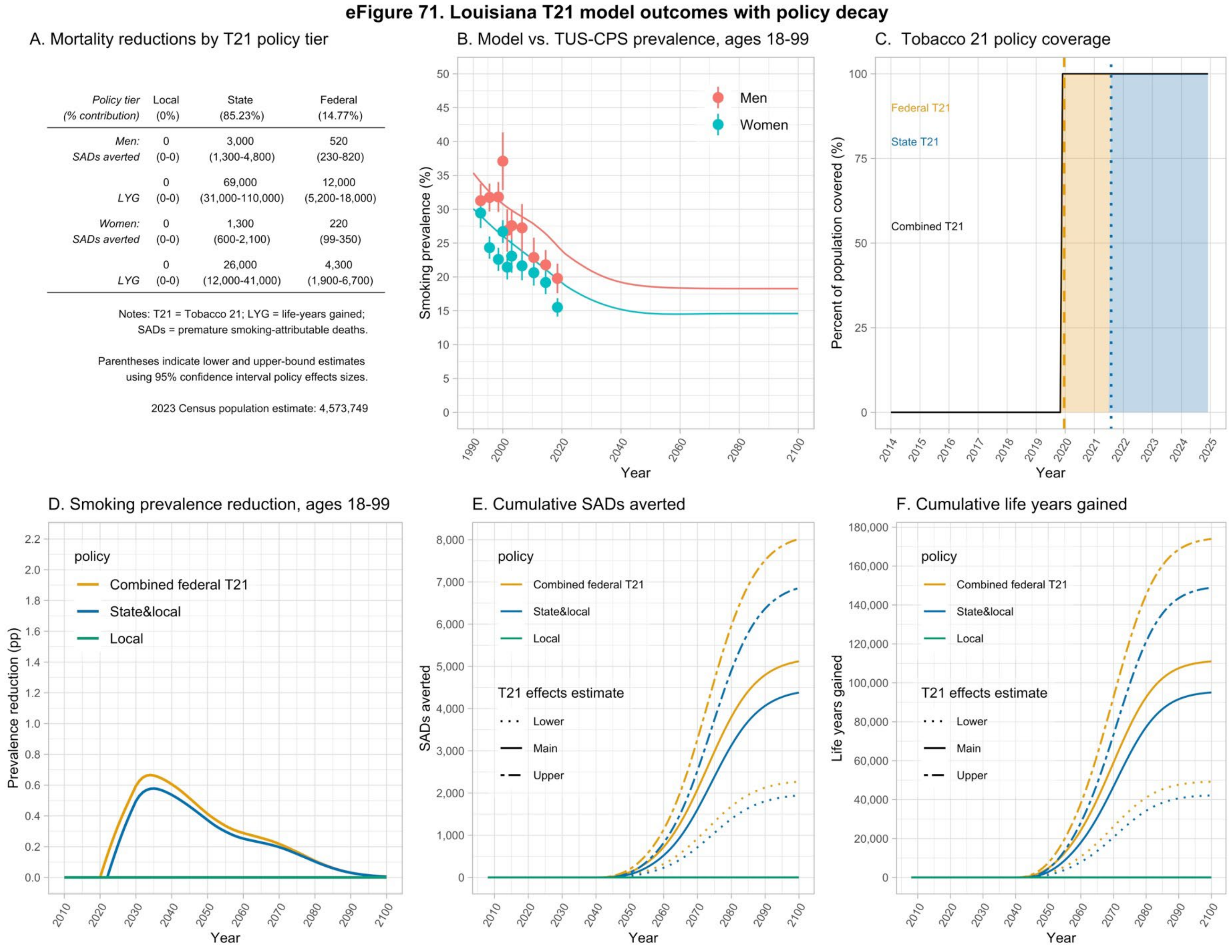

© 2025 Tam J et al. JAMA Health Forum.

© 2025 Tam J et al. JAMA Health Forum.

eFigure 72. Maine T21 model outcomes with policy decay

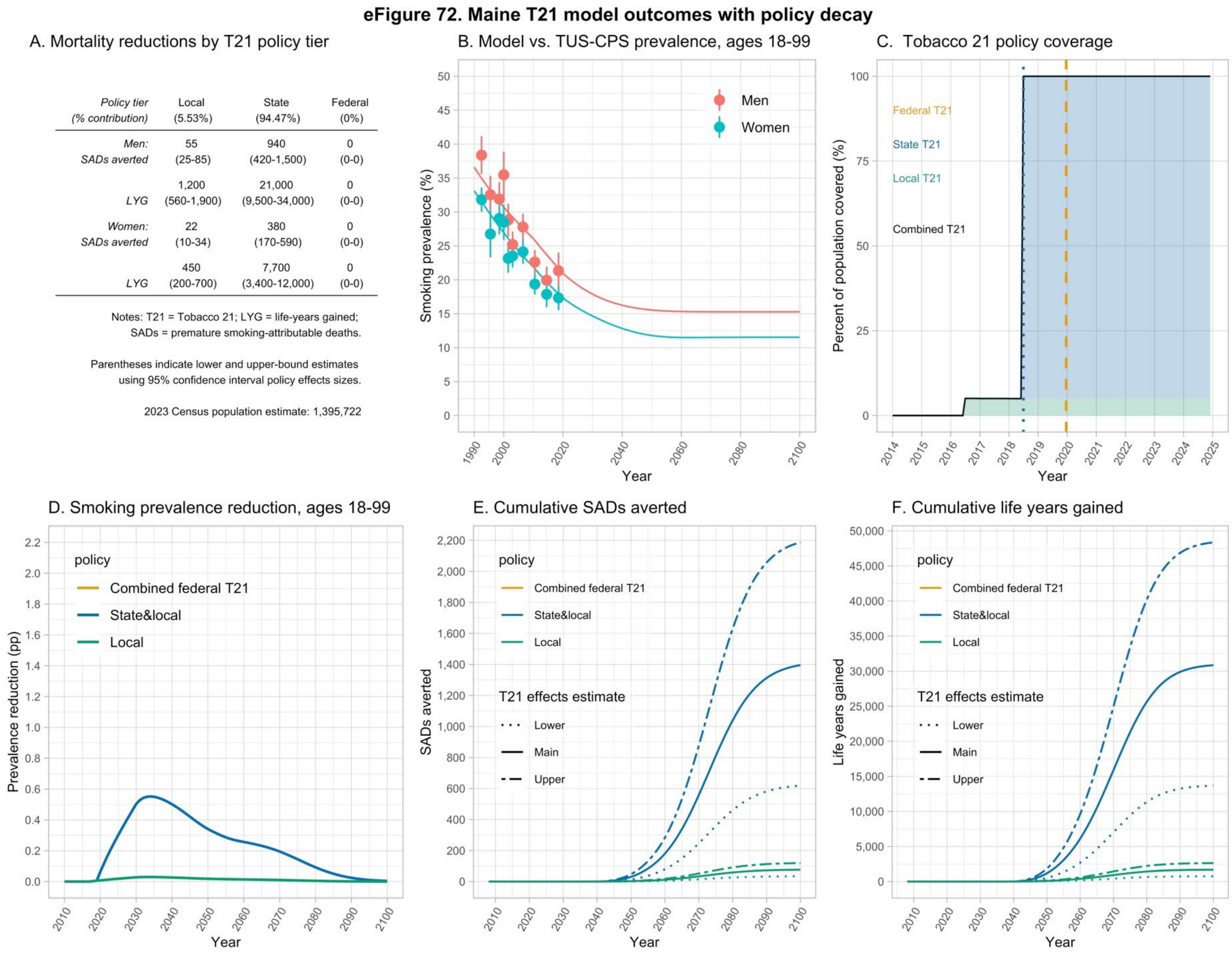

Policy tier (% contribution)	Local (5.53%)	State (94.47%)	Federal (0%)
Men: SADs averted	55 (25-85)	940 (420-1,500)	0 (0-0)
LYG	1,200 (560-1,900)	21,000 (9,500-34,000)	0 (0-0)
Women: SADs averted	22 (10-34)	380 (170-590)	0 (0-0)
LYG	450 (200-700)	7,700 (3,400-12,000)	0 (0-0)

Notes: T21 = Tobacco 21; LYG = life-years gained; SADs = premature smoking-attributable deaths.

Parentheses indicate lower and upper-bound estimates using 95% confidence interval policy effects sizes.

2023 Census population estimate: 1,395,722

© 2025 Tam J et al. JAMA Health Forum.

© 2025 Tam J et al. JAMA Health Forum.

eFigure 73. Maryland T21 model outcomes with policy decay

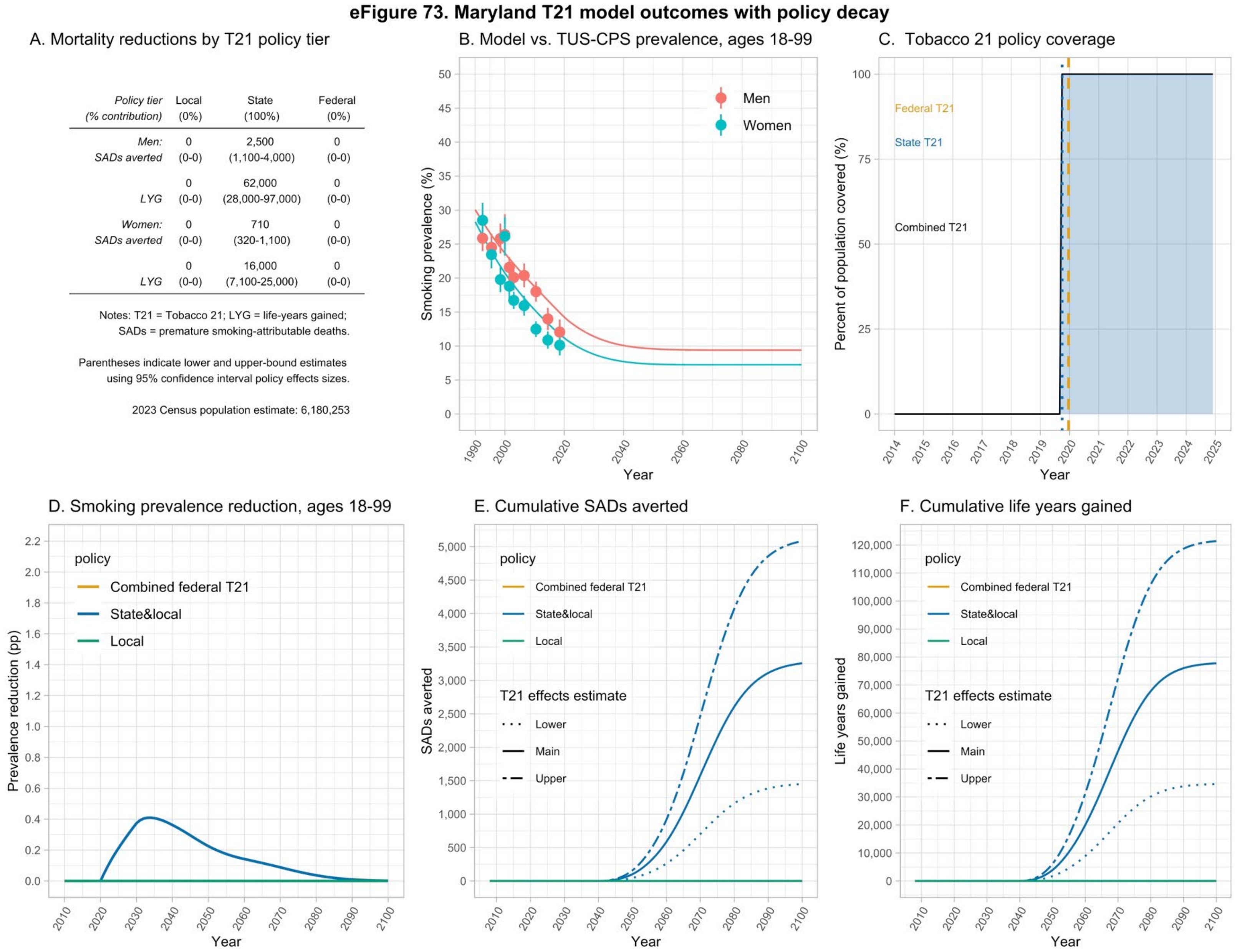

© 2025 Tam J et al. JAMA Health Forum.

© 2025 Tam J et al. JAMA Health Forum.

eFigure 74. Massachusetts T21 model outcomes with policy decay

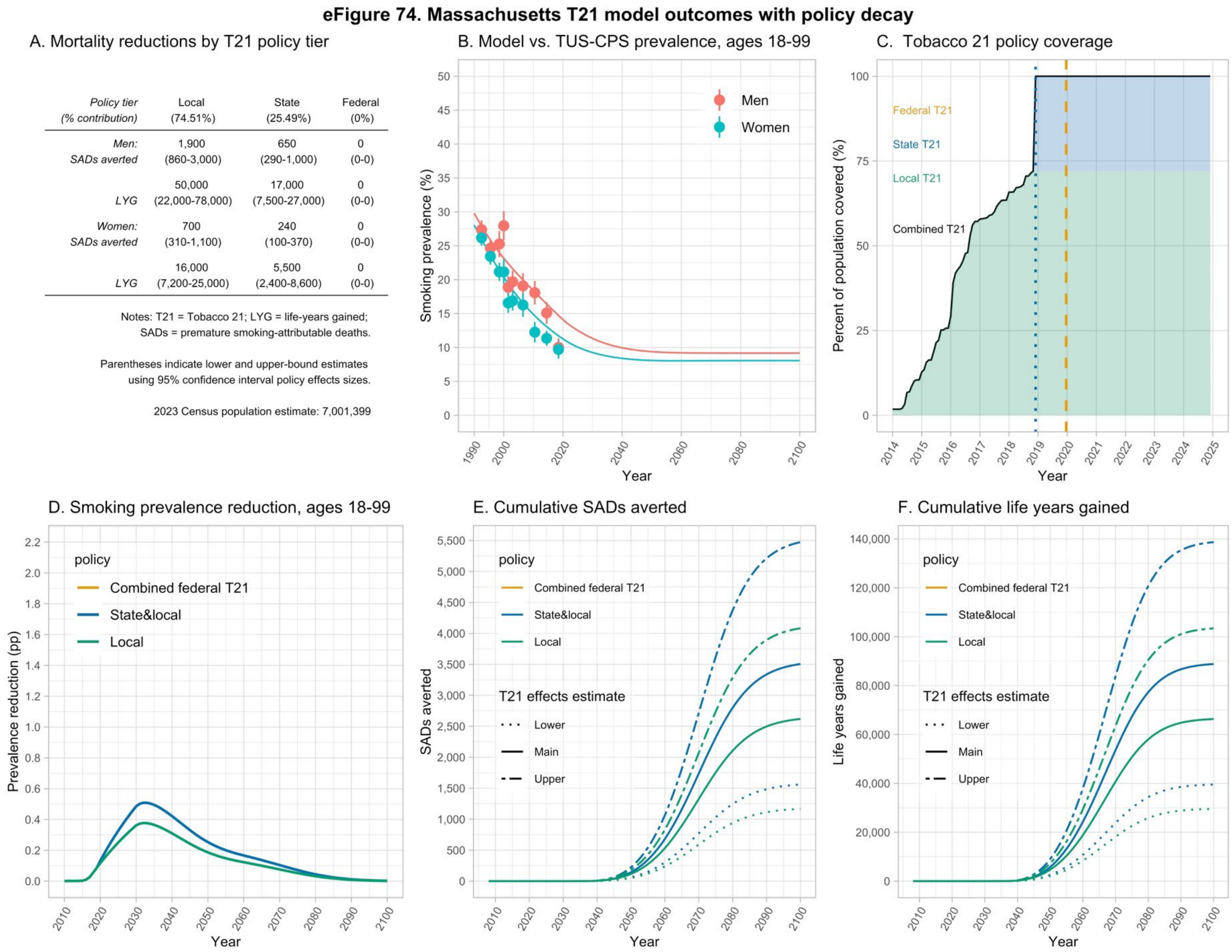

© 2025 Tam J et al. JAMA Health Forum.

© 2025 Tam J et al. JAMA Health Forum.

eFigure 75. Michigan T21 model outcomes with policy decay

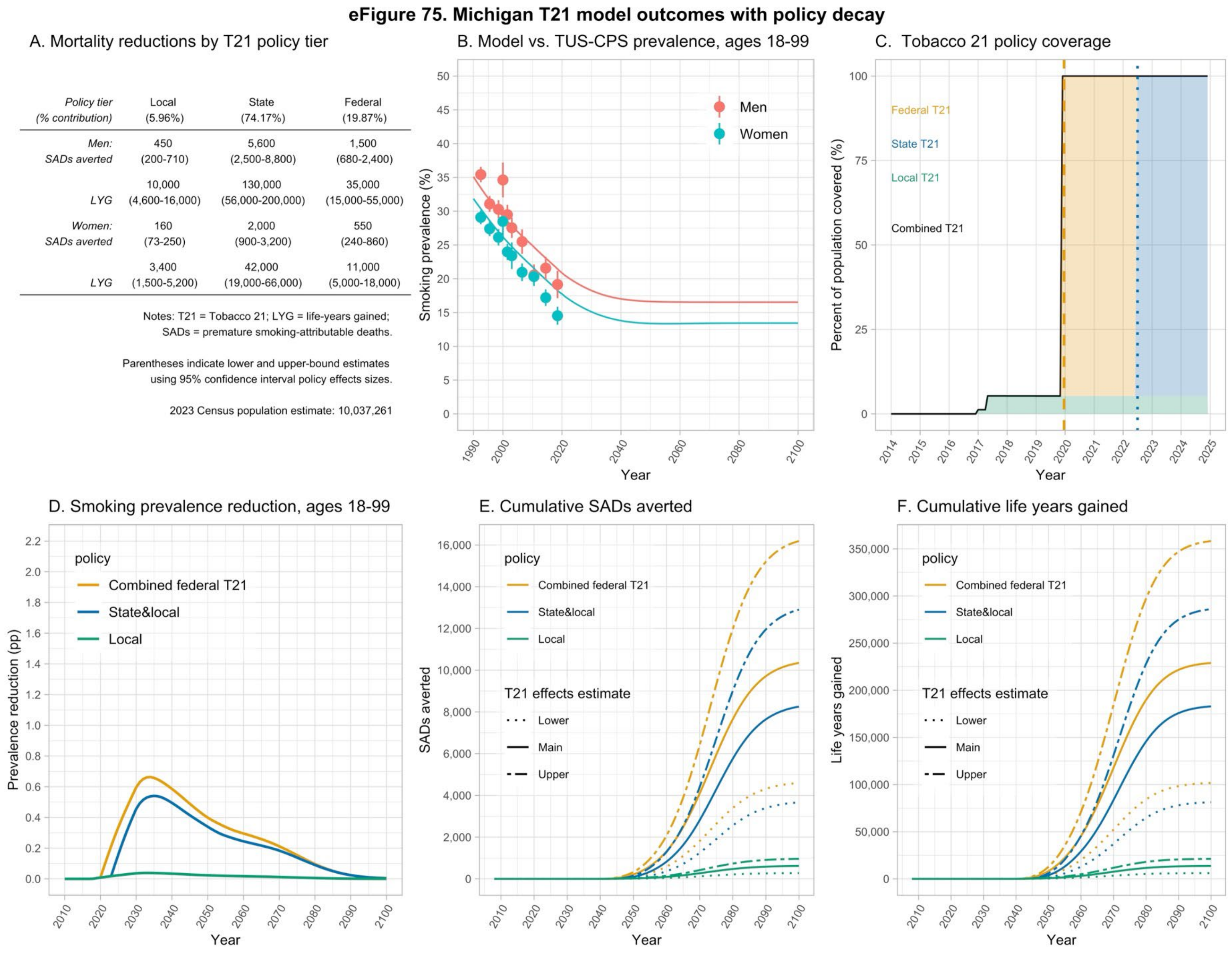

© 2025 Tam J et al. JAMA Health Forum.

© 2025 Tam J et al. JAMA Health Forum.

eFigure 76. Minnesota T21 model outcomes with policy decay

eFigure 76. Minnesota T21 model outcomes with policy decay

A. Mortality reductions by T21 policy tier

Policy tier (% contribution)	Local (50.17%)	State (45.99%)	Federal (3.85%)
Men: SADs averted	1,200 (540-1,900)	1,100 (470-1,700)	92 (40-140)
LYG	32,000 (14,000-49,000)	28,000 (12,000-44,000)	2,400 (1,000-3,700)
Women: SADs averted	350 (160-550)	310 (140-480)	26 (11-40)
LYG	8,100 (3,600-13,000)	7,100 (3,200-11,000)	590 (260-920)

Notes: T21 = Tobacco 21; LYG = life-years gained; SADs = premature smoking-attributable deaths.

Parentheses indicate lower and upper-bound estimates using 95% confidence interval policy effects sizes.

2023 Census population estimate: 5,737,915

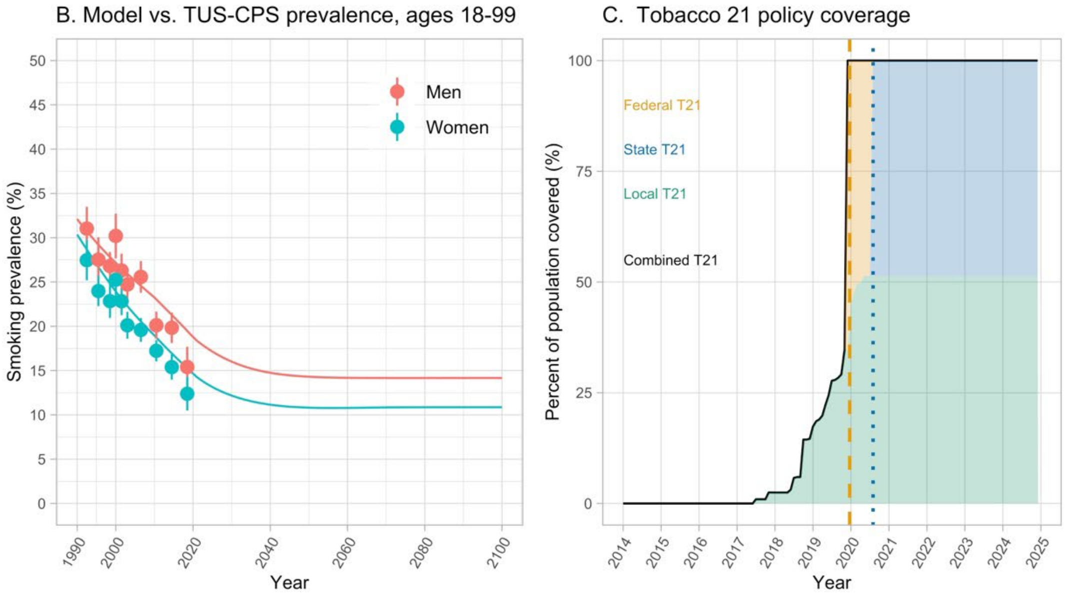

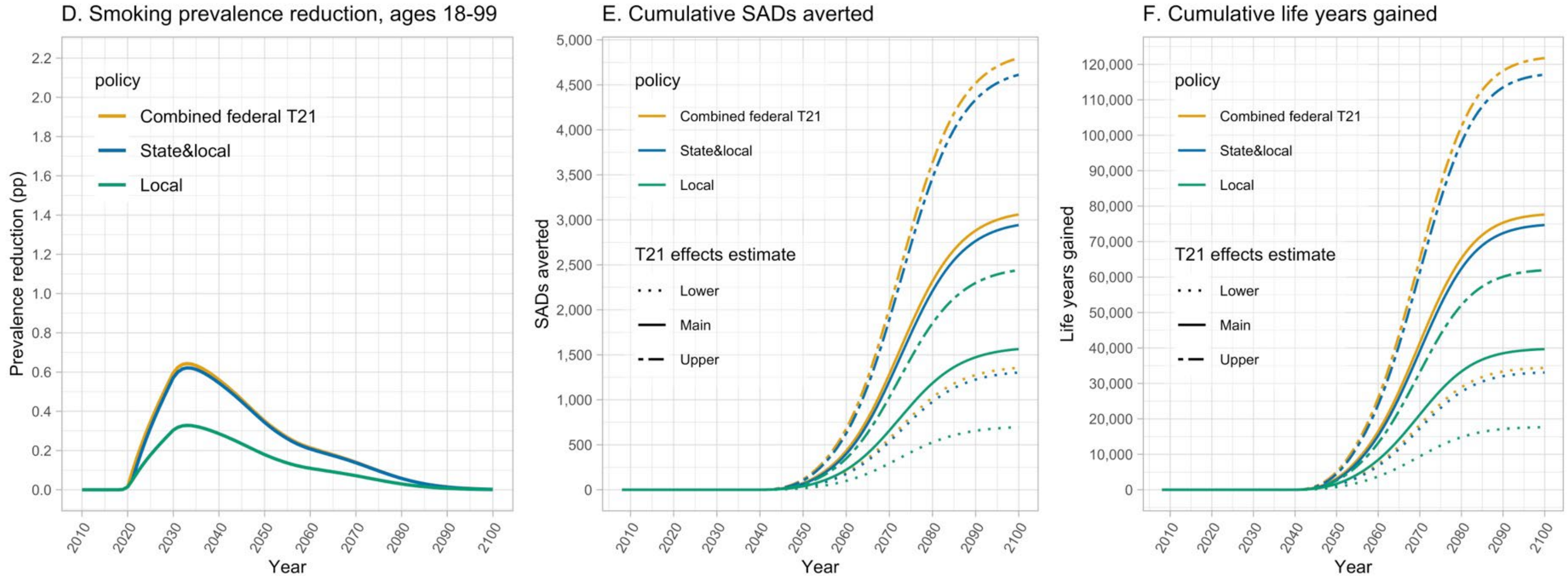

© 2025 Tam J et al. JAMA Health Forum.

© 2025 Tam J et al. JAMA Health Forum.

eFigure 77. Mississippi T21 model outcomes with policy decay

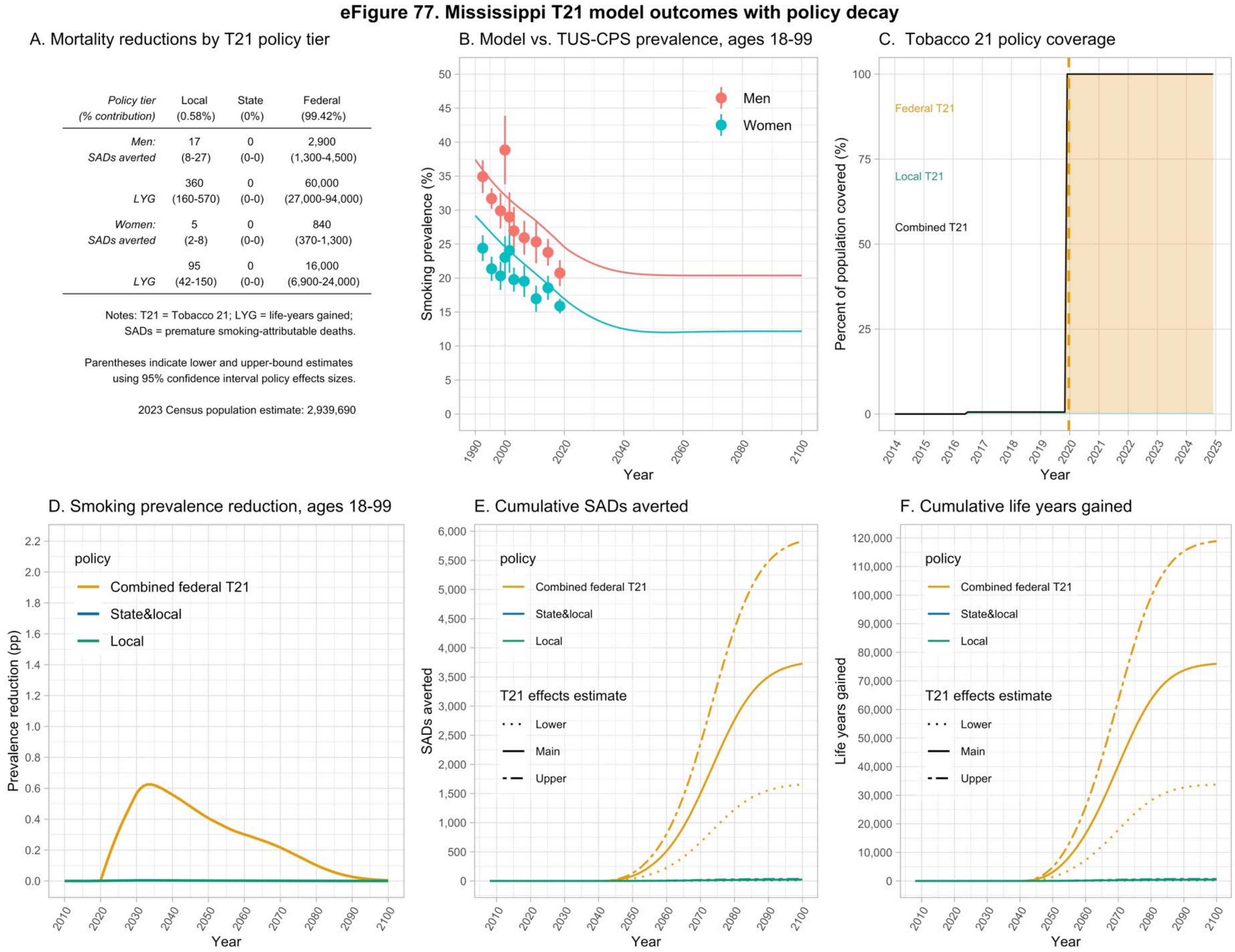

© 2025 Tam J et al. JAMA Health Forum.

© 2025 Tam J et al. JAMA Health Forum.

eFigure 78. Missouri T21 model outcomes with policy decay

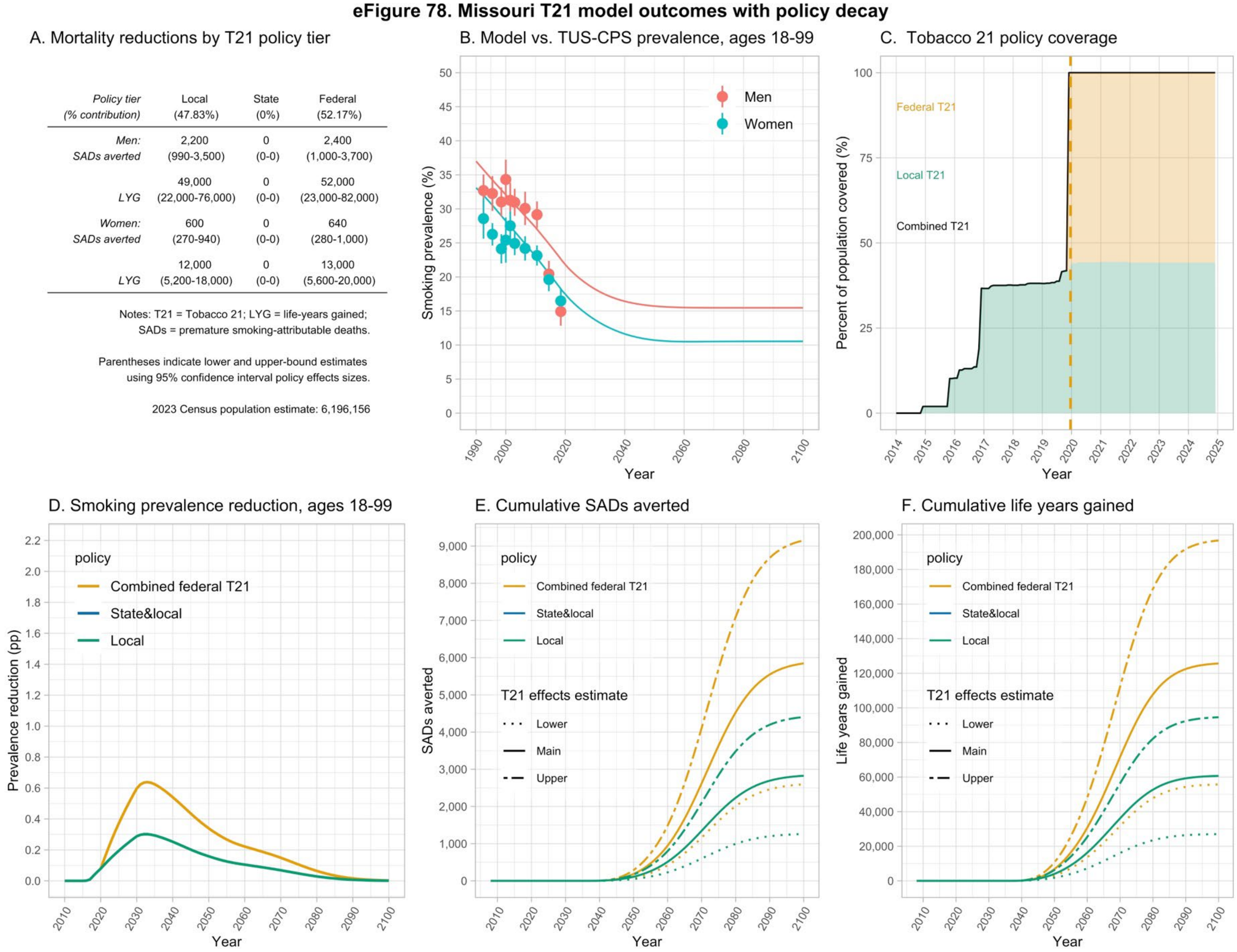

© 2025 Tam J et al. JAMA Health Forum.

© 2025 Tam J et al. JAMA Health Forum.

eFigure 79. Montana T21 model outcomes with policy decay

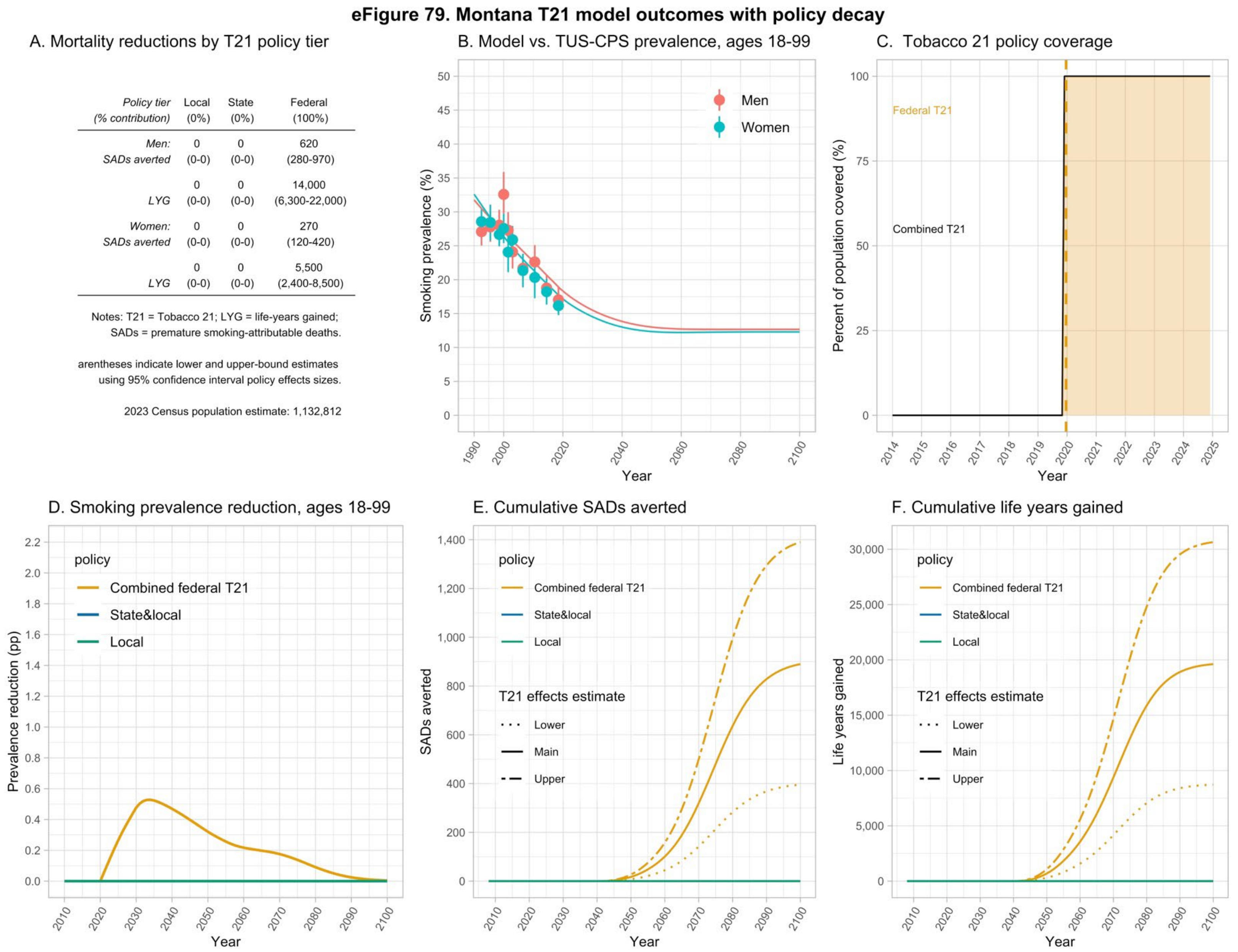

© 2025 Tam J et al. JAMA Health Forum.

© 2025 Tam J et al. JAMA Health Forum.

eFigure 80. Nebraska T21 model outcomes with policy decay

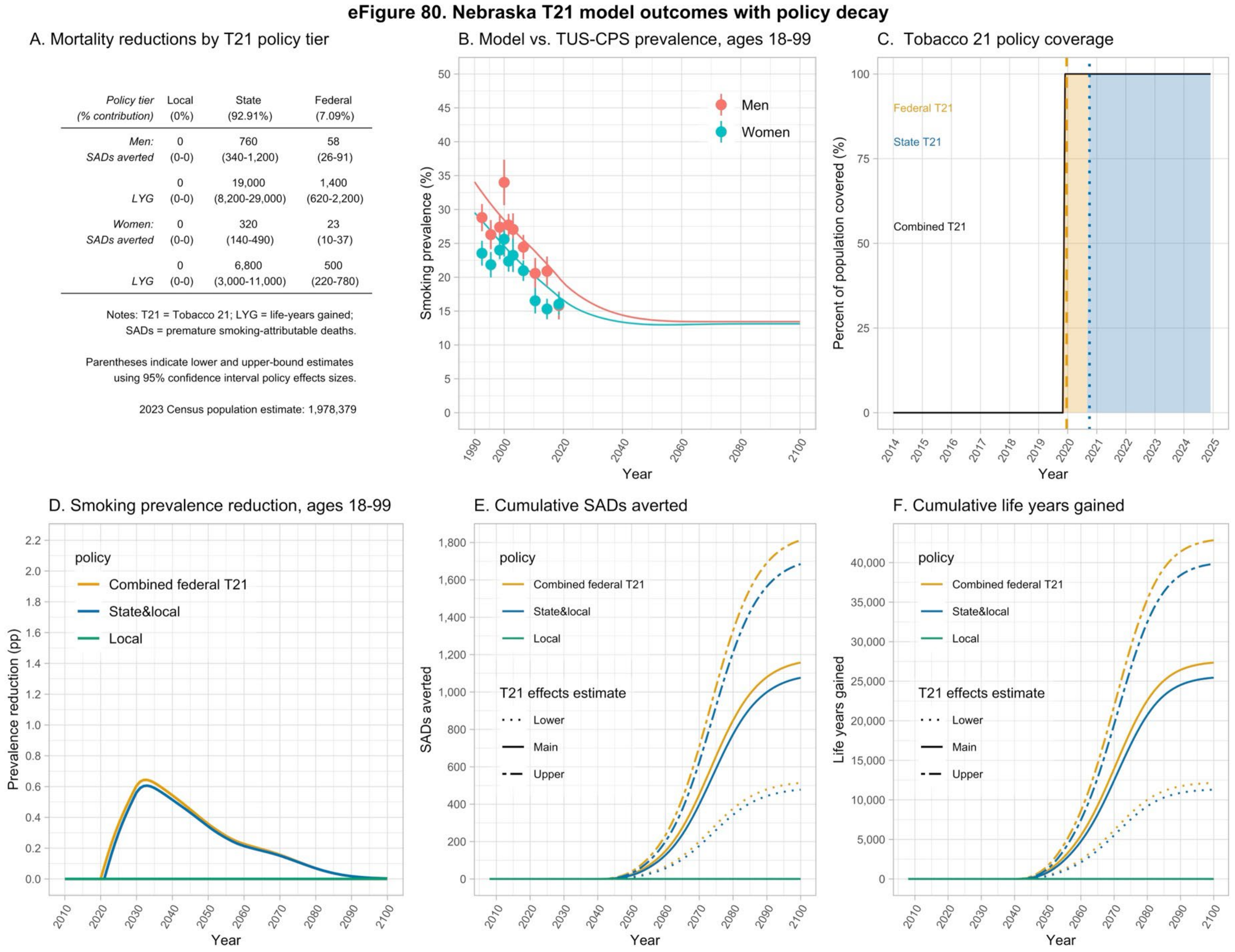

© 2025 Tam J et al. JAMA Health Forum.

© 2025 Tam J et al. JAMA Health Forum.

eFigure 81. Nevada T21 model outcomes with policy decay

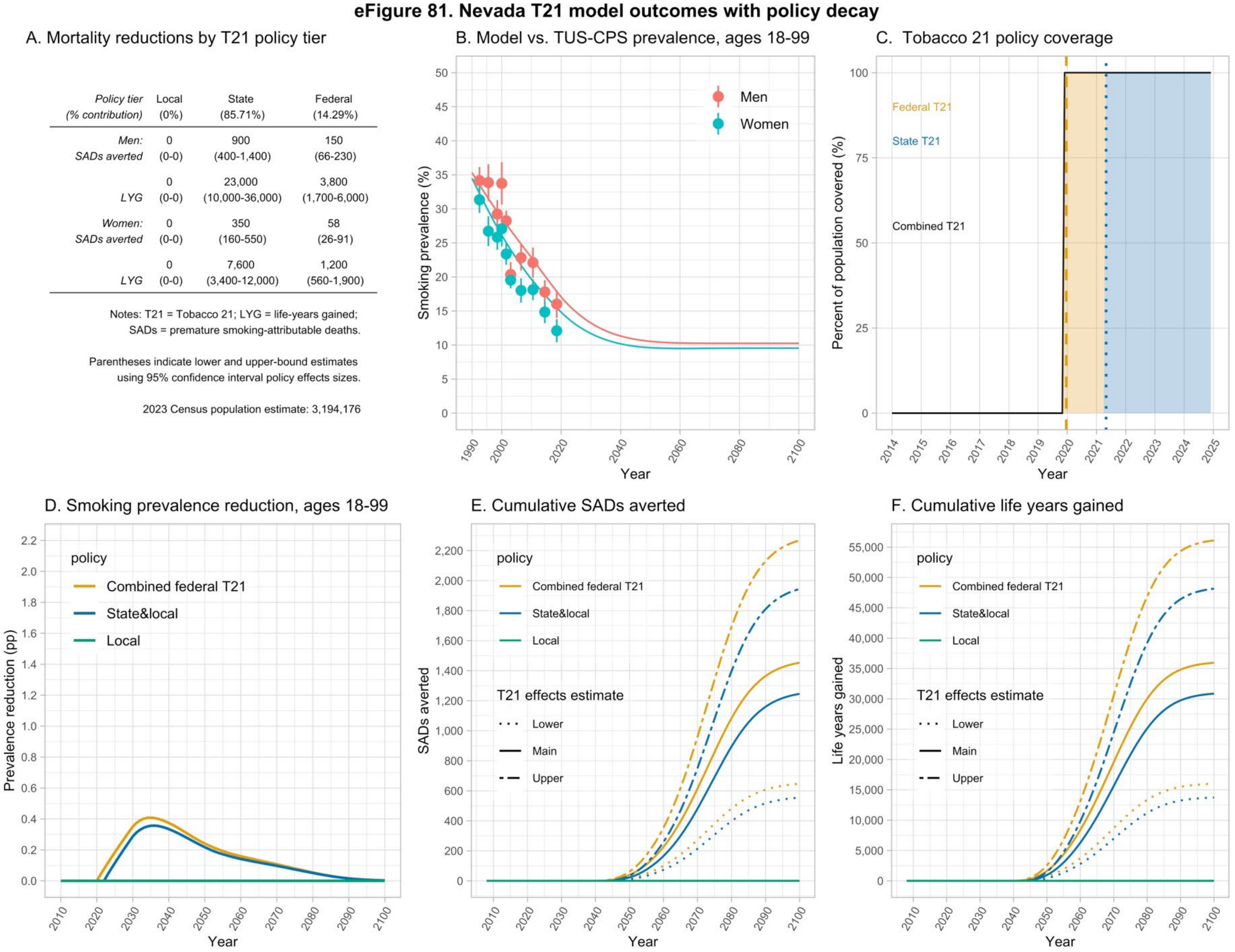

© 2025 Tam J et al. JAMA Health Forum.

© 2025 Tam J et al. JAMA Health Forum.

eFigure 82. New Hampshire T21 model outcomes with policy decay

eFigure 82. New Hampshire T21 model outcomes with policy decay

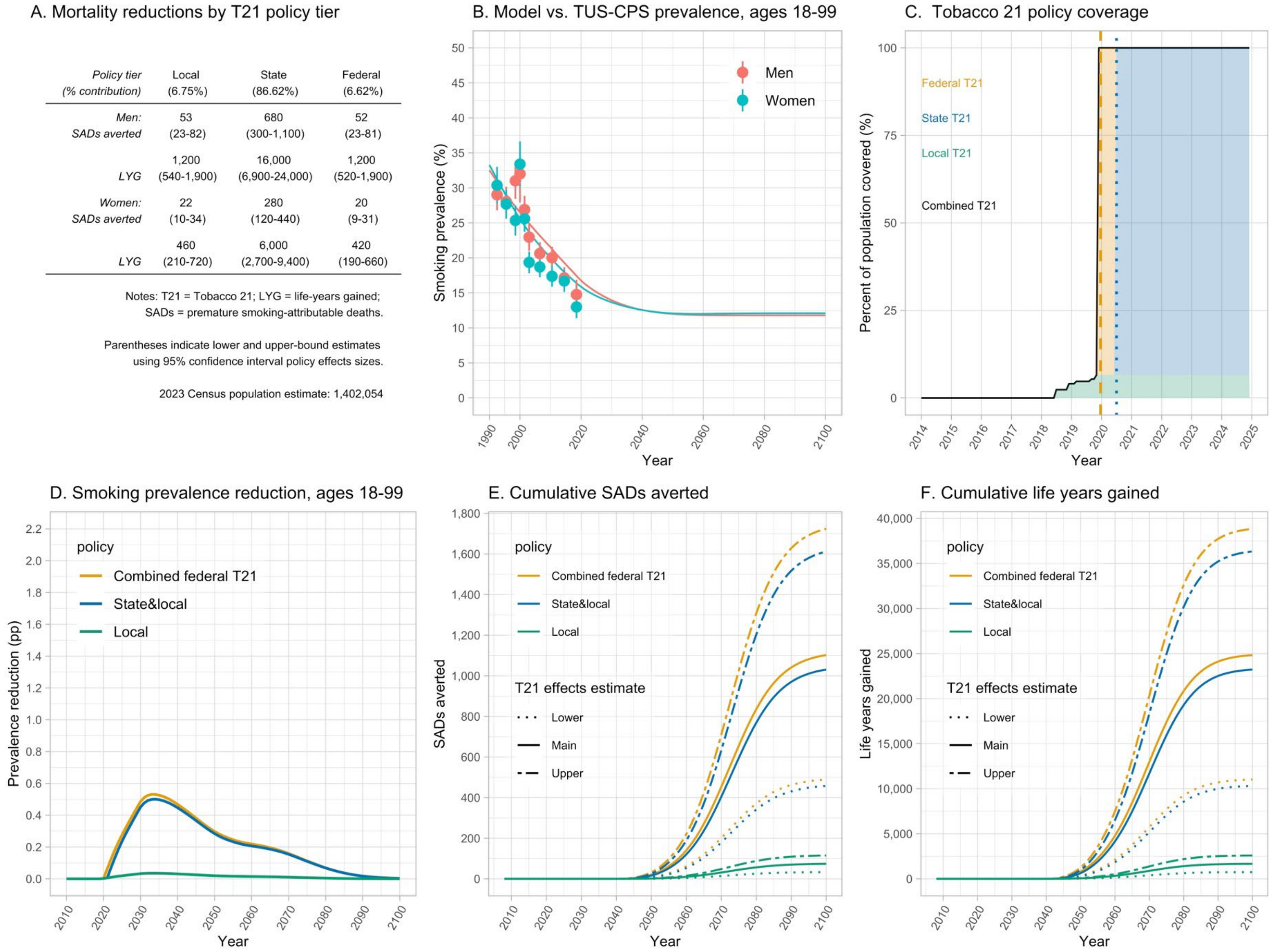

Policy tier (% contribution)	Local (6.75%)	State (86.62%)	Federal (6.62%)
Men: SADs averted	53 (23-82)	680 (300-1,100)	52 (23-81)
LYG	1,200 (540-1,900)	16,000 (6,900-24,000)	1,200 (520-1,900)
Women: SADs averted	22 (10-34)	280 (120-440)	20 (9-31)
LYG	460 (210-720)	6,000 (2,700-9,400)	420 (190-660)

Notes: T21 = Tobacco 21; LYG = life-years gained; SADs = premature smoking-attributable deaths.

Parentheses indicate lower and upper-bound estimates using 95% confidence interval policy effects sizes.

2023 Census population estimate: 1,402,054

© 2025 Tam J et al. JAMA Health Forum.

© 2025 Tam J et al. JAMA Health Forum.

eFigure 83. New Jersey T21 model outcomes with policy decay

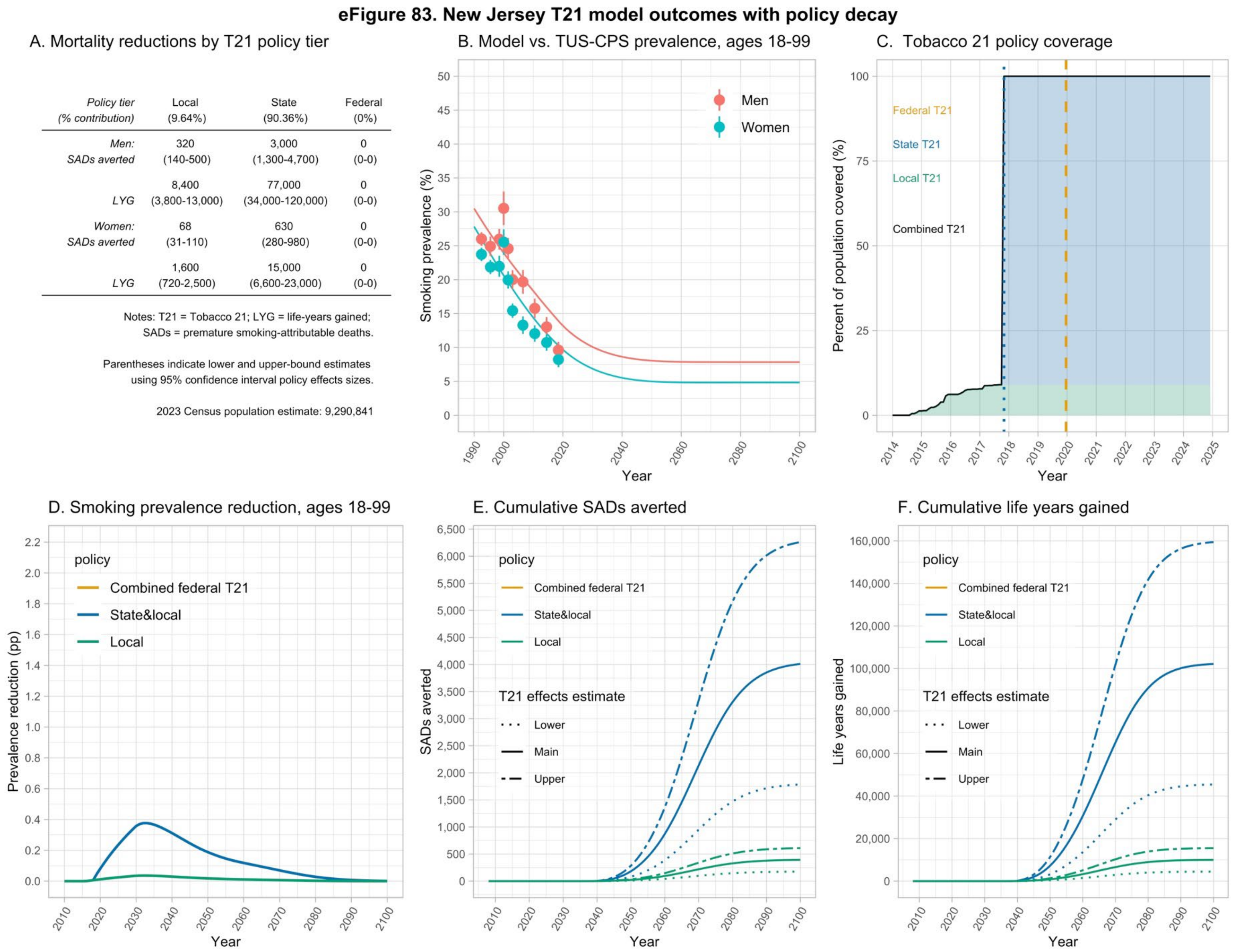

© 2025 Tam J et al. JAMA Health Forum.

© 2025 Tam J et al. JAMA Health Forum.

eFigure 84. New Mexico T21 model outcomes with policy decay

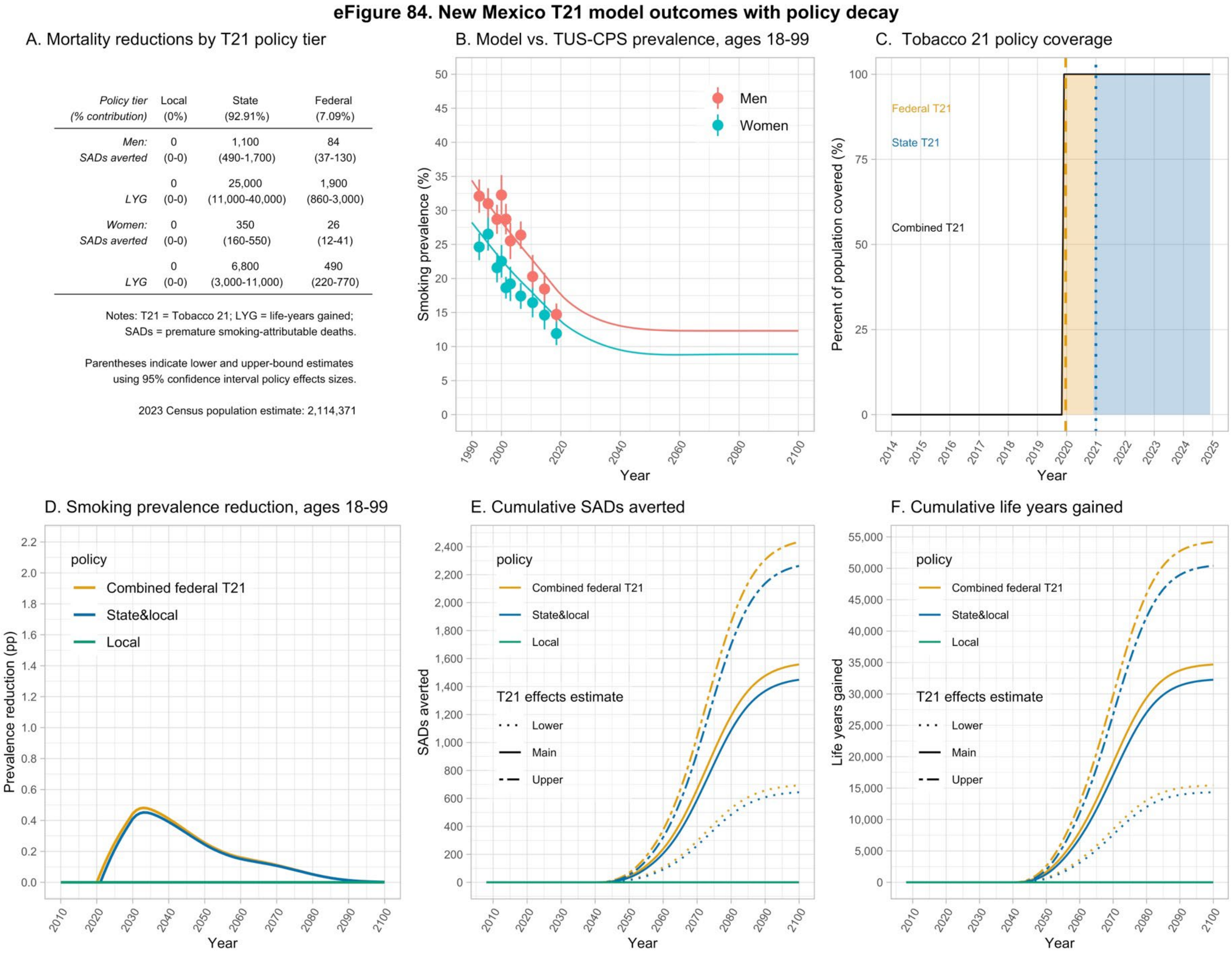

© 2025 Tam J et al. JAMA Health Forum.

© 2025 Tam J et al. JAMA Health Forum.

eFigure 85. New York T21 model outcomes with policy decay

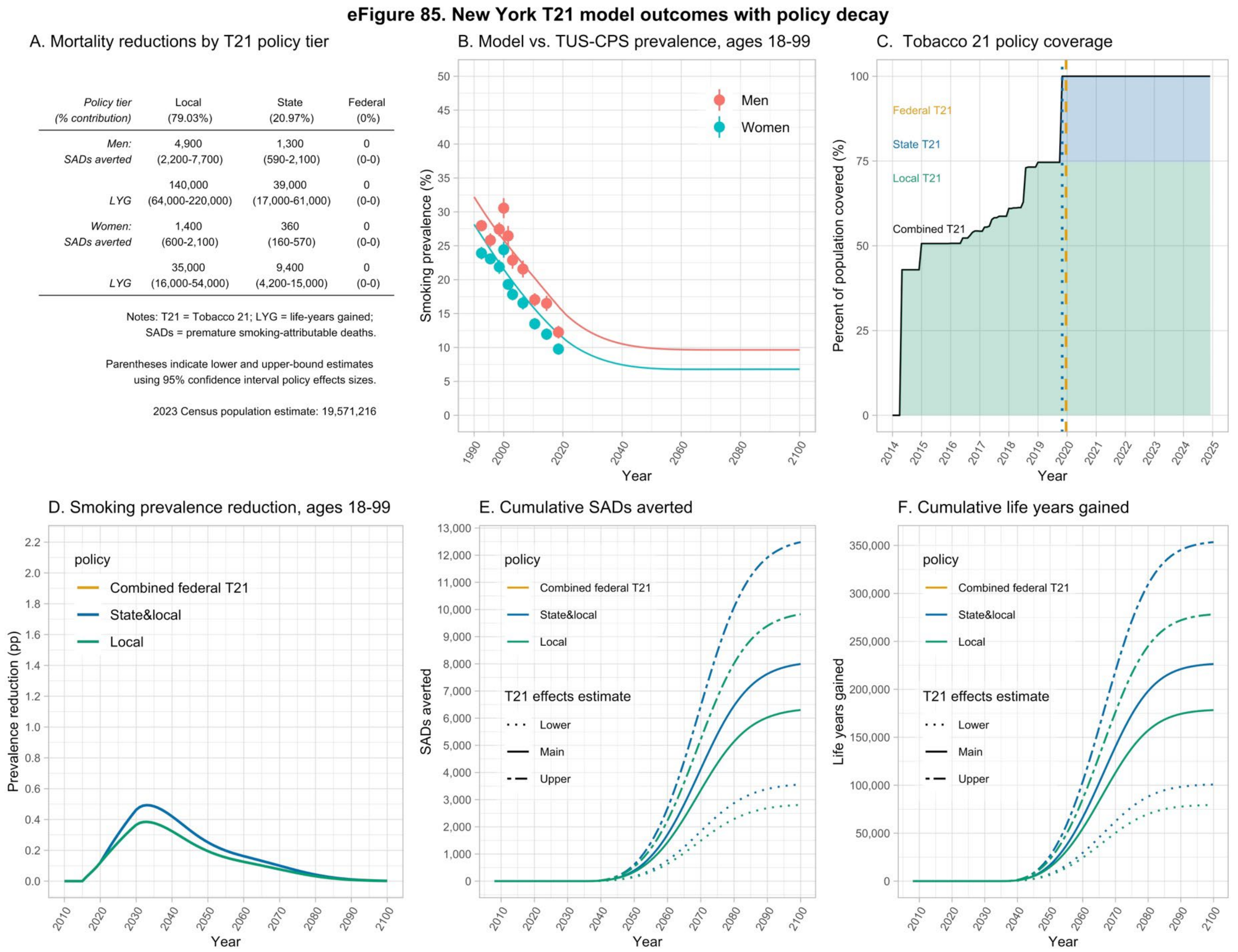

Policy tier (% contribution)	Local (79.03%)	State (20.97%)	Federal (0%)
Men: SADs averted	4,900 (2,200-7,700)	1,300 (590-2,100)	0 (0-0)
LYG	140,000 (64,000-220,000)	39,000 (17,000-61,000)	0 (0-0)
Women: SADs averted	1,400 (600-2,100)	360 (160-570)	0 (0-0)
LYG	35,000 (16,000-54,000)	9,400 (4,200-15,000)	0 (0-0)

Notes: T21 = Tobacco 21; LYG = life-years gained; SADs = premature smoking-attributable deaths.

Parentheses indicate lower and upper-bound estimates using 95% confidence interval policy effects sizes.

2023 Census population estimate: 19,571,216

© 2025 Tam J et al. JAMA Health Forum.

© 2025 Tam J et al. JAMA Health Forum.

eFigure 86. North Carolina T21 model outcomes with policy decay

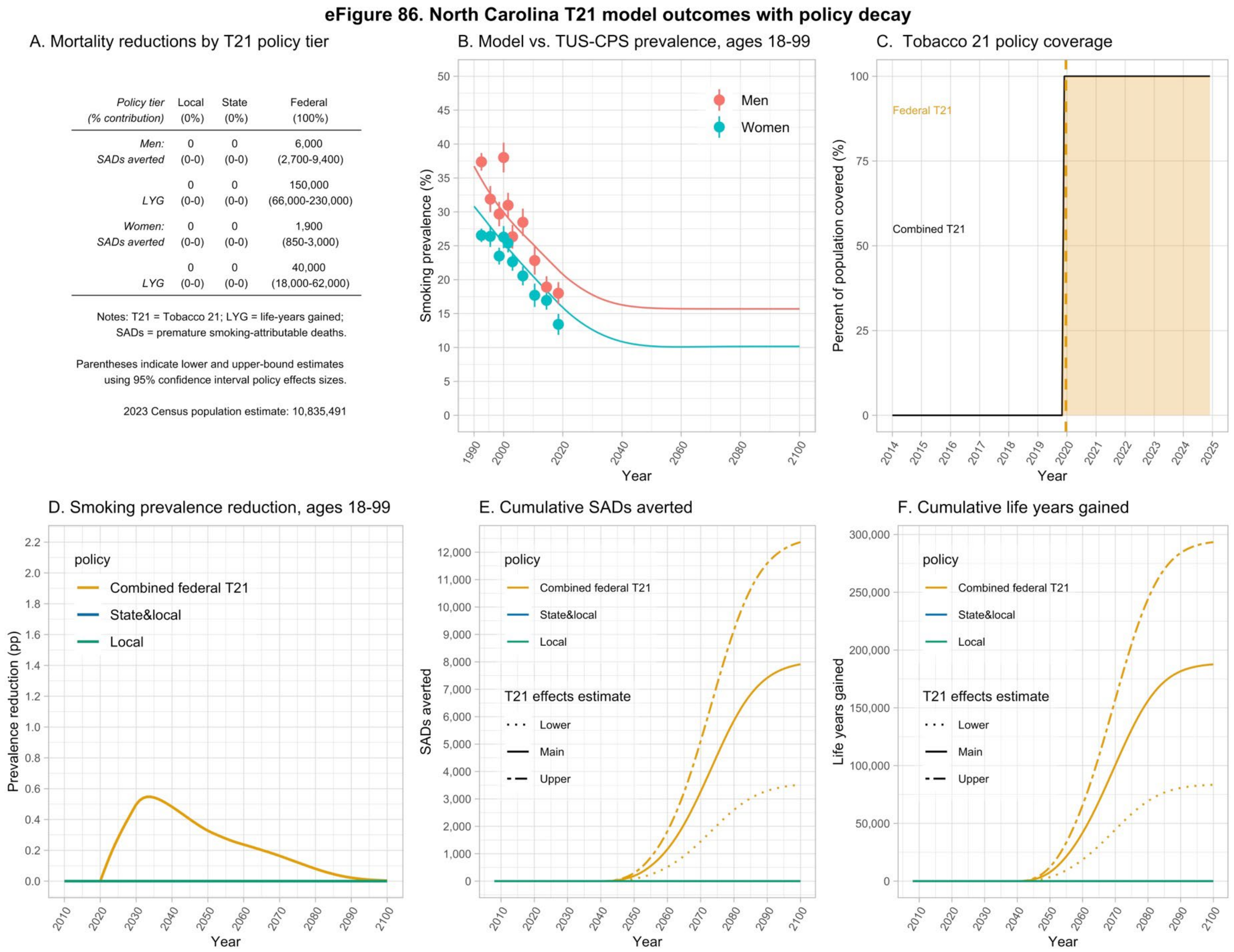

© 2025 Tam J et al. JAMA Health Forum.

© 2025 Tam J et al. JAMA Health Forum.

eFigure 87. North Dakota T21 model outcomes with policy decay

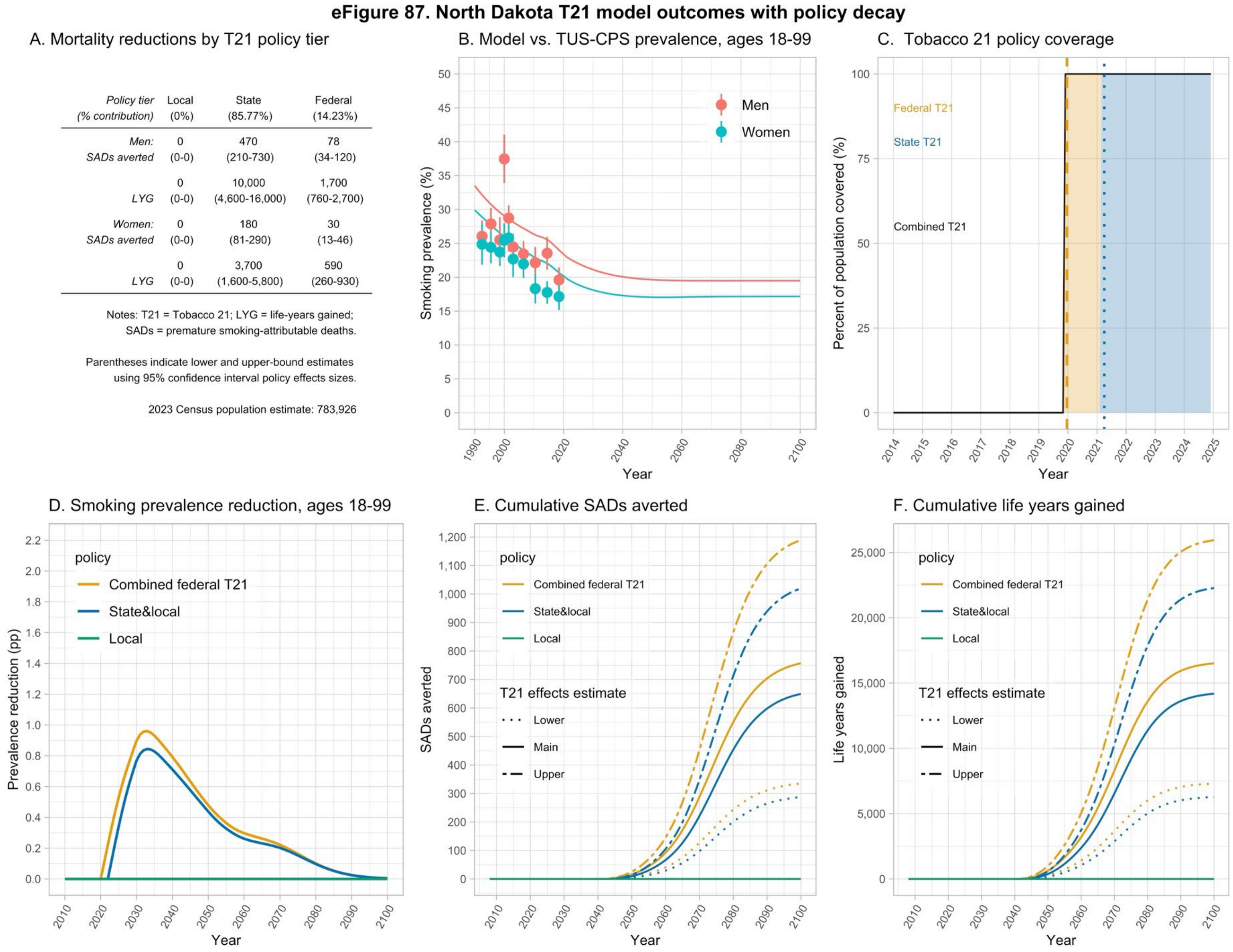

© 2025 Tam J et al. JAMA Health Forum.

© 2025 Tam J et al. JAMA Health Forum.

eFigure 88. Ohio T21 model outcomes with policy decay

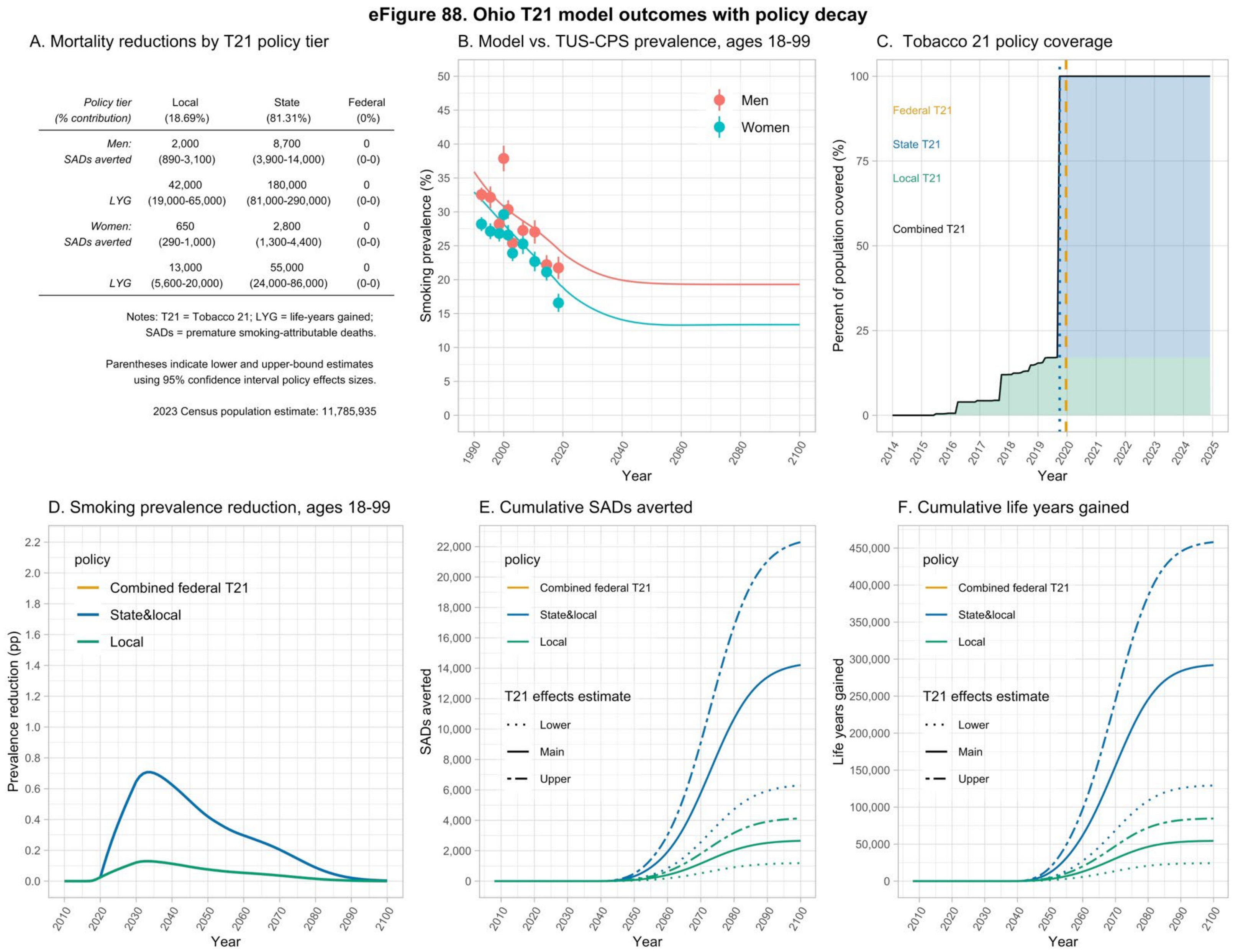

© 2025 Tam J et al. JAMA Health Forum.

© 2025 Tam J et al. JAMA Health Forum.

eFigure 89. Oklahoma T21 model outcomes with policy decay

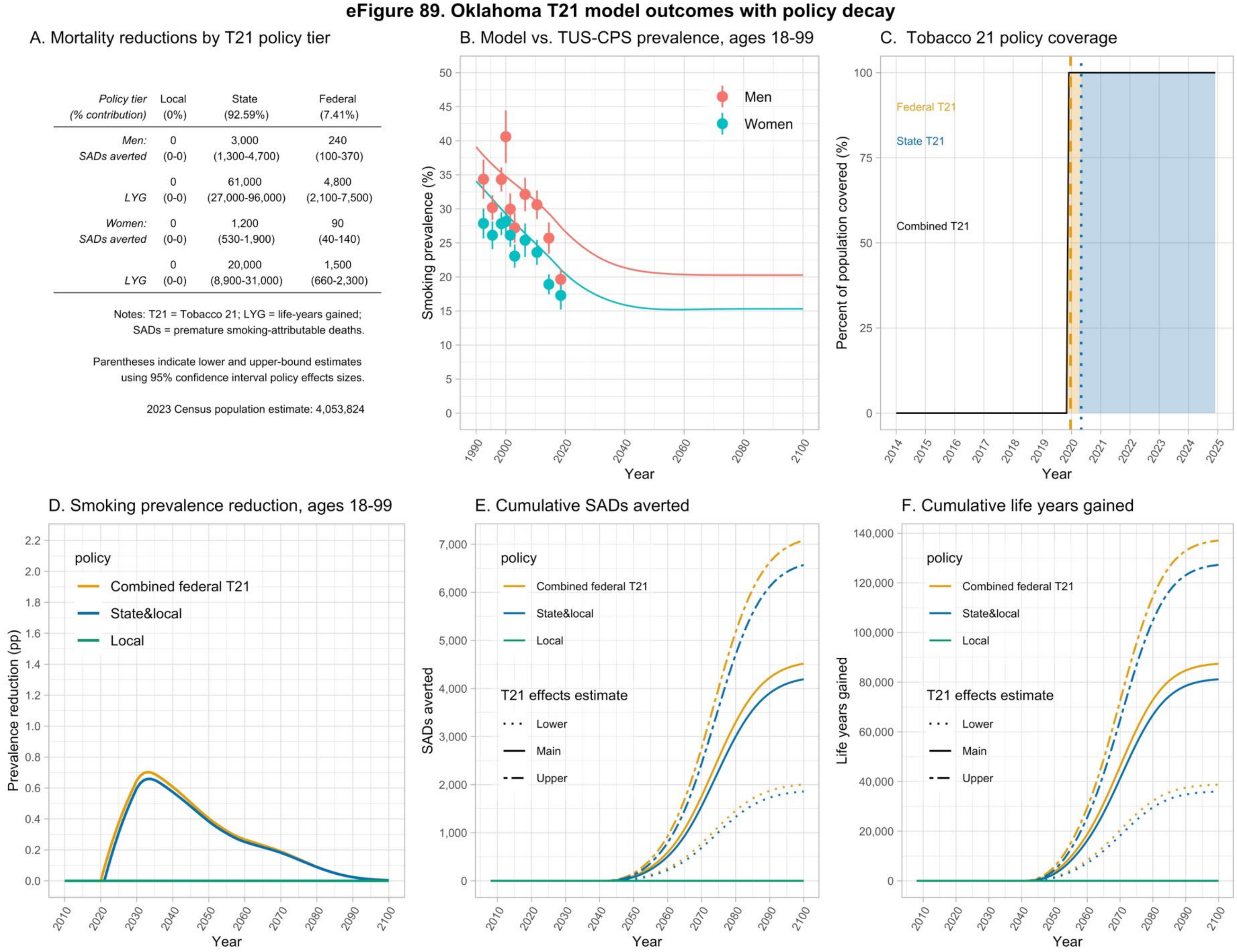

Policy tier (% contribution)	Local (0%)	State (92.59%)	Federal (7.41%)
Men: SADs averted	0 (0-0)	3,000 (1,300-4,700)	240 (100-370)
LYG	0 (0-0)	61,000 (27,000-96,000)	4,800 (2,100-7,500)
Women: SADs averted	0 (0-0)	1,200 (530-1,900)	90 (40-140)
LYG	0 (0-0)	20,000 (8,900-31,000)	1,500 (660-2,300)

Notes: T21 = Tobacco 21; LYG = life-years gained; SADs = premature smoking-attributable deaths.

Parentheses indicate lower and upper-bound estimates using 95% confidence interval policy effects sizes.

2023 Census population estimate: 4,053,824

© 2025 Tam J et al. JAMA Health Forum.

© 2025 Tam J et al. JAMA Health Forum.

eFigure 90. Oregon T21 model outcomes with policy decay

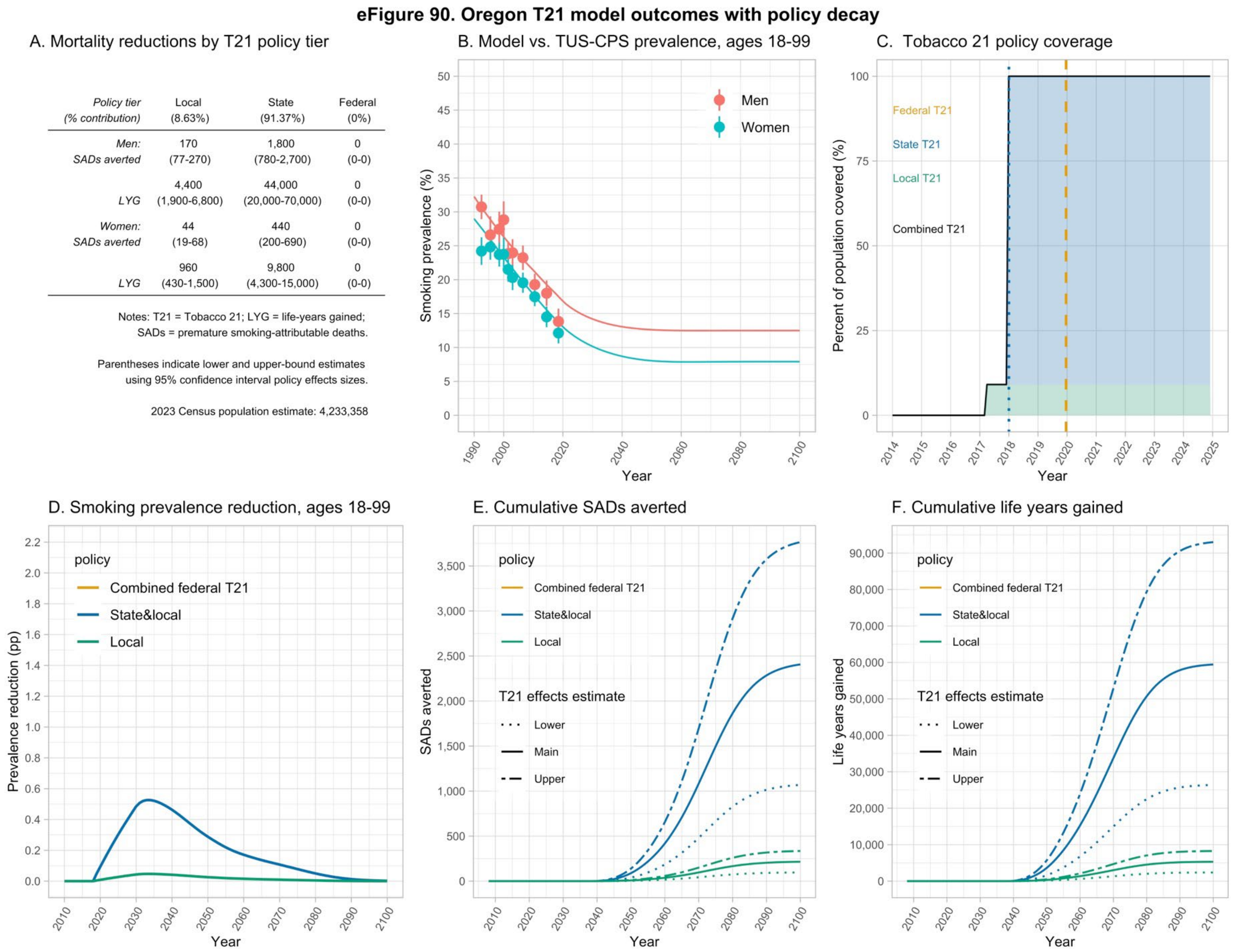

© 2025 Tam J et al. JAMA Health Forum.

© 2025 Tam J et al. JAMA Health Forum.

eFigure 91. Pennsylvania T21 model outcomes with policy decay

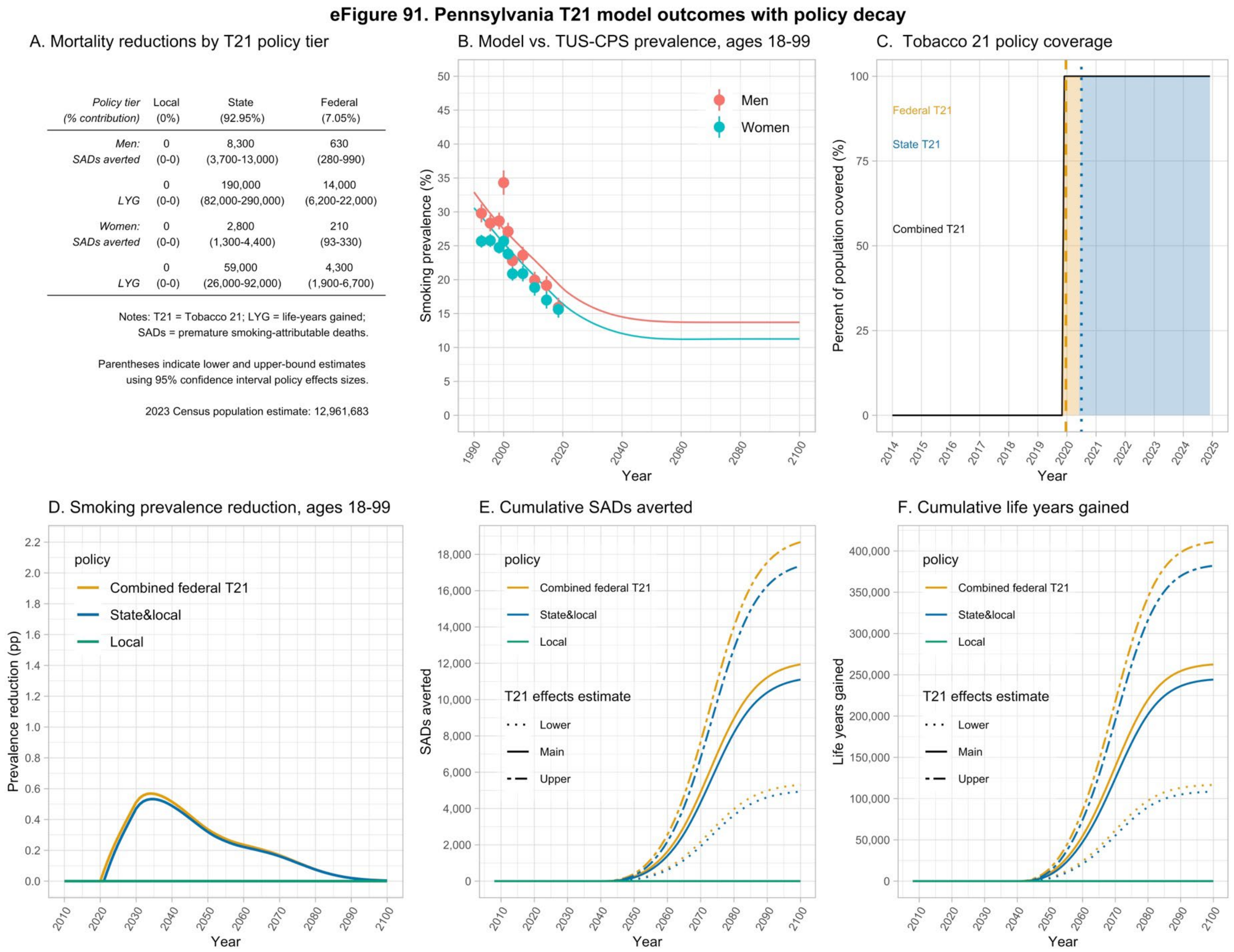

© 2025 Tam J et al. JAMA Health Forum.

© 2025 Tam J et al. JAMA Health Forum.

eFigure 92. Rhode Island T21 model outcomes with policy decay

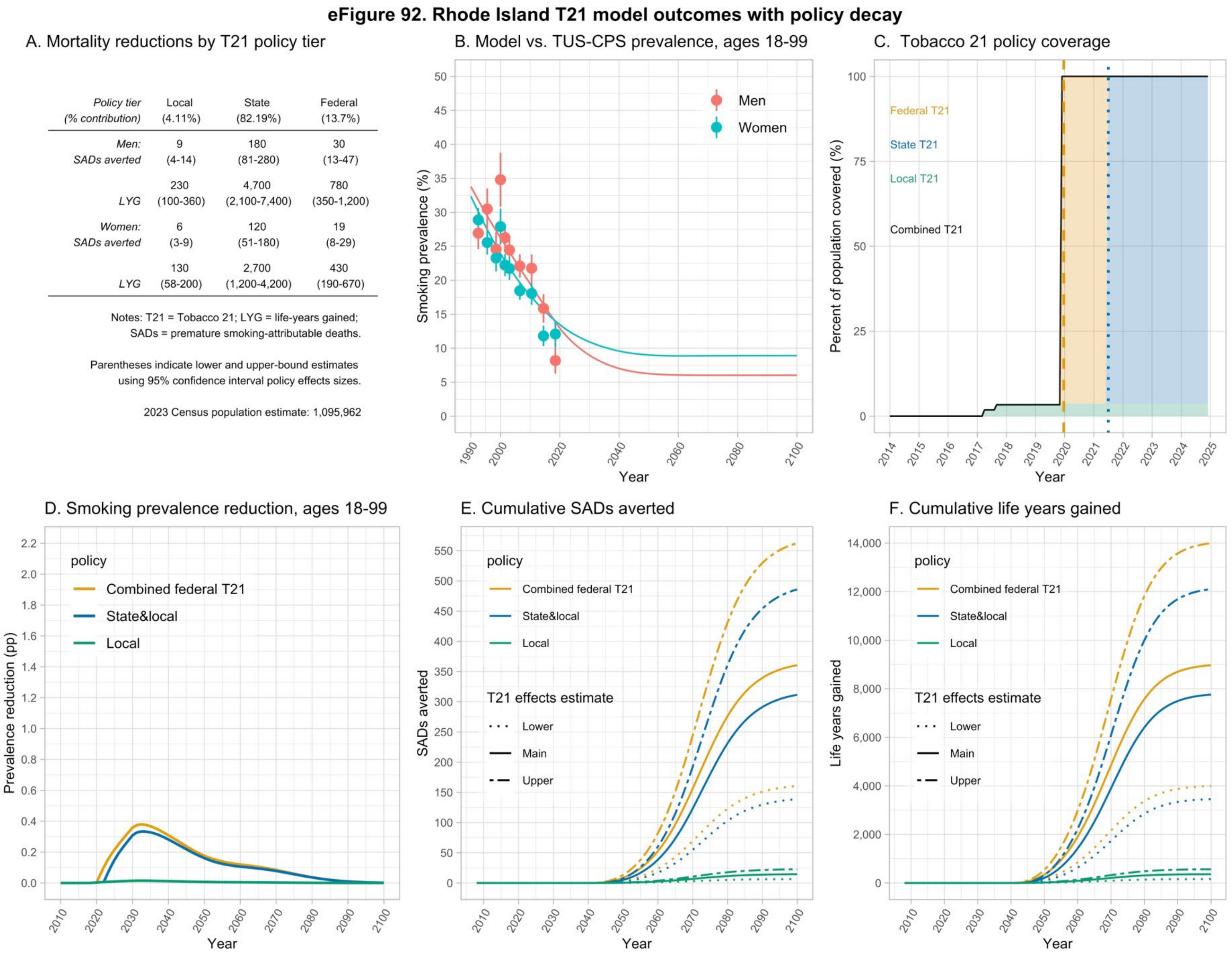

© 2025 Tam J et al. JAMA Health Forum.

© 2025 Tam J et al. JAMA Health Forum.

eFigure 93. South Carolina T21 model outcomes with policy decay

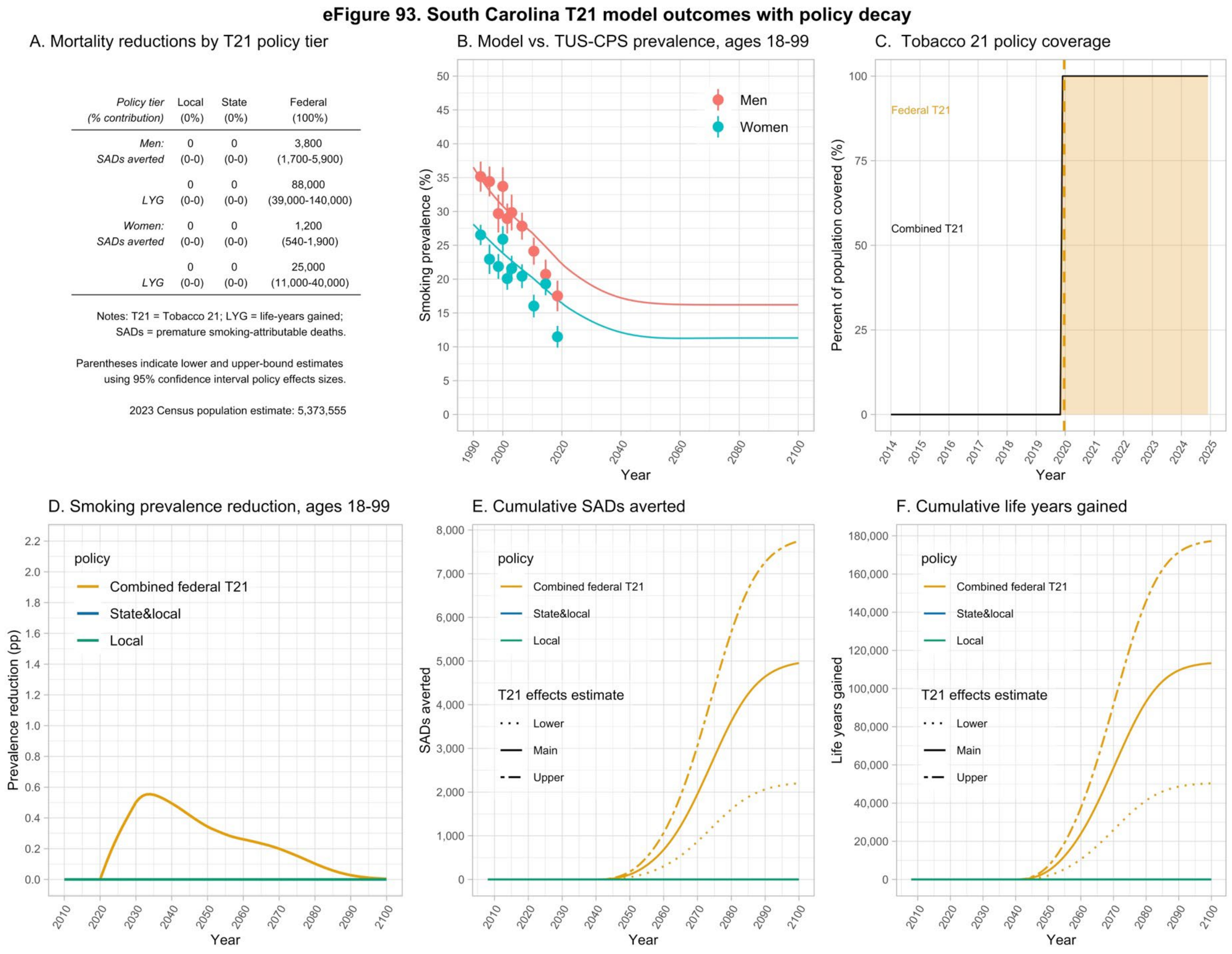

© 2025 Tam J et al. JAMA Health Forum.

© 2025 Tam J et al. JAMA Health Forum.

eFigure 94. South Dakota T21 model outcomes with policy decay

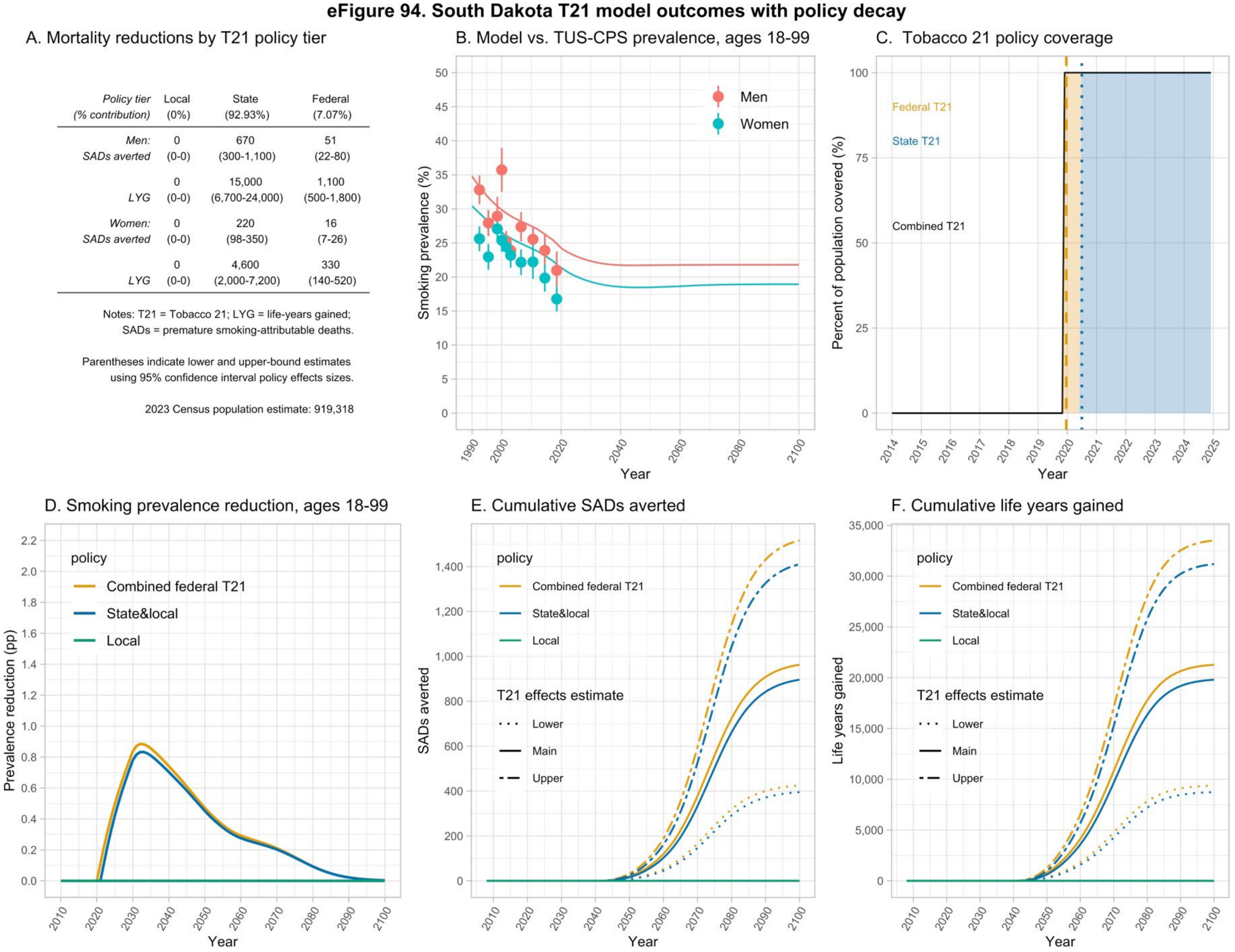

Policy tier (% contribution)	Local (0%)	State (92.93%)	Federal (7.07%)
Men: SADs averted	0 (0-0)	670 (300-1,100)	51 (22-80)
LYG	0 (0-0)	15,000 (6,700-24,000)	1,100 (500-1,800)
Women: SADs averted	0 (0-0)	220 (98-350)	16 (7-26)
LYG	0 (0-0)	4,600 (2,000-7,200)	330 (140-520)

Notes: T21 = Tobacco 21; LYG = life-years gained; SADs = premature smoking-attributable deaths.

Parentheses indicate lower and upper-bound estimates using 95% confidence interval policy effects sizes.

2023 Census population estimate: 919,318

© 2025 Tam J et al. JAMA Health Forum.

eFigure 95. Tennessee T21 model outcomes with policy decay

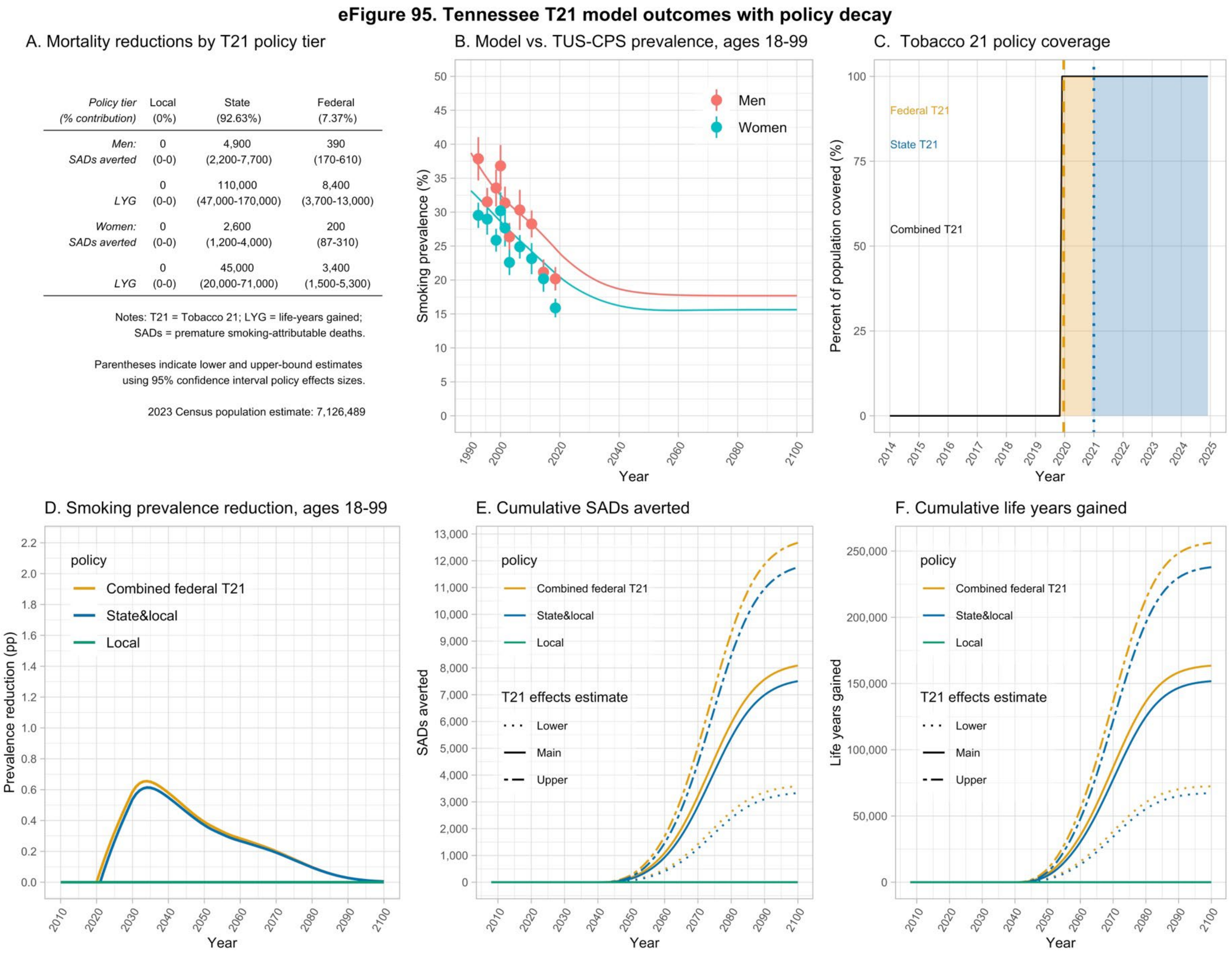

Policy tier (% contribution)	Local (0%)	State (92.63%)	Federal (7.37%)
Men: SADs averted	0 (0-0)	4,900 (2,200-7,700)	390 (170-610)
LYG	0 (0-0)	110,000 (47,000-170,000)	8,400 (3,700-13,000)
Women: SADs averted	0 (0-0)	2,600 (1,200-4,000)	200 (87-310)
LYG	0 (0-0)	45,000 (20,000-71,000)	3,400 (1,500-5,300)

Notes: T21 = Tobacco 21; LYG = life-years gained; SADs = premature smoking-attributable deaths.

Parentheses indicate lower and upper-bound estimates using 95% confidence interval policy effects sizes.

2023 Census population estimate: 7,126,489

© 2025 Tam J et al. JAMA Health Forum.

© 2025 Tam J et al. JAMA Health Forum.

eFigure 96. Texas T21 model outcomes with policy decay

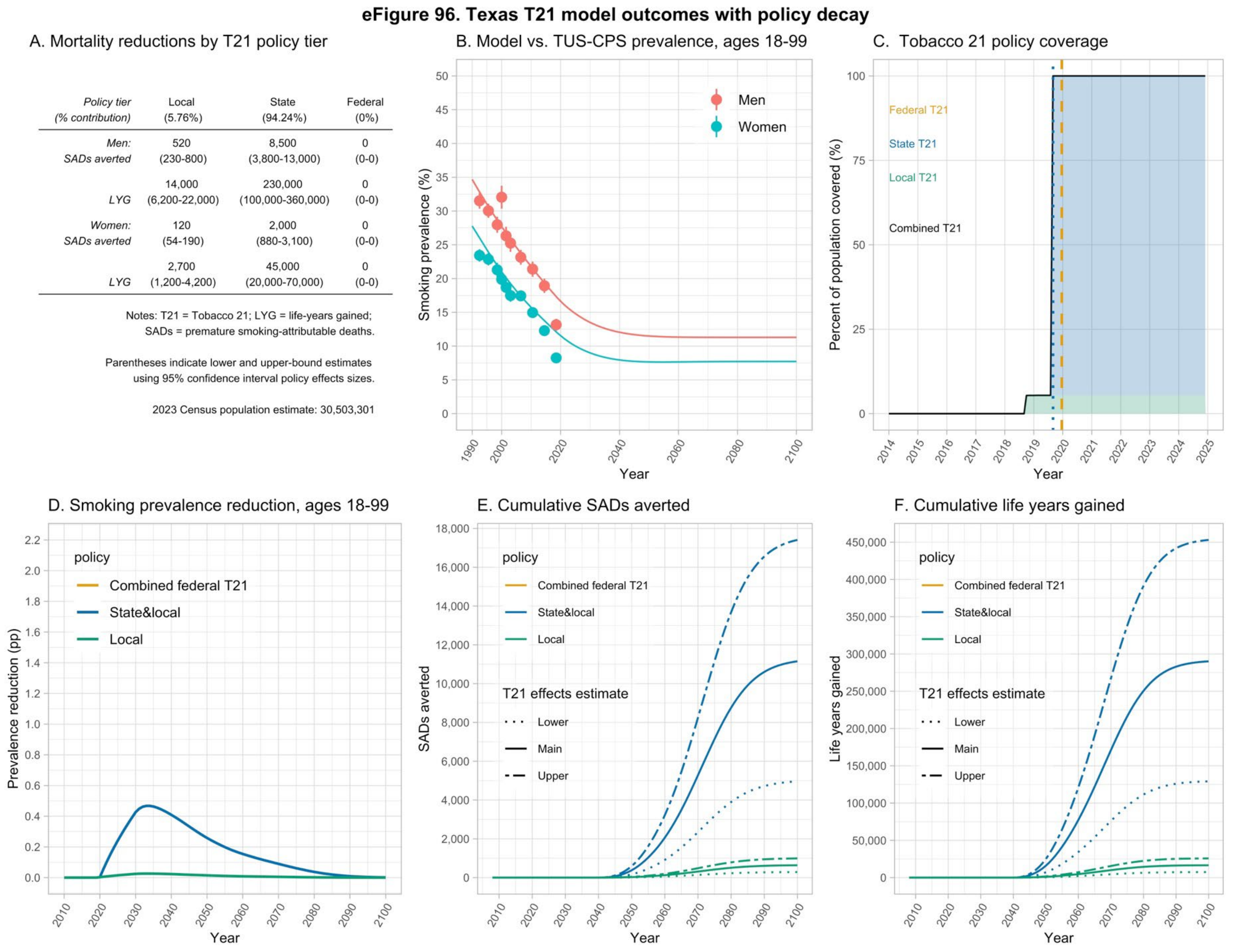

Policy tier (% contribution)	Local (5.76%)	State (94.24%)	Federal (0%)
Men: SADs averted	520 (230-800)	8,500 (3,800-13,000)	0 (0-0)
LYG	14,000 (6,200-22,000)	230,000 (100,000-360,000)	0 (0-0)
Women: SADs averted	120 (54-190)	2,000 (880-3,100)	0 (0-0)
LYG	2,700 (1,200-4,200)	45,000 (20,000-70,000)	0 (0-0)

Notes: T21 = Tobacco 21; LYG = life-years gained; SADs = premature smoking-attributable deaths.

Parentheses indicate lower and upper-bound estimates using 95% confidence interval policy effects sizes.

2023 Census population estimate: 30,503,301

© 2025 Tam J et al. JAMA Health Forum.

© 2025 Tam J et al. JAMA Health Forum.

eFigure 97. Utah T21 model outcomes with policy decay

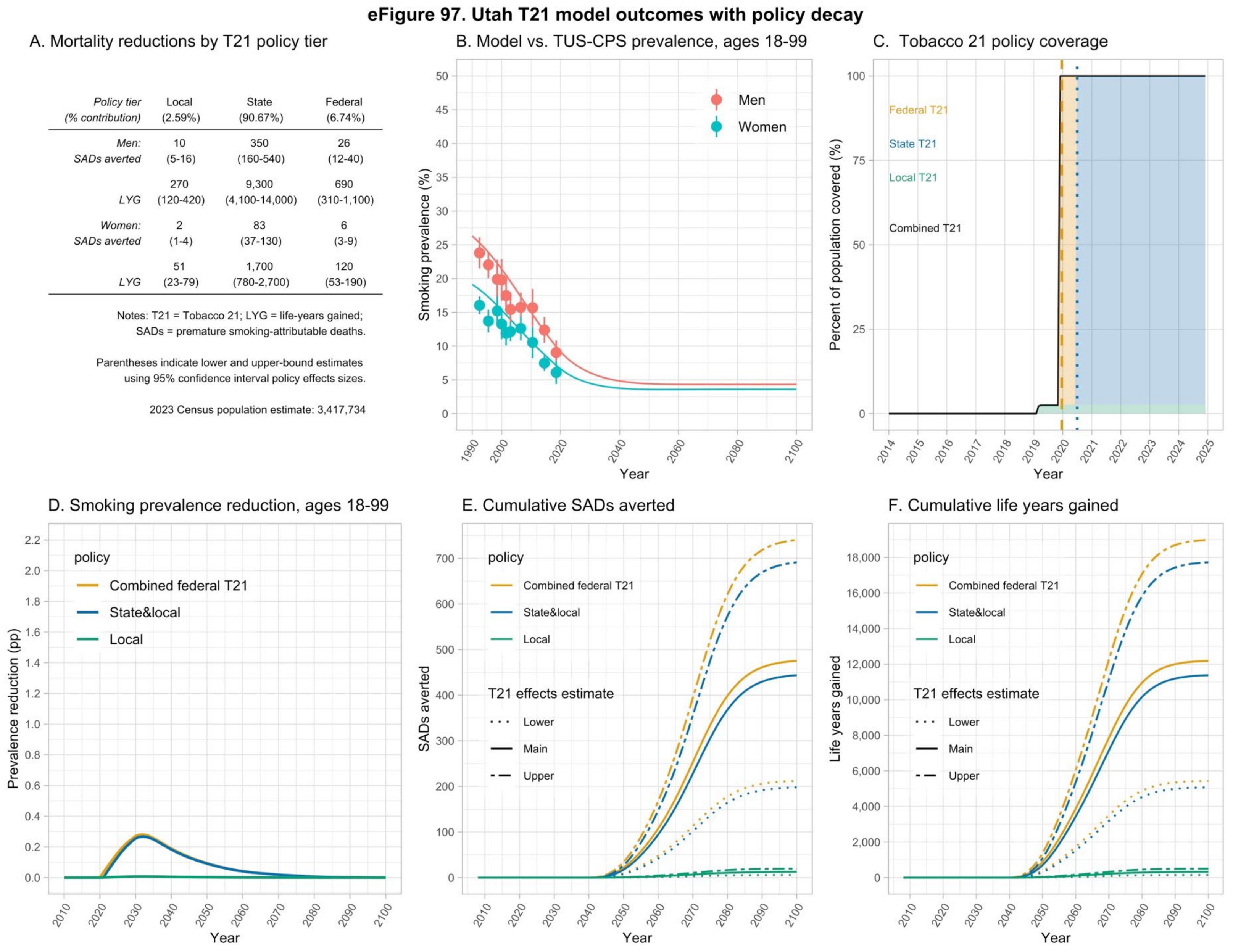

© 2025 Tam J et al. JAMA Health Forum.

eFigure 98. Vermont T21 model outcomes with policy decay

© 2025 Tam J et al. JAMA Health Forum.

eFigure 98. Vermont T21 model outcomes with policy decay

A. Mortality reductions by T21 policy tier

Policy tier (% contribution)	Local (0%)	State (100%)	Federal (0%)
Men: SADs averted	0 (0-0)	350 (160-550)	0 (0-0)
LYG	0 (0-0)	8,800 (3,900-14,000)	0 (0-0)
Women: SADs averted	0 (0-0)	100 (46-160)	0 (0-0)
LYG	0 (0-0)	2,200 (990-3,500)	0 (0-0)

Notes: T21 = Tobacco 21; LYG = life-years gained; SADs = premature smoking-attributable deaths.

Parentheses indicate lower and upper-bound estimates using 95% confidence interval policy effects sizes.

2023 Census population estimate: 647,464

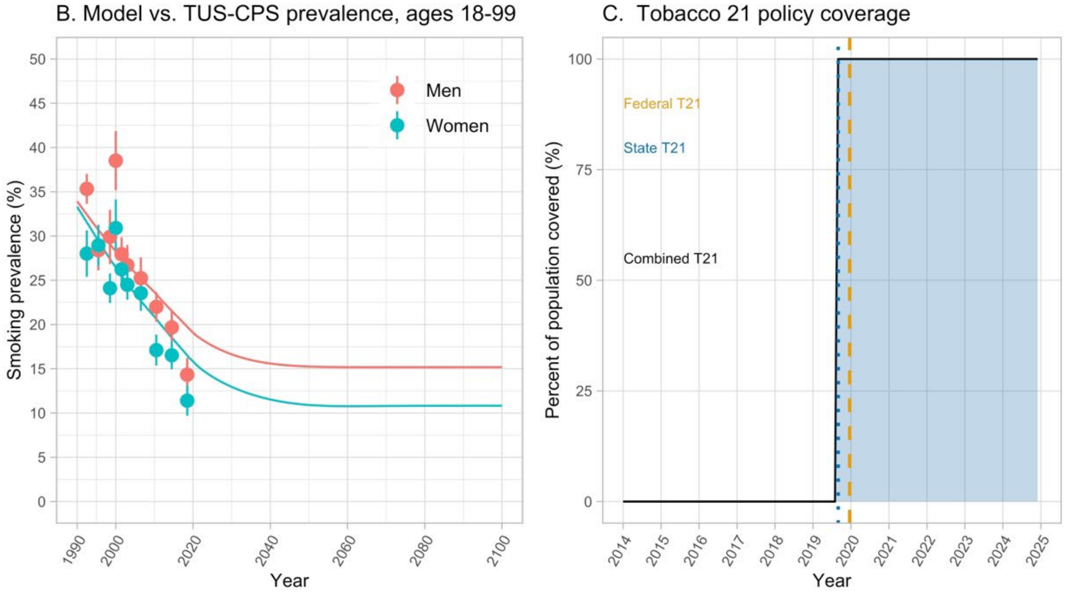

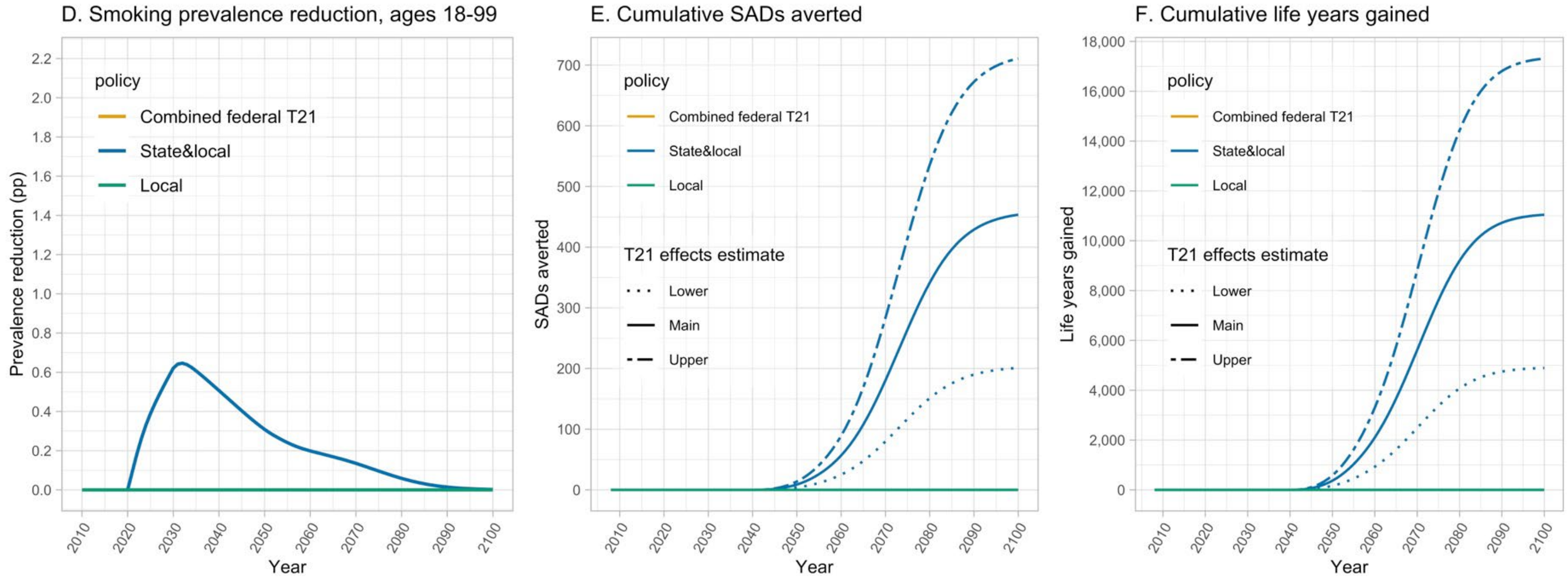

© 2025 Tam J et al. JAMA Health Forum.

eFigure 99. Virginia T21 model outcomes with policy decay

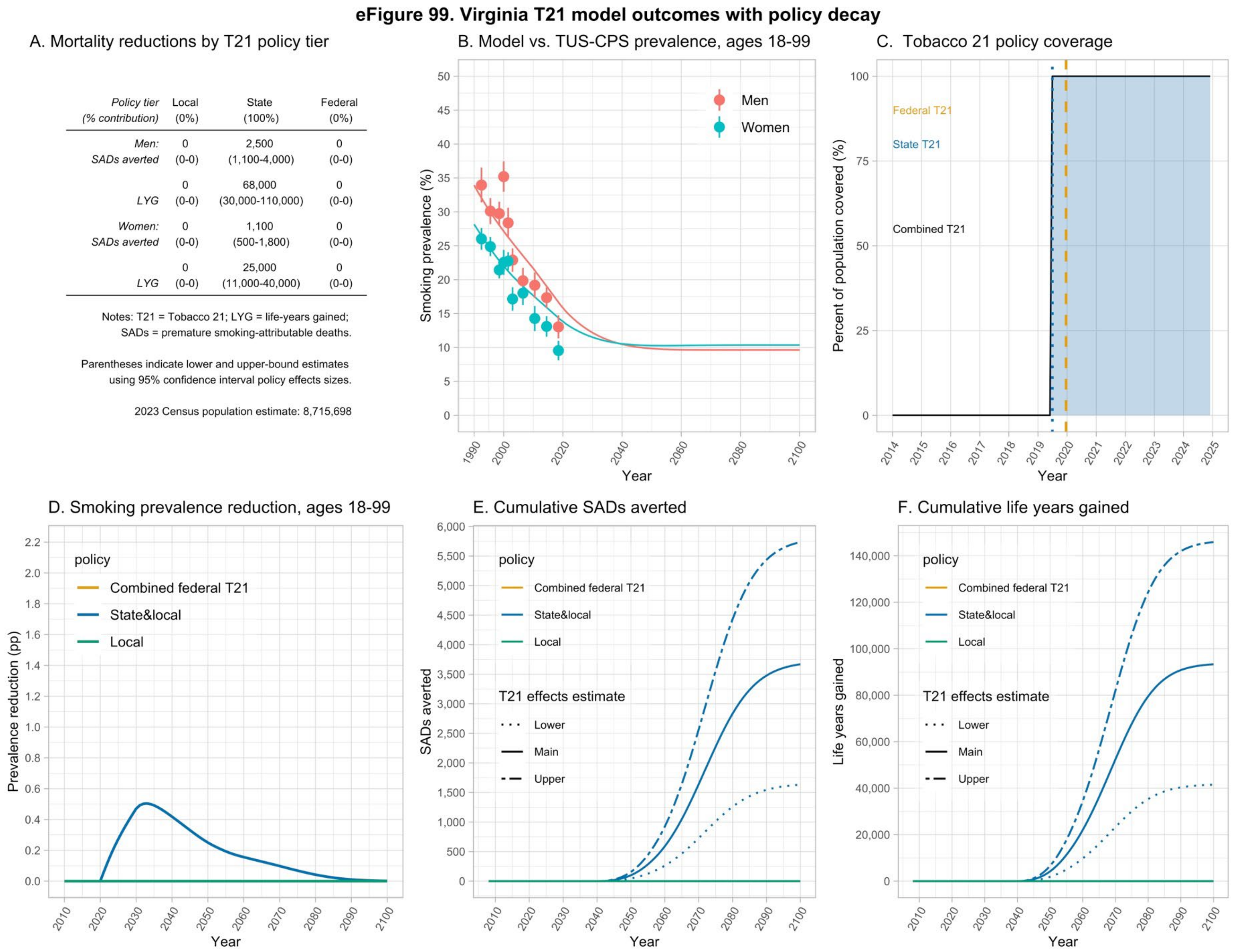

© 2025 Tam J et al. JAMA Health Forum.

© 2025 Tam J et al. JAMA Health Forum.

eFigure 100. Washington T21 model outcomes with policy decay

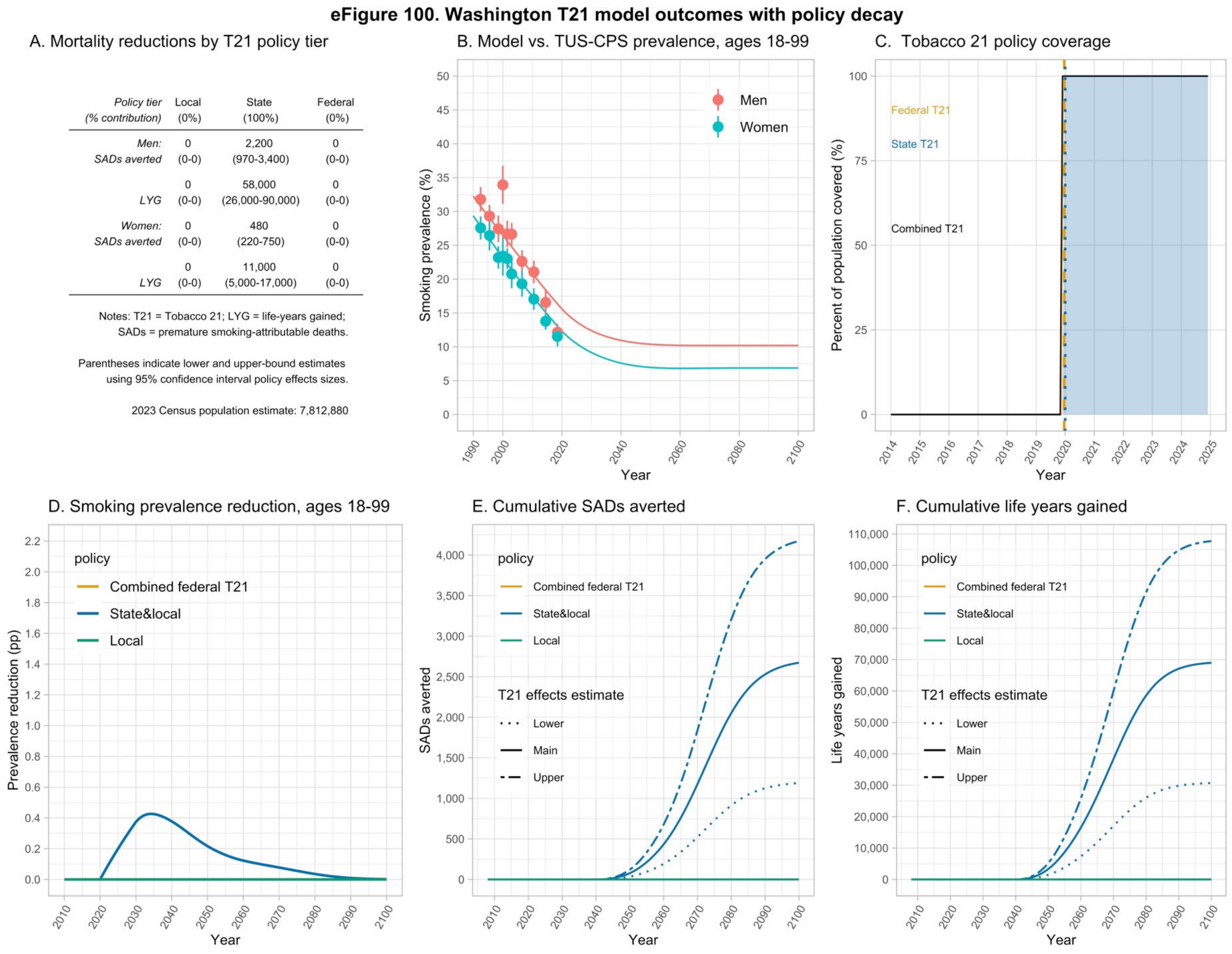

© 2025 Tam J et al. JAMA Health Forum.

© 2025 Tam J et al. JAMA Health Forum.

eFigure 101. West Virginia T21 model outcomes with policy decay

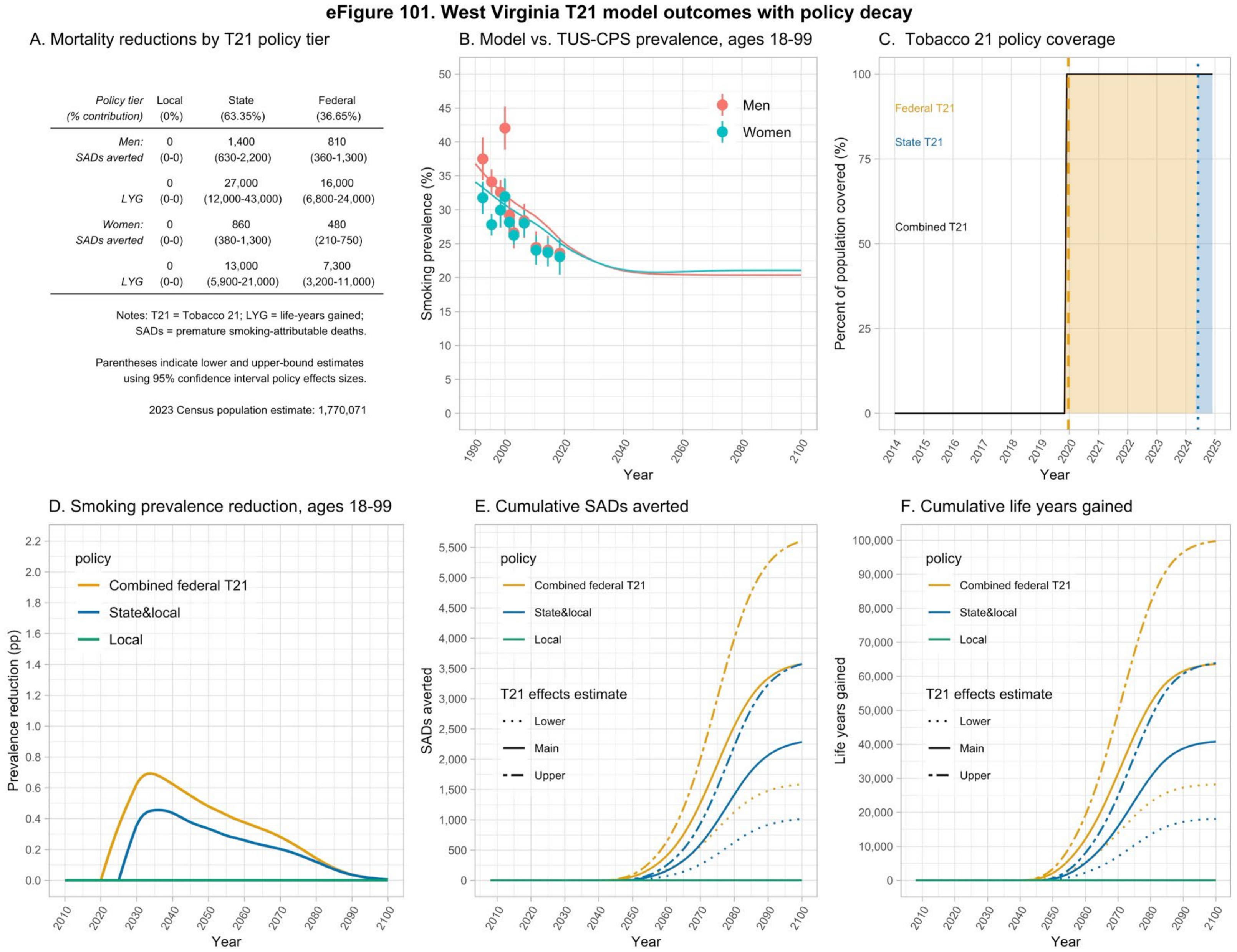

Policy tier (% contribution)	Local (0%)	State (63.35%)	Federal (36.65%)
Men: SADs averted	0 (0-0)	1,400 (630-2,200)	810 (360-1,300)
LYG	0 (0-0)	27,000 (12,000-43,000)	16,000 (6,800-24,000)
Women: SADs averted	0 (0-0)	860 (380-1,300)	480 (210-750)
LYG	0 (0-0)	13,000 (5,900-21,000)	7,300 (3,200-11,000)

Notes: T21 = Tobacco 21; LYG = life-years gained; SADs = premature smoking-attributable deaths.

Parentheses indicate lower and upper-bound estimates using 95% confidence interval policy effects sizes.

2023 Census population estimate: 1,770,071

© 2025 Tam J et al. JAMA Health Forum.

© 2025 Tam J et al. JAMA Health Forum.

eFigure 102. Wisconsin T21 model outcomes with policy decay

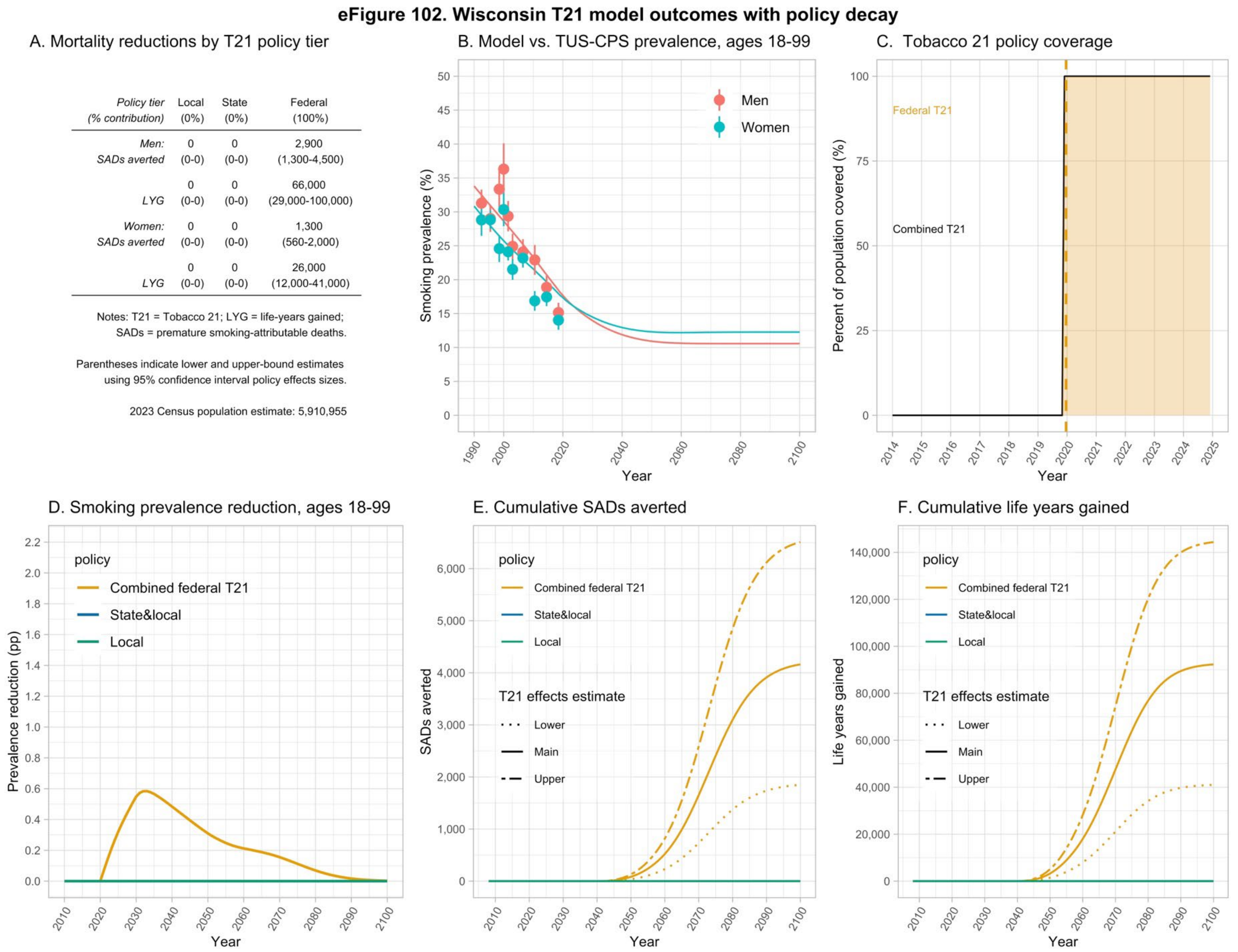

© 2025 Tam J et al. JAMA Health Forum.

© 2025 Tam J et al. JAMA Health Forum.

eFigure 103. Wyoming T21 model outcomes with policy decay

eFigure 103. Wyoming T21 model outcomes with policy decay

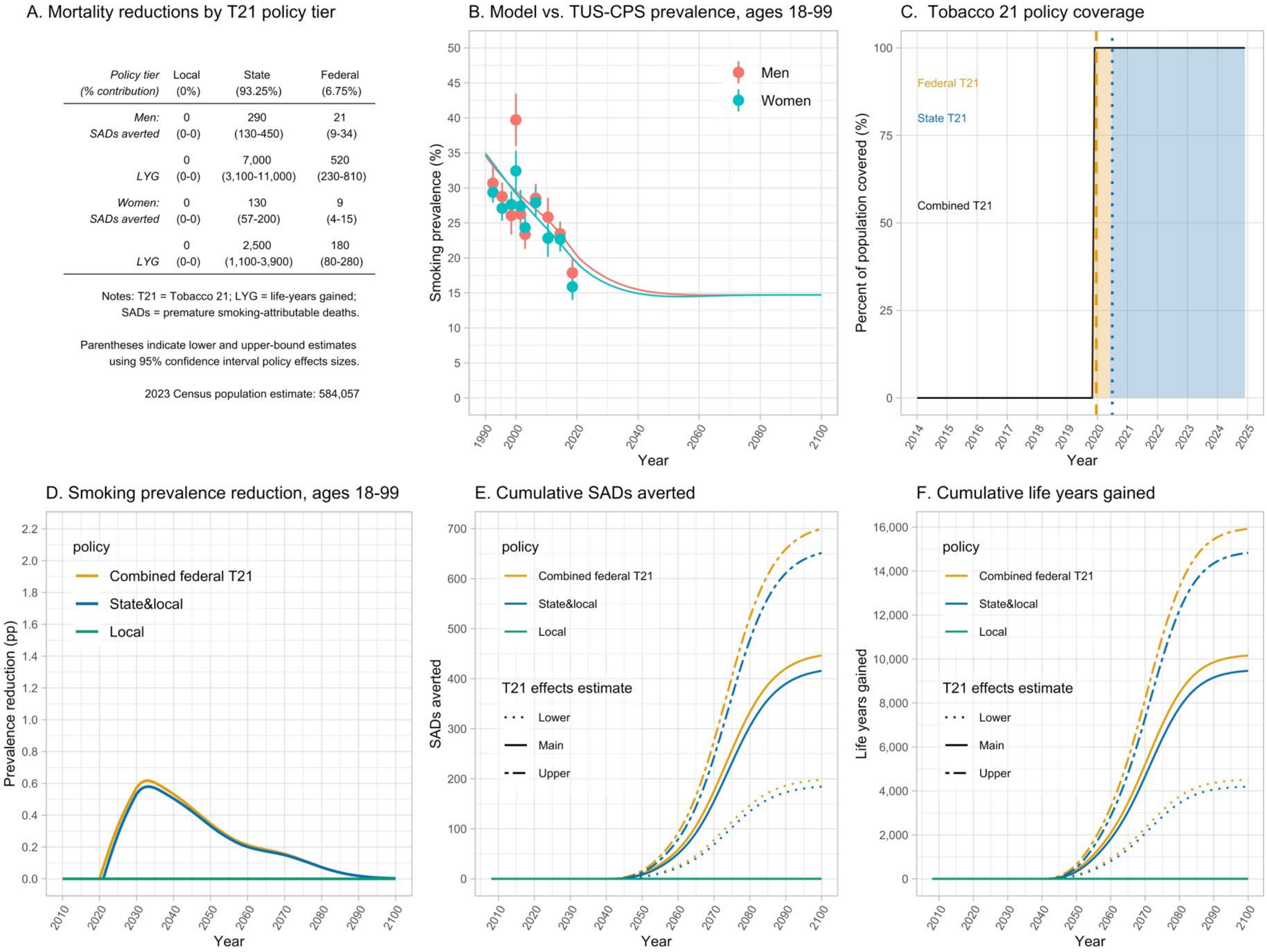

© 2025 Tam J et al. JAMA Health Forum.

© 2025 Tam J et al. JAMA Health Forum.

References

1. Holford TR, McKay L, Jeon J, et al. Smoking Histories by State in the US. *Am J Prev Med*. 2023;64(4)(1):s42-s52. doi:10.1016/j.amepre.2022.08.018

2. Holford TR, Meza R, Warner KE, et al. Tobacco control and the reduction in smoking-related premature deaths in the United States, 1964-2012. *JAMA*. Jan 08 2014;311(2):164-71. doi:10.1001/jama.2013.285112

3. Jeon J, Holford TR, Levy DT, et al. Smoking and Lung Cancer Mortality in the United States From 2015 to 2065: A Comparative Modeling Approach. *Ann Intern Med*. 2018;169(10):684-693. doi:10.7326/M18-1250

4. Tam J, Jeon J, Thrasher JF, et al. Estimated Prevalence of Smoking and Smoking-Attributable Mortality Associated with Graphic Health Warnings on Cigarette Packages in the US From 2022 to 2100. *JAMA Health Forum*. 2021;2(9):e212852. doi:10.1001/jamahealthforum.2021.2852

5. Tam J, Levy DT, Jeon J, et al. Projecting the effects of tobacco control policies in the USA through microsimulation: a study protocol. *BMJ Open*. 2018;8(3)doi:10.1136/bmjopen-2017-019169

6. Le TT, Mendez D. An estimation of the harm of menthol cigarettes in the United States from 1980 to 2018. *Tobacco Control*. 2022;31(4):564-568. doi:10.1136/tobaccocontrol-2020-056256

7. Meza R, Cao P, Jeon J, Warner KE, Levy DT. Trends in US Adult Smoking Prevalence, 2011 to 2022. *JAMA Health Forum*. 2023;4(12):e234213-e234213. doi:10.1001/jamahealthforum.2023.4213

8. Levy DT, Warner KE, Cummings KM, et al. Examining the relationship of vaping to smoking initiation among US youth and young adults: a reality check. *Tob Control*. Nov 20 2019;28(6):629-635. doi:10.1136/tobaccocontrol-2018-054446

9. Wang X, Kim Y, Borowiecki M, Tynan MA, Emery S, King BA. Trends in Cigar Sales and Prices, by Product and Flavor Type—the United States, 2016–2020. *Nicotine & Tobacco Research*. 2021;24(4):606-611. doi:10.1093/ntr/ntab238

10. Delnevo CD, Miller Lo E, Giovenco DP, Cornacchione Ross J, Hrywna M, Strasser AA. Cigar Sales in Convenience Stores in the US, 2009-2020. *JAMA*. 2021;326(23):2429-2432. doi:10.1001/jama.2021.19692

11. National Center for Health Statistics. Data from: Vital Statistics Online Data Portal, Mortality Multiple Cause Files. 2021.

12. Surveillance, Epidemiology, and End Results (SEER) Program. SEER*Stat Database: Incidence - SEER 9 Regs Research Data, Nov 2011 Sub, Vintage 2009 Pops (1973-2009) <Katrina/Rita Population Adjustment> - Linked To County Attributes - Total U.S., 1969-2010 Counties, National Cancer Institute, DCCPS, Surveillance Research Program, Surveillance Systems Branch, released April 2012, based on the November 2011 submission.

13. Gompertz B. XXIV. On the nature of the function expressive of the law of human mortality, and on a new mode of determining the value of life contingencies. In a letter to Francis Baily, Esq. FRS &c. *Philosophical transactions of the Royal Society of London*. 1825;(115):513-583.

14. Rosenberg MA, Feuer EJ, Yu B, et al. Chapter 3: Cohort Life Tables by Smoking Status, Removing Lung Cancer as a Cause of Death. *Risk Analysis*. 2012;32:S25-S38. doi:10.1111/j.1539-6924.2011.01662.x

15. United States Census Bureau. State Population by Characteristics: 2020-2023. Updated December 18, 2023. Accessed December 20, 2023. https://www.census.gov/data/tables/time-series/demo/popest/2020s-state-detail.html

© 2025 Tam J et al. JAMA Health Forum.

16. United States Census Bureau. State Population by Characteristics: 2010-2019. Updated October 8, 2021. Accessed December 20, 2023. https://www.census.gov/data/tables/time-series/demo/popest/2010s-state-detail.html

17. Cancer Intervention Surveillance Modeling Network (CISNET) Lung Group. CISNET Lung Working Group Smoking Parameters. Accessed September 11, 2024. https://apps.cisnetsmokingparameters.org/states/

18. Cancer Intervention Surveillance Modeling Network (CISNET) Lung Group. Cohort Mortality by State - T21 analysis. Updated September 5. Accessed September 11, 2024. https://sph-umich.shinyapps.io/shgdisplayappmortstate_t21/

19. Friedman AS, Wu RJ. Do Local Tobacco-21 Laws Reduce Smoking among 18 to 20 Year-Olds? *Nicotine Tob Res*. Jul 26 2020;22(7):1195-1201. doi:10.1093/ntr/ntz123

20. Hansen B, Sabia JJ, McNichols D, Bryan C. Do tobacco 21 laws work? *J Health Econ*. Dec 2023;92:102818. doi:10.1016/j.jhealeco.2023.102818

21. Abouk R, De PK, Pesko MF. Estimating the effects of tobacco-21 on youth tobacco use and sales. *Journal of Health Economics*. 2024/03/01/ 2024;94:102860. doi:https://doi.org/10.1016/j.jhealeco.2024.102860

22. IOM (Institute of Medicine). *Health Implications of Raising the Minimum Age for Purchasing Tobacco Products*. 2015. http://www.iom.edu/Activities/PublicHealth/TobaccoMinimumAge.aspx

23. Holford TR, Levy DT, McKay LA, et al. Patterns of birth cohort-specific smoking histories, 1965-2009. *Am J Prev Med*. Feb 2014;46(2):e31-7. doi:10.1016/j.amepre.2013.10.022

24. Colston DC, Vander Woude CA, Cohen A, et al. Data from: Tobacco 21 Population Coverage Database, 2014-2020. Version 2.0. 2022;2.0. *Ann Arbor, MI*. Deposited April 5, 2022.

© 2025 Tam J et al. JAMA Health Forum.
