# Supplementary material for: US Tobacco 21 Policies and Potential Mortality Reductions by State
Source: JAMA Health Forum. 2024 Dec 20;5(12):e244445. doi: 10.1001/jamahealthforum.2024.4445 (PMC11662258; doi:10.1001/jamahealthforum.2024.4445)
Supplement: Supplement 2. — Data Sharing Statement [file jamahealthforum-e244445-s002.pdf]

Data Sharing Statement

Tam. US Tobacco 21 Policies and Potential Mortality Reductions by State. *JAMA Health Forum*. Published December 20, 2024. doi:10.1001/jamahealthforum.2024.4445

Data
Data available: No

Additional Information
Explanation for why data not available: All survey data used in the analysis are from publicly available datasets. Model code and related data are available at https://github.com/NCI-CISNET/tcp-model-code.
